# Supplementary material for: Proteomic Analysis of Irradiation with Millimeter Waves on Soybean Growth under Flooding Conditions
Source: Int J Mol Sci. 2020 Jan 12;21(2):486. doi: 10.3390/ijms21020486 (PMC7013696; doi:10.3390/ijms21020486)
Supplement: Supplementary file 1 [file ijms-21-00486-s001.zip › rev Supplemental_Tables.docx]

**Table S1**. List of changed proteins between soybean seedlings irradiated with or without millimeter waves at the starting point.

|  | accessiona | description | M.P.b | fold changec | MW in kDa | pI |
| --- | --- | --- | --- | --- | --- | --- |
| 1 | I1J7P1 | non-specific lipid-transfer protein | 5 | 8.98 | 11.8 | 9.18 |
| 2 | C6TFY7 | uncharacterized protein | 3 | 5.47 | 10.6 | 7.99 |
| 3 | A0A0R4J2V8 | dirigent protein | 3 | 5.32 | 23.9 | 5.85 |
| 4 | I1LH06 | uncharacterized protein | 4 | 4.60 | 50.3 | 5.96 |
| 5 | C6SZA9 | uncharacterized protein | 6 | 4.55 | 17.1 | 5.10 |
| 6 | Q70EM0 | dehydrin | 9 | 4.38 | 23.8 | 5.97 |
| 7 | C6T4K7 | uncharacterized protein | 2 | 4.32 | 20.3 | 5.14 |
| 8 | I1JDS6 | beta-hexosaminidase | 11 | 4.17 | 62.9 | 5.54 |
| 9 | I1N4J8 | uncharacterized protein | 4 | 4.10 | 25.5 | 4.72 |
| 10 | Q9XFI6 | peroxidase | 6 | 3.99 | 37.4 | 8.54 |
| 11 | A0A0R0KFE8 | chlorophyll a-b binding protein | 2 | 3.87 | 31.4 | 5.90 |
| 12 | Q2PMN3 | photosystem i iron-sulfur center | 3 | 3.84 | 9.0 | 6.65 |
| 13 | A5A339 | endonuclease | 3 | 3.83 | 33.5 | 5.92 |
| 14 | G9I8U0 | inositol methyltransferase | 14 | 3.82 | 41.5 | 5.93 |
| 15 | K7N2X4 | uncharacterized protein | 15 | 3.78 | 53.4 | 6.62 |
| 16 | I1NEF3 | uncharacterized protein | 2 | 3.66 | 64.7 | 9.02 |
| 17 | K7KNI8 | uncharacterized protein | 3 | 3.65 | 102.8 | 9.15 |
| 18 | I1L7P6 | uncharacterized protein | 2 | 3.59 | 38.8 | 8.99 |
| 19 | I1L0D9 | peroxidase | 7 | 3.53 | 38.0 | 6.58 |
| 20 | I1KV43 | uncharacterized protein | 2 | 3.53 | 25.3 | 5.69 |
| 21 | Q9MAZ1 | nonclathrin coat protein zeta2-cop | 3 | 3.53 | 19.8 | 4.74 |
| 22 | I1K8M1 | uncharacterized protein | 2 | 3.52 | 19.2 | 7.95 |
| 23 | C6TC69 | uncharacterized protein | 3 | 3.47 | 42.0 | 5.91 |
| 24 | I1JHE4 | uncharacterized protein | 19 | 3.36 | 65.6 | 6.01 |
| 25 | A0A0R0KDG4 | uncharacterized protein | 7 | 3.25 | 26.2 | 5.38 |
| 26 | C6SX14 | ferredoxin | 3 | 3.21 | 16.5 | 6.41 |
| 27 | I1NE45 | lipoxygenase | 2 | 3.21 | 102.8 | 5.93 |
| 28 | C6T1V4 | nucleoside diphosphate kinase | 2 | 3.21 | 25.5 | 9.16 |
| 29 | A6BM07 | isoflavone 7-o-glucosyltransferase 1 | 3 | 3.19 | 52.0 | 4.98 |
| 30 | K7KID6 | uncharacterized protein | 2 | 3.12 | 24.4 | 8.93 |
| 31 | K7KQW5 | uncharacterized protein | 3 | 3.06 | 24.6 | 4.99 |
| 32 | C6T6Y4 | uncharacterized protein | 4 | 3.04 | 31.2 | 9.31 |
| 33 | K7M5U1 | uncharacterized protein | 6 | 3.00 | 103.8 | 6.46 |
| 34 | C6TEX2 | chlorophyll a-b binding protein | 3 | 2.99 | 29.8 | 8.60 |
| 35 | I1MNR2 | xyloglucan endotransglucosylase/hydrolase | 8 | 2.98 | 25.4 | 6.31 |
| 36 | A0A0R0K0E5 | uncharacterized protein | 4 | 2.98 | 59.2 | 7.78 |
| 37 | A0A0R0J324 | uncharacterized protein | 2 | 2.93 | 25.7 | 7.12 |
| 38 | I1JSC6 | uncharacterized protein | 5 | 2.92 | 28.8 | 10.44 |
| 39 | K7LPM5 | uncharacterized protein | 6 | 2.86 | 113.4 | 5.85 |
| 40 | C6TBI8 | uncharacterized protein | 3 | 2.86 | 24.8 | 7.01 |
| 41 | I1NFX6 | uncharacterized protein | 2 | 2.80 | 28.4 | 5.92 |
| 42 | A0A0R0KYU7 | xyloglucan endotransglucosylase/hydrolase | 9 | 2.79 | 36.0 | 7.18 |
| 43 | I1N036 | proliferating cell nuclear antigen | 9 | 2.77 | 29.5 | 4.68 |
| 44 | I1LBJ5 | zeta-carotene desaturase | 3 | 2.75 | 62.9 | 7.92 |
| 45 | C6TLM4 | chlorophyll a-b binding protein | 6 | 2.74 | 27.9 | 5.29 |
| 46 | I1MTI7 | uncharacterized protein | 7 | 2.72 | 49.7 | 8.32 |
| 47 | K7MP10 | uncharacterized protein | 2 | 2.72 | 118.6 | 4.97 |
| 48 | C6SY81 | uncharacterized protein | 3 | 2.70 | 15.1 | 9.33 |
| 49 | A0A0R0EIR6 | glycosyltransferase | 8 | 2.63 | 54.4 | 5.60 |
| 50 | I1N1U5 | glycosyltransferase | 9 | 2.60 | 53.3 | 5.44 |
| 51 | I1KXZ9 | uncharacterized protein | 5 | 2.56 | 43.2 | 5.36 |
| 52 | I1J4K5 | uncharacterized protein | 3 | 2.53 | 19.4 | 4.83 |
| 53 | K7MA29 | uncharacterized protein | 6 | 2.52 | 104.4 | 6.30 |
| 54 | I1JE21 | glycosyltransferase | 2 | 2.51 | 54.2 | 5.31 |
| 55 | I1L327 | uncharacterized protein | 2 | 2.48 | 27.2 | 6.90 |
| 56 | C6TJ75 | peroxidase | 2 | 2.46 | 34.2 | 4.78 |
| 57 | K7K6S5 | uncharacterized protein | 5 | 2.45 | 29.3 | 5.39 |
| 58 | I1LN34 | uncharacterized protein | 21 | 2.43 | 53.1 | 5.74 |
| 59 | I1MMM3 | uncharacterized protein | 4 | 2.40 | 23.3 | 6.58 |
| 60 | C6SZJ5 | uncharacterized protein | 2 | 2.36 | 26.3 | 9.26 |
| 61 | P02957 | photosystem ii protein d1 | 3 | 2.35 | 38.9 | 5.21 |
| 62 | Q2PMP8 | 50s ribosomal protein l16 | 2 | 2.28 | 15.3 | 11.62 |
| 63 | C6TD35 | uncharacterized protein | 2 | 2.27 | 37.0 | 5.20 |
| 64 | I1KUN7 | uncharacterized protein | 5 | 2.27 | 40.2 | 6.10 |
| 65 | C6TLI1 | chlorophyll a-b binding protein | 3 | 2.26 | 27.4 | 6.91 |
| 66 | I1JT46 | uncharacterized protein | 2 | 2.24 | 14.6 | 5.56 |
| 67 | I1LBX5 | uncharacterized protein | 2 | 2.23 | 44.7 | 7.79 |
| 68 | I1KIQ3 | uncharacterized protein | 2 | 2.23 | 70.8 | 8.35 |
| 69 | I1MUF2 | uncharacterized protein | 8 | 2.20 | 282.2 | 5.78 |
| 70 | A0A0R0HYB1 | uncharacterized protein | 9 | 2.17 | 51.6 | 8.58 |
| 71 | I1LTU5 | uncharacterized protein | 4 | 2.12 | 48.5 | 7.04 |
| 72 | A0A0R4J645 | uncharacterized protein | 2 | 2.11 | 41.6 | 5.26 |
| 73 | I1MQU2 | uncharacterized protein | 4 | 2.05 | 28.4 | 5.15 |
| 74 | C6TBY2 | uncharacterized protein | 3 | 2.05 | 17.7 | 5.62 |
| 75 | A0A0R0IF33 | uncharacterized protein | 16 | 1.95 | 61.6 | 4.77 |
| 76 | A0A0R0HCH7 | uncharacterized protein | 4 | 1.95 | 58.7 | 5.59 |
| 77 | Q2PMU3 | photosystem i p700 chlorophyll a apoprotein a1 | 2 | 1.92 | 83.4 | 6.74 |
| 78 | I1LDJ1 | pyruvate kinase | 12 | 1.92 | 63.7 | 7.31 |
| 79 | I1JFJ9 | uncharacterized protein | 11 | 1.90 | 31.9 | 6.51 |
| 80 | A0A0R0G789 | uncharacterized protein | 18 | 1.87 | 56.1 | 9.12 |
| 81 | A0A0R0KAK4 | uncharacterized protein | 2 | 1.86 | 44.0 | 7.56 |
| 82 | Q2PMU9 | ATP synthase epsilon chain | 3 | 1.79 | 14.7 | 5.41 |
| 83 | Q2PMT8 | photosystem ii d2 protein | 5 | 1.72 | 39.5 | 5.33 |
| 84 | Q43437 | chlorophyll a-b binding protein | 8 | 1.70 | 28.0 | 5.14 |
| 85 | K7MTZ4 | uncharacterized protein | 3 | 1.68 | 41.0 | 6.44 |
| 86 | I1JMX7 | uncharacterized protein | 5 | 1.67 | 49.0 | 8.37 |
| 87 | C6TMK9 | uncharacterized protein | 3 | 1.67 | 34.0 | 6.00 |
| 88 | I1MLA1 | uncharacterized protein | 3 | 1.65 | 55.0 | 9.11 |
| 89 | I1K9B3 | uncharacterized protein | 4 | 1.65 | 48.2 | 8.85 |
| 90 | K7K8T8 | uncharacterized protein | 6 | 1.65 | 21.0 | 9.40 |
| 91 | A0A0R0GFB2 | uncharacterized protein | 23 | 1.64 | 57.5 | 8.99 |
| 92 | A0A0R0HHM1 | uncharacterized protein | 2 | 1.64 | 88.6 | 5.89 |
| 93 | I1JYM6 | uncharacterized protein | 4 | 1.64 | 50.5 | 6.34 |
| 94 | Q2TUV8 | anthocyanidin synthase 2 | 5 | 1.62 | 39.9 | 5.62 |
| 95 | C6T064 | 40s ribosomal protein s12 | 8 | 1.61 | 14.9 | 5.50 |
| 96 | C6TCI6 | glutamate decarboxylase | 12 | 1.60 | 56.2 | 5.57 |
| 97 | I1KUQ9 | glycylpeptide n-tetradecanoyltransferase | 13 | 1.60 | 49.9 | 6.07 |
| 98 | P10743 | stem 31 kda glycoprotein | 21 | 1.59 | 29.3 | 5.84 |
| 99 | I1MNI2 | uncharacterized protein | 3 | 1.59 | 39.6 | 6.33 |
| 100 | C6TLX4 | uncharacterized protein | 10 | 1.58 | 35.1 | 5.53 |
| 101 | I1L2X8 | pyruvate kinase | 12 | 1.57 | 63.3 | 7.28 |
| 102 | I1KYC6 | uncharacterized protein | 3 | 1.57 | 51.9 | 8.14 |
| 103 | I1JKE2 | uncharacterized protein | 5 | 1.56 | 67.0 | 8.93 |
| 104 | P15490 | stem 28 kda glycoprotein | 13 | 1.55 | 29.0 | 8.38 |
| 105 | A0A0R0GNH6 | uncharacterized protein | 6 | 1.55 | 50.5 | 6.64 |
| 106 | C6SX81 | ferredoxin | 2 | 1.54 | 15.5 | 4.66 |
| 107 | I1ML46 | uncharacterized protein | 13 | 1.53 | 56.5 | 5.00 |
| 108 | K7LPZ3 | uncharacterized protein | 2 | 1.52 | 8.8 | 4.27 |
| 109 | Q2PMU2 | photosystem i p700 chlorophyll a apoprotein a2 | 8 | 1.52 | 82.4 | 6.80 |
| 110 | I1KKN0 | uncharacterized protein | 6 | 1.51 | 140.7 | 4.49 |
| 111 | A0A0R0EZU8 | uncharacterized protein | 4 | 1.50 | 25.9 | 5.64 |
| 112 | I1M8D6 | uncharacterized protein | 4 | 1.50 | 51.2 | 4.98 |
| 113 | I1M712 | oxygen-evolving enhancer protein 2 | 3 | 1.50 | 28.6 | 7.69 |
| 114 | C6TMX0 | xyloglucan endotransglucosylase/hydrolase | 8 | 1.49 | 34.4 | 9.12 |
| 115 | A0A0R0GWU7 | uncharacterized protein | 4 | 1.49 | 29.3 | 9.57 |
| 116 | C6T9R1 | uncharacterized protein | 4 | 1.49 | 42.4 | 4.79 |
| 117 | C6TCA2 | uncharacterized protein | 2 | 1.49 | 25.7 | 9.83 |
| 118 | I1JJ78 | uncharacterized protein | 3 | 1.48 | 59.2 | 5.37 |
| 119 | B2BF98 | 40s ribosomal protein s6 | 5 | 1.48 | 28.0 | 10.71 |
| 120 | I1JKQ4 | arginase | 13 | 1.48 | 38.7 | 6.04 |
| 121 | I1M984 | uncharacterized protein | 24 | 1.47 | 60.2 | 5.81 |
| 122 | A0A0R0K666 | uncharacterized protein | 9 | 1.46 | 47.0 | 8.34 |
| 123 | C6TNE6 | chlorophyll a-b binding protein | 8 | 1.45 | 27.9 | 5.29 |
| 124 | I1KDK5 | uncharacterized protein | 5 | 1.45 | 67.4 | 6.12 |
| 125 | I1JCY0 | chlorophyll a-b binding protein | 2 | 1.44 | 26.7 | 6.21 |
| 126 | I1MFH3 | uncharacterized protein | 4 | 1.44 | 41.3 | 6.68 |
| 127 | C6SYE7 | uncharacterized protein | 4 | 1.44 | 17.6 | 6.02 |
| 128 | C6TBA0 | uncharacterized protein | 5 | 1.42 | 30.4 | 9.69 |
| 129 | I1LLA1 | uncharacterized protein | 7 | 1.42 | 79.4 | 8.20 |
| 130 | A0A0R0E8N5 | uricase | 13 | 1.39 | 35.1 | 8.31 |
| 131 | Q2PMS9 | ATP synthase subunit b | 3 | 1.39 | 21.1 | 8.55 |
| 132 | A0A0R0GQB4 | uncharacterized protein | 8 | 1.38 | 69.5 | 5.94 |
| 133 | I1JRP8 | uncharacterized protein | 2 | 1.37 | 38.1 | 6.68 |
| 134 | K7K9D0 | uncharacterized protein | 18 | 1.37 | 107.4 | 5.73 |
| 135 | A0A0R0GIJ9 | uncharacterized protein | 4 | 1.37 | 48.1 | 6.77 |
| 136 | I1K4A8 | uncharacterized protein | 6 | 1.34 | 38.9 | 5.22 |
| 137 | P02580 | actin-3 | 10 | 1.30 | 41.6 | 5.23 |
| 138 | K7KIL0 | GTP-binding nuclear protein | 8 | 1.30 | 25.0 | 6.42 |
| 139 | C6T7G2 | glycosyltransferase | 2 | 1.29 | 49.0 | 5.64 |
| 140 | A8HTK0 | thioredoxin | 3 | 1.29 | 13.3 | 5.37 |
| 141 | A0A0R4J559 | uncharacterized protein | 9 | 1.28 | 21.7 | 6.43 |
| 142 | I1L4U2 | serine hydroxymethyl transferase | 17 | 1.27 | 57.0 | 7.20 |
| 143 | A0A0R4J4L1 | uncharacterized protein | 16 | 1.23 | 34.9 | 5.65 |
| 144 | I1JGU8 | phospho-2-dehydro-3-deoxyheptonate aldolase | 12 | 1.22 | 59.0 | 8.57 |
| 145 | I1N8L7 | uncharacterized protein | 10 | 1.21 | 72.0 | 5.04 |
| 146 | A0A0R0F8K4 | uncharacterized protein | 7 | 1.21 | 54.5 | 8.70 |
| 147 | C6T8R7 | chlorophyll a-b binding protein | 6 | 1.21 | 28.6 | 5.46 |
| 148 | P49161 | cytochrome f | 4 | 1.21 | 35.3 | 8.38 |
| 149 | A0A0R4J318 | peptidyl-prolyl cis-trans isomerase | 6 | 1.20 | 18.9 | 7.69 |
| 150 | I1MIC1 | uncharacterized protein | 8 | 1.19 | 45.2 | 4.84 |
| 151 | C6SZA8 | uncharacterized protein | 6 | 1.19 | 22.5 | 6.42 |
| 152 | I1JUN3 | uncharacterized protein | 6 | 1.18 | 44.1 | 7.27 |
| 153 | I1K4C0 | uncharacterized protein | 6 | 1.18 | 57.9 | 6.47 |
| 154 | K7LNG2 | uncharacterized protein | 9 | 1.18 | 41.8 | 8.20 |
| 155 | A0A0R0I8Z5 | chlorophyll a-b binding protein | 5 | 1.18 | 31.0 | 5.72 |
| 156 | I1JTM0 | uncharacterized protein | 4 | 1.17 | 40.8 | 4.83 |
| 157 | I1M4G0 | carboxypeptidase | 16 | 1.16 | 51.0 | 4.82 |
| 158 | K7K557 | uncharacterized protein | 5 | 1.16 | 49.5 | 5.51 |
| 159 | I1KM30 | uncharacterized protein | 13 | 1.16 | 49.8 | 8.89 |
| 160 | I1MIW4 | uncharacterized protein | 6 | 1.16 | 39.8 | 5.55 |
| 161 | A0A0R4J389 | uncharacterized protein | 3 | 1.13 | 24.3 | 9.73 |
| 162 | K7MQY6 | uncharacterized protein | 21 | 1.13 | 75.4 | 4.83 |
| 163 | I1K795 | uncharacterized protein | 2 | 1.13 | 39.5 | 5.51 |
| 164 | Q2PMQ9 | photosystem ii cp47 reaction center protein | 6 | 1.12 | 56.0 | 6.20 |
| 165 | C6SYI8 | uncharacterized protein | 9 | 1.12 | 18.4 | 5.94 |
| 166 | C6SWW6 | uncharacterized protein | 2 | 1.11 | 11.6 | 9.37 |
| 167 | C6TIR2 | uncharacterized protein | 6 | 1.11 | 16.9 | 4.12 |
| 168 | I1KV09 | uncharacterized protein | 10 | 1.09 | 30.3 | 5.81 |
| 169 | I1KUQ6 | lipoxygenase | 18 | 1.09 | 97.1 | 6.93 |
| 170 | C6TL29 | phosphoribulokinase | 7 | 1.09 | 45.3 | 5.89 |
| 171 | I1MIC0 | uncharacterized protein | 9 | 1.08 | 21.5 | 6.29 |
| 172 | I1MTN1 | uncharacterized protein | 6 | 1.08 | 107.3 | 5.39 |
| 173 | A0A0R0IEK0 | uncharacterized protein | 8 | 1.06 | 50.6 | 6.91 |
| 174 | F8WRI3 | gamma-tocopherol methyltransferase | 4 | 1.06 | 33.3 | 6.33 |
| 175 | A0A0R0FC11 | uncharacterized protein | 2 | 1.06 | 52.0 | 9.32 |
| 176 | A0A0R0HPS3 | uncharacterized protein | 8 | 1.04 | 95.6 | 5.61 |
| 177 | I1M9K9 | uncharacterized protein | 5 | 1.04 | 39.8 | 6.53 |
| 178 | I1K6P4 | uncharacterized protein | 4 | 1.02 | 27.1 | 5.44 |
| 179 | C6T763 | uncharacterized protein | 12 | 1.02 | 30.8 | 5.04 |
| 180 | C6TKW2 | chlorophyll a-b binding protein | 5 | 1.02 | 28.8 | 5.11 |
| 181 | I1LDZ8 | uncharacterized protein | 4 | 1.00 | 37.7 | 5.89 |
| 182 | C6SYC1 | ATP synthase subunit d | 10 | 1.00 | 19.9 | 5.20 |
| 183 | Q2PMQ5 | cytochrome b6 | 2 | 1.00 | 24.1 | 8.89 |
| 184 | C6TL64 | peroxidase | 5 | 0.99 | 36.2 | 9.06 |
| 185 | C6TE99 | uncharacterized protein | 4 | 0.99 | 39.8 | 5.64 |
| 186 | I1MRK1 | uncharacterized protein | 18 | 0.97 | 78.7 | 9.14 |
| 187 | I1JDN2 | uncharacterized protein | 4 | 0.97 | 49.8 | 6.09 |
| 188 | I1N019 | uncharacterized protein | 5 | 0.97 | 72.2 | 5.84 |
| 189 | I1JNM9 | uncharacterized protein | 2 | 0.97 | 27.7 | 5.88 |
| 190 | C6SYU5 | uncharacterized protein | 5 | 0.97 | 20.9 | 11.06 |
| 191 | B3TDK9 | lipoxygenase | 43 | 0.96 | 96.3 | 6.34 |
| 192 | C6TGL4 | secretory carrier-associated membrane protein | 3 | 0.96 | 30.5 | 8.30 |
| 193 | I1M170 | glutamine synthetase | 7 | 0.96 | 47.6 | 6.42 |
| 194 | I1JHQ6 | uncharacterized protein | 10 | 0.95 | 60.9 | 8.78 |
| 195 | G0T440 | purple acid phosphatase | 15 | 0.95 | 70.3 | 5.93 |
| 196 | I1MTB1 | uncharacterized protein | 5 | 0.94 | 42.1 | 5.26 |
| 197 | I1KT48 | uncharacterized protein | 9 | 0.94 | 58.9 | 5.74 |
| 198 | I1MY33 | tubulin beta chain | 27 | 0.93 | 50.5 | 4.73 |
| 199 | C6TJP5 | uncharacterized protein | 6 | 0.92 | 25.7 | 5.01 |
| 200 | K7M2P2 | uncharacterized protein | 3 | 0.91 | 203.2 | 8.95 |
| 201 | I1M841 | uncharacterized protein | 10 | 0.90 | 52.4 | 5.73 |
| 202 | I1MDJ2 | uncharacterized protein | 5 | 0.90 | 17.3 | 10.21 |
| 203 | K7KZJ7 | uncharacterized protein | 18 | 0.90 | 86.2 | 6.16 |
| 204 | A0A0R4J4X3 | cysteine proteinase inhibitor | 3 | 0.89 | 10.7 | 5.83 |
| 205 | K7LJP8 | uncharacterized protein | 7 | 0.89 | 182.3 | 5.85 |
| 206 | A0A0R0LKB6 | uncharacterized protein | 8 | 0.89 | 33.9 | 4.56 |
| 207 | C6T0C7 | uncharacterized protein | 7 | 0.88 | 23.4 | 4.73 |
| 208 | I1LKM5 | uncharacterized protein | 9 | 0.88 | 39.3 | 9.43 |
| 209 | A0A0R0GU41 | uncharacterized protein | 4 | 0.88 | 21.7 | 9.17 |
| 210 | Q2PMP0 | protein tic 214 | 9 | 0.88 | 215.8 | 10.00 |
| 211 | I1KHC6 | lipoxygenase | 3 | 0.87 | 104.3 | 7.09 |
| 212 | I1LY05 | uncharacterized protein | 11 | 0.87 | 43.2 | 4.41 |
| 213 | I1MQD2 | uncharacterized protein | 2 | 0.87 | 12.2 | 5.70 |
| 214 | C6T9Q7 | ATP-dependent clp protease proteolytic subunit | 3 | 0.87 | 29.5 | 8.57 |
| 215 | A0A0R0FAD6 | uncharacterized protein | 6 | 0.86 | 51.2 | 9.15 |
| 216 | I1LCI8 | UDP-glycosyltransferase 79a6 | 3 | 0.86 | 52.2 | 5.99 |
| 217 | C6TIW0 | fructose-bisphosphate aldolase | 10 | 0.85 | 42.5 | 8.73 |
| 218 | A0A0R0IYE6 | lipoxygenase | 29 | 0.85 | 96.3 | 6.22 |
| 219 | I1J8R4 | peroxidase | 9 | 0.84 | 36.4 | 9.26 |
| 220 | I1LBH6 | uncharacterized protein | 18 | 0.84 | 50.5 | 4.87 |
| 221 | A8IFI1 | s1 ribosomal protein | 2 | 0.84 | 23.4 | 10.27 |
| 222 | C6SWP9 | uncharacterized protein | 4 | 0.83 | 24.8 | 9.85 |
| 223 | C6K8D0 | trypsin inhibitor 26 kda isoform | 8 | 0.83 | 25.9 | 7.83 |
| 224 | I1KMC5 | uncharacterized protein | 5 | 0.82 | 31.5 | 5.64 |
| 225 | C6TI42 | uncharacterized protein | 3 | 0.82 | 6.3 | 4.28 |
| 226 | C6THU1 | uncharacterized protein | 10 | 0.82 | 39.2 | 5.65 |
| 227 | C6SXD0 | uncharacterized protein | 9 | 0.82 | 17.8 | 5.96 |
| 228 | C6T0E8 | 60s ribosomal protein l18a | 7 | 0.82 | 21.3 | 10.44 |
| 229 | I1LZ92 | uncharacterized protein | 14 | 0.82 | 37.5 | 5.29 |
| 230 | C6T588 | uncharacterized protein | 8 | 0.82 | 16.8 | 4.73 |
| 231 | I1L1Q3 | uncharacterized protein | 3 | 0.81 | 45.1 | 6.56 |
| 232 | K7MGU2 | uncharacterized protein | 2 | 0.81 | 25.7 | 5.60 |
| 233 | I1K4M2 | glycosyltransferase | 11 | 0.81 | 55.8 | 5.74 |
| 234 | A0A0R4J532 | uncharacterized protein | 6 | 0.81 | 21.7 | 6.43 |
| 235 | A0A0R4J3L3 | uncharacterized protein | 6 | 0.80 | 43.0 | 9.12 |
| 236 | I1MNK0 | uncharacterized protein | 9 | 0.80 | 35.0 | 6.66 |
| 237 | I1JBA6 | uncharacterized protein | 2 | 0.80 | 51.0 | 5.51 |
| 238 | C6TI82 | methyltransferase | 8 | 0.80 | 38.5 | 6.22 |
| 239 | I1N272 | acyl-[acyl-carrier-protein] hydrolase | 4 | 0.80 | 41.9 | 8.11 |
| 240 | C6SWX2 | uncharacterized protein | 3 | 0.80 | 15.1 | 5.92 |
| 241 | Q2PMV0 | ATP synthase subunit beta | 22 | 0.79 | 53.7 | 5.29 |
| 242 | A0A0R0J8D5 | uncharacterized protein | 5 | 0.79 | 24.9 | 9.80 |
| 243 | C6TML0 | uncharacterized protein | 9 | 0.78 | 33.1 | 6.17 |
| 244 | K7L949 | uncharacterized protein | 2 | 0.77 | 14.2 | 6.11 |
| 245 | I1KXW8 | reticulon-like protein | 4 | 0.77 | 27.0 | 8.53 |
| 246 | C6TFC1 | non-specific lipid-transfer protein | 5 | 0.76 | 12.4 | 9.03 |
| 247 | I1LFG4 | uncharacterized protein | 14 | 0.76 | 80.2 | 6.12 |
| 248 | I1LYA4 | eukaryotic translation initiation factor 3 subunit k | 8 | 0.75 | 26.2 | 5.47 |
| 249 | C6T5E5 | uncharacterized protein | 3 | 0.75 | 24.1 | 6.24 |
| 250 | A0A0R0FKU0 | uncharacterized protein | 2 | 0.74 | 29.7 | 4.18 |
| 251 | I1K5U0 | uncharacterized protein | 14 | 0.74 | 58.5 | 6.00 |
| 252 | C6SXK8 | uncharacterized protein | 2 | 0.74 | 12.9 | 9.41 |
| 253 | C6KHU4 | phenylalanine ammonia-lyase | 16 | 0.74 | 78.1 | 5.83 |
| 254 | I1M2Y5 | uncharacterized protein | 14 | 0.74 | 38.5 | 6.25 |
| 255 | K7L6U4 | uncharacterized protein | 13 | 0.74 | 42.0 | 9.15 |
| 256 | I1JX24 | uncharacterized protein | 8 | 0.73 | 45.8 | 6.50 |
| 257 | K7K6H1 | uncharacterized protein | 2 | 0.73 | 24.7 | 7.90 |
| 258 | I1J5Q3 | uncharacterized protein | 13 | 0.73 | 68.9 | 6.63 |
| 259 | I1MUQ0 | uncharacterized protein | 2 | 0.73 | 27.4 | 5.98 |
| 260 | A0A0R4J3P1 | glutamine synthetase | 12 | 0.73 | 39.1 | 5.32 |
| 261 | I1MYZ6 | uncharacterized protein | 5 | 0.72 | 79.1 | 5.12 |
| 262 | C6SWY6 | uncharacterized protein | 8 | 0.72 | 16.5 | 4.68 |
| 263 | I1JEV7 | uncharacterized protein | 4 | 0.72 | 29.8 | 4.98 |
| 264 | K7LM18 | serine/threonine-protein phosphatase | 11 | 0.71 | 34.8 | 4.87 |
| 265 | I1LY51 | uncharacterized protein | 15 | 0.70 | 44.1 | 8.22 |
| 266 | I1LRA7 | carboxypeptidase | 8 | 0.70 | 51.0 | 5.11 |
| 267 | I1LU49 | uncharacterized protein | 6 | 0.70 | 38.2 | 5.71 |
| 268 | I1LAH8 | uncharacterized protein | 3 | 0.70 | 23.9 | 5.69 |
| 269 | K7LWI4 | ATP synthase subunit alpha | 8 | 0.69 | 55.7 | 5.15 |
| 270 | I1KM16 | uncharacterized protein | 11 | 0.69 | 28.1 | 5.46 |
| 271 | I1KWP5 | uncharacterized protein | 2 | 0.69 | 45.0 | 6.94 |
| 272 | I1M311 | mannosyltransferase | 2 | 0.69 | 64.1 | 8.43 |
| 273 | I1MFX5 | endoglucanase | 6 | 0.68 | 68.3 | 8.81 |
| 274 | I1MC85 | uncharacterized protein | 7 | 0.68 | 45.8 | 5.55 |
| 275 | I1MBF5 | uncharacterized protein | 4 | 0.67 | 51.2 | 5.75 |
| 276 | C6TK63 | uncharacterized protein | 2 | 0.67 | 35.1 | 6.36 |
| 277 | Q5D1M7 | chloroplast glutamine synthetase | 4 | 0.67 | 30.9 | 5.56 |
| 278 | O04874 | glutathione transferase | 7 | 0.67 | 25.0 | 5.76 |
| 279 | A0A0R0HBF1 | uncharacterized protein | 5 | 0.67 | 25.3 | 5.08 |
| 280 | I1KH84 | uncharacterized protein | 2 | 0.66 | 28.8 | 5.31 |
| 281 | K7KKK3 | uncharacterized protein | 3 | 0.66 | 61.0 | 6.87 |
| 282 | I1JW44 | uncharacterized protein | 10 | 0.66 | 46.9 | 8.91 |
| 283 | A7LCD5 | lipoxygenase | 25 | 0.66 | 96.3 | 6.54 |
| 284 | C6T0H9 | uncharacterized protein | 6 | 0.66 | 15.0 | 10.40 |
| 285 | I1K7Q8 | uncharacterized protein | 11 | 0.66 | 34.2 | 5.73 |
| 286 | A0A0R0EY92 | uncharacterized protein | 9 | 0.66 | 35.8 | 7.04 |
| 287 | C6TLV6 | 40s ribosomal protein s3a | 11 | 0.65 | 29.6 | 9.81 |
| 288 | I1MH86 | uncharacterized protein | 10 | 0.65 | 41.4 | 4.36 |
| 289 | I1ML66 | glyceraldehyde-3-phosphate dehydrogenase | 10 | 0.65 | 43.2 | 7.61 |
| 290 | O23958 | metallothionein-ii protein | 5 | 0.65 | 8.1 | 6.64 |
| 291 | I1KY49 | uncharacterized protein | 8 | 0.65 | 51.2 | 6.73 |
| 292 | I1MNL7 | carboxypeptidase | 6 | 0.65 | 55.5 | 5.12 |
| 293 | A0A0R4J5K2 | ferredoxin--nadp reductase | 10 | 0.65 | 40.5 | 8.04 |
| 294 | A0A0R0KQE9 | uncharacterized protein | 19 | 0.65 | 50.7 | 6.80 |
| 295 | A0A0R0EME4 | uncharacterized protein | 6 | 0.64 | 39.9 | 5.44 |
| 296 | A0A0R0FJI5 | uncharacterized protein | 21 | 0.64 | 152.8 | 6.07 |
| 297 | I1JV39 | glucose-6-phosphate isomerase | 23 | 0.64 | 67.3 | 5.66 |
| 298 | B1ACD3 | kunitz trypsin protease inhibitor | 7 | 0.63 | 23.6 | 4.68 |
| 299 | C6SVL2 | ribulose bisphosphate carboxylase small chain | 6 | 0.62 | 20.0 | 8.87 |
| 300 | I1KFE9 | uncharacterized protein | 10 | 0.62 | 60.1 | 6.93 |
| 301 | C6TBL0 | Mg-protoporphyrin ix chelatase | 8 | 0.62 | 45.8 | 5.60 |
| 302 | I1KLC9 | uncharacterized protein | 18 | 0.61 | 65.2 | 7.55 |
| 303 | A0A0R4J4S6 | uncharacterized protein | 13 | 0.60 | 43.2 | 8.95 |
| 304 | I1MU50 | uncharacterized protein | 4 | 0.59 | 60.8 | 5.88 |
| 305 | C6SWE3 | uncharacterized protein | 5 | 0.59 | 23.9 | 5.69 |
| 306 | A0A0R0FLF0 | xyloglucan endotransglucosylase/hydrolase | 13 | 0.59 | 40.4 | 9.31 |
| 307 | C6TKL8 | uncharacterized protein | 9 | 0.58 | 34.8 | 6.49 |
| 308 | I1K9N4 | glucose-6-phosphate isomerase | 20 | 0.57 | 67.4 | 5.52 |
| 309 | K7LQI0 | dirigent protein | 8 | 0.57 | 38.5 | 9.28 |
| 310 | I1M599 | lipoxygenase | 44 | 0.57 | 92.7 | 6.52 |
| 311 | A0A0R4J3I5 | uncharacterized protein | 12 | 0.57 | 39.9 | 5.58 |
| 312 | I1KXM1 | pyruvate dehydrogenase e1 component subunit alpha | 9 | 0.57 | 43.6 | 7.12 |
| 313 | C6T0A7 | uncharacterized protein | 6 | 0.57 | 17.5 | 10.56 |
| 314 | K7MGN4 | uncharacterized protein | 9 | 0.57 | 132.7 | 6.08 |
| 315 | I1MXB1 | glycine cleavage system p protein | 21 | 0.56 | 114.5 | 6.92 |
| 316 | I1JAN9 | uncharacterized protein | 3 | 0.56 | 17.3 | 9.56 |
| 317 | B0M195 | peroxisomal short-chain dehydrogenase/reductase family protein | 7 | 0.56 | 26.3 | 8.80 |
| 318 | C6TA29 | uncharacterized protein | 6 | 0.56 | 30.7 | 9.45 |
| 319 | B3TDK7 | lipoxygenase | 48 | 0.56 | 96.4 | 5.76 |
| 320 | I1L4K9 | elongation factor ts | 10 | 0.55 | 122.7 | 4.66 |
| 321 | C6TGT0 | coatomer subunit epsilon | 11 | 0.55 | 32.3 | 5.30 |
| 322 | A0A0R0H3D0 | uncharacterized protein | 7 | 0.55 | 28.4 | 7.17 |
| 323 | A0A0R0GWM0 | uncharacterized protein | 8 | 0.55 | 26.6 | 7.99 |
| 324 | I1JMI9 | malic enzyme | 15 | 0.55 | 68.9 | 5.71 |
| 325 | C6TIC4 | reticulon-like protein | 3 | 0.55 | 28.5 | 7.78 |
| 326 | I1MK13 | uncharacterized protein | 13 | 0.54 | 61.1 | 6.98 |
| 327 | I1N6A6 | udp-glucose 6-dehydrogenase | 17 | 0.54 | 53.1 | 6.29 |
| 328 | I1KEW2 | uncharacterized protein | 5 | 0.54 | 40.6 | 5.66 |
| 329 | C6T8Y4 | ferredoxin--nadp reductase | 8 | 0.53 | 42.2 | 8.38 |
| 330 | I1NG28 | uncharacterized protein | 17 | 0.53 | 69.8 | 5.25 |
| 331 | I1MNX6 | uncharacterized protein | 9 | 0.53 | 47.4 | 6.57 |
| 332 | I1K8L5 | uncharacterized protein | 3 | 0.53 | 12.2 | 7.78 |
| 333 | I1L053 | uncharacterized protein | 11 | 0.52 | 39.2 | 5.49 |
| 334 | I1LVA7 | uncharacterized protein | 25 | 0.52 | 75.6 | 6.65 |
| 335 | I1JTU1 | branched-chain-amino-acid aminotransferase | 8 | 0.52 | 42.0 | 6.76 |
| 336 | C6TN20 | eukaryotic translation initiation factor 3 subunit h | 9 | 0.52 | 38.8 | 4.73 |
| 337 | A0A0R0JPL6 | uncharacterized protein | 9 | 0.52 | 50.0 | 8.57 |
| 338 | C6TD22 | uncharacterized protein | 3 | 0.52 | 36.4 | 6.05 |
| 339 | A8C8H3 | glutamate decarboxylase | 23 | 0.52 | 57.1 | 5.52 |
| 340 | C6SVF1 | actin depolymerizing factor 1 | 5 | 0.52 | 16.0 | 6.15 |
| 341 | C6TG68 | uncharacterized protein | 6 | 0.51 | 16.6 | 4.20 |
| 342 | A0A0R0GYY6 | uncharacterized protein | 5 | 0.51 | 30.5 | 5.14 |
| 343 | A0A0R0HR30 | uncharacterized protein | 17 | 0.51 | 47.0 | 5.49 |
| 344 | K7KGI6 | uncharacterized protein | 11 | 0.51 | 55.3 | 6.28 |
| 345 | I1KHB7 | uncharacterized protein | 3 | 0.51 | 23.8 | 6.24 |
| 346 | I1J7Y4 | uncharacterized protein | 15 | 0.51 | 201.7 | 5.49 |
| 347 | C6SXD3 | 40s ribosomal protein s24 | 3 | 0.51 | 15.8 | 10.64 |
| 348 | I1MXL3 | uncharacterized protein | 10 | 0.50 | 52.1 | 5.55 |
| 349 | Q9FUK4 | glutamine synthetase | 16 | 0.50 | 39.0 | 5.48 |
| 350 | I1ME83 | ubiquitin thioesterase | 7 | 0.50 | 34.0 | 4.68 |
| 351 | I1KAS2 | uncharacterized protein | 5 | 0.50 | 86.8 | 6.43 |
| 352 | I1J4B5 | uncharacterized protein | 6 | 0.49 | 51.0 | 6.20 |
| 353 | I1MM08 | malic enzyme | 23 | 0.49 | 65.2 | 5.75 |
| 354 | A0A0R0IYY7 | uncharacterized protein | 22 | 0.49 | 59.0 | 8.78 |
| 355 | I1JAQ7 | uncharacterized protein | 20 | 0.49 | 45.2 | 6.36 |
| 356 | I1MTH5 | uncharacterized protein | 13 | 0.49 | 51.4 | 5.73 |
| 357 | C6TGA6 | 60s acidic ribosomal protein p0 | 11 | 0.49 | 34.2 | 5.00 |
| 358 | C6SXW1 | uncharacterized protein | 3 | 0.49 | 15.1 | 6.29 |
| 359 | I1MJ28 | uncharacterized protein | 22 | 0.48 | 62.8 | 5.72 |
| 360 | I1K711 | uncharacterized protein | 6 | 0.48 | 40.0 | 4.82 |
| 361 | I1MUX7 | uncharacterized protein | 10 | 0.48 | 66.7 | 9.22 |
| 362 | I1MVN5 | peroxidase | 15 | 0.47 | 35.5 | 9.13 |
| 363 | I1JFN3 | uncharacterized protein | 3 | 0.47 | 13.6 | 4.66 |
| 364 | I1KB59 | uncharacterized protein | 2 | 0.47 | 30.8 | 5.36 |
| 365 | I1NHC6 | uncharacterized protein | 15 | 0.46 | 63.2 | 6.22 |
| 366 | I1L6T4 | uncharacterized protein | 8 | 0.46 | 42.5 | 5.48 |
| 367 | I1NBK8 | uncharacterized protein | 9 | 0.46 | 39.4 | 5.75 |
| 368 | A0A0R0JZ42 | uncharacterized protein | 10 | 0.46 | 30.9 | 9.00 |
| 369 | I1NHR8 | uncharacterized protein | 9 | 0.45 | 55.7 | 5.47 |
| 370 | A0A0R4J3E9 | uncharacterized protein | 5 | 0.45 | 17.7 | 7.82 |
| 371 | I1LL59 | protein disulfide-isomerase | 19 | 0.45 | 64.8 | 4.58 |
| 372 | I1KJG0 | uncharacterized protein | 7 | 0.45 | 49.0 | 5.14 |
| 373 | Q2PMS5 | 30s ribosomal protein s16 | 2 | 0.44 | 10.4 | 10.60 |
| 374 | I1NGB9 | uncharacterized protein | 5 | 0.44 | 38.6 | 5.41 |
| 375 | I1KWM7 | 6-phosphogluconate dehydrogenase | 23 | 0.43 | 53.5 | 6.11 |
| 376 | I1M6M4 | uncharacterized protein | 5 | 0.43 | 61.2 | 5.12 |
| 377 | K7M3V5 | coatomer subunit beta' | 28 | 0.43 | 104.1 | 4.97 |
| 378 | K7M387 | uncharacterized protein | 25 | 0.43 | 127.4 | 5.18 |
| 379 | A0A0R0HDA3 | protein disulfide-isomerase | 15 | 0.42 | 67.3 | 4.82 |
| 380 | I1M1P1 | uncharacterized protein | 10 | 0.42 | 33.2 | 5.93 |
| 381 | K7KYB1 | aconitate hydratase | 22 | 0.42 | 106.9 | 6.58 |
| 382 | I1MKT8 | glycosyltransferase | 8 | 0.42 | 54.4 | 6.36 |
| 383 | I1LGS5 | acyl-coenzyme a oxidase | 17 | 0.42 | 74.5 | 7.01 |
| 384 | I1JR35 | uncharacterized protein | 12 | 0.42 | 39.5 | 7.65 |
| 385 | I1MGE7 | uncharacterized protein | 30 | 0.41 | 65.9 | 7.95 |
| 386 | I1JJP2 | glucose-1-phosphate adenylyltransferase | 15 | 0.40 | 56.3 | 6.29 |
| 387 | C6SXF0 | uncharacterized protein | 3 | 0.40 | 22.6 | 10.22 |
| 388 | A0A0R0JYY8 | tubulin beta chain | 21 | 0.40 | 49.9 | 4.89 |
| 389 | I1LR23 | uncharacterized protein | 16 | 0.39 | 109.5 | 7.06 |
| 390 | K7KG80 | uncharacterized protein | 3 | 0.39 | 35.1 | 7.10 |
| 391 | A0A0R0G797 | uncharacterized protein | 11 | 0.39 | 43.2 | 6.48 |
| 392 | A0A0R0JQU1 | uncharacterized protein | 5 | 0.38 | 32.9 | 4.80 |
| 393 | I1JA26 | hydroxymethylglutaryl-coa synthase | 13 | 0.38 | 52.2 | 6.20 |
| 394 | I1K5E6 | uncharacterized protein | 17 | 0.38 | 35.6 | 7.62 |
| 395 | C6TAN4 | uncharacterized protein | 13 | 0.37 | 50.9 | 8.91 |
| 396 | C6SZ14 | uncharacterized protein | 7 | 0.37 | 26.6 | 5.33 |
| 397 | I1JQY5 | pectin acetylesterase | 6 | 0.36 | 45.0 | 5.76 |
| 398 | I1MHG6 | uncharacterized protein | 24 | 0.35 | 54.1 | 5.54 |
| 399 | I1LLG3 | uncharacterized protein | 9 | 0.35 | 46.8 | 9.20 |
| 400 | I1MUR2 | uncharacterized protein | 15 | 0.35 | 36.7 | 5.95 |
| 401 | K7M7P5 | phospho-2-dehydro-3-deoxyheptonate aldolase | 10 | 0.35 | 56.5 | 7.70 |
| 402 | I1LMX6 | uroporphyrinogen decarboxylase | 4 | 0.35 | 42.7 | 7.06 |
| 403 | C6T7Z6 | uncharacterized protein | 5 | 0.35 | 12.3 | 9.61 |
| 404 | C6T504 | uncharacterized protein | 2 | 0.34 | 10.3 | 8.06 |
| 405 | I1MZ47 | uncharacterized protein | 14 | 0.34 | 34.7 | 5.85 |
| 406 | C6TBP8 | uncharacterized protein | 11 | 0.33 | 26.6 | 7.65 |
| 407 | I1LVZ8 | uncharacterized protein | 27 | 0.33 | 85.5 | 8.00 |
| 408 | C6T1N4 | uncharacterized protein | 3 | 0.33 | 17.5 | 4.85 |
| 409 | I1KS65 | adenosylhomocysteinase | 26 | 0.33 | 53.3 | 5.60 |
| 410 | P24095 | seed linoleate 9s-lipoxygenase | 48 | 0.33 | 96.8 | 5.78 |
| 411 | B0M1A5 | betaine aldehyde dehydrogenase | 28 | 0.32 | 54.6 | 5.35 |
| 412 | C6T8K3 | uncharacterized protein | 14 | 0.32 | 27.4 | 5.13 |
| 413 | I1KBB6 | 4-alpha-glucanotransferase | 9 | 0.32 | 63.9 | 5.63 |
| 414 | D4Q9Z4 | soyasapogenol b glucuronide galactosyltransferase | 18 | 0.31 | 55.8 | 5.77 |
| 415 | I1JH86 | fructose-bisphosphate aldolase | 22 | 0.31 | 38.2 | 7.12 |
| 416 | I1JGB2 | s-formylglutathione hydrolase | 11 | 0.31 | 32.1 | 6.55 |
| 417 | C6T514 | 40s ribosomal protein s8 | 9 | 0.31 | 27.6 | 10.19 |
| 418 | K7MVK8 | uncharacterized protein | 11 | 0.30 | 31.5 | 5.90 |
| 419 | C6TN55 | uncharacterized protein | 13 | 0.30 | 43.5 | 6.56 |
| 420 | I1KPJ5 | nucleoside diphosphate kinase | 5 | 0.30 | 25.5 | 9.30 |
| 421 | I1LAL4 | uncharacterized protein | 34 | 0.30 | 61.1 | 5.99 |
| 422 | I1K9J7 | tubulin alpha chain | 21 | 0.29 | 49.7 | 4.87 |
| 423 | C6TJX5 | uncharacterized protein | 9 | 0.29 | 16.6 | 6.96 |
| 424 | I1MMH5 | pectin acetylesterase | 14 | 0.28 | 45.8 | 8.69 |
| 425 | C6TF34 | uncharacterized protein | 6 | 0.28 | 32.4 | 6.04 |
| 426 | I1K8M2 | transmembrane 9 superfamily member | 9 | 0.28 | 73.2 | 7.15 |
| 427 | I1NFK8 | uncharacterized protein | 25 | 0.28 | 84.8 | 6.09 |
| 428 | I1M829 | uncharacterized protein | 6 | 0.28 | 41.0 | 6.28 |
| 429 | K7KNJ0 | uncharacterized protein | 2 | 0.28 | 16.5 | 10.42 |
| 430 | A0A0R4J574 | serine hydroxymethyltransferase | 19 | 0.27 | 56.1 | 8.35 |
| 431 | I1JEH5 | uncharacterized protein | 3 | 0.27 | 19.5 | 4.56 |
| 432 | C6SXS9 | uncharacterized protein | 16 | 0.26 | 23.5 | 5.81 |
| 433 | B0M1A6 | betaine aldehyde dehydrogenase | 29 | 0.26 | 54.7 | 5.23 |
| 434 | K7KJ72 | uncharacterized protein | 19 | 0.24 | 404.8 | 5.26 |
| 435 | O64458 | 34 kda maturing seed protein | 3 | 0.24 | 42.7 | 5.63 |
| 436 | Q71EW8 | methionine synthase | 43 | 0.24 | 84.2 | 5.93 |
| 437 | I1KMX5 | uncharacterized protein | 21 | 0.24 | 86.0 | 9.13 |
| 438 | I1JRS9 | uncharacterized protein | 6 | 0.23 | 62.0 | 4.97 |
| 439 | I1N691 | uncharacterized protein | 18 | 0.23 | 117.0 | 5.54 |
| 440 | I1KDI8 | uncharacterized protein | 8 | 0.21 | 85.2 | 6.52 |
| 441 | C6TAX0 | uncharacterized protein | 10 | 0.21 | 39.8 | 5.56 |
| 442 | I1KZW7 | pyrophosphate--fructose 6-phosphate 1-phosphotransferase subunit beta | 20 | 0.21 | 61.4 | 6.58 |
| 443 | I1LZ03 | uncharacterized protein | 17 | 0.20 | 44.6 | 10.48 |
| 444 | C6SVC4 | uncharacterized protein | 8 | 0.20 | 27.6 | 5.67 |
| 445 | Q76LA6 | cytosolic ascorbate peroxidase 2 | 12 | 0.19 | 27.1 | 5.65 |
| 446 | I1N4I6 | udp-glucose 6-dehydrogenase | 21 | 0.19 | 52.9 | 5.74 |
| 447 | C6THR8 | uncharacterized protein | 11 | 0.19 | 35.6 | 8.91 |
| 448 | K7MJY8 | sucrose synthase | 43 | 0.18 | 92.1 | 5.93 |
| 449 | C6SVD0 | uncharacterized protein | 2 | 0.18 | 12.0 | 5.29 |
| 450 | I1J5Y9 | udp-glucose 6-dehydrogenase | 17 | 0.17 | 52.9 | 6.34 |
| 451 | I1LGI7 | hydroxymethylglutaryl-coa synthase | 14 | 0.16 | 52.4 | 5.77 |
| 452 | I1LNE6 | uncharacterized protein | 23 | 0.08 | 59.2 | 5.60 |
| 453 | I1K565 | uncharacterized protein | 33 | -0.12 | 108.5 | 6.41 |
| 454 | I1L4U3 | uncharacterized protein | 29 | -0.15 | 88.4 | 5.93 |
| 455 | I1N0A1 | uncharacterized protein | 7 | -0.16 | 32.8 | 6.54 |
| 456 | I1LDX0 | uncharacterized protein | 12 | -0.16 | 68.4 | 6.01 |
| 457 | I1K7N6 | thioredoxin | 6 | -0.17 | 13.0 | 5.65 |
| 458 | I1KQD8 | uncharacterized protein | 9 | -0.17 | 41.4 | 6.35 |
| 459 | I7FST9 | protein disulfide-isomerase | 26 | -0.18 | 58.7 | 4.92 |
| 460 | K7LI90 | uncharacterized protein | 18 | -0.19 | 92.4 | 6.69 |
| 461 | A0A0R0J4X0 | uncharacterized protein | 13 | -0.20 | 29.0 | 4.70 |
| 462 | I1J8Y0 | uncharacterized protein | 21 | -0.20 | 73.0 | 5.46 |
| 463 | Q9FQD4 | glutathione s-transferase gst 24 | 9 | -0.20 | 24.8 | 5.74 |
| 464 | I1K5M9 | uncharacterized protein | 29 | -0.20 | 63.5 | 5.33 |
| 465 | I1M6E1 | uncharacterized protein | 40 | -0.20 | 80.1 | 4.98 |
| 466 | C6TJP6 | uncharacterized protein | 2 | -0.21 | 31.0 | 9.35 |
| 467 | A0A0R0LAF9 | proteasome subunit alpha type | 15 | -0.22 | 27.3 | 5.96 |
| 468 | I1JY67 | uncharacterized protein | 8 | -0.22 | 50.8 | 7.05 |
| 469 | I1LHP2 | uncharacterized protein | 10 | -0.22 | 43.2 | 5.51 |
| 470 | I1LM73 | uncharacterized protein | 28 | -0.22 | 51.9 | 5.32 |
| 471 | I1LU40 | uncharacterized protein | 6 | -0.22 | 26.3 | 5.38 |
| 472 | I1N7Z9 | uncharacterized protein | 7 | -0.22 | 97.6 | 5.26 |
| 473 | I1J637 | uncharacterized protein | 20 | -0.23 | 41.7 | 5.52 |
| 474 | I1KBK8 | uncharacterized protein | 16 | -0.23 | 91.0 | 5.14 |
| 475 | C6TBS0 | dolichyl-diphosphooligosaccharide--protein glycosyltransferase 48 kda subunit | 13 | -0.23 | 48.1 | 5.92 |
| 476 | I1KUU5 | protein root hair defective 3 homolog | 15 | -0.23 | 89.5 | 5.33 |
| 477 | I1JPP3 | uncharacterized protein | 35 | -0.24 | 89.6 | 5.13 |
| 478 | I1LCG9 | uncharacterized protein | 3 | -0.24 | 65.5 | 6.48 |
| 479 | I1MUT2 | uncharacterized protein | 13 | -0.24 | 43.5 | 4.93 |
| 480 | I1LIT8 | alanine--trna ligase | 30 | -0.24 | 109.6 | 5.66 |
| 481 | I1LHN4 | uncharacterized protein | 23 | -0.24 | 73.2 | 5.53 |
| 482 | I1JBS3 | uncharacterized protein | 10 | -0.24 | 48.6 | 5.15 |
| 483 | Q9FQ95 | in2-1 protein | 13 | -0.24 | 27.0 | 5.21 |
| 484 | C6TJG0 | uncharacterized protein | 11 | -0.24 | 26.6 | 6.77 |
| 485 | I1MJE1 | uncharacterized protein | 18 | -0.24 | 52.8 | 6.00 |
| 486 | C6TER4 | uncharacterized protein | 10 | -0.24 | 34.9 | 6.33 |
| 487 | I1JJL6 | uncharacterized protein | 38 | -0.25 | 80.3 | 4.96 |
| 488 | I1KGN9 | uncharacterized protein | 9 | -0.25 | 34.2 | 5.77 |
| 489 | C6SYZ8 | uncharacterized protein | 5 | -0.26 | 23.6 | 10.33 |
| 490 | I1KUU6 | transmembrane 9 superfamily member | 11 | -0.26 | 73.6 | 7.94 |
| 491 | I1KJR2 | uncharacterized protein | 13 | -0.26 | 67.8 | 5.47 |
| 492 | I1MY29 | phosphotransferase | 8 | -0.26 | 53.4 | 5.48 |
| 493 | I1KGP8 | uncharacterized protein | 7 | -0.27 | 42.1 | 6.36 |
| 494 | I1N814 | alpha-mannosidase | 10 | -0.27 | 115.7 | 5.88 |
| 495 | K7KEW2 | uncharacterized protein | 19 | -0.27 | 111.5 | 5.88 |
| 496 | C6T530 | 40s ribosomal protein s7 | 6 | -0.27 | 22.0 | 9.80 |
| 497 | I1LIG5 | uncharacterized protein | 12 | -0.27 | 68.3 | 7.81 |
| 498 | C6SWX1 | uncharacterized protein | 3 | -0.27 | 12.0 | 10.59 |
| 499 | C6T8W5 | elongation factor tu | 14 | -0.27 | 49.9 | 6.62 |
| 500 | I1M4Y9 | uncharacterized protein | 13 | -0.27 | 87.5 | 7.29 |
| 501 | I1KQX0 | uncharacterized protein | 15 | -0.27 | 58.2 | 7.61 |
| 502 | I1JWZ3 | plasma membrane atpase | 20 | -0.27 | 105.2 | 6.28 |
| 503 | I1KEY6 | uncharacterized protein | 46 | -0.28 | 97.3 | 5.04 |
| 504 | I1M2V7 | uncharacterized protein | 25 | -0.28 | 75.5 | 6.65 |
| 505 | I1LFL2 | uncharacterized protein | 10 | -0.28 | 117.8 | 4.76 |
| 506 | I1M322 | uncharacterized protein | 17 | -0.28 | 46.8 | 6.48 |
| 507 | I1KJ35 | uncharacterized protein | 11 | -0.29 | 58.7 | 9.05 |
| 508 | C6ZRR6 | serine/threonine protein kinase | 8 | -0.29 | 39.2 | 6.13 |
| 509 | I1LI99 | uncharacterized protein | 10 | -0.29 | 57.3 | 4.74 |
| 510 | C6SY19 | uncharacterized protein | 5 | -0.29 | 20.1 | 9.69 |
| 511 | A0A0R0JSY5 | uncharacterized protein | 8 | -0.29 | 126.1 | 8.64 |
| 512 | I1L7W8 | phi class glutathione s-transferase | 3 | -0.29 | 24.7 | 5.26 |
| 513 | C6TL46 | uncharacterized protein | 5 | -0.29 | 29.6 | 5.77 |
| 514 | C6SZW0 | uncharacterized protein | 9 | -0.29 | 22.1 | 4.43 |
| 515 | K7LAH5 | proliferating cell nuclear antigen | 9 | -0.30 | 36.7 | 4.78 |
| 516 | I1MQG3 | uncharacterized protein | 5 | -0.30 | 22.2 | 4.74 |
| 517 | C6TKA3 | nadh-cytochrome b5 reductase | 10 | -0.30 | 35.1 | 8.63 |
| 518 | I1JY44 | uncharacterized protein | 4 | -0.30 | 58.7 | 5.67 |
| 519 | I1KGB8 | uncharacterized protein | 16 | -0.31 | 61.1 | 7.13 |
| 520 | I1N628 | uncharacterized protein | 8 | -0.31 | 29.7 | 9.06 |
| 521 | I1M1V8 | uncharacterized protein | 17 | -0.31 | 63.4 | 5.73 |
| 522 | I1LME6 | uncharacterized protein | 8 | -0.31 | 53.6 | 5.42 |
| 523 | C6SWH1 | uncharacterized protein | 4 | -0.31 | 17.7 | 5.44 |
| 524 | I1KBT9 | uncharacterized protein | 7 | -0.32 | 68.7 | 5.43 |
| 525 | I1KQ93 | uncharacterized protein | 29 | -0.32 | 63.4 | 5.35 |
| 526 | C6T160 | uncharacterized protein | 3 | -0.32 | 10.3 | 9.99 |
| 527 | I1JXE8 | uncharacterized protein | 18 | -0.32 | 77.9 | 5.09 |
| 528 | I1JGB5 | eukaryotic translation initiation factor 3 subunit e | 20 | -0.32 | 51.1 | 5.53 |
| 529 | I1JXH8 | uncharacterized protein | 16 | -0.32 | 53.2 | 6.21 |
| 530 | Q9LLQ6 | seed maturation protein pm34 | 27 | -0.32 | 31.7 | 6.60 |
| 531 | I1MGV6 | 60s ribosomal protein l27 | 5 | -0.32 | 15.6 | 10.38 |
| 532 | K7LKA7 | uncharacterized protein | 6 | -0.33 | 47.5 | 6.97 |
| 533 | A0A0R4J3H1 | uncharacterized protein | 8 | -0.33 | 30.4 | 4.18 |
| 534 | I1MI23 | uncharacterized protein | 11 | -0.33 | 81.6 | 5.74 |
| 535 | K7M3S8 | uncharacterized protein | 3 | -0.33 | 60.4 | 5.49 |
| 536 | I1K4S0 | uncharacterized protein | 12 | -0.33 | 27.5 | 6.09 |
| 537 | I1LXY1 | uncharacterized protein | 27 | -0.33 | 89.0 | 6.33 |
| 538 | I1MGH2 | uncharacterized protein | 6 | -0.33 | 84.0 | 8.58 |
| 539 | I1KQZ7 | uncharacterized protein | 18 | -0.34 | 137.7 | 5.81 |
| 540 | I1LPN7 | uncharacterized protein | 6 | -0.34 | 18.8 | 6.28 |
| 541 | C6TC72 | eukaryotic translation initiation factor 3 subunit f | 10 | -0.34 | 31.7 | 5.13 |
| 542 | I1KPN2 | uncharacterized protein | 27 | -0.34 | 96.3 | 4.67 |
| 543 | I1LJ94 | v-type proton atpase subunit a | 17 | -0.34 | 92.9 | 5.80 |
| 544 | I1N5S0 | formate dehydrogenase | 17 | -0.35 | 42.8 | 6.28 |
| 545 | P26690 | nad(p)h-dependent 6'-deoxychalcone synthase | 17 | -0.35 | 35.5 | 6.32 |
| 546 | I1L3P6 | t-complex protein 1 subunit gamma | 20 | -0.35 | 60.2 | 5.92 |
| 547 | I1KB52 | uncharacterized protein | 7 | -0.35 | 37.0 | 9.14 |
| 548 | C6SZN7 | thioredoxin | 5 | -0.35 | 13.0 | 5.29 |
| 549 | I1L645 | uncharacterized protein | 12 | -0.35 | 31.8 | 7.63 |
| 550 | C6TB70 | uncharacterized protein | 9 | -0.36 | 24.4 | 6.44 |
| 551 | C6TCK0 | uncharacterized protein | 2 | -0.36 | 30.2 | 4.36 |
| 552 | Q944T1 | inosine-5'-monophosphate dehydrogenase-like protein | 8 | -0.37 | 41.8 | 6.69 |
| 553 | C6TAJ6 | carboxypeptidase | 3 | -0.37 | 54.4 | 8.66 |
| 554 | I1KT65 | uncharacterized protein | 33 | -0.37 | 229.3 | 5.23 |
| 555 | C6TAT2 | tau class glutathione s-transferase | 6 | -0.37 | 25.6 | 5.57 |
| 556 | I1M0S4 | uncharacterized protein | 9 | -0.37 | 126.5 | 5.75 |
| 557 | I1LQ62 | uncharacterized protein | 19 | -0.37 | 83.7 | 5.57 |
| 558 | A0A0R0I9D1 | uncharacterized protein | 8 | -0.37 | 50.7 | 5.62 |
| 559 | K7MYU5 | eukaryotic translation initiation factor 3 subunit a | 26 | -0.37 | 111.7 | 9.36 |
| 560 | A0A0R0I9G1 | uncharacterized protein | 19 | -0.38 | 116.8 | 9.41 |
| 561 | I1JBN4 | uncharacterized protein | 6 | -0.38 | 26.0 | 6.20 |
| 562 | C6TMN0 | ATP-dependent clp protease proteolytic subunit | 5 | -0.38 | 26.0 | 7.76 |
| 563 | C6SWA9 | uncharacterized protein | 7 | -0.38 | 11.4 | 4.32 |
| 564 | I1JYL3 | uncharacterized protein | 8 | -0.38 | 33.9 | 7.09 |
| 565 | I1JIR8 | uncharacterized protein | 5 | -0.38 | 54.0 | 5.22 |
| 566 | A0A0R4J3S4 | uncharacterized protein | 9 | -0.38 | 29.2 | 4.90 |
| 567 | I1LUL9 | ketol-acid reductoisomerase | 21 | -0.38 | 63.3 | 6.85 |
| 568 | I1KEH3 | uncharacterized protein | 8 | -0.38 | 37.6 | 6.20 |
| 569 | K7L8K5 | uncharacterized protein | 9 | -0.39 | 48.8 | 8.40 |
| 570 | I1LCU6 | uncharacterized protein | 18 | -0.39 | 115.8 | 6.20 |
| 571 | I1NCF0 | uncharacterized protein | 49 | -0.39 | 287.7 | 6.38 |
| 572 | C6TAH8 | uncharacterized protein | 5 | -0.39 | 29.1 | 9.11 |
| 573 | A0A0R0KRW9 | uncharacterized protein | 14 | -0.39 | 80.9 | 5.62 |
| 574 | C6SX98 | uncharacterized protein | 2 | -0.39 | 17.2 | 9.33 |
| 575 | I1N9A4 | uncharacterized protein | 7 | -0.40 | 39.9 | 5.83 |
| 576 | A0A0R4J4C4 | uncharacterized protein | 9 | -0.40 | 31.7 | 6.62 |
| 577 | I1N0Y1 | uncharacterized protein | 18 | -0.40 | 62.9 | 5.71 |
| 578 | A0A0R0HL99 | uncharacterized protein | 30 | -0.40 | 237.3 | 5.44 |
| 579 | A0A0R0HAR3 | uncharacterized protein | 5 | -0.40 | 64.3 | 5.92 |
| 580 | B3TDK6 | lipoxygenase | 55 | -0.40 | 96.7 | 6.12 |
| 581 | A0A0R4J410 | obg-like atpase 1 | 17 | -0.40 | 44.4 | 6.36 |
| 582 | I1MJN2 | uncharacterized protein | 11 | -0.40 | 42.1 | 9.72 |
| 583 | C6SVY0 | uncharacterized protein | 7 | -0.40 | 19.9 | 8.72 |
| 584 | I1JKG5 | ATPase asna1 homolog | 7 | -0.40 | 39.8 | 4.77 |
| 585 | I1MBZ3 | uncharacterized protein | 12 | -0.40 | 98.4 | 5.30 |
| 586 | I1NIH9 | uncharacterized protein | 4 | -0.41 | 104.6 | 5.66 |
| 587 | I1MBI6 | uncharacterized protein | 16 | -0.41 | 109.8 | 5.88 |
| 588 | I1NF56 | uncharacterized protein | 2 | -0.41 | 16.9 | 6.29 |
| 589 | P50346 | 60s acidic ribosomal protein p0 | 11 | -0.41 | 34.1 | 5.13 |
| 590 | C6THW8 | uncharacterized protein | 9 | -0.41 | 28.0 | 6.13 |
| 591 | I1JSR9 | uncharacterized protein | 6 | -0.41 | 78.7 | 5.49 |
| 592 | I1N1W7 | uncharacterized protein | 19 | -0.41 | 58.8 | 6.49 |
| 593 | A0A0R4J3N9 | uncharacterized protein | 9 | -0.41 | 43.9 | 6.18 |
| 594 | I1JB64 | NADH dehydrogenase | 13 | -0.41 | 52.9 | 8.31 |
| 595 | I1KTK7 | clustered mitochondria protein homolog | 18 | -0.41 | 157.2 | 5.93 |
| 596 | C6TGU6 | uncharacterized protein | 6 | -0.41 | 45.1 | 4.71 |
| 597 | I1KB01 | uncharacterized protein | 3 | -0.42 | 41.6 | 6.47 |
| 598 | I1LJG9 | uncharacterized protein | 17 | -0.42 | 83.8 | 5.47 |
| 599 | A0A0R4J455 | uricase | 13 | -0.42 | 35.1 | 8.31 |
| 600 | I1KC56 | uncharacterized protein | 20 | -0.42 | 53.3 | 6.31 |
| 601 | I1MVS9 | uncharacterized protein | 6 | -0.43 | 43.8 | 7.57 |
| 602 | A0A0R0K7P8 | uncharacterized protein | 20 | -0.43 | 110.4 | 5.49 |
| 603 | C6T447 | uncharacterized protein | 7 | -0.43 | 25.1 | 5.98 |
| 604 | I1KEN4 | uncharacterized protein | 34 | -0.43 | 110.2 | 5.52 |
| 605 | I1LQR4 | heat shock 70 kda protein | 34 | -0.43 | 71.0 | 5.10 |
| 606 | C6TFI8 | uncharacterized protein | 4 | -0.43 | 16.7 | 11.02 |
| 607 | I1KK66 | uncharacterized protein | 8 | -0.43 | 25.8 | 5.52 |
| 608 | I1KRI3 | uncharacterized protein | 5 | -0.43 | 43.1 | 6.42 |
| 609 | A0A0R0KF64 | uncharacterized protein | 10 | -0.44 | 47.7 | 8.72 |
| 610 | A0A0R0IXP9 | uncharacterized protein | 5 | -0.44 | 26.3 | 9.40 |
| 611 | I1MUP6 | uncharacterized protein | 12 | -0.44 | 46.4 | 5.00 |
| 612 | A0A0R0F2E8 | uncharacterized protein | 8 | -0.44 | 22.1 | 7.09 |
| 613 | I1JDH6 | uncharacterized protein | 11 | -0.44 | 30.2 | 10.36 |
| 614 | I1J4I1 | phosphotransferase | 9 | -0.44 | 53.6 | 5.96 |
| 615 | A0A0R0EKG7 | uncharacterized protein | 10 | -0.44 | 100.4 | 6.03 |
| 616 | I1LYP8 | uncharacterized protein | 14 | -0.44 | 43.8 | 9.22 |
| 617 | I1L3P0 | uncharacterized protein | 2 | -0.44 | 36.8 | 8.93 |
| 618 | I1MJC7 | phosphoglycerate kinase | 22 | -0.44 | 42.4 | 5.96 |
| 619 | I1NBD7 | uncharacterized protein | 23 | -0.44 | 130.0 | 5.46 |
| 620 | I1J881 | uncharacterized protein | 2 | -0.45 | 13.9 | 10.18 |
| 621 | I1MJD7 | inosine-5'-monophosphate dehydrogenase | 11 | -0.45 | 53.3 | 5.77 |
| 622 | K7LK48 | uncharacterized protein | 10 | -0.45 | 70.5 | 6.18 |
| 623 | C6T1E4 | uncharacterized protein | 6 | -0.45 | 11.4 | 4.27 |
| 624 | A0A0R0IY29 | uncharacterized protein | 14 | -0.45 | 54.4 | 5.86 |
| 625 | A0A0R0GHB3 | uncharacterized protein | 22 | -0.45 | 90.4 | 5.13 |
| 626 | I1MF39 | uncharacterized protein | 7 | -0.45 | 43.0 | 4.67 |
| 627 | I1NBE6 | uncharacterized protein | 17 | -0.45 | 82.1 | 7.00 |
| 628 | I1LV40 | uncharacterized protein | 10 | -0.46 | 39.2 | 8.68 |
| 629 | I1L1K9 | glycylpeptide n-tetradecanoyltransferase | 6 | -0.46 | 49.5 | 6.18 |
| 630 | I1L5T1 | eukaryotic translation initiation factor 3 subunit i | 12 | -0.46 | 35.8 | 6.26 |
| 631 | C6SY82 | eukaryotic translation initiation factor 5a | 5 | -0.46 | 17.3 | 5.77 |
| 632 | I1LTQ5 | uncharacterized protein | 21 | -0.46 | 113.7 | 5.88 |
| 633 | K7KQN8 | uncharacterized protein | 11 | -0.46 | 83.5 | 5.87 |
| 634 | I1KY39 | citrate synthase | 13 | -0.46 | 56.4 | 9.22 |
| 635 | C6THU9 | uncharacterized protein | 6 | -0.46 | 34.5 | 6.99 |
| 636 | I1M903 | uncharacterized protein | 11 | -0.46 | 69.1 | 5.54 |
| 637 | I1KY36 | uncharacterized protein | 12 | -0.47 | 54.5 | 5.75 |
| 638 | C6T4P6 | uncharacterized protein | 5 | -0.47 | 20.1 | 7.59 |
| 639 | A0A0R4J4L8 | uncharacterized protein | 17 | -0.47 | 47.4 | 5.42 |
| 640 | I1MVA1 | uncharacterized protein | 8 | -0.47 | 45.4 | 5.17 |
| 641 | I1LLM2 | uncharacterized protein | 20 | -0.47 | 65.2 | 6.16 |
| 642 | I1N1Y6 | 40s ribosomal protein s8 | 9 | -0.47 | 24.7 | 10.40 |
| 643 | I1JUY7 | ATP-dependent 6-phosphofructokinase | 12 | -0.48 | 56.0 | 6.33 |
| 644 | I1LLG6 | uncharacterized protein | 4 | -0.48 | 179.7 | 5.77 |
| 645 | I1MY65 | uncharacterized protein | 12 | -0.48 | 59.9 | 8.89 |
| 646 | K7MT90 | uncharacterized protein | 16 | -0.48 | 35.1 | 5.76 |
| 647 | I1KQB6 | uncharacterized protein | 6 | -0.48 | 80.9 | 5.71 |
| 648 | I1LCC3 | uncharacterized protein | 4 | -0.48 | 27.7 | 7.67 |
| 649 | I1LD52 | uncharacterized protein | 15 | -0.49 | 63.2 | 6.11 |
| 650 | I1KZR0 | uncharacterized protein | 22 | -0.49 | 100.1 | 9.12 |
| 651 | A0A0R0IQZ2 | uncharacterized protein | 11 | -0.49 | 84.8 | 5.62 |
| 652 | K7LKS0 | uncharacterized protein | 6 | -0.49 | 121.7 | 5.29 |
| 653 | I1KZY9 | uncharacterized protein | 14 | -0.49 | 45.8 | 5.85 |
| 654 | K7LSP6 | uncharacterized protein | 2 | -0.49 | 37.2 | 4.89 |
| 655 | A0A0R0GUR9 | uncharacterized protein | 9 | -0.49 | 55.8 | 6.31 |
| 656 | I1LEV4 | uncharacterized protein | 22 | -0.49 | 90.2 | 6.03 |
| 657 | I1JVJ8 | uncharacterized protein | 2 | -0.50 | 54.4 | 8.89 |
| 658 | K7M1C2 | uncharacterized protein | 10 | -0.50 | 129.0 | 6.19 |
| 659 | I1L314 | heat shock protein 90-1 | 27 | -0.50 | 80.3 | 4.95 |
| 660 | I1JV14 | uncharacterized protein | 16 | -0.50 | 74.4 | 6.37 |
| 661 | I1N520 | uncharacterized protein | 21 | -0.50 | 35.1 | 6.12 |
| 662 | I1JXQ5 | alpha-mannosidase | 17 | -0.50 | 114.2 | 6.30 |
| 663 | Q8H928 | phosphoenolpyruvate carboxylase | 43 | -0.50 | 110.6 | 6.05 |
| 664 | I1KHU8 | uncharacterized protein | 8 | -0.50 | 50.7 | 8.87 |
| 665 | I1MXM6 | polyadenylate-binding protein | 13 | -0.50 | 71.9 | 6.70 |
| 666 | I1NCV6 | uncharacterized protein | 11 | -0.50 | 174.4 | 5.66 |
| 667 | K7MIR3 | uncharacterized protein | 17 | -0.50 | 38.3 | 6.35 |
| 668 | I1M1I0 | uncharacterized protein | 23 | -0.50 | 181.7 | 6.21 |
| 669 | I1M0K3 | cysteine proteinase inhibitor | 9 | -0.51 | 27.7 | 6.10 |
| 670 | C6TGE9 | reticulon-like protein | 5 | -0.51 | 28.8 | 6.80 |
| 671 | K7KL44 | glyceraldehyde-3-phosphate dehydrogenase | 21 | -0.51 | 46.7 | 7.27 |
| 672 | C6TH90 | ATP-dependent (s)-nad(p)h-hydrate dehydratase | 7 | -0.51 | 39.6 | 8.28 |
| 673 | I1K7H3 | uncharacterized protein | 3 | -0.51 | 27.4 | 8.78 |
| 674 | K7MUT7 | uncharacterized protein | 7 | -0.51 | 57.8 | 6.77 |
| 675 | I1JR56 | uncharacterized protein | 6 | -0.51 | 19.5 | 6.14 |
| 676 | I1LSW3 | uncharacterized protein | 9 | -0.51 | 50.1 | 5.42 |
| 677 | I1LVI3 | uncharacterized protein | 19 | -0.51 | 100.1 | 9.13 |
| 678 | I1N1K1 | uncharacterized protein | 8 | -0.52 | 49.2 | 5.94 |
| 679 | I1LAQ3 | uncharacterized protein | 7 | -0.52 | 57.0 | 6.03 |
| 680 | I1L6T8 | uncharacterized protein | 7 | -0.52 | 39.9 | 6.26 |
| 681 | I1LE85 | uncharacterized protein | 5 | -0.52 | 37.4 | 9.40 |
| 682 | I1LGM2 | uncharacterized protein | 19 | -0.52 | 65.5 | 6.26 |
| 683 | P18663 | 50s ribosomal protein l2-a | 4 | -0.52 | 29.8 | 10.80 |
| 684 | K7MX62 | uncharacterized protein | 4 | -0.52 | 17.0 | 9.35 |
| 685 | C6SXG5 | uncharacterized protein | 4 | -0.53 | 15.8 | 6.91 |
| 686 | C6TFV2 | uncharacterized protein | 4 | -0.53 | 22.5 | 7.37 |
| 687 | Q9S7N8 | seed maturation protein pm21 | 2 | -0.53 | 10.1 | 4.91 |
| 688 | I1M0N4 | uncharacterized protein | 11 | -0.53 | 124.1 | 5.70 |
| 689 | A0A0R0G6V6 | exocyst complex component | 4 | -0.53 | 92.7 | 6.20 |
| 690 | K7KA83 | uncharacterized protein | 21 | -0.53 | 120.5 | 5.09 |
| 691 | K7LQ69 | poly [adp-ribose] polymerase | 33 | -0.53 | 91.1 | 5.28 |
| 692 | C6TMQ6 | uncharacterized protein | 9 | -0.53 | 44.0 | 9.21 |
| 693 | G3ECQ4 | rfls1 protein | 8 | -0.53 | 69.0 | 6.26 |
| 694 | I1KK35 | uncharacterized protein | 3 | -0.53 | 28.5 | 4.60 |
| 695 | K7L2B3 | uncharacterized protein | 9 | -0.53 | 96.9 | 5.56 |
| 696 | C6TDX5 | uncharacterized protein | 8 | -0.54 | 42.9 | 5.96 |
| 697 | K7MAQ6 | uncharacterized protein | 3 | -0.54 | 35.4 | 8.77 |
| 698 | I1K064 | protein-l-isoaspartate o-methyltransferase | 4 | -0.54 | 24.8 | 5.85 |
| 699 | K7LRH4 | uncharacterized protein | 10 | -0.55 | 32.2 | 9.23 |
| 700 | I1LIT2 | uncharacterized protein | 11 | -0.55 | 78.3 | 5.44 |
| 701 | Q5QJB6 | harpin binding protein 1 | 5 | -0.55 | 28.4 | 7.88 |
| 702 | K7K525 | uncharacterized protein | 12 | -0.55 | 42.1 | 6.27 |
| 703 | C6T9B1 | uncharacterized protein | 12 | -0.56 | 31.4 | 5.99 |
| 704 | I1KBN0 | uncharacterized protein | 10 | -0.56 | 50.7 | 5.57 |
| 705 | I1JF25 | uncharacterized protein | 7 | -0.56 | 42.1 | 6.86 |
| 706 | I1K7W5 | uncharacterized protein | 9 | -0.56 | 42.8 | 5.16 |
| 707 | I1M6L4 | uncharacterized protein | 6 | -0.56 | 53.2 | 4.71 |
| 708 | C6TLS9 | uncharacterized protein | 4 | -0.56 | 19.0 | 8.68 |
| 709 | I1NE40 | uncharacterized protein | 25 | -0.56 | 91.4 | 5.56 |
| 710 | I1K4M7 | uncharacterized protein | 15 | -0.56 | 70.8 | 5.13 |
| 711 | I1KW16 | uncharacterized protein | 3 | -0.56 | 9.3 | 9.21 |
| 712 | I1LA46 | uncharacterized protein | 5 | -0.56 | 30.6 | 6.20 |
| 713 | C6TFH7 | uncharacterized protein | 3 | -0.57 | 20.2 | 9.33 |
| 714 | Q2I0H4 | glyceraldehyde-3-phosphate dehydrogenase | 21 | -0.57 | 36.7 | 6.72 |
| 715 | K7N1Q0 | uncharacterized protein | 9 | -0.57 | 106.1 | 8.93 |
| 716 | C6SVG5 | peptidyl-prolyl cis-trans isomerase | 7 | -0.57 | 21.0 | 8.41 |
| 717 | I1KS98 | uncharacterized protein | 10 | -0.57 | 46.7 | 6.02 |
| 718 | I1NIY6 | uncharacterized protein | 18 | -0.57 | 51.4 | 6.04 |
| 719 | I1JMW9 | uncharacterized protein | 13 | -0.58 | 271.0 | 5.86 |
| 720 | Q42797 | trans-cinnamate 4-monooxygenase | 16 | -0.58 | 58.0 | 9.05 |
| 721 | I1L3K7 | uncharacterized protein | 19 | -0.58 | 48.0 | 5.92 |
| 722 | Q39873 | lea protein | 17 | -0.58 | 49.4 | 6.72 |
| 723 | I1KP14 | uncharacterized protein | 30 | -0.58 | 108.7 | 6.15 |
| 724 | C5HU39 | air12 | 3 | -0.58 | 25.6 | 7.98 |
| 725 | I1LJE7 | uncharacterized protein | 2 | -0.58 | 113.1 | 5.00 |
| 726 | I1J7H3 | ferritin | 7 | -0.58 | 28.7 | 5.77 |
| 727 | I1JPD4 | eukaryotic translation initiation factor 3 subunit a | 24 | -0.58 | 111.9 | 9.33 |
| 728 | I1JSX8 | uncharacterized protein | 11 | -0.58 | 60.6 | 9.42 |
| 729 | C6T9F2 | uncharacterized protein | 3 | -0.58 | 33.8 | 4.86 |
| 730 | I1KRD5 | uncharacterized protein | 7 | -0.58 | 14.9 | 5.60 |
| 731 | I1K7F4 | uncharacterized protein | 9 | -0.59 | 60.8 | 9.38 |
| 732 | I1MCE2 | signal recognition particle 54 kda protein | 8 | -0.59 | 55.2 | 9.23 |
| 733 | I1N877 | h/aca ribonucleoprotein complex subunit | 3 | -0.59 | 20.7 | 11.49 |
| 734 | I1M7Y3 | adenylosuccinate lyase | 8 | -0.59 | 59.3 | 5.96 |
| 735 | I1ND31 | uncharacterized protein | 12 | -0.59 | 42.8 | 6.90 |
| 736 | C6SW69 | uncharacterized protein | 7 | -0.60 | 23.2 | 5.77 |
| 737 | I1KYS4 | uncharacterized protein | 19 | -0.60 | 203.5 | 6.82 |
| 738 | Q9SWA8 | glycine-rich rna-binding protein | 8 | -0.60 | 15.8 | 6.58 |
| 739 | I1LD65 | dihydrolipoamide acetyltransferase component of pyruvate dehydrogenase complex | 9 | -0.60 | 48.9 | 8.84 |
| 740 | I1JTG3 | uncharacterized protein | 13 | -0.60 | 42.7 | 6.13 |
| 741 | I1JQZ7 | uncharacterized protein | 19 | -0.60 | 145.1 | 6.76 |
| 742 | I1NF42 | uncharacterized protein | 4 | -0.60 | 43.5 | 4.67 |
| 743 | I1KEL5 | uncharacterized protein | 12 | -0.60 | 97.7 | 5.85 |
| 744 | I1LKT2 | uncharacterized protein | 5 | -0.60 | 48.5 | 8.46 |
| 745 | I1MQS2 | uncharacterized protein | 2 | -0.60 | 30.9 | 7.71 |
| 746 | I1KIE4 | uncharacterized protein | 5 | -0.60 | 32.2 | 6.35 |
| 747 | C6SXJ9 | cytochrome b-c1 complex subunit 7 | 5 | -0.60 | 14.6 | 9.61 |
| 748 | I1MST3 | uncharacterized protein | 2 | -0.60 | 64.2 | 5.63 |
| 749 | I1M7K1 | uncharacterized protein | 6 | -0.61 | 30.1 | 6.99 |
| 750 | I1J717 | proteasome subunit alpha type | 12 | -0.61 | 25.6 | 5.48 |
| 751 | I1KJQ5 | eukaryotic translation initiation factor 3 subunit b | 23 | -0.61 | 82.6 | 5.14 |
| 752 | I1NJ39 | uncharacterized protein | 6 | -0.61 | 124.7 | 6.97 |
| 753 | K7MNV4 | uncharacterized protein | 4 | -0.61 | 22.1 | 5.13 |
| 754 | C6TMI1 | serine/threonine-protein phosphatase | 7 | -0.62 | 36.9 | 5.49 |
| 755 | A0A0R0GUV6 | uncharacterized protein | 2 | -0.62 | 17.6 | 9.30 |
| 756 | I1NEX1 | phosphoacetylglucosamine mutase | 5 | -0.62 | 60.7 | 5.45 |
| 757 | I1JJW5 | uncharacterized protein | 4 | -0.63 | 61.5 | 5.53 |
| 758 | I1NB34 | uncharacterized protein | 4 | -0.63 | 86.1 | 6.63 |
| 759 | I1LSZ7 | proteasome subunit alpha type | 13 | -0.63 | 27.4 | 5.58 |
| 760 | K7MJ68 | uncharacterized protein | 2 | -0.63 | 33.5 | 7.09 |
| 761 | I1LRC2 | histone deacetylase hdt1 | 5 | -0.63 | 31.7 | 4.75 |
| 762 | C6T460 | tau class glutathione s-transferase | 9 | -0.63 | 25.0 | 5.69 |
| 763 | I1KZW4 | uncharacterized protein | 11 | -0.63 | 88.9 | 5.06 |
| 764 | C6TGU2 | proteasome subunit alpha type | 10 | -0.63 | 26.0 | 4.70 |
| 765 | A0A0R0JVZ4 | uncharacterized protein | 17 | -0.63 | 66.2 | 8.23 |
| 766 | A0A0R4J571 | uncharacterized protein | 2 | -0.64 | 31.1 | 5.71 |
| 767 | I1MPI1 | uncharacterized protein | 27 | -0.64 | 80.2 | 4.97 |
| 768 | I1LD03 | dolichyl-diphosphooligosaccharide--protein glycosyltransferase subunit 1 | 11 | -0.65 | 52.7 | 6.79 |
| 769 | A0A0R0IRU4 | uncharacterized protein | 5 | -0.65 | 127.8 | 4.51 |
| 770 | I1KQ25 | frigida-like protein | 5 | -0.65 | 57.6 | 9.13 |
| 771 | C6THB8 | uncharacterized protein | 9 | -0.65 | 50.1 | 4.72 |
| 772 | I1KEV1 | uncharacterized protein | 2 | -0.66 | 72.5 | 6.36 |
| 773 | K7KRC0 | uncharacterized protein | 10 | -0.66 | 48.2 | 5.41 |
| 774 | A0A0R0EQE5 | uncharacterized protein | 9 | -0.66 | 70.3 | 5.68 |
| 775 | I1NIY9 | mitochondrial rho gtpase | 16 | -0.66 | 71.9 | 5.36 |
| 776 | K7MPH5 | uncharacterized protein | 11 | -0.66 | 56.1 | 8.04 |
| 777 | C6T3U6 | ribosomal protein l19 | 2 | -0.67 | 24.8 | 11.45 |
| 778 | I1MRP7 | uncharacterized protein | 5 | -0.67 | 100.2 | 5.17 |
| 779 | C6TCR3 | uncharacterized protein | 4 | -0.67 | 14.6 | 6.28 |
| 780 | I1KJH7 | uncharacterized protein | 3 | -0.67 | 20.8 | 6.91 |
| 781 | K7KQP0 | uncharacterized protein | 7 | -0.67 | 52.2 | 9.07 |
| 782 | C6TAV3 | uncharacterized protein | 6 | -0.67 | 30.4 | 6.40 |
| 783 | I1JCB1 | uncharacterized protein | 9 | -0.68 | 39.5 | 7.63 |
| 784 | K7KWT9 | uncharacterized protein | 8 | -0.68 | 44.9 | 7.82 |
| 785 | I1JR71 | importin subunit alpha | 17 | -0.68 | 58.8 | 5.23 |
| 786 | C6TDZ1 | uncharacterized protein | 5 | -0.68 | 30.7 | 6.19 |
| 787 | A0A0R0F381 | uncharacterized protein | 3 | -0.68 | 59.5 | 5.24 |
| 788 | I1JMZ6 | uncharacterized protein | 7 | -0.69 | 188.4 | 4.95 |
| 789 | I1KQE2 | uncharacterized protein | 5 | -0.69 | 34.6 | 7.68 |
| 790 | Q7G1G6 | aspartate aminotransferase | 22 | -0.69 | 45.6 | 7.72 |
| 791 | C6T0P8 | uncharacterized protein | 3 | -0.69 | 15.1 | 6.59 |
| 792 | I1MFP3 | inositol-1-monophosphatase | 5 | -0.69 | 29.1 | 4.99 |
| 793 | A0A0R0EM71 | uncharacterized protein | 13 | -0.69 | 62.8 | 6.01 |
| 794 | I1KJK0 | uncharacterized protein | 9 | -0.70 | 39.3 | 5.95 |
| 795 | I1KGC2 | protein root hair defective 3 homolog | 15 | -0.70 | 89.7 | 5.40 |
| 796 | I1MRS5 | DNA helicase | 6 | -0.70 | 106.6 | 5.13 |
| 797 | I1JW13 | uncharacterized protein | 7 | -0.70 | 30.4 | 7.95 |
| 798 | I1JR88 | uncharacterized protein | 6 | -0.71 | 35.9 | 5.34 |
| 799 | I1KW20 | uncharacterized protein | 6 | -0.71 | 30.5 | 7.96 |
| 800 | I1LFD7 | uncharacterized protein | 14 | -0.71 | 134.9 | 5.35 |
| 801 | I1MPF4 | glycosyltransferase | 7 | -0.71 | 53.7 | 6.37 |
| 802 | I1LIF6 | uncharacterized protein | 8 | -0.71 | 81.0 | 5.67 |
| 803 | K7MQ57 | uncharacterized protein | 3 | -0.71 | 115.9 | 5.76 |
| 804 | A0A0R0ENW6 | uncharacterized protein | 9 | -0.71 | 46.3 | 8.73 |
| 805 | K7KGX2 | uncharacterized protein | 6 | -0.71 | 46.3 | 5.59 |
| 806 | I1KXE7 | uncharacterized protein | 18 | -0.71 | 64.2 | 4.86 |
| 807 | I1N3E1 | uncharacterized protein | 5 | -0.72 | 46.9 | 8.35 |
| 808 | I1K7A1 | dolichyl-diphosphooligosaccharide--protein glycosyltransferase subunit 1 | 14 | -0.72 | 68.4 | 6.62 |
| 809 | A0A0R0KKK6 | NADH-cytochrome b5 reductase | 8 | -0.72 | 30.8 | 8.23 |
| 810 | A0A0R4J3Q7 | 18.5 kda class i heat shock protein | 5 | -0.72 | 18.5 | 5.99 |
| 811 | I1LSY9 | uncharacterized protein | 9 | -0.72 | 27.6 | 5.72 |
| 812 | I1MXW9 | uncharacterized protein | 7 | -0.72 | 78.1 | 5.41 |
| 813 | K7LKF8 | histone h2b | 2 | -0.72 | 22.6 | 10.00 |
| 814 | I1LUJ6 | uncharacterized protein | 8 | -0.72 | 37.1 | 9.64 |
| 815 | I1JTW7 | glycine cleavage system p protein | 19 | -0.73 | 111.9 | 6.97 |
| 816 | I1M1W3 | uncharacterized protein | 3 | -0.73 | 27.7 | 5.00 |
| 817 | I1N3E7 | uncharacterized protein | 5 | -0.74 | 46.5 | 8.64 |
| 818 | C6SW17 | uncharacterized protein | 3 | -0.74 | 12.0 | 10.59 |
| 819 | A0A0R0KR16 | uncharacterized protein | 5 | -0.74 | 13.8 | 9.61 |
| 820 | I1NH15 | uncharacterized protein | 13 | -0.74 | 147.9 | 5.20 |
| 821 | A0A0R0IJV1 | uncharacterized protein | 7 | -0.75 | 36.9 | 8.39 |
| 822 | I1JUT9 | uncharacterized protein | 20 | -0.75 | 110.3 | 6.49 |
| 823 | I1NC67 | cysteine synthase | 12 | -0.75 | 34.3 | 5.50 |
| 824 | C6TMK3 | cysteine proteinase inhibitor | 8 | -0.75 | 27.6 | 6.40 |
| 825 | I1JP06 | uncharacterized protein | 10 | -0.75 | 56.4 | 5.06 |
| 826 | I1KYU6 | alpha-1,4 glucan phosphorylase | 27 | -0.75 | 95.8 | 6.03 |
| 827 | A0A0R0J475 | uncharacterized protein | 11 | -0.76 | 79.2 | 7.39 |
| 828 | I1J7C8 | uncharacterized protein | 10 | -0.76 | 42.9 | 6.30 |
| 829 | I1MKG5 | uncharacterized protein | 2 | -0.76 | 31.2 | 10.01 |
| 830 | V6CKR0 | expansin | 5 | -0.76 | 27.5 | 9.16 |
| 831 | K7M988 | uncharacterized protein | 6 | -0.77 | 73.4 | 8.95 |
| 832 | C6SV69 | uncharacterized protein | 3 | -0.77 | 17.8 | 6.62 |
| 833 | I1JMZ7 | uncharacterized protein | 18 | -0.77 | 111.5 | 5.75 |
| 834 | I1MBM5 | uncharacterized protein | 2 | -0.77 | 8.3 | 9.65 |
| 835 | I1NJC2 | uncharacterized protein | 8 | -0.77 | 66.5 | 6.15 |
| 836 | I1KBE3 | uncharacterized protein | 3 | -0.78 | 27.0 | 10.12 |
| 837 | C6TBD2 | vacuolar protein sorting-associated protein 28 homolog | 2 | -0.78 | 24.0 | 5.32 |
| 838 | Q948P5 | ferritin-4 | 11 | -0.78 | 27.5 | 5.21 |
| 839 | I1JYB3 | uncharacterized protein | 4 | -0.78 | 105.3 | 6.02 |
| 840 | I1MU56 | uncharacterized protein | 18 | -0.78 | 82.2 | 5.82 |
| 841 | I1KV08 | uncharacterized protein | 15 | -0.78 | 54.4 | 5.84 |
| 842 | I1JKB0 | uncharacterized protein | 8 | -0.78 | 57.5 | 8.76 |
| 843 | I1NEZ7 | lon protease homolog | 13 | -0.79 | 109.1 | 5.58 |
| 844 | A0A0R0GIX5 | nicotinamide-nucleotide adenylyltransferase | 3 | -0.79 | 31.7 | 6.25 |
| 845 | K7M3Y6 | uncharacterized protein | 12 | -0.79 | 45.9 | 6.10 |
| 846 | K7LRU6 | uncharacterized protein | 8 | -0.79 | 77.8 | 4.76 |
| 847 | C6SVH2 | uncharacterized protein | 3 | -0.79 | 20.7 | 4.46 |
| 848 | I1KMS6 | uncharacterized protein | 4 | -0.80 | 19.0 | 6.96 |
| 849 | K7K2H1 | uncharacterized protein | 4 | -0.80 | 74.1 | 4.71 |
| 850 | I1K146 | uncharacterized protein | 11 | -0.80 | 33.9 | 6.72 |
| 851 | I1LBB9 | lipoxygenase | 17 | -0.80 | 98.1 | 5.62 |
| 852 | I1KS56 | uncharacterized protein | 24 | -0.80 | 100.3 | 7.63 |
| 853 | A0A0R0L894 | uncharacterized protein | 7 | -0.80 | 70.6 | 9.17 |
| 854 | Q2PMP7 | 30s ribosomal protein s3 | 5 | -0.80 | 24.7 | 9.93 |
| 855 | I1LDP2 | uncharacterized protein | 7 | -0.80 | 27.9 | 9.00 |
| 856 | I1MIW3 | ribokinase | 5 | -0.80 | 38.0 | 6.65 |
| 857 | I1JRW5 | uncharacterized protein | 2 | -0.81 | 16.4 | 5.08 |
| 858 | I1JT75 | thioredoxin | 6 | -0.81 | 13.1 | 5.24 |
| 859 | I1M5N2 | uncharacterized protein | 11 | -0.81 | 103.0 | 7.01 |
| 860 | A0A0R0EWR2 | uncharacterized protein | 7 | -0.81 | 52.9 | 6.33 |
| 861 | I1LGI8 | uncharacterized protein | 3 | -0.81 | 79.2 | 4.43 |
| 862 | I1MVW0 | uncharacterized protein | 4 | -0.82 | 49.4 | 6.74 |
| 863 | I1KZ41 | glycosyltransferase | 5 | -0.82 | 54.3 | 6.27 |
| 864 | C6TN36 | glyceraldehyde-3-phosphate dehydrogenase | 19 | -0.82 | 37.0 | 6.97 |
| 865 | A0A0R0EVL3 | delta-1-pyrroline-5-carboxylate synthase | 9 | -0.82 | 78.0 | 6.57 |
| 866 | K7LRU2 | uncharacterized protein | 10 | -0.82 | 33.2 | 10.05 |
| 867 | I1L922 | uncharacterized protein | 8 | -0.82 | 92.8 | 6.03 |
| 868 | I1MCF0 | uncharacterized protein | 10 | -0.83 | 45.2 | 5.97 |
| 869 | C6ZS00 | disease resistance protein/lrr protein-related protein | 9 | -0.83 | 53.8 | 8.61 |
| 870 | C6SYI9 | uncharacterized protein | 3 | -0.83 | 22.6 | 5.05 |
| 871 | I1KGY6 | uncharacterized protein | 16 | -0.84 | 80.7 | 5.40 |
| 872 | I1LS05 | uncharacterized protein | 10 | -0.84 | 45.8 | 6.57 |
| 873 | A0A0R0JTV5 | elongation factor 1-alpha | 16 | -0.84 | 49.2 | 9.15 |
| 874 | A0A0R0L273 | uncharacterized protein | 8 | -0.85 | 23.9 | 7.64 |
| 875 | I1LZZ5 | uncharacterized protein | 2 | -0.85 | 37.1 | 9.83 |
| 876 | I1L974 | uncharacterized protein | 3 | -0.85 | 57.6 | 7.64 |
| 877 | C6SWA6 | histone h2a | 3 | -0.85 | 15.7 | 10.66 |
| 878 | I1KQ70 | uncharacterized protein | 22 | -0.86 | 87.5 | 6.18 |
| 879 | I1MM85 | uncharacterized protein | 14 | -0.86 | 58.5 | 6.26 |
| 880 | I1KYS8 | uncharacterized protein | 4 | -0.86 | 41.1 | 7.69 |
| 881 | I1JAE5 | uncharacterized protein | 12 | -0.86 | 68.3 | 7.29 |
| 882 | I1KZI4 | uncharacterized protein | 8 | -0.87 | 50.3 | 5.69 |
| 883 | I1M222 | uncharacterized protein | 9 | -0.87 | 25.6 | 5.50 |
| 884 | I1KWD8 | uncharacterized protein | 7 | -0.87 | 49.8 | 5.09 |
| 885 | A0A0R4J3X4 | uncharacterized protein | 4 | -0.87 | 25.6 | 6.92 |
| 886 | B5L5C7 | isoflavone synthase 1 | 18 | -0.87 | 58.9 | 9.11 |
| 887 | I1LFH9 | uncharacterized protein | 7 | -0.87 | 219.3 | 8.37 |
| 888 | K7LLW8 | uncharacterized protein | 16 | -0.87 | 117.2 | 8.18 |
| 889 | K7MTY9 | hexosyltransferase | 3 | -0.88 | 71.1 | 7.98 |
| 890 | I1K1B1 | uncharacterized protein | 8 | -0.88 | 283.3 | 5.73 |
| 891 | I1L849 | uncharacterized protein | 8 | -0.88 | 27.4 | 5.16 |
| 892 | D4Q9Z5 | soyasaponin iii rhamnosyltransferase | 15 | -0.90 | 53.5 | 5.87 |
| 893 | C6TM54 | uncharacterized protein | 11 | -0.91 | 40.1 | 8.37 |
| 894 | I1NA94 | uncharacterized protein | 14 | -0.91 | 53.9 | 6.36 |
| 895 | C6T3K0 | uncharacterized protein | 2 | -0.91 | 22.5 | 4.83 |
| 896 | A0A0R4J594 | peroxidase | 7 | -0.91 | 34.8 | 8.14 |
| 897 | I1KHX8 | uncharacterized protein | 4 | -0.91 | 38.2 | 9.35 |
| 898 | K7M540 | uncharacterized protein | 3 | -0.91 | 58.6 | 5.44 |
| 899 | A0A0R0F5Q2 | uncharacterized protein | 4 | -0.92 | 33.5 | 9.36 |
| 900 | A0A0R0FC89 | ATP-dependent clp protease proteolytic subunit | 4 | -0.92 | 38.5 | 9.69 |
| 901 | I1JSN6 | uncharacterized protein | 2 | -0.93 | 19.9 | 5.90 |
| 902 | I1L0S5 | uncharacterized protein | 5 | -0.93 | 19.0 | 6.42 |
| 903 | I1KRJ0 | 6,7-dimethyl-8-ribityllumazine synthase | 2 | -0.93 | 24.2 | 6.70 |
| 904 | I1KRU1 | uncharacterized protein | 6 | -0.93 | 44.3 | 6.33 |
| 905 | E9KNA6 | 14-3-3-like protein a | 13 | -0.94 | 29.0 | 4.70 |
| 906 | I1N058 | uncharacterized protein | 19 | -0.95 | 203.2 | 6.70 |
| 907 | I1LKY8 | uncharacterized protein | 5 | -0.95 | 61.5 | 6.65 |
| 908 | K7MQM3 | uncharacterized protein | 9 | -0.95 | 46.5 | 4.55 |
| 909 | I1N9J8 | uncharacterized protein | 6 | -0.96 | 36.4 | 4.78 |
| 910 | I1M3A7 | uncharacterized protein | 5 | -0.96 | 63.8 | 5.55 |
| 911 | K7LB13 | uncharacterized protein | 2 | -0.96 | 40.4 | 6.95 |
| 912 | K7LXR3 | uncharacterized protein | 6 | -0.97 | 83.1 | 6.46 |
| 913 | K7LAM2 | ubiquinone biosynthesis monooxygenase coq6 | 6 | -0.97 | 55.8 | 8.26 |
| 914 | I1L0Z7 | uncharacterized protein | 5 | -0.97 | 168.3 | 7.29 |
| 915 | A0A0R0L186 | uncharacterized protein | 6 | -0.98 | 17.3 | 6.76 |
| 916 | K7MAB8 | uncharacterized protein | 9 | -0.98 | 118.3 | 6.02 |
| 917 | I1MUR0 | uncharacterized protein | 6 | -0.99 | 65.5 | 4.62 |
| 918 | C6T2G1 | uncharacterized protein | 3 | -0.99 | 26.4 | 5.42 |
| 919 | I1JHP9 | uncharacterized protein | 8 | -1.00 | 22.7 | 8.71 |
| 920 | C6T374 | uncharacterized protein | 7 | -1.00 | 15.4 | 5.41 |
| 921 | K7MYR4 | uncharacterized protein | 5 | -1.01 | 49.6 | 4.99 |
| 922 | I1KLU0 | mitogen-activated protein kinase | 3 | -1.01 | 49.9 | 5.62 |
| 923 | I1LQN8 | uncharacterized protein | 6 | -1.02 | 89.3 | 7.96 |
| 924 | I1KC06 | uncharacterized protein | 5 | -1.02 | 60.8 | 5.97 |
| 925 | C6TJY3 | peroxidase | 3 | -1.02 | 36.0 | 9.77 |
| 926 | K7KEY6 | uncharacterized protein | 2 | -1.02 | 73.6 | 8.17 |
| 927 | C6T1S9 | uncharacterized protein os=glycine max pe=2 sv=1 | 2 | -1.03 | 13.6 | 10.45 |
| 928 | C6TJ04 | uncharacterized protein | 5 | -1.05 | 36.2 | 4.86 |
| 929 | C6TJN4 | uncharacterized protein | 4 | -1.05 | 31.4 | 9.22 |
| 930 | K7L5V1 | signal recognition particle subunit srp72 | 12 | -1.05 | 72.9 | 9.08 |
| 931 | I1KUU8 | uncharacterized protein | 6 | -1.05 | 56.2 | 8.95 |
| 932 | I1J922 | uncharacterized protein | 2 | -1.05 | 51.4 | 6.77 |
| 933 | C6SX29 | uncharacterized protein | 3 | -1.05 | 16.6 | 4.62 |
| 934 | I1J8D4 | uncharacterized protein | 9 | -1.06 | 57.2 | 4.77 |
| 935 | K7K9Y5 | uncharacterized protein | 17 | -1.06 | 401.4 | 5.12 |
| 936 | A0A0R0KL81 | uncharacterized protein | 4 | -1.07 | 80.9 | 6.65 |
| 937 | A0A0R0KKU3 | uncharacterized protein | 35 | -1.07 | 71.4 | 5.04 |
| 938 | I1LBC8 | mitochondrial rho gtpase | 15 | -1.08 | 71.9 | 5.40 |
| 939 | I1KVJ8 | uncharacterized protein | 5 | -1.08 | 26.5 | 6.14 |
| 940 | I1LF33 | mitochondrial pyruvate carrier | 2 | -1.08 | 12.0 | 9.10 |
| 941 | I1LGB8 | uncharacterized protein | 4 | -1.08 | 57.1 | 6.33 |
| 942 | I1M5B6 | beta-galactosidase | 8 | -1.08 | 80.1 | 7.65 |
| 943 | A0A0R0K553 | uncharacterized protein | 7 | -1.08 | 34.8 | 5.42 |
| 944 | I1KG51 | uncharacterized protein | 4 | -1.08 | 84.8 | 5.16 |
| 945 | I1L602 | uncharacterized protein | 9 | -1.08 | 29.7 | 8.57 |
| 946 | I1LFY1 | uncharacterized protein | 4 | -1.08 | 140.0 | 6.36 |
| 947 | I1M368 | probable bifunctional methylthioribulose-1-phosphate dehydratase/enolase-phosphatase e1 | 4 | -1.09 | 57.4 | 5.96 |
| 948 | A0A0R0IIS7 | uncharacterized protein | 6 | -1.10 | 45.4 | 8.21 |
| 949 | I1NA37 | uncharacterized protein | 4 | -1.11 | 21.0 | 6.45 |
| 950 | B8XJZ3 | cold-regulated protein | 2 | -1.11 | 30.8 | 6.15 |
| 951 | K7MJZ6 | uncharacterized protein | 6 | -1.12 | 99.3 | 4.47 |
| 952 | I1L7W1 | uncharacterized protein | 6 | -1.13 | 167.6 | 8.42 |
| 953 | O23957 | dehydrin | 3 | -1.13 | 17.3 | 9.22 |
| 954 | Q9XET0 | seed maturation protein pm30 | 10 | -1.13 | 15.1 | 8.95 |
| 955 | I1L957 | uncharacterized protein | 18 | -1.15 | 48.8 | 6.12 |
| 956 | O03376 | alternative oxidase 3 | 2 | -1.15 | 37.0 | 8.88 |
| 957 | I1JPR3 | serine/threonine protein phosphatase 2a regulatory subunit | 4 | -1.16 | 53.9 | 6.21 |
| 958 | I1K096 | uncharacterized protein | 19 | -1.17 | 61.0 | 8.42 |
| 959 | I1L2U5 | uncharacterized protein | 6 | -1.17 | 103.3 | 8.56 |
| 960 | K7KWZ7 | uncharacterized protein | 7 | -1.18 | 30.8 | 5.87 |
| 961 | A0A0R4J4G9 | uncharacterized protein | 3 | -1.19 | 23.7 | 6.46 |
| 962 | I1LHR1 | uncharacterized protein | 3 | -1.19 | 57.6 | 8.34 |
| 963 | I1JBF6 | ATP-dependent clp protease proteolytic subunit | 5 | -1.19 | 33.8 | 8.67 |
| 964 | I1KF75 | uncharacterized protein | 2 | -1.19 | 33.1 | 8.59 |
| 965 | C6TCH7 | uncharacterized protein | 4 | -1.19 | 29.0 | 9.57 |
| 966 | I1N9W2 | uncharacterized protein | 2 | -1.20 | 70.5 | 6.19 |
| 967 | Q70MR5 | ornithine decarboxylase | 3 | -1.20 | 46.3 | 5.41 |
| 968 | O23959 | Ca2+-binding ef hand protein | 14 | -1.21 | 27.0 | 5.98 |
| 969 | Q09WE7 | UDP-sugar pyrophosphorylase 1 | 15 | -1.22 | 66.1 | 5.70 |
| 970 | Q5DP49 | isoflavone synthase 2 | 16 | -1.22 | 59.3 | 9.17 |
| 971 | A0A0R0K0U9 | uncharacterized protein | 11 | -1.22 | 101.3 | 5.93 |
| 972 | C6SY32 | uncharacterized protein | 2 | -1.23 | 26.1 | 9.06 |
| 973 | A0A0R0GAV8 | uncharacterized protein | 10 | -1.23 | 92.0 | 8.63 |
| 974 | K7M8Y1 | coatomer subunit beta' | 26 | -1.26 | 104.1 | 4.91 |
| 975 | K7M9T4 | uncharacterized protein | 5 | -1.26 | 66.2 | 9.28 |
| 976 | I1LE27 | uncharacterized protein | 5 | -1.26 | 93.3 | 8.66 |
| 977 | C6SY25 | uncharacterized protein | 5 | -1.27 | 13.8 | 9.61 |
| 978 | I1K1N4 | methionine aminopeptidase 2 | 3 | -1.27 | 41.8 | 5.31 |
| 979 | I1LZQ9 | uncharacterized protein | 12 | -1.27 | 82.1 | 5.92 |
| 980 | I1MZN4 | uncharacterized protein | 5 | -1.27 | 29.8 | 10.32 |
| 981 | A0A0R0ELP8 | uncharacterized protein | 6 | -1.27 | 58.7 | 6.56 |
| 982 | I1L3P5 | eukaryotic translation initiation factor 6 | 4 | -1.28 | 26.5 | 4.63 |
| 983 | C6THT7 | uncharacterized protein | 6 | -1.29 | 49.1 | 8.09 |
| 984 | K7MQ23 | uncharacterized protein | 7 | -1.30 | 62.2 | 6.51 |
| 985 | I1J9Q7 | glutamate dehydrogenase | 12 | -1.31 | 44.8 | 5.97 |
| 986 | I1M8P0 | polyadenylate-binding protein | 13 | -1.31 | 71.7 | 7.61 |
| 987 | C6T321 | uncharacterized protein | 3 | -1.31 | 19.7 | 4.74 |
| 988 | I1JND9 | uncharacterized protein | 8 | -1.32 | 82.3 | 6.18 |
| 989 | I1MM35 | uncharacterized protein | 2 | -1.32 | 36.4 | 6.72 |
| 990 | K7LB94 | uncharacterized protein | 3 | -1.32 | 186.0 | 6.25 |
| 991 | I1MDW4 | uncharacterized protein | 4 | -1.34 | 45.1 | 4.51 |
| 992 | K7MHI8 | uncharacterized protein | 3 | -1.34 | 98.7 | 5.54 |
| 993 | C6T4V0 | uncharacterized protein | 2 | -1.35 | 8.0 | 4.25 |
| 994 | I1N5P1 | uncharacterized protein | 5 | -1.36 | 19.9 | 5.45 |
| 995 | A0A0R0GML1 | uncharacterized protein | 2 | -1.36 | 26.0 | 9.21 |
| 996 | C6TET2 | rar1-2 | 3 | -1.37 | 24.6 | 8.32 |
| 997 | C6T2G5 | nascent polypeptide-associated complex subunit beta | 8 | -1.37 | 17.4 | 6.16 |
| 998 | C6SVV1 | uncharacterized protein | 3 | -1.37 | 18.4 | 10.78 |
| 999 | A0A0R4J3N3 | uncharacterized protein | 3 | -1.37 | 18.2 | 6.97 |
| 1000 | C6TAY3 | uncharacterized protein | 8 | -1.38 | 31.8 | 9.42 |
| 1001 | I1NAK9 | uncharacterized protein | 19 | -1.39 | 98.8 | 5.36 |
| 1002 | I1JMI7 | uncharacterized protein | 2 | -1.39 | 69.1 | 7.23 |
| 1003 | C6SVW6 | uncharacterized protein | 3 | -1.39 | 26.6 | 5.84 |
| 1004 | I1JLB9 | uncharacterized protein | 14 | -1.39 | 65.1 | 5.91 |
| 1005 | I1MAK3 | uncharacterized protein | 11 | -1.39 | 233.0 | 6.15 |
| 1006 | I1N0X9 | uncharacterized protein | 3 | -1.40 | 118.3 | 6.10 |
| 1007 | K7KEG4 | uncharacterized protein | 7 | -1.41 | 54.8 | 8.38 |
| 1008 | C6TMK2 | uncharacterized protein | 2 | -1.41 | 31.9 | 9.28 |
| 1009 | C6K8D1 | seed biotinylated protein 68 kda isoform | 15 | -1.42 | 67.9 | 6.18 |
| 1010 | I1L9H8 | uncharacterized protein | 7 | -1.43 | 16.6 | 6.20 |
| 1011 | I1KWS8 | uncharacterized protein | 2 | -1.43 | 36.0 | 5.83 |
| 1012 | I1N505 | uncharacterized protein | 3 | -1.44 | 58.3 | 8.48 |
| 1013 | K7N4I7 | uncharacterized protein | 3 | -1.44 | 22.1 | 9.27 |
| 1014 | A0A0R0G5H5 | uncharacterized protein | 18 | -1.44 | 142.2 | 6.66 |
| 1015 | I1N3M6 | uncharacterized protein | 3 | -1.45 | 31.7 | 6.03 |
| 1016 | K7KXX0 | uncharacterized protein | 6 | -1.46 | 116.4 | 8.74 |
| 1017 | I1M9J3 | uncharacterized protein | 3 | -1.48 | 51.7 | 8.84 |
| 1018 | I1M3W1 | uncharacterized protein | 8 | -1.49 | 22.6 | 9.51 |
| 1019 | I1MRL9 | uncharacterized protein | 6 | -1.50 | 71.1 | 9.30 |
| 1020 | Q9M7N4 | mfp1 attachment factor 1 | 2 | -1.51 | 14.0 | 4.60 |
| 1021 | I1ML97 | alpha-mannosidase | 14 | -1.51 | 117.0 | 5.60 |
| 1022 | K7MIH4 | uncharacterized protein | 7 | -1.51 | 24.2 | 4.54 |
| 1023 | I1KB85 | uncharacterized protein | 4 | -1.51 | 105.1 | 6.14 |
| 1024 | K7K1R2 | uncharacterized protein | 7 | -1.51 | 47.8 | 5.61 |
| 1025 | Q9XER5 | seed maturation protein pm22 | 5 | -1.53 | 16.7 | 5.16 |
| 1026 | K7KR21 | RNA cytidine acetyltransferase | 3 | -1.53 | 116.1 | 7.80 |
| 1027 | I1MB58 | uncharacterized protein | 2 | -1.55 | 29.0 | 11.37 |
| 1028 | Q39839 | nucleoside diphosphate kinase 1 | 7 | -1.55 | 16.4 | 5.91 |
| 1029 | I1JN48 | DNA gyrase subunit b | 6 | -1.55 | 81.1 | 8.58 |
| 1030 | A0A024BSQ0 | acyl-[acyl-carrier-protein] desaturase | 3 | -1.58 | 44.1 | 6.58 |
| 1031 | A0A0R0JL71 | uncharacterized protein | 2 | -1.58 | 43.7 | 5.49 |
| 1032 | C6TJZ2 | er membrane protein complex subunit 3 | 4 | -1.59 | 27.7 | 9.24 |
| 1033 | P04776 | glycinin g1 | 12 | -1.59 | 55.7 | 5.19 |
| 1034 | K7MSV4 | uncharacterized protein | 2 | -1.60 | 84.3 | 6.32 |
| 1035 | C6T8S2 | uncharacterized protein | 5 | -1.60 | 32.5 | 5.15 |
| 1036 | I1M676 | uncharacterized protein | 4 | -1.62 | 19.6 | 4.09 |
| 1037 | C6SZ13 | uncharacterized protein | 3 | -1.62 | 15.8 | 9.35 |
| 1038 | A0A088AWV3 | gamyb-binding protein family protein | 4 | -1.63 | 69.1 | 8.80 |
| 1039 | B4YB07 | phytochrome | 7 | -1.63 | 124.1 | 6.21 |
| 1040 | C6T8H4 | uncharacterized protein | 3 | -1.64 | 35.8 | 7.33 |
| 1041 | I1MX58 | uncharacterized protein | 26 | -1.65 | 60.2 | 6.03 |
| 1042 | I1M138 | uncharacterized protein | 5 | -1.65 | 51.2 | 8.34 |
| 1043 | I1NB78 | uncharacterized protein | 6 | -1.66 | 35.8 | 6.56 |
| 1044 | A0A0R0GAY5 | peroxidase | 2 | -1.68 | 37.8 | 8.47 |
| 1045 | K7MID0 | uncharacterized protein | 13 | -1.71 | 96.9 | 5.94 |
| 1046 | I1K702 | uncharacterized protein | 2 | -1.71 | 36.2 | 8.14 |
| 1047 | I1KR99 | uncharacterized protein | 5 | -1.74 | 37.4 | 8.13 |
| 1048 | I1JUR5 | uncharacterized protein | 2 | -1.75 | 57.1 | 6.26 |
| 1049 | Q9SEK9 | seed maturation protein pm25 | 9 | -1.75 | 25.7 | 4.99 |
| 1050 | K7L4G7 | uncharacterized protein | 8 | -1.75 | 95.3 | 6.12 |
| 1051 | A0A0R4J3S5 | uncharacterized protein | 17 | -1.76 | 66.7 | 7.71 |
| 1052 | C6THL5 | uncharacterized protein | 2 | -1.76 | 27.3 | 5.72 |
| 1053 | I1LMM9 | uncharacterized protein | 2 | -1.77 | 21.7 | 9.09 |
| 1054 | K7MNG0 | uncharacterized protein | 6 | -1.78 | 77.3 | 8.80 |
| 1055 | K7LKM1 | uncharacterized protein | 3 | -1.79 | 99.7 | 7.33 |
| 1056 | I1J7U9 | 3-phosphoshikimate 1-carboxyvinyltransferase | 5 | -1.81 | 55.8 | 8.29 |
| 1057 | K7K4G2 | uncharacterized protein | 8 | -1.81 | 71.7 | 5.38 |
| 1058 | C6THB5 | uncharacterized protein | 3 | -1.82 | 19.8 | 5.70 |
| 1059 | I1MB15 | uncharacterized protein | 18 | -1.82 | 402.1 | 5.16 |
| 1060 | A0A0R4J337 | uncharacterized protein | 3 | -1.83 | 27.6 | 8.63 |
| 1061 | C6TMH1 | uncharacterized protein | 5 | -1.85 | 40.1 | 5.27 |
| 1062 | K7LDT9 | uncharacterized protein | 24 | -1.86 | 103.3 | 6.26 |
| 1063 | K7M2H6 | uncharacterized protein | 4 | -1.87 | 20.1 | 5.41 |
| 1064 | I1LE33 | uncharacterized protein | 8 | -1.89 | 68.2 | 5.94 |
| 1065 | I1KH50 | ADP,ATP carrier protein | 6 | -1.90 | 67.7 | 9.39 |
| 1066 | I1LKD3 | uncharacterized protein | 12 | -1.90 | 52.9 | 5.73 |
| 1067 | K7K8E5 | uncharacterized protein | 13 | -1.93 | 57.7 | 4.69 |
| 1068 | I1L1E2 | uncharacterized protein | 8 | -1.94 | 47.5 | 5.36 |
| 1069 | C6TG28 | uncharacterized protein | 4 | -1.95 | 38.9 | 6.55 |
| 1070 | I1NAH1 | uncharacterized protein | 2 | -1.95 | 28.3 | 6.58 |
| 1071 | K7KBX9 | uncharacterized protein | 4 | -1.97 | 149.8 | 4.26 |
| 1072 | I1LWP5 | uncharacterized protein | 15 | -1.98 | 117.4 | 5.62 |
| 1073 | C6TI81 | ferritin | 6 | -1.98 | 27.9 | 5.71 |
| 1074 | I1KZY8 | uncharacterized protein | 4 | -2.00 | 59.0 | 5.00 |
| 1075 | C6TNZ5 | uncharacterized protein | 2 | -2.02 | 33.0 | 5.67 |
| 1076 | C6TIQ8 | uncharacterized protein | 8 | -2.03 | 47.9 | 7.26 |
| 1077 | A0A0R0J299 | uncharacterized protein | 2 | -2.05 | 53.4 | 9.17 |
| 1078 | I1JV82 | uncharacterized protein | 5 | -2.06 | 24.1 | 8.81 |
| 1079 | I1KJ98 | uncharacterized protein | 2 | -2.08 | 77.6 | 6.63 |
| 1080 | I1K3H5 | uncharacterized protein | 3 | -2.10 | 34.8 | 6.08 |
| 1081 | C6SWJ6 | uncharacterized protein | 2 | -2.12 | 18.2 | 5.80 |
| 1082 | I1KQB7 | uncharacterized protein | 8 | -2.12 | 162.8 | 5.71 |
| 1083 | A0A0R0GIY4 | uncharacterized protein | 12 | -2.14 | 110.1 | 6.26 |
| 1084 | A0A0R0J9B8 | uncharacterized protein | 19 | -2.17 | 124.1 | 6.64 |
| 1085 | C6T871 | uncharacterized protein | 6 | -2.17 | 40.1 | 6.22 |
| 1086 | I1JQQ6 | uncharacterized protein | 4 | -2.18 | 38.8 | 6.14 |
| 1087 | K7LK00 | uncharacterized protein | 3 | -2.21 | 83.4 | 5.68 |
| 1088 | C6T491 | uncharacterized protein | 2 | -2.22 | 11.1 | 5.35 |
| 1089 | C6T1Q7 | uncharacterized protein | 2 | -2.24 | 17.8 | 5.99 |
| 1090 | C6SX10 | mitochondrial fission 1 protein | 4 | -2.26 | 18.7 | 6.96 |
| 1091 | I1JXD8 | protein-l-isoaspartate o-methyltransferase | 3 | -2.26 | 35.8 | 9.02 |
| 1092 | I1J8V4 | uncharacterized protein | 3 | -2.28 | 57.4 | 8.91 |
| 1093 | K7KTE8 | uncharacterized protein | 3 | -2.29 | 99.3 | 6.79 |
| 1094 | C6TL41 | eukaryotic translation initiation factor 3 subunit i | 12 | -2.31 | 35.9 | 6.26 |
| 1095 | A0A0R0HSY5 | uncharacterized protein | 5 | -2.35 | 132.1 | 6.42 |
| 1096 | I1LG49 | peptidylprolyl isomerase | 2 | -2.39 | 55.4 | 5.45 |
| 1097 | I1MDY6 | uncharacterized protein | 2 | -2.43 | 60.6 | 9.53 |
| 1098 | I1JHQ8 | auxin response factor | 6 | -2.46 | 94.1 | 6.06 |
| 1099 | K7LD07 | uncharacterized protein | 7 | -2.48 | 213.1 | 8.56 |
| 1100 | C6T142 | small ubiquitin-related modifier | 4 | -2.48 | 11.2 | 4.95 |
| 1101 | I1M928 | uncharacterized protein | 2 | -2.52 | 51.0 | 8.91 |
| 1102 | Q42447 | maturation protein | 6 | -2.57 | 25.6 | 6.02 |
| 1103 | I1LD09 | uncharacterized protein | 10 | -2.58 | 58.1 | 5.88 |
| 1104 | I1NBR9 | uncharacterized protein | 3 | -2.59 | 19.4 | 6.82 |
| 1105 | I1KJ56 | uncharacterized protein | 12 | -2.63 | 61.4 | 9.01 |
| 1106 | K7KTR9 | oleosin | 2 | -2.63 | 22.6 | 8.81 |
| 1107 | K7KC67 | uncharacterized protein | 2 | -2.68 | 49.2 | 9.01 |
| 1108 | C6SWW4 | uncharacterized protein | 5 | -2.68 | 22.4 | 4.75 |
| 1109 | I1K5Z3 | uncharacterized protein | 5 | -2.68 | 34.9 | 8.86 |
| 1110 | I1LVS8 | xyloglucan endotransglucosylase/hydrolase | 5 | -2.70 | 33.2 | 7.75 |
| 1111 | I1L6S3 | uncharacterized protein | 2 | -2.71 | 57.6 | 6.02 |
| 1112 | K7LEQ5 | uncharacterized protein | 10 | -2.75 | 26.6 | 6.29 |
| 1113 | Q9SDY6 | chitinase class i | 2 | -2.79 | 34.3 | 6.57 |
| 1114 | A0A0R0J452 | uncharacterized protein | 2 | -2.82 | 25.2 | 5.69 |
| 1115 | F7J077 | beta-conglycinin beta subunit | 11 | -2.82 | 50.4 | 5.67 |
| 1116 | I1KW97 | uncharacterized protein | 2 | -2.83 | 43.0 | 8.38 |
| 1117 | K7MNQ7 | uncharacterized protein | 4 | -2.84 | 30.4 | 8.74 |
| 1118 | C6TMH8 | ribosomal protein l19 | 2 | -2.89 | 24.7 | 11.45 |
| 1119 | I1LVC1 | uncharacterized protein | 5 | -2.90 | 14.9 | 5.54 |
| 1120 | A0A0R0FK88 | protein yipf | 2 | -2.95 | 30.5 | 9.51 |
| 1121 | I1L860 | uncharacterized protein | 12 | -3.01 | 58.0 | 5.92 |
| 1122 | I1KXB7 | uncharacterized protein | 2 | -3.02 | 64.0 | 8.35 |
| 1123 | I1NAI0 | uncharacterized protein | 2 | -3.04 | 16.2 | 4.89 |
| 1124 | Q01527 | maturation protein | 4 | -3.05 | 15.6 | 9.66 |
| 1125 | K7LEW3 | serine/threonine-protein phosphatase | 2 | -3.06 | 61.5 | 6.75 |
| 1126 | O22378 | metallothionein-ii protein | 2 | -3.07 | 8.3 | 7.36 |
| 1127 | I1J4K3 | uncharacterized protein | 2 | -3.10 | 78.0 | 5.02 |
| 1128 | I1L295 | uncharacterized protein | 2 | -3.13 | 60.1 | 6.15 |
| 1129 | C6SV67 | uncharacterized protein | 4 | -3.20 | 16.4 | 5.85 |
| 1130 | I1KVG8 | uncharacterized protein | 16 | -3.22 | 80.7 | 5.40 |
| 1131 | I1M8S3 | uncharacterized protein | 13 | -3.27 | 82.1 | 6.67 |
| 1132 | A0A0R0J1S6 | uncharacterized protein | 3 | -3.27 | 83.7 | 5.08 |
| 1133 | K7MVZ6 | glucose-6-phosphate isomerase | 12 | -3.42 | 62.7 | 5.47 |
| 1134 | I1N8U7 | uncharacterized protein | 5 | -3.45 | 40.4 | 6.60 |
| 1135 | I1LNN8 | uncharacterized protein | 3 | -3.66 | 39.9 | 9.35 |
| 1136 | A0A0R0KHW0 | 40s ribosomal protein s26 | 4 | -3.76 | 14.8 | 10.92 |
| 1137 | K7LSG9 | uncharacterized protein | 2 | -3.77 | 10.8 | 5.29 |
| 1138 | C6T0U3 | uncharacterized protein | 5 | -3.79 | 13.8 | 9.61 |
| 1139 | I1L7B5 | uncharacterized protein | 9 | -3.85 | 67.9 | 8.57 |
| 1140 | I1M786 | uncharacterized protein | 14 | -4.01 | 24.2 | 4.53 |
| 1141 | A0A0R0KK84 | uncharacterized protein | 10 | -4.02 | 54.6 | 5.23 |
| 1142 | I1JIH0 | uncharacterized protein | 4 | -4.08 | 30.0 | 6.94 |
| 1143 | I1L8A9 | uncharacterized protein | 2 | -4.23 | 30.9 | 10.61 |
| 1144 | I1JHW4 | uncharacterized protein | 2 | -4.25 | 18.8 | 10.42 |
| 1145 | I1KNP5 | endoglucanase | 4 | -4.31 | 68.7 | 8.87 |
| 1146 | I1KQV7 | uncharacterized protein | 22 | -4.37 | 72.5 | 5.82 |
| 1147 | I1LD16 | chorismate synthase | 3 | -4.87 | 47.4 | 7.13 |
| 1148 | C6SVG3 | uncharacterized protein | 6 | -4.88 | 17.5 | 4.60 |
| 1149 | Q93YH8 | hmg i/y like protein | 2 | -5.94 | 44.2 | 10.91 |
| 1150 | I1JYP1 | uncharacterized protein | 2 | -6.33 | 25.7 | 5.77 |
| 1151 | Q0WX04 | polygalacturonase inhibiting protein | 12 | -6.57 | 37.5 | 9.10 |

a “accession” is determined according to UniprotKB *Glycine* max (Soybean) protein database. b “M.P.” means the number of matched peptides. c “fold change” indicates log2 fold change of identified proteins from soybean treated with millimeter waves radiation compared to control at the starting point.

**Table S2**. List of changed proteins between irradiated and unirradiated soybean seedlings without flooding stress.

| number | accession a | description | M.P.b | fold change c | MW in kDa | | pI | |
| --- | --- | --- | --- | --- | --- | --- | --- | --- |
| 1 | A0A0R0J569 | uncharacterized protein | 3 | 5.92 | 46.0 | | 7.67 | |
| 2 | I1N0I7 | uncharacterized protein | 4 | 5.58 | 28.1 | | 9.08 | |
| 3 | K7N2X4 | uncharacterized protein | 15 | 4.40 | 53.4 | | 6.62 | |
| 4 | I1NE45 | lipoxygenase | 2 | 4.16 | 102.8 | 5.93 | |
| 5 | I1MXJ1 | uncharacterized protein | 13 | 3.98 | 82.2 | | 6.21 | |
| 6 | I1K4R1 | uncharacterized protein | 9 | 3.54 | 25.8 | | 10.18 | |
| 7 | A0A0R0JBP2 | uncharacterized protein | 8 | 3.14 | 24.1 | | 5.46 | |
| 8 | I1KRI3 | uncharacterized protein | 5 | 3.13 | 43.1 | | 6.42 | |
| 9 | I1KFZ1 | uncharacterized protein | 2 | 3.10 | 58.4 | | 10.02 | |
| 10 | I1L3K3 | uncharacterized protein | 5 | 3.09 | 27.4 | | 9.13 | |
| 11 | K7KPU4 | uncharacterized protein | 9 | 2.94 | 68.8 | | 6.06 | |
| 12 | I1M8S3 | uncharacterized protein | 13 | 2.84 | 82.1 | | 6.67 | |
| 13 | K7LSC0 | uncharacterized protein | 3 | 2.75 | 41.8 | | 5.14 | |
| 14 | O22378 | metallothionein-ii protein | 2 | 2.56 | 8.3 | | 7.36 | |
| 15 | A0A0R0J1S6 | uncharacterized protein | 3 | 2.56 | 83.7 | | 5.08 | |
| 16 | I1LFF1 | chalcone-flavonone isomerase family protein | 8 | 2.49 | 25.0 | | 5.17 | |
| 17 | B0M194 | peroxisomal 3-hydroxyacyl-coa dehydrogenase-like protein | 3 | 2.48 | 35.0 | | 9.48 | |
| 18 | A0A0R0I1Y2 | uncharacterized protein | 5 | 2.44 | 42.6 | | 6.11 | |
| 19 | A0A0R0EIR6 | glycosyltransferase | 8 | 2.39 | 54.4 | | 5.60 | |
| 20 | I1MG74 | uncharacterized protein | 5 | 2.38 | 104.9 | | 6.65 | |
| 21 | A0A0R0HTH9 | uncharacterized protein | 6 | 2.36 | 94.7 | | 4.52 | |
| 22 | A5Z2K3 | photosystem i subunit psad | 2 | 2.33 | 23.0 | | 9.63 | |
| 23 | I1N3E1 | uncharacterized protein | 5 | 2.31 | 46.9 | | 8.35 | |
| 24 | I1MVH5 | uncharacterized protein | 4 | 2.29 | 121.8 | | 5.41 | |
| 25 | I1KYC4 | uncharacterized protein | 6 | 2.24 | 51.9 | | 8.43 | |
| 26 | A0A0R0INP8 | peroxidase | 4 | 2.20 | 34.5 | | 9.05 | |
| 27 | C6SZQ8 | uncharacterized protein | 2 | 2.03 | 17.3 | | 6.21 | |
| 28 | C6TGB2 | uncharacterized protein | 3 | 1.96 | 36.2 | | 9.57 | |
| 29 | I1MNR2 | xyloglucan endotransglucosylase/hydrolase | 8 | 1.96 | 25.4 | | 6.31 | |
| 30 | I1NCU3 | uncharacterized protein | 3 | 1.96 | 51.6 | | 8.52 | |
| 31 | C6TKJ2 | xyloglucan endotransglucosylase/hydrolase | 3 | 1.95 | 35.6 | | 8.53 | |
| 32 | K7M6X1 | protein disulfide-isomerase | 5 | 1.90 | 55.8 | | 5.13 | |
| 33 | A0A0R4J4X3 | cysteine proteinase inhibitor | 3 | 1.88 | 10.7 | | 5.83 | |
| 34 | C6SVL0 | uncharacterized protein | 6 | 1.78 | 21.8 | | 4.91 | |
| 35 | K7M352 | uncharacterized protein | 3 | 1.76 | 60.4 | | 5.50 | |
| 36 | I1KP27 | signal recognition particle subunit srp68 | 8 | 1.76 | 68.2 | | 9.04 | |
| 37 | C6TGT5 | uncharacterized protein | 2 | 1.73 | 37.7 | | 6.71 | |
| 38 | I1K4K8 | acyl-coenzyme a oxidase | 3 | 1.67 | 75.6 | | 9.04 | |
| 39 | I1JY96 | uncharacterized protein | 3 | 1.67 | 113.6 | | 6.54 | |
| 40 | I1M928 | uncharacterized protein | 2 | 1.61 | 51.0 | | 8.91 | |
| 41 | Q2LAL4 | cytochrome p450 monooxygenase cyp83e8 | 7 | 1.60 | 57.4 | | 8.20 | |
| 42 | A0A0R0KUR1 | phenylalanine ammonia-lyase | 8 | 1.44 | 77.9 | | 6.00 | |
| 43 | I1M6K2 | uncharacterized protein | 3 | 1.44 | 46.1 | | 4.57 | |
| 44 | A0A0R0IYE6 | lipoxygenase | 29 | 1.43 | 96.3 | | 6.22 | |
| 45 | C6TDJ1 | uncharacterized protein | 4 | 1.43 | 52.5 | | 5.40 | |
| 46 | I1K2X6 | uncharacterized protein | 9 | 1.42 | 59.5 | | 9.09 | |
| 47 | I1KHB7 | uncharacterized protein | 3 | 1.41 | 23.8 | | 6.24 | |
| 48 | F8WRI3 | gamma-tocopherol methyltransferase | 4 | 1.37 | 33.3 | | 6.33 | |
| 49 | I1LDZ8 | uncharacterized protein | 4 | 1.35 | 37.7 | | 5.89 | |
| 50 | C6SYL5 | uncharacterized protein | 2 | 1.32 | 22.2 | | 5.80 | |
| 51 | A0A0R0GFB2 | uncharacterized protein | 23 | 1.29 | 57.5 | | 8.99 | |
| 52 | A0A0R0HPS3 | uncharacterized protein | 8 | 1.27 | 95.6 | | 5.61 | |
| 53 | I1KBS7 | uncharacterized protein | 14 | 1.26 | 62.5 | | 4.81 | |
| 54 | C6TDZ1 | uncharacterized protein | 5 | 1.25 | 30.7 | | 6.19 | |
| 55 | K7LZ50 | uncharacterized protein | 16 | 1.25 | 115.6 | | 5.67 | |
| 56 | I1J5S9 | uncharacterized protein | 3 | 1.24 | 17.4 | | 4.75 | |
| 57 | A0A0R0K666 | uncharacterized protein | 9 | 1.21 | 47.0 | | 8.34 | |
| 58 | I1KSW9 | alpha-galactosidase | 6 | 1.21 | 71.2 | | 7.29 | |
| 59 | I1N610 | uncharacterized protein | 4 | 1.18 | 35.5 | | 5.61 | |
| 60 | I1JPR3 | serine/threonine protein phosphatase 2a regulatory subunit | 4 | 1.17 | 53.9 | | 6.21 | |
| 61 | Q2PMT9 | photosystem ii cp43 reaction center protein | 5 | 1.15 | 51.9 | | 6.34 | |
| 62 | K7M2D8 | uncharacterized protein | 8 | 1.15 | 140.9 | | 5.74 | |
| 63 | I1L3K1 | uncharacterized protein | 4 | 1.15 | 158.7 | | 5.74 | |
| 64 | A0A0R0K0E5 | uncharacterized protein | 4 | 1.13 | 59.2 | | 7.78 | |
| 65 | I1NHU7 | uncharacterized protein | 3 | 1.13 | 39.0 | | 5.86 | |
| 66 | C6T0C7 | uncharacterized protein | 7 | 1.11 | 23.4 | | 4.73 | |
| 67 | A0A0R4J339 | uncharacterized protein | 5 | 1.11 | 82.3 | | 8.57 | |
| 68 | A0A0R0JPL6 | uncharacterized protein | 9 | 1.10 | 50.0 | | 8.57 | |
| 69 | I1KFE9 | uncharacterized protein | 10 | 1.10 | 60.1 | | 6.93 | |
| 70 | I1ML46 | uncharacterized protein | 13 | 1.08 | 56.5 | | 5.00 | |
| 71 | K7MNL1 | uncharacterized protein | 2 | 1.05 | 29.9 | | 5.78 | |
| 72 | C6F122 | beta-galactosidase | 4 | 1.02 | 79.8 | | 8.12 | |
| 73 | I1KRB4 | uncharacterized protein | 3 | 1.02 | 60.4 | | 8.57 | |
| 74 | C6TJ98 | uncharacterized protein | 7 | 1.01 | 27.6 | | 4.76 | |
| 75 | K7M9Q3 | chlorophyll a-b binding protein | 2 | 0.99 | 27.6 | | 6.15 | |
| 76 | C6SVR0 | plastocyanin | 3 | 0.99 | 16.8 | | 5.04 | |
| 77 | I1L8V8 | uncharacterized protein | 5 | 0.99 | 17.7 | | 8.83 | |
| 78 | I1KZI4 | uncharacterized protein | 8 | 0.98 | 50.3 | | 5.69 | |
| 79 | K7K6S5 | uncharacterized protein | 5 | 0.96 | 29.3 | | 5.39 | |
| 80 | C6TGA9 | uncharacterized protein | 4 | 0.95 | 38.9 | | 5.78 | |
| 81 | A0A0R4J389 | uncharacterized protein | 3 | 0.94 | 24.3 | | 9.73 | |
| 82 | P15490 | stem 28 kda glycoprotein | 13 | 0.92 | 29.0 | | 8.38 | |
| 83 | A0A0R4J645 | uncharacterized protein | 2 | 0.92 | 41.6 | | 5.26 | |
| 84 | C6SZV4 | plastocyanin | 3 | 0.92 | 16.5 | | 4.82 | |
| 85 | A0A0R0I8Z5 | chlorophyll a-b binding protein | 5 | 0.91 | 31.0 | | 5.72 | |
| 86 | I1KCP1 | plasma membrane atpase | 19 | 0.91 | 105.2 | | 6.39 | |
| 87 | I1M026 | uncharacterized protein | 4 | 0.88 | 58.6 | | 7.15 | |
| 88 | C6TMN2 | uncharacterized protein | 4 | 0.87 | 26.9 | | 9.43 | |
| 89 | C6TD35 | uncharacterized protein | 2 | 0.86 | 37.0 | | 5.20 | |
| 90 | K7MMN7 | uncharacterized protein | 9 | 0.86 | 66.1 | | 8.26 | |
| 91 | I1KUF1 | uncharacterized protein | 8 | 0.85 | 83.8 | | 5.67 | |
| 92 | P49161 | cytochrome f | 4 | 0.85 | 35.3 | | 8.38 | |
| 93 | K7N4X8 | uncharacterized protein | 2 | 0.83 | 21.5 | | 6.44 | |
| 94 | A0A0R4J610 | uncharacterized protein | 5 | 0.83 | 57.1 | | 6.96 | |
| 95 | A0A0R0KHF0 | uncharacterized protein | 3 | 0.81 | 19.2 | | 5.14 | |
| 96 | Q09WE7 | udp-sugar pyrophosphorylase 1 | 15 | 0.81 | 66.1 | | 5.70 | |
| 97 | I1JNM9 | uncharacterized protein | 2 | 0.79 | 27.7 | | 5.88 | |
| 98 | I1JEW0 | uncharacterized protein | 2 | 0.78 | 33.7 | | 5.79 | |
| 99 | A0A0R0KAT4 | beta-galactosidase | 20 | 0.78 | 93.9 | | 7.96 | |
| 100 | K7LC03 | glucose-6-phosphate 1-epimerase | 11 | 0.77 | 36.1 | | 5.75 | |
| 101 | A0A0R0I1Y7 | peroxidase | 3 | 0.77 | 37.9 | | 8.08 | |
| 102 | C6T1C8 | uncharacterized protein | 3 | 0.77 | 22.8 | | 9.32 | |
| 103 | P10743 | stem 31 kda glycoprotein | 21 | 0.77 | 29.3 | | 5.84 | |
| 104 | I1MRK1 | uncharacterized protein | 18 | 0.77 | 78.7 | | 9.14 | |
| 105 | I1MRT3 | uncharacterized protein | 13 | 0.75 | 54.2 | | 5.88 | |
| 106 | I1JQ51 | uncharacterized protein | 4 | 0.75 | 39.7 | | 5.29 | |
| 107 | M1FPG4 | nadh dehydrogenase subunit 2 | 3 | 0.74 | 53.3 | | 8.89 | |
| 108 | A0A0R0HAR3 | uncharacterized protein | 5 | 0.74 | 64.3 | | 5.92 | |
| 109 | Q2PMQ9 | photosystem ii cp47 reaction center protein | 6 | 0.74 | 56.0 | | 6.20 | |
| 110 | A0A0R0JYS9 | peroxidase | 15 | 0.74 | 35.5 | | 8.92 | |
| 111 | A0A0R0J8D5 | uncharacterized protein | 5 | 0.73 | 24.9 | | 9.80 | |
| 112 | I1L5K6 | carboxypeptidase | 7 | 0.73 | 55.6 | | 6.39 | |
| 113 | I1M2X3 | uncharacterized protein | 9 | 0.72 | 110.0 | | 5.42 | |
| 114 | I1JS35 | uncharacterized protein | 8 | 0.72 | 65.5 | | 6.60 | |
| 115 | A0A0R0FVK4 | carboxypeptidase | 3 | 0.72 | 55.7 | | 5.53 | |
| 116 | A0A0R0IM08 | uncharacterized protein | 10 | 0.71 | 30.9 | | 8.61 | |
| 117 | A0A0R0GYY6 | uncharacterized protein | 5 | 0.71 | 30.5 | | 5.14 | |
| 118 | I1JE93 | uncharacterized protein | 2 | 0.71 | 25.9 | | 8.26 | |
| 119 | Q2PMU2 | photosystem i p700 chlorophyll a apoprotein a2 | 8 | 0.70 | 82.4 | | 6.80 | |
| 120 | I1MUQ0 | uncharacterized protein | 2 | 0.69 | 27.4 | | 5.98 | |
| 121 | I1LU69 | uncharacterized protein | 6 | 0.69 | 34.5 | | 6.99 | |
| 122 | I1JD78 | uncharacterized protein | 12 | 0.69 | 54.0 | | 5.66 | |
| 123 | C6SYP7 | dirigent protein | 2 | 0.68 | 24.2 | | 7.09 | |
| 124 | A0A0R0H3D0 | uncharacterized protein | 7 | 0.68 | 28.4 | | 7.17 | |
| 125 | C6TKW2 | chlorophyll a-b binding protein | 5 | 0.67 | 28.8 | | 5.11 | |
| 126 | Q03773 | glucan endo-1,3-beta-glucosidase | 18 | 0.67 | 38.1 | | 8.47 | |
| 127 | I1L3T1 | udp-glycosyltransferase 708d1 | 8 | 0.67 | 52.5 | | 5.42 | |
| 128 | K7MGN4 | uncharacterized protein | 9 | 0.66 | 132.7 | | 6.08 | |
| 129 | A0A0R0LA35 | uncharacterized protein | 10 | 0.65 | 35.2 | | 6.07 | |
| 130 | I1MAR9 | uncharacterized protein | 24 | 0.65 | 85.0 | | 7.14 | |
| 131 | I1LWC8 | uncharacterized protein | 3 | 0.65 | 13.1 | | 5.13 | |
| 132 | I1MBZ5 | uncharacterized protein | 10 | 0.64 | 60.8 | | 9.08 | |
| 133 | I1J937 | uncharacterized protein | 16 | 0.64 | 60.3 | | 8.82 | |
| 134 | C6SWD5 | uncharacterized protein | 2 | 0.63 | 19.7 | | 8.61 | |
| 135 | I1K7W5 | uncharacterized protein | 9 | 0.63 | 42.8 | | 5.16 | |
| 136 | C6TNL4 | uncharacterized protein | 5 | 0.62 | 37.3 | | 8.24 | |
| 137 | I1KEL7 | peroxidase | 9 | 0.60 | 34.0 | | 8.66 | |
| 138 | I1LLG3 | uncharacterized protein | 9 | 0.60 | 46.8 | | 9.20 | |
| 139 | I1N641 | uncharacterized protein | 8 | 0.59 | 136.1 | | 8.09 | |
| 140 | I1L1Q3 | uncharacterized protein | 3 | 0.59 | 45.1 | | 6.56 | |
| 141 | A0A0R0FW44 | uncharacterized protein | 11 | 0.59 | 42.8 | | 6.38 | |
| 142 | I1L5L1 | ribosomal protein | 4 | 0.58 | 37.9 | | 9.25 | |
| 143 | I1LBV4 | ubiquitin carboxyl-terminal hydrolase | 5 | 0.58 | 38.3 | | 6.10 | |
| 144 | I1LCP7 | uncharacterized protein | 2 | 0.58 | 54.8 | | 6.73 | |
| 145 | I1NGN1 | uncharacterized protein | 9 | 0.58 | 37.3 | | 8.63 | |
| 146 | I1N5R1 | uncharacterized protein | 8 | 0.57 | 42.6 | | 8.68 | |
| 147 | I1M4L7 | beta-galactosidase | 6 | 0.57 | 91.6 | | 6.18 | |
| 148 | C6TL93 | uncharacterized protein | 7 | 0.57 | 41.9 | | 5.76 | |
| 149 | K7L6U4 | uncharacterized protein | 13 | 0.56 | 42.0 | | 9.15 | |
| 150 | I1K5U0 | uncharacterized protein | 14 | 0.55 | 58.5 | | 6.00 | |
| 151 | A0A0R0JPA1 | beta-galactosidase | 16 | 0.54 | 94.0 | | 7.21 | |
| 152 | I1K711 | uncharacterized protein | 6 | 0.54 | 40.0 | | 4.82 | |
| 153 | I1LY51 | uncharacterized protein | 15 | 0.53 | 44.1 | | 8.22 | |
| 154 | P93159 | beta-glucan-elicitor receptor | 17 | 0.53 | 74.6 | | 5.29 | |
| 155 | I1LU45 | uncharacterized protein | 9 | 0.52 | 59.7 | | 6.68 | |
| 156 | C6TJ77 | uncharacterized protein | 6 | 0.52 | 48.9 | | 5.28 | |
| 157 | C6SVN5 | uncharacterized protein | 2 | 0.52 | 19.3 | | 8.89 | |
| 158 | I1M4G0 | carboxypeptidase | 16 | 0.52 | 51.0 | | 4.82 | |
| 159 | A0A0R0H1W3 | uncharacterized protein | 11 | 0.51 | 96.9 | | 5.92 | |
| 160 | I1M292 | uncharacterized protein | 4 | 0.49 | 25.7 | | 8.55 | |
| 161 | A0A0R4J532 | uncharacterized protein | 6 | 0.48 | 21.7 | | 6.43 | |
| 162 | I1JQY5 | pectin acetylesterase | 6 | 0.48 | 45.0 | | 5.76 | |
| 163 | C6T4X8 | uncharacterized protein | 3 | 0.48 | 17.1 | | 9.24 | |
| 164 | Q06197 | isocitrate dehydrogenase | 20 | 0.48 | 46.0 | | 5.87 | |
| 165 | C6TIQ5 | uncharacterized protein | 18 | 0.47 | 37.9 | | 5.93 | |
| 166 | I1MVG7 | uncharacterized protein | 5 | 0.47 | 76.9 | | 4.26 | |
| 167 | I1LV30 | uncharacterized protein | 5 | 0.47 | 37.5 | | 5.22 | |
| 168 | I1KR53 | uncharacterized protein | 18 | 0.46 | 82.5 | | 7.83 | |
| 169 | I1LJP3 | uncharacterized protein | 14 | 0.45 | 35.4 | | 4.96 | |
| 170 | I1JF82 | methylcrotonoyl-coa carboxylase subunit alpha | 14 | 0.44 | 81.1 | | 6.88 | |
| 171 | C6TD38 | uncharacterized protein | 5 | 0.44 | 22.0 | | 6.43 | |
| 172 | I1JHQ6 | uncharacterized protein | 10 | 0.44 | 60.9 | | 8.78 | |
| 173 | C6TFM6 | peptidyl-prolyl cis-trans isomerase | 2 | 0.44 | 26.0 | | 6.16 | |
| 174 | I1MK13 | uncharacterized protein | 13 | 0.43 | 61.1 | | 6.91 | |
| 175 | C6THI3 | uncharacterized protein | 11 | 0.42 | 39.1 | | 6.37 | |
| 176 | A0A0R4J5Q8 | uncharacterized protein | 21 | 0.41 | 79.9 | | 6.35 | |
| 177 | K7LHD8 | uncharacterized protein | 3 | 0.41 | 21.9 | | 9.51 | |
| 178 | O65016 | actin 4 | 19 | 0.41 | 41.4 | | 5.60 | |
| 179 | Q9MAX8 | coatomer subunit epsilon | 11 | 0.40 | 32.3 | | 5.31 | |
| 180 | C6TGV2 | isocitrate dehydrogenase | 11 | 0.39 | 46.7 | | 8.67 | |
| 181 | K7LH21 | uncharacterized protein | 12 | 0.39 | 57.8 | | 4.65 | |
| 182 | I1LIN2 | uncharacterized protein | 48 | 0.39 | 153.2 | | 4.68 | |
| 183 | I1JMY1 | uncharacterized protein | 13 | 0.39 | 73.8 | | 5.68 | |
| 184 | A0A0R0IPU2 | uncharacterized protein | 8 | 0.39 | 59.4 | | 7.00 | |
| 185 | A0A0R0JD84 | glycine cleavage system h protein | 2 | 0.39 | 20.0 | | 4.84 | |
| 186 | C6TM13 | uncharacterized protein | 3 | 0.38 | 30.0 | | 5.64 | |
| 187 | I1NBP7 | uncharacterized protein | 12 | 0.38 | 39.5 | | 8.06 | |
| 188 | Q2PMU3 | photosystem i p700 chlorophyll a apoprotein a1 | 2 | 0.38 | 83.4 | | 6.74 | |
| 189 | I1KEW2 | uncharacterized protein | 5 | 0.38 | 40.6 | | 5.66 | |
| 190 | A0A0R0F2G6 | uncharacterized protein | 13 | 0.37 | 52.5 | | 8.04 | |
| 191 | I1KH38 | uncharacterized protein | 4 | 0.37 | 26.0 | | 7.05 | |
| 192 | I1N4F6 | uncharacterized protein | 7 | 0.37 | 57.9 | | 6.47 | |
| 193 | I1KE09 | uncharacterized protein | 7 | 0.37 | 17.3 | | 4.79 | |
| 194 | I1JEV7 | uncharacterized protein | 4 | 0.35 | 29.8 | | 4.98 | |
| 195 | I1M6Y5 | uncharacterized protein | 6 | 0.35 | 91.4 | | 6.83 | |
| 196 | I1NF54 | uncharacterized protein | 11 | 0.34 | 63.7 | | 7.61 | |
| 197 | C6TF34 | uncharacterized protein | 6 | 0.34 | 32.4 | | 6.04 | |
| 198 | I1N8S9 | uncharacterized protein | 25 | 0.32 | 172.1 | | 6.69 | |
| 199 | Q6YGT9 | purple acid phosphatase | 16 | 0.31 | 59.5 | | 8.10 | |
| 200 | I1LEI1 | uncharacterized protein | 8 | 0.31 | 98.1 | | 6.29 | |
| 201 | I1L4J2 | uncharacterized protein | 24 | 0.30 | 78.1 | | 9.28 | |
| 202 | I1MUX7 | uncharacterized protein | 10 | 0.29 | 66.7 | | 9.22 | |
| 203 | I1LHP2 | uncharacterized protein | 10 | 0.27 | 43.2 | | 5.51 | |
| 204 | I1NF29 | uncharacterized protein | 25 | 0.27 | 86.4 | | 5.80 | |
| 205 | I1KXM1 | pyruvate dehydrogenase e1 component subunit alpha | 9 | 0.27 | 43.6 | | 7.12 | |
| 206 | C6TE22 | uncharacterized protein | 3 | 0.27 | 27.0 | | 5.41 | |
| 207 | K7L350 | carboxypeptidase | 12 | 0.27 | 50.7 | | 8.13 | |
| 208 | Q42795 | beta-amylase | 26 | 0.26 | 56.0 | | 5.33 | |
| 209 | I1JN59 | uncharacterized protein | 24 | 0.26 | 177.4 | | 6.44 | |
| 210 | B1ACD3 | putative kunitz trypsin protease inhibitor | 7 | 0.24 | 23.6 | | 4.68 | |
| 211 | I1N8P5 | tubulin beta chain | 28 | 0.24 | 50.5 | | 4.70 | |
| 212 | K7MV52 | uncharacterized protein | 3 | 0.24 | 70.9 | | 9.08 | |
| 213 | I1KPF0 | uncharacterized protein | 9 | 0.24 | 53.3 | | 8.69 | |
| 214 | I1LYN0 | succinate--coa ligase | 13 | 0.24 | 45.3 | | 5.83 | |
| 215 | I1KIP9 | uncharacterized protein | 29 | 0.23 | 78.1 | | 9.31 | |
| 216 | I1MGL5 | uncharacterized protein | 5 | 0.22 | 60.5 | | 5.27 | |
| 217 | I1KY45 | glutamyl-trna(gln) amidotransferase subunit a | 5 | 0.21 | 57.6 | | 6.28 | |
| 218 | C6T7R6 | uncharacterized protein | 14 | 0.21 | 47.5 | | 5.50 | |
| 219 | B0M1A5 | betaine aldehyde dehydrogenase | 28 | 0.21 | 54.6 | | 5.35 | |
| 220 | I1JMI9 | malic enzyme | 15 | 0.20 | 68.9 | | 5.71 | |
| 221 | I1JIL1 | uncharacterized protein | 2 | 0.20 | 19.1 | | 5.91 | |
| 222 | C6TAX0 | uncharacterized protein | 10 | -0.10 | 39.8 | | 5.56 | |
| 223 | I1J5B4 | uncharacterized protein | 27 | -0.11 | 82.3 | | 7.11 | |
| 224 | I1K670 | uncharacterized protein | 28 | -0.12 | 73.6 | | 5.02 | |
| 225 | I1LWI3 | uncharacterized protein | 7 | -0.16 | 31.5 | | 5.11 | |
| 226 | I1NJ85 | uncharacterized protein | 27 | -0.16 | 67.4 | | 6.51 | |
| 227 | I1LKU1 | uncharacterized protein | 23 | -0.16 | 57.0 | | 5.55 | |
| 228 | P29756 | catalase-1/2 | 18 | -0.17 | 56.8 | | 6.77 | |
| 229 | I1KXG9 | uncharacterized protein | 22 | -0.19 | 58.4 | | 7.19 | |
| 230 | I1LMA5 | uncharacterized protein | 23 | -0.19 | 81.3 | | 6.29 | |
| 231 | A0A0R4J410 | obg-like atpase 1 | 17 | -0.21 | 44.4 | | 6.36 | |
| 232 | C6SVR8 | uncharacterized protein | 5 | -0.21 | 16.0 | | 10.24 | |
| 233 | I1M3M0 | 14-3-3-like protein d | 14 | -0.21 | 29.4 | | 4.80 | |
| 234 | C6TH59 | proteasome subunit alpha type | 13 | -0.21 | 25.6 | | 5.48 | |
| 235 | A0A0R0ID57 | 4-hydroxy-4-methyl-2-oxoglutarate aldolase | 4 | -0.21 | 17.8 | | 5.38 | |
| 236 | C6TAZ2 | uncharacterized protein | 11 | -0.21 | 23.2 | | 6.96 | |
| 237 | I1KMX5 | uncharacterized protein | 21 | -0.22 | 86.0 | | 9.01 | |
| 238 | A0A0R4J2M8 | proteasome subunit alpha type | 8 | -0.22 | 27.1 | | 6.98 | |
| 239 | I1NIY6 | uncharacterized protein | 18 | -0.23 | 51.4 | | 6.04 | |
| 240 | I1M7C7 | uncharacterized protein | 5 | -0.23 | 13.6 | | 9.48 | |
| 241 | I1J4I1 | phosphotransferase | 9 | -0.24 | 53.6 | | 5.96 | |
| 242 | I1NEA8 | uncharacterized protein | 9 | -0.24 | 79.3 | | 5.92 | |
| 243 | C6TLT3 | 40s ribosomal protein s3a | 12 | -0.25 | 29.7 | | 9.81 | |
| 244 | C6TNC2 | uncharacterized protein | 8 | -0.25 | 41.9 | | 5.52 | |
| 245 | I1JJB5 | uncharacterized protein | 7 | -0.25 | 50.5 | | 9.25 | |
| 246 | I1JZT5 | plasma membrane atpase | 25 | -0.26 | 105.0 | | 6.28 | |
| 247 | I1JQD9 | eukaryotic translation initiation factor 3 subunit c | 27 | -0.26 | 104.6 | | 5.44 | |
| 248 | I1MXM6 | polyadenylate-binding protein | 13 | -0.26 | 71.9 | | 6.70 | |
| 249 | K7M387 | uncharacterized protein | 25 | -0.26 | 127.4 | | 5.18 | |
| 250 | C6TMT8 | uncharacterized protein | 4 | -0.27 | 42.9 | | 5.12 | |
| 251 | I1LAZ3 | uncharacterized protein | 12 | -0.27 | 56.9 | | 6.30 | |
| 252 | I1LJ17 | uncharacterized protein | 7 | -0.27 | 61.7 | | 6.28 | |
| 253 | I1MC31 | uncharacterized protein | 30 | -0.27 | 97.3 | | 4.87 | |
| 254 | I1M3C3 | uncharacterized protein | 16 | -0.27 | 34.0 | | 9.36 | |
| 255 | A0A0R0FZ23 | uncharacterized protein | 10 | -0.27 | 16.3 | | 11.02 | |
| 256 | C6T211 | uncharacterized protein | 4 | -0.28 | 9.3 | | 7.67 | |
| 257 | I1LCN1 | uncharacterized protein | 11 | -0.28 | 36.3 | | 6.26 | |
| 258 | I1L6V1 | uncharacterized protein | 4 | -0.29 | 47.1 | | 8.61 | |
| 259 | I1KS98 | uncharacterized protein | 10 | -0.29 | 46.7 | | 6.02 | |
| 260 | I1MIC1 | uncharacterized protein | 8 | -0.29 | 45.2 | | 4.84 | |
| 261 | K7KIL0 | gtp-binding nuclear protein | 8 | -0.30 | 25.0 | | 6.42 | |
| 262 | I1K5E6 | uncharacterized protein | 17 | -0.30 | 35.6 | | 7.62 | |
| 263 | I7FST9 | protein disulfide-isomerase | 26 | -0.30 | 58.7 | | 4.92 | |
| 264 | I1LNM2 | nadh dehydrogenase subunit 9 | 6 | -0.31 | 22.6 | | 7.71 | |
| 265 | A0A0R0K6C1 | uncharacterized protein | 22 | -0.31 | 61.6 | | 4.70 | |
| 266 | B5L5C7 | isoflavone synthase 1 | 18 | -0.31 | 58.9 | | 9.11 | |
| 267 | I1LZ03 | uncharacterized protein | 17 | -0.31 | 44.6 | | 10.48 | |
| 268 | I1NCV4 | uncharacterized protein | 14 | -0.32 | 50.0 | | 6.29 | |
| 269 | A0A0R4J364 | uncharacterized protein | 16 | -0.32 | 40.9 | | 5.97 | |
| 270 | I1NJ75 | uncharacterized protein | 5 | -0.32 | 29.3 | | 5.46 | |
| 271 | I1MRP0 | uncharacterized protein | 2 | -0.32 | 62.4 | | 8.58 | |
| 272 | I1KEH3 | uncharacterized protein | 8 | -0.32 | 37.6 | | 6.20 | |
| 273 | I1KT97 | uncharacterized protein | 9 | -0.32 | 28.4 | | 10.18 | |
| 274 | C6T9Z5 | formate dehydrogenase | 17 | -0.32 | 42.8 | | 6.28 | |
| 275 | I1K0G0 | uncharacterized protein | 11 | -0.33 | 43.1 | | 4.89 | |
| 276 | I1JSK2 | uncharacterized protein | 6 | -0.33 | 43.1 | | 6.13 | |
| 277 | K7MS29 | uncharacterized protein | 30 | -0.33 | 164.0 | | 6.34 | |
| 278 | I1M6D0 | uncharacterized protein | 9 | -0.33 | 83.1 | | 5.73 | |
| 279 | I1MU56 | uncharacterized protein | 18 | -0.34 | 82.2 | | 5.82 | |
| 280 | I1MNU2 | uncharacterized protein | 2 | -0.34 | 37.9 | | 6.75 | |
| 281 | A0A0R0ERR0 | uncharacterized protein | 19 | -0.34 | 47.4 | | 4.98 | |
| 282 | A0A0R0F7T3 | uncharacterized protein | 5 | -0.34 | 44.8 | | 7.19 | |
| 283 | I1KVA8 | adp,atp carrier protein | 6 | -0.35 | 67.8 | | 9.34 | |
| 284 | C6SWW6 | uncharacterized protein | 2 | -0.35 | 11.6 | | 9.37 | |
| 285 | I1MYB0 | uncharacterized protein | 23 | -0.35 | 53.4 | | 5.67 | |
| 286 | I1JXE8 | uncharacterized protein | 18 | -0.35 | 77.9 | | 5.09 | |
| 287 | K7K4Y1 | ubiquitin carboxyl-terminal hydrolase | 3 | -0.35 | 29.0 | | 5.26 | |
| 288 | C6SZW0 | uncharacterized protein | 9 | -0.36 | 22.1 | | 4.43 | |
| 289 | A0A0R4J4D6 | uncharacterized protein | 30 | -0.36 | 71.0 | | 5.10 | |
| 290 | C6TI64 | 40s ribosomal protein s3a | 13 | -0.36 | 29.7 | | 9.81 | |
| 291 | D2DKF1 | homoserine kinase | 7 | -0.36 | 37.6 | | 8.73 | |
| 292 | I1LE85 | uncharacterized protein | 5 | -0.37 | 37.4 | | 9.40 | |
| 293 | I1L1H2 | uncharacterized protein | 6 | -0.37 | 57.1 | | 6.10 | |
| 294 | I1K4Q5 | uncharacterized protein | 8 | -0.38 | 25.8 | | 10.18 | |
| 295 | V6CKR0 | expansin | 5 | -0.38 | 27.5 | | 9.16 | |
| 296 | K7KJ72 | uncharacterized protein | 19 | -0.38 | 404.8 | | 5.26 | |
| 297 | C6SXJ9 | cytochrome b-c1 complex subunit 7 | 5 | -0.38 | 14.6 | | 9.61 | |
| 298 | I1JCA6 | uncharacterized protein | 9 | -0.38 | 58.0 | | 4.70 | |
| 299 | I1MTJ0 | uncharacterized protein | 2 | -0.38 | 20.1 | | 9.30 | |
| 300 | C6T3K0 | uncharacterized protein | 2 | -0.39 | 22.5 | | 4.83 | |
| 301 | I1KY39 | citrate synthase | 13 | -0.40 | 56.4 | | 9.22 | |
| 302 | C6SWL4 | uncharacterized protein | 6 | -0.40 | 20.4 | | 10.24 | |
| 303 | I1LD03 | dolichyl-diphosphooligosaccharide--protein glycosyltransferase subunit 1 | 11 | -0.40 | 52.7 | | 6.79 | |
| 304 | C6SVV1 | uncharacterized protein | 3 | -0.40 | 18.4 | | 10.78 | |
| 305 | I1KXQ3 | uncharacterized protein | 7 | -0.40 | 29.8 | | 7.07 | |
| 306 | K7M9J2 | histone h4 | 5 | -0.40 | 20.6 | | 10.45 | |
| 307 | I1L571 | 60s ribosomal protein l13 | 6 | -0.41 | 23.8 | | 11.02 | |
| 308 | I1JNY7 | uncharacterized protein | 5 | -0.41 | 33.0 | | 8.90 | |
| 309 | I1NBE6 | uncharacterized protein | 17 | -0.41 | 82.1 | | 7.00 | |
| 310 | I1N1W7 | uncharacterized protein | 19 | -0.41 | 58.8 | | 6.49 | |
| 311 | I1JMB4 | glyceraldehyde-3-phosphate dehydrogenase | 9 | -0.41 | 44.6 | | 8.71 | |
| 312 | K7N1Q0 | uncharacterized protein | 9 | -0.41 | 106.1 | | 8.93 | |
| 313 | I1KWT3 | uncharacterized protein | 12 | -0.41 | 37.5 | | 5.96 | |
| 314 | I1MBM5 | uncharacterized protein | 2 | -0.41 | 8.3 | | 9.65 | |
| 315 | K7MAP3 | uncharacterized protein | 6 | -0.41 | 29.7 | | 9.34 | |
| 316 | A0A0R0FCD2 | uncharacterized protein | 12 | -0.42 | 48.7 | | 9.16 | |
| 317 | I1JIR8 | uncharacterized protein | 5 | -0.42 | 54.0 | | 5.22 | |
| 318 | I1KJW4 | uncharacterized protein | 3 | -0.42 | 8.7 | | 8.74 | |
| 319 | A0A0R4J3M4 | uncharacterized protein | 16 | -0.42 | 65.1 | | 5.85 | |
| 320 | C6TBS0 | dolichyl-diphosphooligosaccharide--protein glycosyltransferase 48 kda subunit | 13 | -0.42 | 48.1 | | 5.92 | |
| 321 | K7M8E9 | uncharacterized protein | 6 | -0.42 | 59.8 | | 5.28 | |
| 322 | C6TEF1 | uncharacterized protein | 4 | -0.42 | 40.8 | | 6.93 | |
| 323 | A0A0R0IY29 | uncharacterized protein | 14 | -0.43 | 54.4 | | 5.86 | |
| 324 | K7KHJ7 | uncharacterized protein | 7 | -0.43 | 18.0 | | 7.83 | |
| 325 | I1KQK6 | uncharacterized protein | 7 | -0.43 | 63.8 | | 8.89 | |
| 326 | I1NJF1 | uncharacterized protein | 7 | -0.43 | 52.3 | | 5.77 | |
| 327 | C6TKH1 | uncharacterized protein | 4 | -0.43 | 34.3 | | 6.46 | |
| 328 | I1N1X2 | uncharacterized protein | 13 | -0.43 | 54.2 | | 5.48 | |
| 329 | I1LV12 | uncharacterized protein | 19 | -0.43 | 68.3 | | 9.24 | |
| 330 | C6SY97 | mitochondrial pyruvate carrier | 4 | -0.44 | 12.2 | | 9.79 | |
| 331 | C6TDL2 | uncharacterized protein | 4 | -0.44 | 13.7 | | 9.16 | |
| 332 | I1LFH9 | uncharacterized protein | 7 | -0.44 | 219.3 | | 8.37 | |
| 333 | C6TCU7 | homoserine dehydrogenase | 10 | -0.44 | 40.6 | | 6.40 | |
| 334 | I1KP14 | uncharacterized protein | 30 | -0.44 | 108.7 | | 6.15 | |
| 335 | I1LRC2 | histone deacetylase hdt1 | 5 | -0.44 | 31.7 | | 4.75 | |
| 336 | I1KGH6 | uncharacterized protein | 16 | -0.44 | 54.4 | | 5.89 | |
| 337 | I1KSD7 | uncharacterized protein | 12 | -0.45 | 32.0 | | 9.41 | |
| 338 | D2D5G4 | methyltransferase | 12 | -0.46 | 40.3 | | 6.28 | |
| 339 | I1MZJ7 | uncharacterized protein | 4 | -0.46 | 59.9 | | 5.06 | |
| 340 | C6T9Q3 | uncharacterized protein | 7 | -0.46 | 17.2 | | 10.29 | |
| 341 | I1M1I0 | uncharacterized protein | 23 | -0.46 | 181.7 | | 6.21 | |
| 342 | I1LDE9 | uncharacterized protein | 18 | -0.46 | 44.4 | | 10.17 | |
| 343 | I1NB44 | uncharacterized protein | 10 | -0.47 | 31.5 | | 7.72 | |
| 344 | I1MDT4 | uncharacterized protein | 14 | -0.47 | 41.6 | | 5.31 | |
| 345 | C6TMM7 | uncharacterized protein | 8 | -0.47 | 23.4 | | 5.54 | |
| 346 | K7MB15 | uncharacterized protein | 8 | -0.47 | 89.9 | | 5.24 | |
| 347 | A0A0R4J5B9 | uncharacterized protein | 7 | -0.47 | 21.1 | | 8.76 | |
| 348 | I1M0K3 | cysteine proteinase inhibitor | 9 | -0.47 | 27.7 | | 6.10 | |
| 349 | I1KX25 | uncharacterized protein | 12 | -0.47 | 43.4 | | 9.02 | |
| 350 | I1L6A1 | uncharacterized protein | 17 | -0.48 | 47.2 | | 5.36 | |
| 351 | I1LXQ1 | uncharacterized protein | 13 | -0.48 | 79.5 | | 7.83 | |
| 352 | I1MTH5 | uncharacterized protein | 13 | -0.48 | 51.4 | | 5.73 | |
| 353 | I1K5D3 | eukaryotic translation initiation factor 3 subunit c | 21 | -0.48 | 104.2 | | 5.57 | |
| 354 | I1KGP3 | alpha-1,2-mannosidase | 8 | -0.48 | 65.1 | | 6.37 | |
| 355 | C6SV65 | histone h2a | 3 | -0.49 | 14.7 | | 10.36 | |
| 356 | C6SZN6 | uncharacterized protein | 8 | -0.49 | 17.9 | | 5.60 | |
| 357 | C6TGE9 | reticulon-like protein | 5 | -0.49 | 28.8 | | 6.80 | |
| 358 | I1NH00 | pyruvate kinase | 13 | -0.49 | 63.1 | | 6.82 | |
| 359 | A0A0R0I5G1 | uncharacterized protein | 27 | -0.49 | 124.7 | | 6.44 | |
| 360 | C6T5C9 | uncharacterized protein | 9 | -0.49 | 27.6 | | 6.39 | |
| 361 | C6SY14 | uncharacterized protein | 5 | -0.49 | 11.0 | | 9.35 | |
| 362 | Q00M91 | polyadenylate-binding protein | 9 | -0.50 | 68.5 | | 6.15 | |
| 363 | I1KXN9 | uncharacterized protein | 3 | -0.50 | 55.8 | | 6.62 | |
| 364 | C6TJ17 | uncharacterized protein | 11 | -0.50 | 33.6 | | 6.46 | |
| 365 | C6T891 | uncharacterized protein | 3 | -0.51 | 32.7 | | 9.47 | |
| 366 | K7L1L7 | tau class glutathione s-transferase | 5 | -0.51 | 25.8 | | 5.17 | |
| 367 | Q8W1A0 | cysteine synthase | 11 | -0.51 | 34.2 | | 5.69 | |
| 368 | I1ND96 | uncharacterized protein | 5 | -0.51 | 10.6 | | 6.82 | |
| 369 | A0A0R0KHH6 | uncharacterized protein | 8 | -0.51 | 22.5 | | 5.09 | |
| 370 | I1KTK0 | uncharacterized protein | 11 | -0.52 | 25.9 | | 10.07 | |
| 371 | C6TDW7 | uncharacterized protein | 4 | -0.52 | 25.5 | | 8.05 | |
| 372 | I1JJQ4 | uncharacterized protein | 19 | -0.52 | 89.7 | | 5.25 | |
| 373 | I1JHM4 | uncharacterized protein | 7 | -0.52 | 30.3 | | 5.86 | |
| 374 | C6SXD3 | 40s ribosomal protein s24 | 3 | -0.52 | 15.8 | | 10.64 | |
| 375 | C6TDZ0 | glutamate dehydrogenase | 15 | -0.53 | 44.5 | | 5.97 | |
| 376 | C6T7X6 | signal peptidase i | 4 | -0.54 | 20.0 | | 7.02 | |
| 377 | I1K974 | uncharacterized protein | 13 | -0.54 | 50.8 | | 9.31 | |
| 378 | A0A0R0GAV8 | uncharacterized protein | 10 | -0.54 | 92.0 | | 8.63 | |
| 379 | I1M5B6 | beta-galactosidase | 8 | -0.55 | 80.1 | | 7.65 | |
| 380 | I1K096 | uncharacterized protein | 19 | -0.55 | 61.0 | | 8.42 | |
| 381 | I1MJF7 | uncharacterized protein | 12 | -0.55 | 234.2 | | 5.01 | |
| 382 | I1JJN9 | uncharacterized protein | 8 | -0.55 | 51.5 | | 5.50 | |
| 383 | A0A0R0HSJ5 | peptidyl-prolyl cis-trans isomerase | 4 | -0.56 | 21.9 | | 8.60 | |
| 384 | C6T3V3 | glutathione peroxidase | 3 | -0.56 | 19.1 | | 9.63 | |
| 385 | Q9LLQ6 | seed maturation protein pm34 | 27 | -0.56 | 31.7 | | 6.60 | |
| 386 | A0A0R0I9M4 | annexin | 9 | -0.56 | 35.5 | | 8.85 | |
| 387 | K7LEA7 | uncharacterized protein | 4 | -0.56 | 39.1 | | 4.43 | |
| 388 | A0A0R0FB49 | uncharacterized protein | 4 | -0.57 | 66.5 | | 5.61 | |
| 389 | C6TDV2 | serine/threonine protein kinase | 11 | -0.57 | 39.1 | | 5.95 | |
| 390 | C6T2Y2 | uncharacterized protein | 3 | -0.57 | 18.6 | | 4.95 | |
| 391 | C6SWM6 | uncharacterized protein | 2 | -0.57 | 19.1 | | 8.86 | |
| 392 | A0A0R4J4S6 | uncharacterized protein | 13 | -0.57 | 43.2 | | 8.95 | |
| 393 | I1MUN3 | uncharacterized protein | 19 | -0.57 | 61.1 | | 8.55 | |
| 394 | I1JAC1 | uncharacterized protein | 4 | -0.58 | 83.2 | | 8.33 | |
| 395 | A0A0R4J5C6 | uncharacterized protein | 5 | -0.58 | 14.9 | | 5.74 | |
| 396 | Q948P5 | ferritin-4 | 11 | -0.59 | 27.5 | | 5.21 | |
| 397 | I1KVL0 | 40s ribosomal protein s12 | 8 | -0.59 | 14.9 | | 5.50 | |
| 398 | I1LPJ9 | uncharacterized protein | 12 | -0.59 | 77.5 | | 5.35 | |
| 399 | I1JD52 | uncharacterized protein | 19 | -0.59 | 58.6 | | 5.25 | |
| 400 | K7K223 | uncharacterized protein | 6 | -0.59 | 68.5 | | 6.20 | |
| 401 | I1M596 | lipoxygenase | 51 | -0.59 | 97.2 | | 6.21 | |
| 402 | C6SZX7 | glutathione peroxidase | 8 | -0.60 | 18.5 | | 6.59 | |
| 403 | I1KUQ9 | glycylpeptide n-tetradecanoyltransferase | 13 | -0.60 | 49.9 | | 6.07 | |
| 404 | C6KXH6 | protein disulfide isomerase l-3b | 11 | -0.60 | 58.8 | | 4.79 | |
| 405 | I1K146 | uncharacterized protein | 11 | -0.60 | 33.9 | | 6.72 | |
| 406 | I1LLM7 | uncharacterized protein | 20 | -0.60 | 65.2 | | 6.16 | |
| 407 | I1N3B9 | uncharacterized protein | 7 | -0.60 | 36.9 | | 8.93 | |
| 408 | K7S7Y8 | acyl-[acyl-carrier-protein] desaturase | 9 | -0.61 | 45.6 | | 6.35 | |
| 409 | K7LRU6 | uncharacterized protein | 8 | -0.61 | 77.8 | | 4.76 | |
| 410 | I1J9Q7 | glutamate dehydrogenase | 12 | -0.61 | 44.8 | | 5.97 | |
| 411 | C6SVX3 | uncharacterized protein | 8 | -0.61 | 17.7 | | 5.68 | |
| 412 | A0A0R4J5B2 | uncharacterized protein | 6 | -0.61 | 14.8 | | 5.34 | |
| 413 | I1JGU8 | phospho-2-dehydro-3-deoxyheptonate aldolase | 12 | -0.61 | 59.0 | | 8.57 | |
| 414 | I1KPQ5 | uncharacterized protein | 3 | -0.61 | 110.0 | | 6.34 | |
| 415 | C6TH97 | uncharacterized protein | 4 | -0.61 | 39.7 | | 7.05 | |
| 416 | I1LPS2 | uncharacterized protein | 6 | -0.62 | 202.2 | | 5.81 | |
| 417 | I1M5T2 | phospholipase d | 24 | -0.63 | 92.0 | | 5.90 | |
| 418 | K7MMF4 | uncharacterized protein | 2 | -0.63 | 22.0 | | 10.01 | |
| 419 | I1MT10 | uncharacterized protein | 27 | -0.63 | 70.8 | | 5.27 | |
| 420 | I1KBE3 | uncharacterized protein | 3 | -0.63 | 27.0 | | 10.12 | |
| 421 | C6TGZ7 | uncharacterized protein | 7 | -0.63 | 29.8 | | 7.07 | |
| 422 | C6T073 | uncharacterized protein | 7 | -0.63 | 21.2 | | 8.74 | |
| 423 | C6TFY7 | uncharacterized protein | 3 | -0.63 | 10.6 | | 7.99 | |
| 424 | K7LRU2 | uncharacterized protein | 10 | -0.63 | 33.2 | | 10.05 | |
| 425 | C6T0W1 | uncharacterized protein | 5 | -0.64 | 16.9 | | 11.05 | |
| 426 | A0A0R4J460 | uncharacterized protein | 11 | -0.64 | 46.3 | | 9.42 | |
| 427 | A0A0R0L5Y8 | uncharacterized protein | 3 | -0.64 | 28.9 | | 8.94 | |
| 428 | I1JLC4 | uncharacterized protein | 13 | -0.64 | 43.0 | | 6.25 | |
| 429 | C6TFT8 | annexin | 11 | -0.64 | 35.6 | | 6.06 | |
| 430 | I1JE70 | uncharacterized protein | 10 | -0.65 | 160.6 | | 7.40 | |
| 431 | I1LKP2 | nicalin | 15 | -0.65 | 62.6 | | 6.20 | |
| 432 | I1M984 | uncharacterized protein | 24 | -0.65 | 60.2 | | 5.81 | |
| 433 | A0A0R0K0U9 | uncharacterized protein | 11 | -0.66 | 101.3 | | 5.93 | |
| 434 | I1K6P4 | uncharacterized protein | 4 | -0.66 | 27.1 | | 5.44 | |
| 435 | I1LA26 | uncharacterized protein | 4 | -0.66 | 25.4 | | 6.09 | |
| 436 | I1MRP7 | uncharacterized protein | 5 | -0.66 | 100.2 | | 5.17 | |
| 437 | A0A0R0K553 | uncharacterized protein | 7 | -0.67 | 34.8 | | 5.42 | |
| 438 | I1LTF0 | uncharacterized protein | 2 | -0.67 | 51.8 | | 8.89 | |
| 439 | C6TK57 | uncharacterized protein | 5 | -0.67 | 36.2 | | 7.05 | |
| 440 | I1MDJ2 | uncharacterized protein | 5 | -0.67 | 17.3 | | 10.21 | |
| 441 | C6TGK4 | uncharacterized protein | 7 | -0.67 | 39.3 | | 6.63 | |
| 442 | A0A0R0FH00 | uncharacterized protein | 30 | -0.68 | 71.2 | | 5.09 | |
| 443 | I1K7L1 | uncharacterized protein | 3 | -0.68 | 58.0 | | 8.70 | |
| 444 | I1LZP7 | uncharacterized protein | 8 | -0.69 | 79.9 | | 5.81 | |
| 445 | C6TB70 | uncharacterized protein | 9 | -0.69 | 24.4 | | 6.44 | |
| 446 | A0A0R4J3L4 | uncharacterized protein | 10 | -0.69 | 27.4 | | 5.83 | |
| 447 | I1JDD2 | uncharacterized protein | 7 | -0.69 | 48.8 | | 6.74 | |
| 448 | I1N665 | uncharacterized protein | 5 | -0.69 | 58.3 | | 6.34 | |
| 449 | K7KNJ0 | uncharacterized protein | 2 | -0.70 | 16.5 | | 10.42 | |
| 450 | I1MVA1 | uncharacterized protein | 8 | -0.70 | 45.4 | | 5.17 | |
| 451 | I1LJA4 | uncharacterized protein | 15 | -0.70 | 60.8 | | 9.07 | |
| 452 | I1M5V6 | uncharacterized protein | 6 | -0.71 | 31.4 | | 8.10 | |
| 453 | K7LZJ0 | uncharacterized protein | 6 | -0.71 | 16.6 | | 10.53 | |
| 454 | I1L849 | uncharacterized protein | 8 | -0.72 | 27.4 | | 5.16 | |
| 455 | C6TJP8 | uncharacterized protein | 3 | -0.72 | 32.2 | | 5.07 | |
| 456 | A0A0R0GHN2 | uncharacterized protein | 18 | -0.72 | 44.6 | | 8.25 | |
| 457 | I1MQL3 | uncharacterized protein | 17 | -0.72 | 116.4 | | 9.39 | |
| 458 | I1LIM0 | uncharacterized protein | 11 | -0.72 | 92.0 | | 5.03 | |
| 459 | A0A0R0G0T0 | uncharacterized protein | 9 | -0.72 | 37.9 | | 5.41 | |
| 460 | I1KLL8 | uncharacterized protein | 2 | -0.72 | 32.0 | | 9.19 | |
| 461 | C6TK27 | uncharacterized protein | 5 | -0.72 | 38.1 | | 8.65 | |
| 462 | I1NGS1 | uncharacterized protein | 10 | -0.73 | 24.9 | | 4.51 | |
| 463 | K7M1P8 | uncharacterized protein | 3 | -0.73 | 107.6 | | 5.64 | |
| 464 | C6TJP6 | uncharacterized protein | 2 | -0.73 | 31.0 | | 9.35 | |
| 465 | C6TKW5 | uncharacterized protein | 5 | -0.74 | 49.7 | | 6.69 | |
| 466 | K7KEB2 | uncharacterized protein | 4 | -0.74 | 90.3 | | 5.19 | |
| 467 | I1KAR6 | uncharacterized protein | 6 | -0.74 | 34.2 | | 5.61 | |
| 468 | A0A0R0HI47 | uncharacterized protein | 3 | -0.75 | 50.2 | | 8.95 | |
| 469 | I1KMJ8 | uncharacterized protein | 7 | -0.75 | 122.1 | | 4.72 | |
| 470 | I1KUN9 | transmembrane 9 superfamily member | 6 | -0.75 | 66.9 | | 7.53 | |
| 471 | K7KPQ8 | uncharacterized protein | 7 | -0.75 | 48.9 | | 8.42 | |
| 472 | B3TDK6 | lipoxygenase | 55 | -0.75 | 96.7 | | 6.12 | |
| 473 | C6SWA6 | histone h2a | 3 | -0.75 | 15.7 | | 10.66 | |
| 474 | I1LPX6 | fructose-bisphosphate aldolase | 12 | -0.75 | 42.8 | | 6.86 | |
| 475 | K7KDF4 | uncharacterized protein | 3 | -0.75 | 20.9 | | 4.80 | |
| 476 | G3E7M9 | annexin | 17 | -0.76 | 35.9 | | 6.48 | |
| 477 | I1LE41 | uncharacterized protein | 9 | -0.76 | 26.2 | | 4.75 | |
| 478 | I1LV40 | uncharacterized protein | 10 | -0.76 | 39.2 | | 8.68 | |
| 479 | I1K3K3 | urease | 34 | -0.76 | 90.3 | | 5.70 | |
| 480 | I1J4C7 | uncharacterized protein | 2 | -0.76 | 57.4 | | 5.41 | |
| 481 | K7MTU1 | uncharacterized protein | 4 | -0.76 | 34.6 | | 6.77 | |
| 482 | K7M380 | mlo-like protein | 5 | -0.77 | 66.8 | | 10.35 | |
| 483 | I1LK85 | uncharacterized protein | 10 | -0.77 | 84.4 | | 5.87 | |
| 484 | A0A0R0ISD1 | uncharacterized protein | 6 | -0.77 | 84.6 | | 5.60 | |
| 485 | C6TBI4 | uncharacterized protein | 3 | -0.77 | 12.8 | | 10.61 | |
| 486 | I1JYG7 | uncharacterized protein | 7 | -0.79 | 24.2 | | 4.26 | |
| 487 | I1N4G3 | pectinesterase | 3 | -0.79 | 35.5 | | 5.92 | |
| 488 | A0A0R0I4F6 | uncharacterized protein | 25 | -0.79 | 31.8 | | 6.95 | |
| 489 | I1LQ57 | dolichyl-diphosphooligosaccharide--protein glycosyltransferase 48 kda subunit | 13 | -0.80 | 48.2 | | 5.92 | |
| 490 | M1FIP8 | nadh-ubiquinone oxidoreductase chain 5 | 2 | -0.80 | 74.0 | | 7.97 | |
| 491 | K7MQ57 | uncharacterized protein | 3 | -0.80 | 115.9 | | 5.76 | |
| 492 | I1KB73 | uncharacterized protein | 9 | -0.81 | 42.3 | | 5.91 | |
| 493 | K4FZF8 | serine hydroxymethyltransferase | 23 | -0.81 | 51.7 | | 7.59 | |
| 494 | C6T9S5 | uncharacterized protein | 4 | -0.81 | 48.5 | | 8.53 | |
| 495 | I1M6H0 | uncharacterized protein | 6 | -0.81 | 38.0 | | 8.16 | |
| 496 | A0A0R0IH13 | uncharacterized protein | 2 | -0.81 | 15.0 | | 8.46 | |
| 497 | I1LN49 | uncharacterized protein | 14 | -0.82 | 27.0 | | 6.56 | |
| 498 | C6TH20 | uncharacterized protein | 8 | -0.83 | 39.4 | | 5.96 | |
| 499 | I1LFA4 | uncharacterized protein | 7 | -0.83 | 43.2 | | 4.58 | |
| 500 | A0A0R0GBB6 | uncharacterized protein | 13 | -0.84 | 45.9 | | 5.90 | |
| 501 | I1JGK9 | uncharacterized protein | 8 | -0.84 | 15.9 | | 10.22 | |
| 502 | I1JIF2 | uncharacterized protein | 2 | -0.85 | 59.3 | | 6.26 | |
| 503 | I1M6X0 | uncharacterized protein | 6 | -0.86 | 35.0 | | 5.04 | |
| 504 | I1MBI9 | peroxidase | 9 | -0.86 | 40.0 | | 9.62 | |
| 505 | I1N628 | uncharacterized protein | 8 | -0.86 | 29.7 | | 9.06 | |
| 506 | K7MIW7 | uncharacterized protein | 7 | -0.86 | 82.3 | | 9.19 | |
| 507 | I1MYM9 | uncharacterized protein | 3 | -0.87 | 45.8 | | 8.17 | |
| 508 | I1LST6 | uncharacterized protein | 14 | -0.87 | 52.1 | | 8.97 | |
| 509 | C6TC87 | uncharacterized protein | 2 | -0.88 | 31.4 | | 4.64 | |
| 510 | K7KPF5 | uncharacterized protein | 6 | -0.90 | 47.7 | | 9.14 | |
| 511 | K7LDR4 | uncharacterized protein | 6 | -0.90 | 101.8 | | 4.83 | |
| 512 | I1L0S5 | uncharacterized protein | 5 | -0.91 | 19.0 | | 6.42 | |
| 513 | I1L386 | uncharacterized protein | 8 | -0.91 | 24.1 | | 4.47 | |
| 514 | B0M197 | peroxisomal voltage-dependent anion-selective channel protein | 9 | -0.92 | 29.8 | | 8.57 | |
| 515 | I1NHP0 | uncharacterized protein | 12 | -0.92 | 117.7 | | 6.44 | |
| 516 | I1J7C8 | uncharacterized protein | 10 | -0.92 | 42.9 | | 6.30 | |
| 517 | I1KFL7 | uncharacterized protein | 2 | -0.92 | 40.4 | | 5.44 | |
| 518 | C6THE6 | uncharacterized protein | 5 | -0.93 | 24.2 | | 9.10 | |
| 519 | C6SWV4 | uncharacterized protein | 6 | -0.93 | 18.8 | | 5.91 | |
| 520 | I1J6C3 | uncharacterized protein | 9 | -0.93 | 43.2 | | 9.03 | |
| 521 | C6T2L5 | uncharacterized protein | 2 | -0.93 | 16.3 | | 9.80 | |
| 522 | A0A0R4J3N3 | uncharacterized protein | 3 | -0.93 | 18.2 | | 6.97 | |
| 523 | I1MMC9 | uncharacterized protein | 5 | -0.94 | 23.7 | | 10.33 | |
| 524 | I1MJD2 | cysteine synthase | 6 | -0.94 | 33.9 | | 5.34 | |
| 525 | C6T433 | uncharacterized protein | 4 | -0.95 | 14.7 | | 5.09 | |
| 526 | A0A0R4J3Q7 | 18.5 kda class i heat shock protein | 5 | -0.95 | 18.5 | | 5.99 | |
| 527 | I1M281 | uncharacterized protein | 5 | -0.96 | 66.2 | | 5.18 | |
| 528 | C6SYZ8 | uncharacterized protein | 5 | -0.96 | 23.6 | | 10.33 | |
| 529 | C6SW67 | uncharacterized protein | 2 | -0.96 | 17.4 | | 5.16 | |
| 530 | I1L7G1 | cyanate hydratase | 6 | -0.97 | 18.6 | | 6.43 | |
| 531 | A0A0R0KMR6 | ribosomal protein l19 | 2 | -0.98 | 23.9 | | 11.38 | |
| 532 | C6SZZ3 | uncharacterized protein | 2 | -0.99 | 15.7 | | 5.29 | |
| 533 | A0A0R0I7I9 | uncharacterized protein | 5 | -1.00 | 44.8 | | 8.90 | |
| 534 | A0A0R0GPK2 | uncharacterized protein | 7 | -1.01 | 14.8 | | 10.58 | |
| 535 | K7MSK3 | uncharacterized protein | 2 | -1.01 | 51.0 | | 5.27 | |
| 536 | I1M5M5 | uncharacterized protein | 5 | -1.01 | 48.4 | | 4.86 | |
| 537 | I1KQK1 | uncharacterized protein | 5 | -1.01 | 27.2 | | 9.13 | |
| 538 | A0A0R4J467 | uncharacterized protein | 7 | -1.02 | 27.1 | | 7.13 | |
| 539 | A0A0R0HTR5 | uncharacterized protein | 16 | -1.03 | 125.8 | | 6.24 | |
| 540 | I1L4I8 | uncharacterized protein | 2 | -1.03 | 70.1 | | 8.09 | |
| 541 | K7MY05 | translocase of chloroplast | 8 | -1.03 | 39.6 | | 9.46 | |
| 542 | I1L1W0 | uncharacterized protein | 9 | -1.05 | 76.6 | | 5.43 | |
| 543 | K7KZI7 | uncharacterized protein | 2 | -1.06 | 154.1 | | 6.50 | |
| 544 | I1LLS4 | uncharacterized protein | 6 | -1.08 | 37.4 | | 7.59 | |
| 545 | K7LKS0 | uncharacterized protein | 6 | -1.08 | 121.7 | | 5.29 | |
| 546 | K7KQN4 | uncharacterized protein | 8 | -1.08 | 133.3 | | 4.48 | |
| 547 | I1KRD5 | uncharacterized protein | 7 | -1.09 | 14.9 | | 5.60 | |
| 548 | A0A0R0JPK0 | uncharacterized protein | 8 | -1.09 | 133.9 | | 6.24 | |
| 549 | K7KWZ7 | uncharacterized protein | 7 | -1.09 | 30.8 | | 5.87 | |
| 550 | C6T7I3 | uncharacterized protein | 3 | -1.09 | 37.3 | | 7.66 | |
| 551 | I1LBC8 | mitochondrial rho gtpase | 15 | -1.10 | 71.9 | | 5.40 | |
| 552 | K7MZX7 | histone h2a | 3 | -1.10 | 20.4 | | 10.99 | |
| 553 | C6T0R5 | uncharacterized protein | 5 | -1.12 | 21.0 | | 11.18 | |
| 554 | I1KU63 | uncharacterized protein | 32 | -1.12 | 247.2 | | 5.58 | |
| 555 | C6T4V0 | uncharacterized protein | 2 | -1.13 | 8.0 | | 4.25 | |
| 556 | I1J7A3 | uncharacterized protein | 2 | -1.13 | 51.6 | | 4.33 | |
| 557 | C6SZ97 | uncharacterized protein | 2 | -1.13 | 19.9 | | 9.62 | |
| 558 | C6SWT1 | uncharacterized protein | 5 | -1.13 | 15.9 | | 10.35 | |
| 559 | I1N877 | h/aca ribonucleoprotein complex subunit | 3 | -1.14 | 20.7 | | 11.49 | |
| 560 | I1L4W3 | uncharacterized protein | 6 | -1.15 | 73.1 | | 7.12 | |
| 561 | I1KCD7 | uncharacterized protein | 33 | -1.15 | 90.9 | | 5.11 | |
| 562 | I1KIE4 | uncharacterized protein | 5 | -1.16 | 32.2 | | 6.35 | |
| 563 | I1ML93 | uncharacterized protein | 4 | -1.17 | 57.3 | | 5.46 | |
| 564 | I1JYR8 | uncharacterized protein | 5 | -1.17 | 71.5 | | 5.60 | |
| 565 | Q9S7N8 | seed maturation protein pm21 | 2 | -1.17 | 10.1 | | 4.91 | |
| 566 | I1JB81 | uncharacterized protein | 3 | -1.18 | 47.1 | | 6.63 | |
| 567 | I1JFY3 | uncharacterized protein | 9 | -1.19 | 66.3 | | 8.00 | |
| 568 | K7MPZ2 | uncharacterized protein | 6 | -1.21 | 47.3 | | 6.06 | |
| 569 | I1KUJ7 | uncharacterized protein | 3 | -1.21 | 58.4 | | 6.08 | |
| 570 | I1LIY4 | uncharacterized protein | 9 | -1.21 | 149.2 | | 4.63 | |
| 571 | I1KPJ3 | endoglucanase | 4 | -1.25 | 68.7 | | 8.81 | |
| 572 | I1KG93 | glycylpeptide n-tetradecanoyltransferase | 13 | -1.27 | 49.9 | | 6.11 | |
| 573 | C6TN05 | hypersensitive induced reaction protein 4 | 4 | -1.27 | 32.6 | | 5.21 | |
| 574 | A0A0R0F4W4 | uncharacterized protein | 2 | -1.29 | 43.8 | | 9.74 | |
| 575 | I1J9W0 | 40s ribosomal protein s30 | 2 | -1.29 | 11.6 | | 12.07 | |
| 576 | I1KDP2 | uncharacterized protein | 7 | -1.30 | 49.3 | | 5.64 | |
| 577 | I1MDC4 | uncharacterized protein | 13 | -1.33 | 86.8 | | 7.04 | |
| 578 | I1JGY5 | glucose-6-phosphate isomerase | 19 | -1.33 | 67.2 | | 5.76 | |
| 579 | I1KB56 | uncharacterized protein | 2 | -1.34 | 37.7 | | 9.60 | |
| 580 | I1KUF4 | phosphatidylserine decarboxylase proenzyme 2 | 2 | -1.36 | 69.8 | | 6.03 | |
| 581 | I1M5K9 | kinesin-like protein | 3 | -1.36 | 78.5 | | 5.94 | |
| 582 | I1JTW0 | uncharacterized protein | 6 | -1.36 | 23.5 | | 9.53 | |
| 583 | C6SV69 | uncharacterized protein | 3 | -1.36 | 17.8 | | 6.62 | |
| 584 | I1JB78 | uncharacterized protein | 2 | -1.39 | 48.9 | | 5.53 | |
| 585 | C6SX10 | mitochondrial fission 1 protein | 4 | -1.39 | 18.7 | | 6.96 | |
| 586 | K7KG80 | uncharacterized protein | 3 | -1.42 | 35.1 | | 7.10 | |
| 587 | I1LBZ6 | uncharacterized protein | 6 | -1.43 | 19.0 | | 7.97 | |
| 588 | A0A0R0I7A8 | uncharacterized protein | 5 | -1.43 | 20.7 | | 6.44 | |
| 589 | I1MHX4 | uncharacterized protein | 2 | -1.43 | 61.7 | | 5.52 | |
| 590 | I1LCG9 | uncharacterized protein | 3 | -1.45 | 65.5 | | 6.48 | |
| 591 | K7L032 | uncharacterized protein | 3 | -1.46 | 58.6 | | 8.94 | |
| 592 | I1M4P9 | uncharacterized protein | 3 | -1.46 | 40.6 | | 8.70 | |
| 593 | A0A0R0EWR2 | uncharacterized protein | 7 | -1.47 | 52.9 | | 6.33 | |
| 594 | C6T3U6 | ribosomal protein l19 | 2 | -1.48 | 24.8 | | 11.45 | |
| 595 | I1K635 | uncharacterized protein | 7 | -1.50 | 54.7 | | 5.69 | |
| 596 | A0A0R0JQA9 | uncharacterized protein | 2 | -1.51 | 21.5 | | 6.85 | |
| 597 | I1JPH2 | uncharacterized protein | 4 | -1.52 | 20.2 | | 6.45 | |
| 598 | I1KVT5 | uncharacterized protein | 2 | -1.56 | 15.5 | | 5.76 | |
| 599 | I1JND9 | uncharacterized protein | 8 | -1.57 | 82.3 | | 6.18 | |
| 600 | I1LZJ8 | uncharacterized protein | 6 | -1.60 | 51.1 | | 5.37 | |
| 601 | K7K1R2 | uncharacterized protein | 7 | -1.63 | 47.8 | | 5.61 | |
| 602 | K7MPZ4 | uncharacterized protein | 2 | -1.64 | 15.7 | | 5.29 | |
| 603 | I1JCC9 | uncharacterized protein | 2 | -1.67 | 32.8 | | 11.33 | |
| 604 | A0A0G4DBR5 | udp-glycosyltransferase 79b30 | 2 | -1.69 | 51.1 | | 5.51 | |
| 605 | C6T5V0 | uncharacterized protein | 2 | -1.71 | 19.5 | | 9.65 | |
| 606 | I1N6R2 | 60s ribosomal protein l36 | 2 | -1.71 | 13.3 | | 10.99 | |
| 607 | I1MUP1 | uncharacterized protein | 5 | -1.71 | 84.4 | | 5.57 | |
| 608 | I1LJC4 | putative rrna methyltransferase | 2 | -1.72 | 93.7 | | 8.65 | |
| 609 | I1M4T8 | uncharacterized protein | 8 | -1.74 | 23.8 | | 6.74 | |
| 610 | A0A0R0IH39 | uncharacterized protein | 2 | -1.76 | 212.1 | | 6.95 | |
| 611 | I1LZK9 | uncharacterized protein | 3 | -1.76 | 65.4 | | 6.42 | |
| 612 | I1LE33 | uncharacterized protein | 8 | -1.83 | 68.2 | | 5.94 | |
| 613 | I1JZ49 | aldehyde dehydrogenase | 6 | -1.84 | 53.1 | | 7.64 | |
| 614 | C6TAN1 | uncharacterized protein | 19 | -1.86 | 47.4 | | 4.98 | |
| 615 | K7LXV5 | uncharacterized protein | 4 | -1.86 | 57.2 | | 9.16 | |
| 616 | A0A0R4J3L3 | uncharacterized protein | 6 | -1.88 | 43.0 | | 9.12 | |
| 617 | I1NA37 | uncharacterized protein | 4 | -1.91 | 21.0 | | 6.45 | |
| 618 | I1LKQ4 | uncharacterized protein | 2 | -1.93 | 12.3 | | 9.62 | |
| 619 | I1MCL4 | uncharacterized protein | 11 | -1.93 | 102.8 | | 6.48 | |
| 620 | A0A0R4J5L2 | uncharacterized protein | 5 | -1.95 | 27.1 | | 6.54 | |
| 621 | K7LDT9 | uncharacterized protein | 24 | -1.96 | 103.3 | | 6.26 | |
| 622 | K7KW51 | uncharacterized protein | 2 | -1.97 | 237.3 | | 6.16 | |
| 623 | O23957 | dehydrin | 3 | -1.99 | 17.3 | | 9.22 | |
| 624 | C6T4I7 | ribosomal protein l19 | 2 | -2.00 | 24.6 | | 11.45 | |
| 625 | I1KSN3 | uncharacterized protein | 6 | -2.06 | 77.0 | | 6.35 | |
| 626 | I1LB97 | uncharacterized protein | 6 | -2.07 | 14.1 | | 9.57 | |
| 627 | A0A0R0FTZ3 | uncharacterized protein | 7 | -2.12 | 51.0 | | 8.88 | |
| 628 | I1JYX6 | transcription elongation factor spt5 | 5 | -2.13 | 116.1 | | 5.17 | |
| 629 | C6SXM8 | ribosomal protein l19 | 2 | -2.16 | 24.6 | | 11.45 | |
| 630 | K7MEV8 | uncharacterized protein | 3 | -2.22 | 89.5 | | 9.65 | |
| 631 | A0A0R0F3J1 | uncharacterized protein | 3 | -2.23 | 53.2 | | 5.95 | |
| 632 | B4YB07 | phytochrome | 7 | -2.23 | 124.1 | | 6.21 | |
| 633 | I1LHP6 | uncharacterized protein | 10 | -2.27 | 81.7 | | 5.29 | |
| 634 | I1N1R1 | uncharacterized protein | 4 | -2.33 | 16.8 | | 4.64 | |
| 635 | M1FIU0 | ribosomal protein s3 | 2 | -2.34 | 65.1 | | 10.48 | |
| 636 | I1JXD8 | protein-l-isoaspartate o-methyltransferase | 3 | -2.38 | 35.8 | | 9.02 | |
| 637 | I1KV01 | uncharacterized protein | 3 | -2.39 | 36.1 | | 5.14 | |
| 638 | Q9M7N4 | mfp1 attachment factor 1 | 2 | -2.43 | 14.0 | | 4.60 | |
| 639 | C6T1Q2 | uncharacterized protein | 3 | -2.44 | 21.3 | | 8.64 | |
| 640 | I1K609 | uncharacterized protein | 9 | -2.47 | 55.1 | | 5.68 | |
| 641 | I1MTH6 | uncharacterized protein | 4 | -2.47 | 54.2 | | 5.47 | |
| 642 | I1N6Y7 | uncharacterized protein | 3 | -2.48 | 85.9 | | 6.66 | |
| 643 | Q9SEK9 | seed maturation protein pm25 | 9 | -2.49 | 25.7 | | 4.99 | |
| 644 | A0A0R4J4G9 | uncharacterized protein | 3 | -2.53 | 23.7 | | 6.46 | |
| 645 | A0A0R0F137 | uncharacterized protein | 5 | -2.54 | 25.5 | | 11.05 | |
| 646 | C6T3S4 | uncharacterized protein | 2 | -2.56 | 26.8 | | 6.41 | |
| 647 | I1LQA8 | uncharacterized protein | 5 | -2.56 | 38.9 | | 9.34 | |
| 648 | I1KAS7 | uncharacterized protein | 3 | -2.64 | 71.4 | | 5.56 | |
| 649 | I1JNW2 | trna (guanine-n(7)-)-methyltransferase non-catalytic subunit | 2 | -2.69 | 44.7 | | 5.59 | |
| 650 | C6TKX3 | uncharacterized protein | 2 | -2.70 | 27.2 | | 5.98 | |
| 651 | I1K246 | uncharacterized protein | 9 | -2.71 | 62.1 | | 5.60 | |
| 652 | A0A0R0KSG7 | ribosomal protein l19 | 2 | -2.72 | 24.7 | | 11.39 | |
| 653 | A0A0R4J2I4 | uncharacterized protein | 14 | -2.74 | 65.6 | | 5.62 | |
| 654 | K7MND3 | uncharacterized protein | 3 | -2.78 | 11.4 | | 9.16 | |
| 655 | C6T2R9 | uncharacterized protein | 4 | -2.80 | 19.8 | | 9.13 | |
| 656 | A0A0R0JG07 | uncharacterized protein | 2 | -2.81 | 49.3 | | 5.72 | |
| 657 | I1JMI7 | uncharacterized protein | 2 | -2.93 | 69.1 | | 7.23 | |
| 658 | I1N2Z5 | protein sle1 | 2 | -2.95 | 12.2 | | 5.33 | |
| 659 | I1NAW5 | uncharacterized protein | 2 | -2.97 | 50.5 | | 6.10 | |
| 660 | A0A0R4J354 | uncharacterized protein | 4 | -3.01 | 68.9 | | 5.20 | |
| 661 | I1KZ20 | peptidylprolyl isomerase | 12 | -3.05 | 60.9 | | 5.31 | |
| 662 | I1MSK1 | uncharacterized protein | 2 | -3.08 | 39.2 | | 8.85 | |
| 663 | K7L3S3 | uncharacterized protein | 4 | -3.12 | 410.5 | | 5.16 | |
| 664 | A0A0B5E8W0 | tau class glutathione s-transferase | 5 | -3.23 | 25.9 | | 5.82 | |
| 665 | I1MGF2 | uncharacterized protein | 2 | -3.29 | 65.5 | | 6.93 | |
| 666 | I1KTK9 | uncharacterized protein | 2 | -3.34 | 47.9 | | 8.77 | |
| 667 | I1JP51 | uncharacterized protein | 3 | -3.45 | 53.5 | | 9.76 | |
| 668 | I1L860 | uncharacterized protein | 12 | -3.54 | 58.0 | | 5.92 | |
| 669 | I1LS52 | uncharacterized protein | 2 | -3.79 | 41.9 | | 5.06 | |
| 670 | C6T085 | uncharacterized protein | 2 | -3.98 | 19.2 | | 6.88 | |
| 671 | Q39874 | lipoxygenase | 24 | -4.45 | 70.5 | | 8.86 | |
| 672 | C6T390 | uncharacterized protein | 5 | -5.43 | 16.2 | | 5.66 | |
| 673 | C6T9K9 | elongation factor tu | 14 | -5.58 | 49.8 | | 6.58 | |
| 674 | I1LPL2 | uncharacterized protein | 12 | -5.82 | 29.4 | | 10.21 | |

a “accession” is determined according to UniprotKB *Glycine* max (Soybean) protein database. b “M.P.” means the number of matched peptides. c “fold change” indicates log2 fold change of identified proteins from soybean irradiated with millimeter waves compared to unirradiated soybean under unflooded conditions.

**Table S3**. List of changed proteins between irradiated and unirradiated soybean seedlings with flooding stress.

| number | accessiona | description | M.P.b | fold changec | MW in kDa | pI |
| --- | --- | --- | --- | --- | --- | --- |
| 1 | I1MGV6 | 60s ribosomal protein l27 | 5 | 8.39 | 15.6 | 10.38 |
| 2 | K7K9Q0 | uncharacterized protein | 4 | 6.75 | 52.1 | 9.19 |
| 3 | I1J870 | uncharacterized protein | 6 | 6.52 | 105.9 | 5.88 |
| 4 | I1N4U1 | 6-phosphogluconate dehydrogenase | 23 | 5.72 | 53.6 | 5.68 |
| 5 | I1KTY1 | uncharacterized protein | 2 | 5.53 | 138 | 5.5 |
| 6 | I1LVS4 | uncharacterized protein | 6 | 5.5 | 61.7 | 5.04 |
| 7 | C6T0U3 | uncharacterized protein | 5 | 5.25 | 13.8 | 9.61 |
| 8 | C6SVG3 | uncharacterized protein | 6 | 5.19 | 17.5 | 4.6 |
| 9 | I1NHI4 | uncharacterized protein | 10 | 5.16 | 118.1 | 5.6 |
| 10 | K7MVZ7 | uncharacterized protein | 2 | 5.07 | 62.2 | 6.61 |
| 11 | I1L8F1 | uncharacterized protein | 4 | 5.04 | 30.5 | 6.52 |
| 12 | A0A0R0EW30 | uncharacterized protein | 17 | 4.95 | 47.3 | 5.42 |
| 13 | K7M5G4 | uncharacterized protein | 2 | 4.95 | 205.6 | 6.03 |
| 14 | K7LSG9 | uncharacterized protein | 2 | 4.92 | 10.8 | 5.29 |
| 15 | I1M3K1 | uncharacterized protein | 9 | 4.85 | 24.1 | 6.19 |
| 16 | I1NBA5 | uncharacterized protein | 2 | 4.84 | 26.4 | 9.28 |
| 17 | C6SVL0 | uncharacterized protein | 6 | 4.79 | 21.8 | 4.91 |
| 18 | I1KVT1 | uncharacterized protein | 3 | 4.65 | 40.8 | 4.53 |
| 19 | C6T488 | uncharacterized protein | 7 | 4.64 | 24.1 | 5.16 |
| 20 | I1MQD0 | t-complex protein 1 subunit gamma | 19 | 4.63 | 60.2 | 5.86 |
| 21 | C6T482 | uncharacterized protein | 2 | 4.6 | 10.6 | 9.37 |
| 22 | C6SXI6 | 40s ribosomal protein s26 | 5 | 4.55 | 14.9 | 10.92 |
| 23 | I1KB15 | uncharacterized protein | 2 | 4.51 | 32.8 | 6.34 |
| 24 | I1LL60 | uncharacterized protein | 2 | 4.51 | 31.3 | 7.06 |
| 25 | A0A0R0F3J1 | uncharacterized protein | 3 | 4.48 | 53.2 | 5.95 |
| 26 | I1MJK2 | uncharacterized protein | 3 | 4.44 | 73.5 | 7.64 |
| 27 | C6TGK4 | uncharacterized protein | 7 | 4.41 | 39.3 | 6.63 |
| 28 | I1JYP1 | uncharacterized protein | 2 | 4.33 | 25.7 | 5.77 |
| 29 | C6TEB0 | uncharacterized protein | 2 | 4.3 | 30 | 4.87 |
| 30 | I1J999 | glycosyltransferases | 2 | 4.25 | 55.4 | 9.62 |
| 31 | I1KVG8 | uncharacterized protein | 16 | 4.21 | 80.7 | 5.4 |
| 32 | C6T4K7 | uncharacterized protein | 2 | 4.19 | 20.3 | 5.14 |
| 33 | C6TL41 | eukaryotic translation initiation factor 3 subunit i | 12 | 4.11 | 35.9 | 6.26 |
| 34 | I1JEZ2 | eukaryotic translation initiation factor 2a | 3 | 4.1 | 56.7 | 8.98 |
| 35 | K7KBX9 | uncharacterized protein | 4 | 4.08 | 149.8 | 4.26 |
| 36 | I1KLZ7 | uncharacterized protein | 3 | 4.07 | 67.5 | 5.88 |
| 37 | I1LMM9 | uncharacterized protein | 2 | 4.06 | 21.7 | 9.09 |
| 38 | I1N4I0 | uncharacterized protein | 14 | 4 | 109.7 | 6.1 |
| 39 | I1MJH5 | uncharacterized protein | 6 | 3.97 | 46.7 | 5.48 |
| 40 | C6TE76 | uncharacterized protein | 5 | 3.93 | 27.3 | 6.42 |
| 41 | Q38JD2 | temperature-induced lipocalin | 4 | 3.86 | 21.4 | 6.62 |
| 42 | I1JBA6 | uncharacterized protein | 2 | 3.82 | 51 | 5.51 |
| 43 | C6TIG1 | uncharacterized protein | 2 | 3.82 | 28.7 | 9.65 |
| 44 | C6T2R9 | uncharacterized protein | 4 | 3.81 | 19.8 | 9.13 |
| 45 | I1N4F6 | uncharacterized protein | 7 | 3.79 | 57.9 | 6.47 |
| 46 | I1LG49 | peptidylprolyl isomerase | 2 | 3.75 | 55.4 | 5.45 |
| 47 | I1KPM9 | uncharacterized protein | 6 | 3.74 | 63.2 | 5.29 |
| 48 | C6TEX2 | chlorophyll a-b binding protein | 3 | 3.72 | 29.8 | 8.6 |
| 49 | I1MXJ1 | uncharacterized protein | 13 | 3.7 | 82.2 | 6.21 |
| 50 | C6TJL7 | xyloglucan endotransglucosylase/hydrolase | 4 | 3.67 | 34.2 | 8.25 |
| 51 | A0A0R0J9B8 | uncharacterized protein | 19 | 3.67 | 124.1 | 6.64 |
| 52 | C6SYN8 | uncharacterized protein | 3 | 3.6 | 15.5 | 7.74 |
| 53 | I1KUB0 | uncharacterized protein | 2 | 3.59 | 32.3 | 9.44 |
| 54 | I1L1E2 | uncharacterized protein | 8 | 3.54 | 47.5 | 5.36 |
| 55 | Q39834 | clathrin heavy chain | 52 | 3.53 | 193.2 | 5.37 |
| 56 | I1J9Q7 | glutamate dehydrogenase | 12 | 3.52 | 44.8 | 5.97 |
| 57 | I1L235 | uncharacterized protein | 4 | 3.51 | 36.1 | 4.56 |
| 58 | C6TLI1 | chlorophyll a-b binding protein | 3 | 3.51 | 27.4 | 6.91 |
| 59 | C6SW65 | uncharacterized protein | 3 | 3.43 | 20.1 | 8.74 |
| 60 | K7MJN4 | uncharacterized protein | 2 | 3.4 | 40 | 6.19 |
| 61 | I1KIQ3 | uncharacterized protein | 2 | 3.39 | 70.8 | 8.35 |
| 62 | I1LSH5 | uncharacterized protein | 2 | 3.37 | 44.7 | 9.65 |
| 63 | I1J550 | uncharacterized protein | 2 | 3.34 | 45.1 | 8.17 |
| 64 | I1K3E9 | uncharacterized protein | 10 | 3.34 | 88.5 | 7.54 |
| 65 | I1KWS8 | uncharacterized protein | 2 | 3.33 | 36 | 5.83 |
| 66 | I1K5Q4 | uncharacterized protein | 2 | 3.33 | 51.2 | 6.54 |
| 67 | K7LPS4 | uncharacterized protein | 3 | 3.28 | 70 | 7.13 |
| 68 | C6TBY2 | uncharacterized protein | 3 | 3.24 | 17.7 | 5.62 |
| 69 | Q70MR5 | ornithine decarboxylase | 3 | 3.21 | 46.3 | 5.41 |
| 70 | C6TEE8 | uroporphyrinogen decarboxylase | 4 | 3.2 | 42.7 | 7.67 |
| 71 | I1LRP4 | uncharacterized protein | 9 | 3.18 | 60.2 | 6.65 |
| 72 | I1L7G1 | cyanate hydratase | 6 | 3.15 | 18.6 | 6.43 |
| 73 | I1MMJ4 | uncharacterized protein | 5 | 3.15 | 68.9 | 8.88 |
| 74 | A0A0R0IIX2 | uncharacterized protein | 3 | 3.14 | 51.6 | 6.33 |
| 75 | I1MXV3 | uncharacterized protein | 2 | 3.14 | 72.9 | 6.04 |
| 76 | A0A0R0IG82 | uncharacterized protein | 6 | 3.13 | 56.5 | 5.99 |
| 77 | A0A0R0K8M4 | uncharacterized protein | 3 | 3.12 | 37.9 | 5.57 |
| 78 | C6T056 | uncharacterized protein | 3 | 3.11 | 25 | 8.95 |
| 79 | I1MKV5 | uncharacterized protein | 2 | 3.1 | 249.6 | 9.05 |
| 80 | K7MYF8 | uncharacterized protein | 2 | 3.1 | 53.2 | 5.58 |
| 81 | I1JJT0 | uncharacterized protein | 3 | 3.09 | 75.3 | 5.38 |
| 82 | K7KSI5 | uncharacterized protein | 2 | 3.09 | 41.8 | 6.03 |
| 83 | I1MVV5 | uncharacterized protein | 3 | 3.06 | 47.1 | 6.45 |
| 84 | I1L5W0 | uncharacterized protein | 3 | 3.06 | 17 | 9.27 |
| 85 | C6TFH1 | uncharacterized protein | 2 | 3.05 | 23.8 | 7.73 |
| 86 | K7KTE8 | uncharacterized protein | 3 | 3.05 | 99.3 | 6.79 |
| 87 | I1MXC3 | uncharacterized protein | 2 | 3.05 | 38.5 | 6.27 |
| 88 | Q2PMU2 | photosystem i p700 chlorophyll a apoprotein a2 | 8 | 3.04 | 82.4 | 6.8 |
| 89 | A0A0R0GIY4 | uncharacterized protein | 12 | 3.03 | 110.1 | 6.26 |
| 90 | A0A0R4J354 | uncharacterized protein | 4 | 3.03 | 68.9 | 5.2 |
| 91 | I1KV12 | uncharacterized protein | 3 | 3.02 | 30.9 | 8.23 |
| 92 | K7LK00 | uncharacterized protein | 3 | 3.02 | 83.4 | 5.68 |
| 93 | I1MYV4 | uncharacterized protein | 9 | 3.02 | 33 | 10.05 |
| 94 | K7LH76 | uncharacterized protein | 6 | 3.01 | 64.6 | 4.8 |
| 95 | A0A0R4J389 | uncharacterized protein | 3 | 3 | 24.3 | 9.73 |
| 96 | K7KW60 | uncharacterized protein | 4 | 2.99 | 31.8 | 7.6 |
| 97 | C6TH01 | uncharacterized protein | 3 | 2.99 | 29.3 | 6.61 |
| 98 | C6T1W3 | uncharacterized protein | 4 | 2.99 | 12.6 | 6.36 |
| 99 | Q2PMN3 | photosystem i iron-sulfur center | 3 | 2.99 | 9 | 6.65 |
| 100 | C6TE07 | uncharacterized protein | 19 | 2.98 | 47.1 | 8.85 |
| 101 | C6TIQ8 | uncharacterized protein | 8 | 2.96 | 47.9 | 7.26 |
| 102 | C6TJ88 | uncharacterized protein | 2 | 2.91 | 33.7 | 8.6 |
| 103 | I1L295 | uncharacterized protein | 2 | 2.89 | 60.1 | 6.15 |
| 104 | A0A0R0IH39 | uncharacterized protein | 2 | 2.87 | 212.1 | 6.95 |
| 105 | I1N084 | uncharacterized protein | 2 | 2.86 | 73.2 | 9.06 |
| 106 | K7KE29 | uncharacterized protein | 2 | 2.84 | 66.1 | 7.88 |
| 107 | I1MGL5 | uncharacterized protein | 5 | 2.83 | 60.5 | 5.27 |
| 108 | I1L8I0 | s-(hydroxymethyl)glutathione dehydrogenase | 2 | 2.83 | 40.7 | 6.51 |
| 109 | I1LYK3 | structural maintenance of chromosomes protein | 2 | 2.82 | 141.9 | 6.52 |
| 110 | C6TC17 | glycosyltransferase | 4 | 2.81 | 48.1 | 8.65 |
| 111 | I1MY29 | phosphotransferase | 8 | 2.8 | 53.4 | 5.48 |
| 112 | K7LAV5 | uncharacterized protein | 9 | 2.8 | 109.9 | 6.35 |
| 113 | C6TC69 | uncharacterized protein | 3 | 2.79 | 42 | 5.91 |
| 114 | A0A0M3Q190 | plastid gamma tocopherol methyl transferase | 3 | 2.77 | 32.9 | 7.05 |
| 115 | C6T9R6 | uncharacterized protein | 4 | 2.77 | 39.4 | 8.78 |
| 116 | I1L7R7 | CTP synthase | 4 | 2.77 | 61.6 | 5.69 |
| 117 | K7LD07 | uncharacterized protein | 7 | 2.76 | 213.1 | 8.56 |
| 118 | K7MFE9 | uncharacterized protein | 3 | 2.75 | 114.4 | 5.18 |
| 119 | C6T1F3 | uncharacterized protein | 2 | 2.73 | 12.1 | 9.62 |
| 120 | K7M2P2 | uncharacterized protein | 3 | 2.69 | 203.2 | 8.95 |
| 121 | I1JIF2 | uncharacterized protein | 2 | 2.64 | 59.3 | 6.26 |
| 122 | A0A0R0J0J8 | uncharacterized protein | 3 | 2.63 | 30.1 | 7.42 |
| 123 | C6TBP9 | uncharacterized protein | 2 | 2.62 | 36.3 | 6.94 |
| 124 | B0M194 | peroxisomal 3-hydroxyacyl-coa dehydrogenase-like protein | 3 | 2.62 | 35 | 9.48 |
| 125 | I1NCU3 | uncharacterized protein | 3 | 2.62 | 51.6 | 8.52 |
| 126 | Q9M7N4 | mfp1 attachment factor 1 | 2 | 2.6 | 14 | 4.6 |
| 127 | A0A0R0KPC8 | uncharacterized protein | 4 | 2.57 | 29.8 | 5.5 |
| 128 | I1JXR7 | uncharacterized protein | 2 | 2.57 | 47.1 | 6.2 |
| 129 | I1KTK9 | uncharacterized protein | 2 | 2.57 | 47.9 | 8.77 |
| 130 | A0A0R0JQA9 | uncharacterized protein | 2 | 2.56 | 21.5 | 6.85 |
| 131 | B0M1B1 | peroxisomal glycolate oxidase | 6 | 2.55 | 40.8 | 9.16 |
| 132 | K7K3P5 | uncharacterized protein | 6 | 2.55 | 26.8 | 7.01 |
| 133 | I1LPD3 | beta-adaptin-like protein | 15 | 2.55 | 99.9 | 4.96 |
| 134 | I1KXB7 | uncharacterized protein | 2 | 2.53 | 64 | 8.35 |
| 135 | I1LL42 | uncharacterized protein | 2 | 2.52 | 27.5 | 6.32 |
| 136 | C6TF49 | secretory carrier-associated membrane protein | 2 | 2.52 | 34.2 | 8.81 |
| 137 | K7MNQ7 | uncharacterized protein | 4 | 2.5 | 30.4 | 8.74 |
| 138 | I1LQ64 | uncharacterized protein | 7 | 2.5 | 52.1 | 5.34 |
| 139 | C6TI98 | uncharacterized protein | 6 | 2.47 | 35.9 | 8.37 |
| 140 | I1JR04 | uncharacterized protein | 3 | 2.47 | 77.1 | 8.5 |
| 141 | C6TCW7 | grpe protein homolog | 7 | 2.47 | 32.4 | 5.42 |
| 142 | I1JHW4 | uncharacterized protein | 2 | 2.44 | 18.8 | 10.42 |
| 143 | A0A0R0GUV6 | uncharacterized protein | 2 | 2.43 | 17.6 | 9.3 |
| 144 | I1JUS4 | uncharacterized protein | 7 | 2.42 | 54.9 | 5.14 |
| 145 | C6TFM7 | uncharacterized protein | 4 | 2.42 | 25.1 | 8.85 |
| 146 | I1MS38 | eukaryotic translation initiation factor 3 subunit k | 6 | 2.42 | 26.1 | 5.44 |
| 147 | I1LQ55 | glycosyltransferase | 2 | 2.4 | 49.6 | 8.64 |
| 148 | I1NIW7 | uncharacterized protein | 2 | 2.39 | 35.3 | 10.19 |
| 149 | I1MXT2 | uncharacterized protein | 3 | 2.39 | 101.1 | 5.6 |
| 150 | C6TMC2 | uncharacterized protein | 2 | 2.39 | 27.4 | 6.96 |
| 151 | C6SZH2 | uncharacterized protein | 6 | 2.38 | 17.5 | 4.52 |
| 152 | I1MB15 | uncharacterized protein | 18 | 2.38 | 402.1 | 5.16 |
| 153 | C6TGA9 | uncharacterized protein | 4 | 2.37 | 38.9 | 5.78 |
| 154 | I1N8U7 | uncharacterized protein | 5 | 2.33 | 40.4 | 6.6 |
| 155 | I1K9A8 | uncharacterized protein | 2 | 2.33 | 41.1 | 5.32 |
| 156 | B0M193 | peroxisomal enoyl-coa hydratase/isomerase family protein | 2 | 2.33 | 30.6 | 9.26 |
| 157 | A0A0R0J452 | uncharacterized protein | 2 | 2.31 | 25.2 | 5.69 |
| 158 | A0A0K2CT67 | bhlh transcription factor | 2 | 2.31 | 10.4 | 7.94 |
| 159 | I1MDR5 | uncharacterized protein | 5 | 2.3 | 48.8 | 5.27 |
| 160 | I1LG61 | beta-adaptin-like protein | 17 | 2.27 | 99.9 | 4.99 |
| 161 | K7KKI8 | uncharacterized protein | 3 | 2.24 | 31.5 | 6 |
| 162 | I1JVS1 | uncharacterized protein | 2 | 2.23 | 65.9 | 6.49 |
| 163 | I1M4P9 | uncharacterized protein | 3 | 2.23 | 40.6 | 8.7 |
| 164 | I1MM85 | uncharacterized protein | 14 | 2.21 | 58.5 | 6.26 |
| 165 | I1NIN5 | uncharacterized protein | 8 | 2.21 | 108.4 | 5.28 |
| 166 | I1MJ44 | uncharacterized protein | 2 | 2.2 | 99.6 | 4.91 |
| 167 | I1LPH2 | uncharacterized protein | 2 | 2.19 | 21 | 9.28 |
| 168 | I1LQY0 | uncharacterized protein | 4 | 2.16 | 92.9 | 8.79 |
| 169 | I1KY47 | uncharacterized protein | 4 | 2.16 | 53.9 | 8.41 |
| 170 | A0A0R0IVU0 | uncharacterized protein | 7 | 2.16 | 26.3 | 5.71 |
| 171 | I1L6C2 | uncharacterized protein | 8 | 2.15 | 85.6 | 6.38 |
| 172 | A0A0R0G3L8 | phospho-2-dehydro-3-deoxyheptonate aldolase | 7 | 2.14 | 54.9 | 8.54 |
| 173 | I1MT80 | uncharacterized protein | 4 | 2.14 | 14.4 | 9.39 |
| 174 | C6TJ97 | uncharacterized protein | 2 | 2.13 | 31.9 | 9.19 |
| 175 | A0A0R0JIT8 | uncharacterized protein | 12 | 2.13 | 99.7 | 5.93 |
| 176 | Q2PMT8 | photosystem ii d2 protein | 5 | 2.12 | 39.5 | 5.33 |
| 177 | Q43437 | chlorophyll a-b binding protein | 8 | 2.1 | 28 | 5.14 |
| 178 | I1JGY5 | glucose-6-phosphate isomerase | 19 | 2.09 | 67.2 | 5.76 |
| 179 | I1K775 | uncharacterized protein | 7 | 2.08 | 48.3 | 4.84 |
| 180 | C6TEX7 | uncharacterized protein | 2 | 2.07 | 28.9 | 5.69 |
| 181 | A0A0R0FS66 | uncharacterized protein | 5 | 2.05 | 106.7 | 4.69 |
| 182 | I1N2X1 | uncharacterized protein | 2 | 2.05 | 42.7 | 6.31 |
| 183 | I1JIS7 | uncharacterized protein | 3 | 2.04 | 57.4 | 9.31 |
| 184 | I1M4Y9 | uncharacterized protein | 13 | 2.03 | 87.5 | 7.29 |
| 185 | A0A0R0FVK4 | carboxypeptidase | 3 | 2.03 | 55.7 | 5.53 |
| 186 | I1K9T4 | uncharacterized protein | 14 | 2.02 | 401.4 | 5.23 |
| 187 | I1LGI8 | uncharacterized protein | 3 | 2.02 | 79.2 | 4.43 |
| 188 | I1MIW3 | ribokinase | 5 | 2.02 | 38 | 6.65 |
| 189 | C6TD22 | uncharacterized protein | 3 | 2.01 | 36.4 | 6.05 |
| 190 | I1J8D7 | phosphoserine aminotransferase | 11 | 2 | 45.5 | 8.45 |
| 191 | C6T0B7 | glycine cleavage system h protein | 3 | 1.99 | 21.9 | 5.8 |
| 192 | F7J077 | beta-conglycinin beta subunit | 11 | 1.99 | 50.4 | 5.67 |
| 193 | C6T142 | small ubiquitin-related modifier | 4 | 1.99 | 11.2 | 4.95 |
| 194 | I1L467 | uncharacterized protein | 3 | 1.98 | 53.4 | 10.39 |
| 195 | C6TGJ0 | uncharacterized protein | 2 | 1.98 | 32 | 4.67 |
| 196 | K7KZK7 | uncharacterized protein | 3 | 1.98 | 122 | 5.32 |
| 197 | I1JDN2 | uncharacterized protein | 4 | 1.96 | 49.8 | 6.09 |
| 198 | I1M311 | mannosyltransferase | 2 | 1.96 | 64.1 | 8.43 |
| 199 | A0A0R0KKU3 | uncharacterized protein | 35 | 1.95 | 71.4 | 5.04 |
| 200 | I1JJU7 | transmembrane 9 superfamily member | 5 | 1.94 | 75.1 | 6.44 |
| 201 | I1J785 | uncharacterized protein | 2 | 1.94 | 52 | 7.68 |
| 202 | I1K1T0 | uncharacterized protein | 18 | 1.94 | 176.1 | 8.38 |
| 203 | K7MGU2 | uncharacterized protein | 2 | 1.93 | 25.7 | 5.6 |
| 204 | I1KI20 | uncharacterized protein | 2 | 1.93 | 42.7 | 5.6 |
| 205 | K7M9Q3 | chlorophyll a-b binding protein | 2 | 1.92 | 27.6 | 6.15 |
| 206 | I1KZH6 | uncharacterized protein | 2 | 1.91 | 75.3 | 9.43 |
| 207 | C6SWH0 | uncharacterized protein | 2 | 1.9 | 18.3 | 10.09 |
| 208 | I1MT39 | uncharacterized protein | 5 | 1.9 | 21.2 | 5.76 |
| 209 | K7L5T9 | uncharacterized protein | 3 | 1.9 | 107.5 | 5.08 |
| 210 | I1MKY3 | uncharacterized protein | 5 | 1.89 | 12.6 | 6.24 |
| 211 | K7L6B6 | uncharacterized protein | 2 | 1.89 | 45.7 | 9.07 |
| 212 | I1JGD6 | uncharacterized protein | 3 | 1.88 | 63.1 | 5.61 |
| 213 | C6TBZ8 | uncharacterized protein | 2 | 1.87 | 40.4 | 8.4 |
| 214 | C6SX09 | uncharacterized protein | 7 | 1.87 | 29.2 | 5.91 |
| 215 | C6T9B7 | uncharacterized protein | 4 | 1.87 | 31.6 | 6.93 |
| 216 | I1N583 | uncharacterized protein | 6 | 1.87 | 48.5 | 5.75 |
| 217 | I1MVC1 | uncharacterized protein | 13 | 1.86 | 69.4 | 8.64 |
| 218 | I1KAV2 | uncharacterized protein | 4 | 1.86 | 38.8 | 4.49 |
| 219 | K7KGZ3 | uncharacterized protein | 3 | 1.85 | 129.5 | 5.67 |
| 220 | I1N7K5 | uncharacterized protein | 2 | 1.85 | 29.1 | 6.68 |
| 221 | I1MVH5 | uncharacterized protein | 4 | 1.83 | 121.8 | 5.41 |
| 222 | I1L281 | uncharacterized protein | 5 | 1.83 | 59.1 | 5.89 |
| 223 | I1N0Q9 | uncharacterized protein | 3 | 1.83 | 85.8 | 5.57 |
| 224 | I1NG02 | uncharacterized protein | 2 | 1.82 | 24.4 | 5.41 |
| 225 | A0A0R4J573 | uncharacterized protein | 3 | 1.82 | 39.9 | 9.23 |
| 226 | C6T487 | uncharacterized protein | 2 | 1.81 | 19.8 | 6.73 |
| 227 | I1MGG7 | uncharacterized protein | 9 | 1.81 | 60.1 | 6.88 |
| 228 | Q2PMQ5 | cytochrome b6 | 2 | 1.81 | 24.1 | 8.89 |
| 229 | K7M324 | uncharacterized protein | 4 | 1.81 | 15.3 | 4.96 |
| 230 | I1M1M2 | uncharacterized protein | 2 | 1.79 | 41.2 | 5.02 |
| 231 | I1JJF1 | uncharacterized protein | 6 | 1.78 | 44 | 6.16 |
| 232 | I1MUP1 | uncharacterized protein | 5 | 1.77 | 84.4 | 5.57 |
| 233 | K7L9D8 | uncharacterized protein | 3 | 1.76 | 143.1 | 5.83 |
| 234 | C6TKL8 | uncharacterized protein | 9 | 1.76 | 34.8 | 6.49 |
| 235 | C6TJY3 | peroxidase | 3 | 1.75 | 36 | 9.77 |
| 236 | I1KL35 | uncharacterized protein | 6 | 1.74 | 55 | 4.68 |
| 237 | C6TA28 | nucleoside diphosphate kinase | 2 | 1.74 | 25.2 | 9.16 |
| 238 | C6TM07 | uncharacterized protein | 10 | 1.74 | 24 | 5.99 |
| 239 | I1JNI2 | uncharacterized protein | 6 | 1.74 | 89 | 9.16 |
| 240 | I1KPW0 | uncharacterized protein | 4 | 1.74 | 54.4 | 5.92 |
| 241 | I1K3H5 | uncharacterized protein | 3 | 1.74 | 34.8 | 6.08 |
| 242 | I1MYM9 | uncharacterized protein | 3 | 1.73 | 45.8 | 8.17 |
| 243 | A0A0R0F2V7 | uncharacterized protein | 13 | 1.73 | 324 | 5.44 |
| 244 | I1NGD4 | uncharacterized protein | 4 | 1.73 | 32.6 | 5.47 |
| 245 | I1MLL7 | uncharacterized protein | 13 | 1.72 | 128.9 | 5.5 |
| 246 | C6SYG7 | cysteine proteinase inhibitor | 3 | 1.72 | 10.7 | 5.83 |
| 247 | A0A0R0I8Z5 | chlorophyll a-b binding protein | 5 | 1.72 | 31 | 5.72 |
| 248 | I1MRJ8 | uncharacterized protein | 6 | 1.7 | 78.9 | 8.86 |
| 249 | I1NBW7 | pescadillo homolog | 3 | 1.7 | 69.2 | 8.95 |
| 250 | I1L6I9 | uncharacterized protein | 4 | 1.7 | 27.3 | 5.14 |
| 251 | I1LQN8 | uncharacterized protein | 6 | 1.69 | 89.3 | 7.96 |
| 252 | I1J769 | uncharacterized protein | 4 | 1.69 | 98.9 | 4.77 |
| 253 | I1KTD7 | uncharacterized protein | 2 | 1.68 | 63.3 | 5.95 |
| 254 | I1KLY3 | uncharacterized protein | 2 | 1.68 | 35 | 5.47 |
| 255 | K7MZX7 | histone h2a | 3 | 1.68 | 20.4 | 10.99 |
| 256 | I1LBJ5 | zeta-carotene desaturase | 3 | 1.68 | 62.9 | 7.92 |
| 257 | I1K7A7 | glyceraldehyde-3-phosphate dehydrogenase | 11 | 1.67 | 48.4 | 6.76 |
| 258 | A0A0R4J642 | uncharacterized protein | 2 | 1.67 | 31.2 | 5.25 |
| 259 | I1MRC4 | uncharacterized protein | 9 | 1.65 | 97.1 | 7.37 |
| 260 | C6T4P6 | uncharacterized protein | 5 | 1.65 | 20.1 | 7.59 |
| 261 | I1MIT6 | annexin | 10 | 1.64 | 35.8 | 8.57 |
| 262 | A0A0R0JB68 | uncharacterized protein | 3 | 1.64 | 139.3 | 5.6 |
| 263 | C6T9K9 | elongation factor tu | 14 | 1.64 | 49.8 | 6.58 |
| 264 | I1JFE9 | serine/threonine-protein phosphatase | 5 | 1.63 | 96.1 | 5.97 |
| 265 | I1LJG3 | uncharacterized protein | 4 | 1.62 | 27.3 | 5.74 |
| 266 | Q96446 | actin | 4 | 1.61 | 22.4 | 5.45 |
| 267 | A0A0R0GIJ9 | uncharacterized protein | 4 | 1.58 | 48.1 | 6.77 |
| 268 | K7MSV4 | uncharacterized protein | 2 | 1.58 | 84.3 | 6.32 |
| 269 | C6T988 | imidazoleglycerol-phosphate dehydratase | 4 | 1.57 | 30.2 | 6.93 |
| 270 | I1MNK0 | uncharacterized protein | 9 | 1.57 | 35 | 6.66 |
| 271 | I1JQ96 | uncharacterized protein | 2 | 1.57 | 145.6 | 6.34 |
| 272 | I1LYC8 | uncharacterized protein | 2 | 1.56 | 22.5 | 9.52 |
| 273 | K7M6X1 | protein disulfide-isomerase | 5 | 1.56 | 55.8 | 5.13 |
| 274 | K7KVG4 | uncharacterized protein | 9 | 1.56 | 32.4 | 10 |
| 275 | A0A0R0JL71 | uncharacterized protein | 2 | 1.55 | 43.7 | 5.49 |
| 276 | A0A0R0H094 | pantothenate kinase 2 | 2 | 1.55 | 101.9 | 5.65 |
| 277 | D4Q9Z5 | soyasaponin iii rhamnosyltransferase | 15 | 1.54 | 53.5 | 5.87 |
| 278 | P15490 | stem 28 kda glycoprotein | 13 | 1.54 | 29 | 8.38 |
| 279 | I1J7Z7 | elongation factor ts | 8 | 1.54 | 122.5 | 4.63 |
| 280 | K7KL81 | uncharacterized protein | 4 | 1.54 | 59.5 | 5.87 |
| 281 | I1MIW4 | uncharacterized protein | 6 | 1.53 | 39.8 | 5.55 |
| 282 | I1L4U2 | serine hydroxymethyltransferase | 17 | 1.53 | 57 | 7.2 |
| 283 | C6TK14 | uncharacterized protein | 4 | 1.53 | 41.7 | 6.05 |
| 284 | I1NAK9 | uncharacterized protein | 19 | 1.52 | 98.8 | 5.36 |
| 285 | I1LFC4 | elongator complex protein 1 | 7 | 1.52 | 145.9 | 5.31 |
| 286 | A4ZPP2 | ethylene receptor | 2 | 1.52 | 70.9 | 6.25 |
| 287 | I1KRF9 | DNA helicase | 3 | 1.52 | 86.7 | 6.17 |
| 288 | K7MHI8 | uncharacterized protein | 3 | 1.52 | 98.7 | 5.54 |
| 289 | I1MRP7 | uncharacterized protein | 5 | 1.51 | 100.2 | 5.17 |
| 290 | I1LPY8 | coatomer subunit alpha | 31 | 1.5 | 136.7 | 6.55 |
| 291 | C6TGL4 | secretory carrier-associated membrane protein | 3 | 1.49 | 30.5 | 8.3 |
| 292 | I1L2W3 | uncharacterized protein | 2 | 1.49 | 180.5 | 6.47 |
| 293 | I1M3W1 | uncharacterized protein | 8 | 1.49 | 22.6 | 9.51 |
| 294 | A0A0R0KKR3 | uncharacterized protein | 16 | 1.48 | 35.2 | 6.02 |
| 295 | I1K642 | uncharacterized protein | 5 | 1.48 | 53.6 | 6.21 |
| 296 | I1LRH3 | uncharacterized protein | 10 | 1.47 | 82.7 | 4.84 |
| 297 | I1KMB5 | uncharacterized protein | 2 | 1.47 | 42.3 | 5.56 |
| 298 | I1J4C7 | uncharacterized protein | 2 | 1.47 | 57.4 | 5.41 |
| 299 | I1KZV8 | uncharacterized protein | 2 | 1.46 | 60.5 | 6.2 |
| 300 | I1K3D6 | uncharacterized protein | 2 | 1.46 | 45.9 | 4.96 |
| 301 | C6TIH7 | uncharacterized protein | 9 | 1.46 | 30.8 | 6.53 |
| 302 | K7KHN1 | uncharacterized protein | 4 | 1.46 | 101.5 | 4.98 |
| 303 | I1M8J4 | uncharacterized protein | 3 | 1.46 | 47 | 7.12 |
| 304 | I1KWP5 | uncharacterized protein | 2 | 1.46 | 45 | 6.94 |
| 305 | I1J8I8 | vacuolar protein sorting-associated protein 35 | 10 | 1.46 | 89.7 | 5.28 |
| 306 | I1KYS6 | uncharacterized protein | 2 | 1.46 | 35.2 | 6.39 |
| 307 | C6TMA8 | uncharacterized protein | 6 | 1.46 | 12.2 | 4.69 |
| 308 | I1JB05 | uncharacterized protein | 2 | 1.45 | 28.2 | 8.28 |
| 309 | I1JUR5 | uncharacterized protein | 2 | 1.45 | 57.1 | 6.26 |
| 310 | I1JBF6 | ATP-dependent clp protease proteolytic subunit | 5 | 1.45 | 33.8 | 8.67 |
| 311 | C6T2G5 | nascent polypeptide-associated complex subunit beta | 8 | 1.45 | 17.4 | 6.16 |
| 312 | I1JMW9 | uncharacterized protein | 13 | 1.45 | 271 | 5.82 |
| 313 | I1LFX6 | uncharacterized protein | 7 | 1.45 | 96.9 | 4.87 |
| 314 | I1J9V8 | protein transport protein sec16 | 4 | 1.45 | 154.3 | 4.96 |
| 315 | K7ML33 | uncharacterized protein | 6 | 1.44 | 128.6 | 4.74 |
| 316 | K7MTC5 | laccase | 2 | 1.43 | 64.2 | 8.47 |
| 317 | I1N5R7 | uncharacterized protein | 3 | 1.43 | 34.7 | 5.87 |
| 318 | I1KUU8 | uncharacterized protein | 6 | 1.43 | 56.2 | 8.95 |
| 319 | K7KSH9 | uncharacterized protein | 6 | 1.42 | 78.8 | 5.46 |
| 320 | Q2PMQ9 | photosystem ii cp47 reaction center protein | 6 | 1.42 | 56 | 6.2 |
| 321 | A0A0R0JZ89 | uncharacterized protein | 3 | 1.41 | 41.6 | 9.12 |
| 322 | A0A0R0G789 | uncharacterized protein | 18 | 1.41 | 56.1 | 9.12 |
| 323 | K7M988 | uncharacterized protein | 6 | 1.4 | 73.4 | 8.95 |
| 324 | K7KS32 | uncharacterized protein | 4 | 1.4 | 54.8 | 5.31 |
| 325 | I1M170 | glutamine synthetase | 7 | 1.4 | 47.6 | 6.42 |
| 326 | C6SYK2 | uncharacterized protein | 4 | 1.4 | 22.4 | 4.91 |
| 327 | I1L0S5 | uncharacterized protein | 5 | 1.39 | 19 | 6.42 |
| 328 | I1JP19 | deoxyhypusine hydroxylase | 3 | 1.39 | 32.6 | 4.68 |
| 329 | C6SWY4 | non-specific lipid-transfer protein | 3 | 1.39 | 12.5 | 8.69 |
| 330 | I1KAQ7 | uncharacterized protein | 4 | 1.39 | 55.1 | 5.76 |
| 331 | I1ML97 | alpha-mannosidase | 14 | 1.39 | 117 | 5.6 |
| 332 | A0A0R0J895 | terpene cyclase/mutase family member | 3 | 1.38 | 87.5 | 6.29 |
| 333 | I1NDG9 | uncharacterized protein | 2 | 1.38 | 43.1 | 5.77 |
| 334 | K7M1C2 | uncharacterized protein | 10 | 1.37 | 129 | 6.19 |
| 335 | K7MC48 | uncharacterized protein | 2 | 1.37 | 22.7 | 6.14 |
| 336 | I1MHS7 | uncharacterized protein | 3 | 1.37 | 65.4 | 9.5 |
| 337 | K7M923 | uncharacterized protein | 2 | 1.37 | 126.1 | 5.69 |
| 338 | I1KB42 | uncharacterized protein | 6 | 1.36 | 27.6 | 9.79 |
| 339 | K7MRN9 | uncharacterized protein | 3 | 1.36 | 93.7 | 5.4 |
| 340 | C6T0E3 | uncharacterized protein | 2 | 1.36 | 22.7 | 5.57 |
| 341 | C6TK27 | uncharacterized protein | 5 | 1.36 | 38.1 | 8.65 |
| 342 | Q2LAL4 | cytochrome p450 monooxygenase cyp83e8 | 7 | 1.36 | 57.4 | 8.2 |
| 343 | C6SVV1 | uncharacterized protein | 3 | 1.35 | 18.4 | 10.78 |
| 344 | K7K9K2 | uncharacterized protein | 2 | 1.35 | 240.7 | 5.34 |
| 345 | F6KBT6 | allene oxide cyclase 6 | 3 | 1.35 | 28.3 | 9.13 |
| 346 | A0A0R0F5Q2 | uncharacterized protein | 4 | 1.35 | 33.5 | 9.36 |
| 347 | A7LCD5 | lipoxygenase | 25 | 1.34 | 96.3 | 6.54 |
| 348 | I1MJC1 | uncharacterized protein | 2 | 1.34 | 137.9 | 5.56 |
| 349 | I1MPF4 | glycosyltransferase | 7 | 1.34 | 53.7 | 6.37 |
| 350 | A0A0R0IYE6 | lipoxygenase | 29 | 1.34 | 96.3 | 6.22 |
| 351 | I1KN82 | uncharacterized protein | 5 | 1.34 | 53.4 | 6.06 |
| 352 | I1NBX9 | ATP-dependent clp protease proteolytic subunit | 4 | 1.33 | 36 | 8.98 |
| 353 | I1MX58 | uncharacterized protein | 26 | 1.33 | 60.2 | 6.03 |
| 354 | I1JPP8 | uncharacterized protein | 3 | 1.33 | 56 | 6.26 |
| 355 | A0A0R0ELP8 | uncharacterized protein | 6 | 1.33 | 58.7 | 6.56 |
| 356 | A0A0R0IIS7 | uncharacterized protein | 6 | 1.33 | 45.4 | 8.21 |
| 357 | I1JZG1 | uncharacterized protein | 2 | 1.32 | 36.7 | 8.55 |
| 358 | I1JR00 | uncharacterized protein | 2 | 1.32 | 47.1 | 6.78 |
| 359 | A0A0R0JWL2 | uncharacterized protein | 2 | 1.32 | 21.2 | 8.8 |
| 360 | I1LWE5 | uncharacterized protein | 3 | 1.31 | 66.1 | 7.86 |
| 361 | K7LZ50 | uncharacterized protein | 16 | 1.31 | 115.6 | 5.67 |
| 362 | I1M6M4 | uncharacterized protein | 5 | 1.31 | 61.2 | 5.12 |
| 363 | I1JJ62 | citrate synthase | 6 | 1.31 | 56.5 | 8.74 |
| 364 | I1KM56 | carboxypeptidase | 8 | 1.31 | 49.1 | 6.03 |
| 365 | I1J5A5 | uncharacterized protein | 14 | 1.3 | 58.4 | 6.38 |
| 366 | I1MNI2 | uncharacterized protein | 3 | 1.3 | 39.6 | 6.33 |
| 367 | C6SVX9 | uncharacterized protein | 3 | 1.3 | 13.6 | 4.65 |
| 368 | K7MX92 | uncharacterized protein | 2 | 1.3 | 65.9 | 6.33 |
| 369 | Q9XHC6 | beta-amyrin 24-hydroxylase | 9 | 1.3 | 58.2 | 8.65 |
| 370 | I1MFL4 | uncharacterized protein | 9 | 1.29 | 33.3 | 5.73 |
| 371 | I1LPC3 | uncharacterized protein | 18 | 1.29 | 134.7 | 6.14 |
| 372 | C6T1W7 | NADH dehydrogenase | 4 | 1.29 | 18.3 | 8.87 |
| 373 | M1FIU8 | ATPase subunit 8 | 4 | 1.28 | 18.3 | 9.77 |
| 374 | K7M156 | uncharacterized protein | 3 | 1.28 | 38.5 | 6.17 |
| 375 | I1MZM7 | uncharacterized protein | 3 | 1.28 | 26.7 | 5.99 |
| 376 | I1LE01 | uncharacterized protein | 7 | 1.27 | 38.7 | 6.09 |
| 377 | I1LNT7 | uncharacterized protein | 4 | 1.26 | 83.7 | 5.74 |
| 378 | K7MF05 | uncharacterized protein | 8 | 1.25 | 88.7 | 5.95 |
| 379 | C6TGB2 | uncharacterized protein | 3 | 1.25 | 36.2 | 9.57 |
| 380 | I1K4M2 | glycosyltransferase | 11 | 1.25 | 55.8 | 5.74 |
| 381 | I1K3M8 | uncharacterized protein | 4 | 1.24 | 13.6 | 5.86 |
| 382 | I1N6Z5 | uncharacterized protein | 6 | 1.23 | 101.1 | 4.72 |
| 383 | K7KQN8 | uncharacterized protein | 11 | 1.23 | 83.5 | 5.87 |
| 384 | I1LEV4 | uncharacterized protein | 22 | 1.23 | 90.2 | 6.03 |
| 385 | I1LN65 | uncharacterized protein | 3 | 1.22 | 13.7 | 5.86 |
| 386 | C6TEP8 | uncharacterized protein | 4 | 1.22 | 38.5 | 6.34 |
| 387 | I1KRU1 | uncharacterized protein | 6 | 1.22 | 44.3 | 6.33 |
| 388 | I1JLC7 | uncharacterized protein | 12 | 1.21 | 96.2 | 6.34 |
| 389 | I1K4K1 | uncharacterized protein | 2 | 1.2 | 20.7 | 6.06 |
| 390 | I1LI35 | vacuolar protein sorting-associated protein 35 | 11 | 1.2 | 89.7 | 5.28 |
| 391 | C6T0C7 | uncharacterized protein | 7 | 1.2 | 23.4 | 4.73 |
| 392 | I1K380 | uncharacterized protein | 3 | 1.2 | 40.2 | 6.4 |
| 393 | K7LWI4 | ATP synthase subunit alpha | 8 | 1.2 | 55.7 | 5.15 |
| 394 | I1L5G4 | amidophosphoribosyltransferase | 12 | 1.19 | 62 | 6.38 |
| 395 | I1M676 | uncharacterized protein | 4 | 1.19 | 19.6 | 4.09 |
| 396 | K7M1G7 | alpha-1,4 glucan phosphorylase | 2 | 1.19 | 113.5 | 6.13 |
| 397 | I1MUE0 | uncharacterized protein | 2 | 1.19 | 38.2 | 6.5 |
| 398 | I1K4M7 | uncharacterized protein | 15 | 1.18 | 70.8 | 5.13 |
| 399 | A0A0R0HY16 | uncharacterized protein | 2 | 1.18 | 39.3 | 8.22 |
| 400 | I1M3C3 | uncharacterized protein | 16 | 1.18 | 34 | 9.36 |
| 401 | Q2PMS9 | ATP synthase subunit b | 3 | 1.18 | 21.1 | 8.55 |
| 402 | I1KV09 | uncharacterized protein | 10 | 1.18 | 30.3 | 5.81 |
| 403 | I1LJH2 | uncharacterized protein | 7 | 1.17 | 52 | 5.26 |
| 404 | C6TE24 | uncharacterized protein | 3 | 1.17 | 30.4 | 9.49 |
| 405 | I1KZI4 | uncharacterized protein | 8 | 1.17 | 50.3 | 5.69 |
| 406 | I1L8X8 | uncharacterized protein | 2 | 1.17 | 169.3 | 5.55 |
| 407 | K7K2H1 | uncharacterized protein | 4 | 1.16 | 74.1 | 4.71 |
| 408 | I1JKA3 | uncharacterized protein | 3 | 1.16 | 114 | 9.1 |
| 409 | I1MU67 | uncharacterized protein | 20 | 1.16 | 124.8 | 6.61 |
| 410 | I1KFL7 | uncharacterized protein | 2 | 1.15 | 40.4 | 5.44 |
| 411 | K7M5B1 | uncharacterized protein | 3 | 1.15 | 165 | 5.4 |
| 412 | I1JN71 | uncharacterized protein | 9 | 1.14 | 50.4 | 5.7 |
| 413 | Q39839 | nucleoside diphosphate kinase 1 | 7 | 1.14 | 16.4 | 5.91 |
| 414 | A0A0R4J2V8 | dirigent protein | 3 | 1.14 | 23.9 | 5.85 |
| 415 | K7K2V9 | uncharacterized protein | 8 | 1.13 | 196.5 | 6.96 |
| 416 | C6SVH4 | uncharacterized protein | 4 | 1.13 | 16 | 6.3 |
| 417 | I1KQK6 | uncharacterized protein | 7 | 1.13 | 63.8 | 8.89 |
| 418 | A0A0R0EYW9 | uncharacterized protein | 2 | 1.13 | 94.4 | 6.05 |
| 419 | C6TFQ5 | uncharacterized protein | 2 | 1.13 | 19.4 | 4.87 |
| 420 | A0A0R0FC89 | ATP-dependent clp protease proteolytic subunit | 4 | 1.12 | 38.5 | 9.69 |
| 421 | I1KAD3 | uncharacterized protein | 10 | 1.12 | 79.9 | 6.17 |
| 422 | K7MKF1 | uncharacterized protein | 2 | 1.12 | 29.5 | 9.3 |
| 423 | Q2V732 | vhs and gat domain protein | 4 | 1.12 | 72.9 | 5.39 |
| 424 | I1KXC2 | uncharacterized protein | 29 | 1.12 | 163.7 | 6.41 |
| 425 | I1JKB0 | uncharacterized protein | 8 | 1.12 | 57.5 | 8.76 |
| 426 | C6SWL3 | sm-like protein lsm2 | 2 | 1.11 | 10.7 | 6.83 |
| 427 | I1LIT2 | uncharacterized protein | 11 | 1.11 | 78.3 | 5.44 |
| 428 | K7L3Q8 | uncharacterized protein | 2 | 1.11 | 53.5 | 5.78 |
| 429 | I1KZ34 | hexosyltransferase | 2 | 1.11 | 64.1 | 9.21 |
| 430 | I1LCI8 | UDP-glycosyltransferase 79a6 | 3 | 1.1 | 52.2 | 5.99 |
| 431 | I1L5L1 | ribosomal protein | 4 | 1.1 | 37.9 | 9.25 |
| 432 | A0A0R0GIX5 | nicotinamide-nucleotide adenylyltransferase | 3 | 1.1 | 31.7 | 6.25 |
| 433 | I1JAY8 | uncharacterized protein | 10 | 1.1 | 71.2 | 5.52 |
| 434 | C6TNE6 | chlorophyll a-b binding protein | 8 | 1.1 | 27.9 | 5.29 |
| 435 | I1K5H2 | uncharacterized protein | 24 | 1.1 | 109.4 | 5.14 |
| 436 | I1KPH2 | uncharacterized protein | 10 | 1.1 | 26.8 | 5.87 |
| 437 | A0A0R0I3N7 | uncharacterized protein | 4 | 1.09 | 52.6 | 9.1 |
| 438 | I1N0P5 | uncharacterized protein | 5 | 1.09 | 20 | 9.3 |
| 439 | I1KEL7 | peroxidase | 9 | 1.09 | 34 | 8.66 |
| 440 | I1JEW0 | uncharacterized protein | 2 | 1.09 | 33.7 | 5.79 |
| 441 | C6SZ88 | translocon-associated protein subunit beta | 4 | 1.09 | 20.9 | 9.47 |
| 442 | I1LYP2 | uncharacterized protein | 3 | 1.09 | 10.3 | 10.26 |
| 443 | I1KR17 | uncharacterized protein | 3 | 1.08 | 32.5 | 8.99 |
| 444 | I1KJR9 | uncharacterized protein | 7 | 1.08 | 66.1 | 6.22 |
| 445 | I1LV56 | uncharacterized protein | 2 | 1.08 | 76.2 | 8.97 |
| 446 | A5A339 | endonuclease | 3 | 1.08 | 33.5 | 5.92 |
| 447 | P93164 | gamma-glutamyl hydrolase | 11 | 1.08 | 37.7 | 6.27 |
| 448 | Q9SLW5 | protoporphyrinogen oxidase | 4 | 1.08 | 55 | 9.05 |
| 449 | K7N241 | uncharacterized protein | 2 | 1.07 | 39.9 | 8.68 |
| 450 | I1KSA8 | serine/threonine-protein phosphatase | 8 | 1.07 | 35.6 | 5.21 |
| 451 | A0A0R0JPH2 | uncharacterized protein | 3 | 1.07 | 69.6 | 8.53 |
| 452 | I1KSA2 | uncharacterized protein | 4 | 1.07 | 19.5 | 5.83 |
| 453 | I1MUQ0 | uncharacterized protein | 2 | 1.07 | 27.4 | 5.98 |
| 454 | K7MNV4 | uncharacterized protein | 4 | 1.07 | 22.1 | 5.13 |
| 455 | I1LG13 | uncharacterized protein | 14 | 1.07 | 122.9 | 5.41 |
| 456 | I1NJ39 | uncharacterized protein | 6 | 1.07 | 124.7 | 6.97 |
| 457 | I1LT58 | uncharacterized protein | 6 | 1.07 | 21.3 | 8.48 |
| 458 | Q2PMQ2 | 30s ribosomal protein s11 | 2 | 1.06 | 15 | 12.11 |
| 459 | A8VFK7 | msi1 | 4 | 1.06 | 48.4 | 4.77 |
| 460 | I1L899 | uncharacterized protein | 10 | 1.06 | 58.5 | 6.85 |
| 461 | I1MVS9 | uncharacterized protein | 6 | 1.06 | 43.8 | 7.57 |
| 462 | C6SWV6 | 40s ribosomal protein s27 | 4 | 1.06 | 10.1 | 8.98 |
| 463 | C6TM13 | uncharacterized protein | 3 | 1.06 | 30 | 5.64 |
| 464 | I1ML46 | uncharacterized protein | 13 | 1.06 | 56.5 | 5 |
| 465 | K7LNX9 | uncharacterized protein | 3 | 1.06 | 153.3 | 5.97 |
| 466 | K7LRR0 | phosphoinositide phospholipase c | 5 | 1.05 | 69 | 6.22 |
| 467 | I1L362 | uncharacterized protein | 46 | 1.05 | 147.8 | 5.82 |
| 468 | I1LJG9 | uncharacterized protein | 17 | 1.05 | 83.8 | 5.47 |
| 469 | I1K764 | glucose-1-phosphate adenylyltransferase | 8 | 1.05 | 57 | 6.39 |
| 470 | I1KKF1 | uncharacterized protein | 2 | 1.04 | 30.4 | 6.17 |
| 471 | I1L1F3 | uncharacterized protein | 8 | 1.04 | 28.3 | 9.83 |
| 472 | K7KI34 | calcium-transporting atpase | 5 | 1.04 | 109.8 | 5.67 |
| 473 | I1NAV1 | uncharacterized protein | 4 | 1.04 | 145.3 | 6.19 |
| 474 | A0A0R0FJ21 | uncharacterized protein | 5 | 1.04 | 22.4 | 5.15 |
| 475 | A0A0R0FAD6 | uncharacterized protein | 6 | 1.03 | 51.2 | 9.15 |
| 476 | I1JWV8 | uncharacterized protein | 4 | 1.03 | 35.7 | 5.26 |
| 477 | I1NET6 | uncharacterized protein | 12 | 1.02 | 82 | 4.34 |
| 478 | Q6T300 | cyclin-dependent kinases regulatory subunit | 2 | 1.02 | 10.6 | 9.05 |
| 479 | I1JCZ0 | v-type proton atpase subunit c | 8 | 1.02 | 42.5 | 5.67 |
| 480 | I1MCV9 | uncharacterized protein | 6 | 1.02 | 52.8 | 5.82 |
| 481 | F8SMC4 | 1-aminocyclopropane-1-carboxylate oxidase | 11 | 1.02 | 35.8 | 5.04 |
| 482 | I1KTL3 | uncharacterized protein | 2 | 1.02 | 75.4 | 9.29 |
| 483 | I1KTY9 | sulfurtransferase | 11 | 1.02 | 41.8 | 6.37 |
| 484 | K7LCK9 | uncharacterized protein | 3 | 1.02 | 108.4 | 5.91 |
| 485 | I1LR23 | uncharacterized protein | 16 | 1.01 | 109.5 | 7.06 |
| 486 | I1JY44 | uncharacterized protein | 4 | 1.01 | 58.7 | 5.67 |
| 487 | I1NCB1 | uncharacterized protein | 7 | 1.01 | 47 | 5.46 |
| 488 | I1LGW7 | uncharacterized protein | 16 | 1.01 | 487.9 | 5.86 |
| 489 | I1J4B5 | uncharacterized protein | 6 | 1.01 | 51 | 6.2 |
| 490 | I1ME34 | uncharacterized protein | 5 | 1.01 | 66.2 | 6.23 |
| 491 | K7LLB1 | uncharacterized protein | 13 | 1.01 | 58 | 4.68 |
| 492 | I1N783 | proteasome subunit alpha type | 10 | 1.01 | 27.3 | 5.59 |
| 493 | C6TG34 | 4-hydroxy-tetrahydrodipicolinate synthase | 6 | 1.01 | 39.9 | 6.41 |
| 494 | I1KUE9 | uncharacterized protein | 2 | 1 | 38.8 | 8.51 |
| 495 | C6SY25 | uncharacterized protein | 5 | 1 | 13.8 | 9.61 |
| 496 | I1JUQ6 | tRNA (guanine-n(7)-)-methyltransferase | 2 | 1 | 28.9 | 6.4 |
| 497 | Q2TUV8 | anthocyanidin synthase 2 | 5 | 1 | 39.9 | 5.62 |
| 498 | A0A0R0IYY7 | uncharacterized protein | 22 | 0.99 | 59 | 8.78 |
| 499 | I1N6T7 | uncharacterized protein | 5 | 0.99 | 46 | 5.96 |
| 500 | I1L555 | uncharacterized protein | 8 | 0.99 | 68.4 | 8.48 |
| 501 | I1JIR2 | uncharacterized protein | 6 | 0.99 | 101.1 | 9.04 |
| 502 | I1K5A7 | uncharacterized protein | 8 | 0.99 | 70.9 | 6.16 |
| 503 | C6T778 | uncharacterized protein | 5 | 0.99 | 28.6 | 9.41 |
| 504 | D4N5G0 | alpha-form rubisco activase | 9 | 0.99 | 52.2 | 5.94 |
| 505 | I1NHC6 | uncharacterized protein | 15 | 0.98 | 63.2 | 6.22 |
| 506 | Q2PMQ0 | 30s ribosomal protein s8 | 3 | 0.98 | 15.7 | 10.31 |
| 507 | C6TE22 | uncharacterized protein | 3 | 0.98 | 27 | 5.41 |
| 508 | I1M928 | uncharacterized protein | 2 | 0.98 | 51 | 8.91 |
| 509 | I1JS41 | uncharacterized protein | 8 | 0.98 | 82.9 | 4.89 |
| 510 | Q6LED6 | ATP synthase subunit 9 | 2 | 0.98 | 7.6 | 8.28 |
| 511 | I1MFD9 | uncharacterized protein | 5 | 0.98 | 53.2 | 5.92 |
| 512 | C5HU39 | air12 | 3 | 0.98 | 25.6 | 7.98 |
| 513 | I1MJM1 | clustered mitochondria protein homolog | 14 | 0.97 | 155.9 | 5.94 |
| 514 | C6ZS03 | leucine rich repeat protein | 15 | 0.97 | 39.7 | 8.51 |
| 515 | I1LVI3 | uncharacterized protein | 19 | 0.97 | 100.1 | 9.13 |
| 516 | I1MAY4 | uncharacterized protein | 8 | 0.96 | 71.5 | 5.04 |
| 517 | I1NGC4 | uncharacterized protein | 11 | 0.96 | 76.9 | 6.77 |
| 518 | C6SVL2 | ribulose bisphosphate carboxylase small chain | 6 | 0.96 | 20 | 8.87 |
| 519 | I1MZJ7 | uncharacterized protein | 4 | 0.96 | 59.9 | 5.06 |
| 520 | C6SV85 | v-type proton atpase subunit g | 2 | 0.96 | 12.3 | 6.85 |
| 521 | C6SWD5 | uncharacterized protein | 2 | 0.95 | 19.7 | 8.61 |
| 522 | I1K0Z2 | lon protease homolog 2 | 2 | 0.95 | 98.2 | 8.49 |
| 523 | C6TNL4 | uncharacterized protein | 5 | 0.95 | 37.3 | 8.24 |
| 524 | A0A0R0I5R9 | uncharacterized protein | 14 | 0.95 | 48 | 6.06 |
| 525 | K7KID6 | uncharacterized protein | 2 | 0.95 | 24.4 | 8.93 |
| 526 | I1LM96 | 60s ribosomal export protein nmd3 | 3 | 0.94 | 58.5 | 6.27 |
| 527 | A0A0R0IQZ2 | uncharacterized protein | 11 | 0.94 | 84.8 | 5.62 |
| 528 | I1KXI0 | uncharacterized protein | 4 | 0.94 | 96.8 | 5.75 |
| 529 | K7KS35 | theta class glutathione s-transferase | 4 | 0.94 | 28.4 | 9.66 |
| 530 | I1KY45 | glutamyl-trna(gln) amidotransferase subunit a | 5 | 0.94 | 57.6 | 6.28 |
| 531 | Q2PMS5 | 30s ribosomal protein s16 | 2 | 0.94 | 10.4 | 10.6 |
| 532 | A0A0R0I3E2 | uncharacterized protein | 2 | 0.94 | 94.5 | 6.71 |
| 533 | I1JQF9 | uncharacterized protein | 3 | 0.94 | 46.9 | 9 |
| 534 | I1JN48 | DNA gyrase subunit b | 6 | 0.94 | 81.1 | 8.58 |
| 535 | I1MNV0 | superoxide dismutase | 2 | 0.94 | 17.2 | 7.19 |
| 536 | I1KS24 | uncharacterized protein | 9 | 0.93 | 32.7 | 8.56 |
| 537 | K7KZJ7 | uncharacterized protein | 18 | 0.93 | 86.2 | 6.16 |
| 538 | K7K5W8 | uncharacterized protein | 5 | 0.93 | 86.4 | 5.83 |
| 539 | C6T3L6 | uncharacterized protein | 2 | 0.93 | 19.3 | 4.55 |
| 540 | K7K6H1 | uncharacterized protein | 2 | 0.93 | 24.7 | 7.9 |
| 541 | K7KMK0 | uncharacterized protein | 3 | 0.93 | 64.8 | 9.7 |
| 542 | I1LBC8 | mitochondrial rho gtpase | 15 | 0.93 | 71.9 | 5.4 |
| 543 | A7BIC9 | malonyl-coa:isoflavone 7-o-glucoside-6''-o-malonyltransferase | 14 | 0.93 | 51.6 | 5.98 |
| 544 | I1M375 | uncharacterized protein | 18 | 0.93 | 69.3 | 9.02 |
| 545 | I1MCE9 | uncharacterized protein | 3 | 0.93 | 40.8 | 5.76 |
| 546 | C6THU0 | uncharacterized protein | 11 | 0.92 | 35 | 5.29 |
| 547 | C6TLM5 | uncharacterized protein | 8 | 0.92 | 27.7 | 8.67 |
| 548 | A0A0R0FB29 | uncharacterized protein | 3 | 0.92 | 29.6 | 8.66 |
| 549 | C6TDZ1 | uncharacterized protein | 5 | 0.92 | 30.7 | 6.19 |
| 550 | A0A0R0IX14 | uncharacterized protein | 14 | 0.92 | 109.7 | 6.34 |
| 551 | C6TNA3 | uncharacterized protein | 11 | 0.92 | 26.7 | 7.79 |
| 552 | I1ML09 | uncharacterized protein | 14 | 0.92 | 123.4 | 4.88 |
| 553 | I1JXD5 | uncharacterized protein | 5 | 0.91 | 83.1 | 5.17 |
| 554 | I1KV23 | uncharacterized protein | 11 | 0.91 | 167.7 | 8.08 |
| 555 | I1KPV1 | uncharacterized protein | 16 | 0.91 | 54.8 | 5.56 |
| 556 | I1JVJ8 | uncharacterized protein | 2 | 0.91 | 54.4 | 8.89 |
| 557 | I1KW34 | uncharacterized protein | 8 | 0.91 | 45 | 4.87 |
| 558 | K7LLJ2 | uncharacterized protein | 8 | 0.91 | 68.8 | 5.87 |
| 559 | I1JE93 | uncharacterized protein | 2 | 0.91 | 25.9 | 8.26 |
| 560 | I1MG54 | histidinol dehydrogenase | 8 | 0.9 | 51.3 | 6.29 |
| 561 | I1M0X0 | uncharacterized protein | 11 | 0.9 | 64.5 | 6.69 |
| 562 | C6TB02 | uncharacterized protein | 5 | 0.9 | 36.5 | 6.04 |
| 563 | A0A0R4J4S6 | uncharacterized protein | 13 | 0.9 | 43.2 | 8.95 |
| 564 | A0A0R0J8F6 | uncharacterized protein | 3 | 0.9 | 59.7 | 5.91 |
| 565 | C6T504 | uncharacterized protein | 2 | 0.9 | 10.3 | 8.06 |
| 566 | I1JSJ3 | fructose-bisphosphate aldolase | 7 | 0.89 | 42.5 | 6.38 |
| 567 | I1JGN1 | uncharacterized protein | 9 | 0.89 | 53.2 | 6.76 |
| 568 | I1JIU3 | uncharacterized protein | 3 | 0.89 | 68.3 | 6.99 |
| 569 | C6SYA3 | vacuolar protein sorting-associated protein 29 | 3 | 0.89 | 21.1 | 6.09 |
| 570 | I1JUQ4 | uncharacterized protein | 12 | 0.89 | 49.5 | 9.18 |
| 571 | I1JHJ8 | uncharacterized protein | 19 | 0.89 | 109.9 | 5.98 |
| 572 | K7MH27 | uncharacterized protein | 5 | 0.89 | 26.5 | 9.23 |
| 573 | K7KRC0 | uncharacterized protein | 10 | 0.88 | 48.2 | 5.41 |
| 574 | I1JYG3 | uncharacterized protein | 12 | 0.88 | 21.7 | 9.52 |
| 575 | I1KEH3 | uncharacterized protein | 8 | 0.88 | 37.6 | 6.2 |
| 576 | A0A0R0GAV8 | uncharacterized protein | 10 | 0.88 | 92 | 8.63 |
| 577 | A0A0R0IRU4 | uncharacterized protein | 5 | 0.88 | 127.8 | 4.51 |
| 578 | Q9FQE7 | glutathione s-transferase gst 11 | 7 | 0.88 | 25.5 | 5.89 |
| 579 | I1JEW3 | uncharacterized protein | 6 | 0.88 | 47.3 | 6.7 |
| 580 | K7MV36 | uncharacterized protein | 26 | 0.88 | 568.2 | 5.77 |
| 581 | I1MJI9 | citrate synthase | 16 | 0.88 | 52.5 | 8.52 |
| 582 | C6T7J7 | uncharacterized protein | 6 | 0.87 | 46 | 5.74 |
| 583 | I1LRA7 | carboxypeptidase | 8 | 0.87 | 51 | 5.11 |
| 584 | C6TCH7 | uncharacterized protein | 4 | 0.87 | 29 | 9.57 |
| 585 | I1MPF6 | uncharacterized protein | 8 | 0.87 | 40.4 | 5.18 |
| 586 | I1M329 | uncharacterized protein | 3 | 0.87 | 63.5 | 5.18 |
| 587 | C6K8D0 | trypsin inhibitor 26 kda isoform | 8 | 0.87 | 25.9 | 7.83 |
| 588 | I1L0W9 | uncharacterized protein | 9 | 0.87 | 85.2 | 8.36 |
| 589 | C6SY19 | uncharacterized protein | 5 | 0.87 | 20.1 | 9.69 |
| 590 | I1KQ25 | frigida-like protein | 5 | 0.87 | 57.6 | 9.13 |
| 591 | C6THI7 | uncharacterized protein | 3 | 0.87 | 31.3 | 8.96 |
| 592 | I1LI58 | phospholipase d | 4 | 0.87 | 98.4 | 6.56 |
| 593 | I1L5L0 | uncharacterized protein | 4 | 0.86 | 40.1 | 9.07 |
| 594 | I1K7J4 | tubulin beta chain | 27 | 0.86 | 50.4 | 4.76 |
| 595 | I1M292 | uncharacterized protein | 4 | 0.86 | 25.7 | 8.55 |
| 596 | I1NA37 | uncharacterized protein | 4 | 0.86 | 21 | 6.45 |
| 597 | I1LGG2 | glutathione peroxidase | 8 | 0.86 | 18.5 | 5.88 |
| 598 | I1LCG9 | uncharacterized protein | 3 | 0.86 | 65.5 | 6.48 |
| 599 | A0A0R4J2L6 | uncharacterized protein | 33 | 0.86 | 121.3 | 5.78 |
| 600 | C6TH00 | uncharacterized protein | 2 | 0.86 | 36.6 | 8.54 |
| 601 | K7KWT9 | uncharacterized protein | 8 | 0.86 | 44.9 | 7.82 |
| 602 | I1KM07 | uncharacterized protein | 12 | 0.86 | 60 | 8.93 |
| 603 | P10743 | stem 31 kda glycoprotein | 21 | 0.85 | 29.3 | 5.84 |
| 604 | I1JR56 | uncharacterized protein | 6 | 0.85 | 19.5 | 6.14 |
| 605 | I1M860 | uncharacterized protein | 5 | 0.85 | 37.8 | 5.18 |
| 606 | I1N0U1 | uncharacterized protein | 11 | 0.85 | 50.8 | 8.45 |
| 607 | A0A0R0IJV1 | uncharacterized protein | 7 | 0.84 | 36.9 | 8.39 |
| 608 | I1KXX2 | membrin | 3 | 0.84 | 25.9 | 9.54 |
| 609 | I1MAX9 | uncharacterized protein | 5 | 0.84 | 46 | 5.9 |
| 610 | I1NGB9 | uncharacterized protein | 5 | 0.84 | 38.6 | 5.41 |
| 611 | I1M2V0 | uncharacterized protein | 3 | 0.84 | 106.3 | 8.61 |
| 612 | I1M5T2 | phospholipase d | 24 | 0.84 | 92 | 5.9 |
| 613 | I1M6L4 | uncharacterized protein | 6 | 0.84 | 53.2 | 4.71 |
| 614 | I1L3P5 | eukaryotic translation initiation factor 6 | 4 | 0.84 | 26.5 | 4.63 |
| 615 | I1LIK9 | uncharacterized protein | 3 | 0.84 | 46.3 | 5.42 |
| 616 | I1JMQ2 | uncharacterized protein | 3 | 0.83 | 33.8 | 6.56 |
| 617 | I1MHA4 | DNA-directed rna polymerase subunit beta | 11 | 0.83 | 135.2 | 7.35 |
| 618 | I1L1W0 | uncharacterized protein | 9 | 0.83 | 76.6 | 5.43 |
| 619 | C6TB56 | uncharacterized protein | 7 | 0.83 | 38.6 | 6.75 |
| 620 | I1NEU4 | uncharacterized protein | 12 | 0.83 | 56 | 6.29 |
| 621 | I1JRM3 | uncharacterized protein | 2 | 0.83 | 82.5 | 5.23 |
| 622 | I1NEZ7 | lon protease homolog | 13 | 0.82 | 109.1 | 5.58 |
| 623 | I1K2H3 | uncharacterized protein | 6 | 0.82 | 120.9 | 5.47 |
| 624 | B3TDK9 | lipoxygenase | 43 | 0.82 | 96.3 | 6.34 |
| 625 | I1N0T6 | tryptophan synthase | 7 | 0.82 | 51.2 | 6.45 |
| 626 | A0A0R4J4C4 | uncharacterized protein | 9 | 0.82 | 31.7 | 6.62 |
| 627 | C6SZP7 | uncharacterized protein | 2 | 0.82 | 11 | 9.43 |
| 628 | I1LFD7 | uncharacterized protein | 14 | 0.82 | 134.9 | 5.35 |
| 629 | I1MSP4 | plasma membrane atpase | 16 | 0.82 | 104.8 | 6.41 |
| 630 | K7L6U4 | uncharacterized protein | 13 | 0.82 | 42 | 9.15 |
| 631 | C6T770 | uncharacterized protein | 4 | 0.81 | 37.2 | 8.44 |
| 632 | I1M4G0 | carboxypeptidase | 16 | 0.81 | 51 | 4.82 |
| 633 | I1K2Y0 | tubulin beta chain | 26 | 0.81 | 49.7 | 4.82 |
| 634 | I1JFN3 | uncharacterized protein | 3 | 0.81 | 13.6 | 4.66 |
| 635 | C6T7H9 | uncharacterized protein | 3 | 0.81 | 34.7 | 9.42 |
| 636 | A0A0R0IW79 | uncharacterized protein | 22 | 0.81 | 146.2 | 5.57 |
| 637 | C6SXC9 | prefoldin subunit 4 | 3 | 0.81 | 14.7 | 4.41 |
| 638 | I1NF53 | dihydroorotate dehydrogenase (quinone) | 6 | 0.8 | 48.2 | 9.14 |
| 639 | K7LAK0 | uncharacterized protein | 11 | 0.8 | 197.9 | 7.07 |
| 640 | I1LWJ8 | uncharacterized protein | 3 | 0.8 | 85.4 | 8.12 |
| 641 | I1L7C9 | uncharacterized protein | 2 | 0.8 | 53.6 | 8.42 |
| 642 | G0T440 | purple acid phosphatase | 15 | 0.8 | 70.3 | 5.93 |
| 643 | I1KXJ9 | eukaryotic translation initiation factor 3 subunit g | 8 | 0.8 | 32.1 | 8.32 |
| 644 | K7LB13 | uncharacterized protein | 2 | 0.8 | 40.4 | 6.95 |
| 645 | K7MJZ6 | uncharacterized protein | 6 | 0.8 | 99.3 | 4.47 |
| 646 | Q9ZNZ5 | peroxidase | 5 | 0.79 | 38.5 | 8.45 |
| 647 | C6TGN8 | uncharacterized protein | 4 | 0.79 | 41 | 5.33 |
| 648 | I1LF15 | uncharacterized protein | 6 | 0.79 | 152.4 | 5.72 |
| 649 | I1M042 | uncharacterized protein | 10 | 0.79 | 61.4 | 8.75 |
| 650 | I1N9J8 | uncharacterized protein | 6 | 0.79 | 36.4 | 4.78 |
| 651 | I1LMF0 | uncharacterized protein | 8 | 0.78 | 33.8 | 5.76 |
| 652 | I1MY65 | uncharacterized protein | 12 | 0.78 | 59.9 | 8.89 |
| 653 | I1J416 | uncharacterized protein | 5 | 0.78 | 34.5 | 5.4 |
| 654 | Q8S5C1 | 4-coumarate:coa ligase isoenzyme 2 | 6 | 0.78 | 60.1 | 6.26 |
| 655 | I1KCP1 | plasma membrane atpase | 19 | 0.78 | 105.2 | 6.39 |
| 656 | I1KA91 | uncharacterized protein | 6 | 0.78 | 43.4 | 4.77 |
| 657 | I1KEW1 | uncharacterized protein | 4 | 0.78 | 40.6 | 5.28 |
| 658 | B6CAM2 | matrix metalloproteinase | 5 | 0.77 | 44 | 5.41 |
| 659 | I1MMG2 | uncharacterized protein | 7 | 0.77 | 40.8 | 5.63 |
| 660 | Q02226 | cytochrome c oxidase subunit 2 | 3 | 0.77 | 44.3 | 4.81 |
| 661 | I1M510 | uncharacterized protein | 25 | 0.77 | 149.2 | 6.38 |
| 662 | I1LWF0 | peroxidase | 8 | 0.77 | 34.9 | 8.63 |
| 663 | I1K8F4 | uncharacterized protein | 12 | 0.77 | 38.5 | 7.68 |
| 664 | I1LF85 | uncharacterized protein | 2 | 0.76 | 31.8 | 4.58 |
| 665 | I1JL83 | uncharacterized protein | 2 | 0.76 | 61.6 | 5.96 |
| 666 | C6TH50 | xyloglucan endotransglucosylase/hydrolase | 5 | 0.76 | 35 | 4.81 |
| 667 | I1KEV4 | acyl-coenzyme a oxidase | 8 | 0.76 | 75.6 | 8.69 |
| 668 | I1N675 | uncharacterized protein | 10 | 0.76 | 40.5 | 4.67 |
| 669 | K7KZU4 | uncharacterized protein | 21 | 0.76 | 107.7 | 5.15 |
| 670 | I1LK85 | uncharacterized protein | 10 | 0.76 | 84.4 | 5.87 |
| 671 | C6TB35 | uncharacterized protein | 4 | 0.76 | 31.5 | 9.1 |
| 672 | C6TLS9 | uncharacterized protein | 4 | 0.76 | 19 | 8.68 |
| 673 | C6TMK3 | cysteine proteinase inhibitor | 8 | 0.76 | 27.6 | 6.4 |
| 674 | I1JQS1 | uncharacterized protein | 17 | 0.76 | 106.1 | 5.82 |
| 675 | I1NH38 | uncharacterized protein | 6 | 0.76 | 49.2 | 6.9 |
| 676 | C6TM03 | malate dehydrogenase | 7 | 0.76 | 37.4 | 8.52 |
| 677 | I1MLH0 | uncharacterized protein | 2 | 0.75 | 63.6 | 8.28 |
| 678 | I1MB71 | fructose-bisphosphate aldolase | 21 | 0.75 | 38.3 | 7.12 |
| 679 | I1LCN1 | uncharacterized protein | 11 | 0.75 | 36.3 | 6.26 |
| 680 | I1JPW6 | uncharacterized protein | 2 | 0.75 | 36.2 | 9.52 |
| 681 | I1JAF7 | beta-adaptin-like protein | 16 | 0.75 | 99 | 4.94 |
| 682 | C6T4X8 | uncharacterized protein | 3 | 0.75 | 17.1 | 9.24 |
| 683 | K7MMK0 | uncharacterized protein | 4 | 0.75 | 42.2 | 9.05 |
| 684 | I1LZK9 | uncharacterized protein | 3 | 0.74 | 65.4 | 6.42 |
| 685 | A0A0R0K5H8 | uncharacterized protein | 36 | 0.74 | 111.2 | 6.24 |
| 686 | I1MMM3 | uncharacterized protein | 4 | 0.74 | 23.3 | 6.58 |
| 687 | I1ML36 | uncharacterized protein | 3 | 0.74 | 52.5 | 5.21 |
| 688 | C6TFM1 | uncharacterized protein | 8 | 0.73 | 40.9 | 7.13 |
| 689 | I1MB50 | uncharacterized protein | 9 | 0.73 | 28.7 | 5.65 |
| 690 | C6TIM5 | ferredoxin--nadp reductase | 8 | 0.73 | 42.2 | 8.52 |
| 691 | I1JGM7 | uncharacterized protein | 4 | 0.73 | 51 | 5.57 |
| 692 | A0A0R0I1T7 | uncharacterized protein | 6 | 0.73 | 35.6 | 6.46 |
| 693 | A8IKE5 | alanine aminotransferase 2 | 11 | 0.72 | 53.5 | 5.42 |
| 694 | I1M2Y5 | uncharacterized protein | 14 | 0.72 | 38.5 | 6.71 |
| 695 | I1K711 | uncharacterized protein | 6 | 0.72 | 40 | 4.82 |
| 696 | K7LRU6 | uncharacterized protein | 8 | 0.72 | 77.8 | 4.76 |
| 697 | I1L4W8 | uncharacterized protein | 3 | 0.72 | 30.4 | 9.53 |
| 698 | I1MHG0 | uncharacterized protein | 4 | 0.72 | 39 | 6.2 |
| 699 | I1KBA7 | uncharacterized protein | 2 | 0.72 | 113.6 | 6.4 |
| 700 | I1JRJ3 | uncharacterized protein | 4 | 0.72 | 133.9 | 5.93 |
| 701 | I1NHF4 | chorismate synthase | 8 | 0.72 | 47 | 7.63 |
| 702 | C6SVC7 | uncharacterized protein | 5 | 0.72 | 20.2 | 8.15 |
| 703 | I1KZW4 | uncharacterized protein | 11 | 0.72 | 88.9 | 5.06 |
| 704 | I1KID4 | uncharacterized protein | 4 | 0.72 | 21.6 | 8.43 |
| 705 | I1LMA5 | uncharacterized protein | 23 | 0.72 | 81.3 | 6.29 |
| 706 | I1LFG4 | uncharacterized protein | 14 | 0.71 | 80.2 | 6.12 |
| 707 | I1LD65 | dihydrolipoamide acetyltransferase component of pyruvate dehydrogenase complex | 9 | 0.71 | 48.9 | 8.84 |
| 708 | I1JTU1 | branched-chain-amino-acid aminotransferase | 8 | 0.71 | 42 | 6.76 |
| 709 | I1M6B9 | uncharacterized protein | 11 | 0.71 | 84.5 | 6.86 |
| 710 | K7KTV2 | uncharacterized protein | 2 | 0.71 | 45.4 | 9.26 |
| 711 | I1K5D3 | eukaryotic translation initiation factor 3 subunit c | 21 | 0.7 | 104.2 | 5.57 |
| 712 | I1MMC9 | uncharacterized protein | 5 | 0.7 | 23.7 | 10.33 |
| 713 | I1K7F4 | uncharacterized protein | 9 | 0.7 | 60.8 | 9.38 |
| 714 | C6TNN5 | uncharacterized protein | 8 | 0.7 | 43.4 | 5.59 |
| 715 | I1MSB3 | uncharacterized protein | 11 | 0.7 | 44.2 | 8.22 |
| 716 | I1MZF9 | uncharacterized protein | 22 | 0.7 | 81.2 | 6.21 |
| 717 | C6TLC2 | uncharacterized protein | 9 | 0.7 | 37.9 | 4.53 |
| 718 | I1JG98 | uncharacterized protein | 6 | 0.7 | 34.3 | 6.26 |
| 719 | I1KKQ1 | uncharacterized protein | 18 | 0.7 | 58.7 | 8.72 |
| 720 | I1KRI1 | uncharacterized protein | 8 | 0.7 | 54.1 | 5.88 |
| 721 | I1JKP7 | 3-phosphoshikimate 1-carboxyvinyltransferase | 8 | 0.7 | 55.8 | 6.29 |
| 722 | M1FN34 | NADH-ubiquinone oxidoreductase chain 1 | 2 | 0.7 | 35.5 | 9.32 |
| 723 | K7KQP0 | uncharacterized protein | 7 | 0.69 | 52.2 | 9.07 |
| 724 | K7MQ23 | uncharacterized protein | 7 | 0.69 | 62.2 | 6.51 |
| 725 | P49161 | cytochrome f | 4 | 0.69 | 35.3 | 8.38 |
| 726 | I1JSF3 | uncharacterized protein | 3 | 0.69 | 18.7 | 5.92 |
| 727 | I1ND31 | uncharacterized protein | 12 | 0.69 | 42.8 | 6.9 |
| 728 | I1JS33 | uncharacterized protein | 2 | 0.69 | 53.3 | 4.84 |
| 729 | I1KCF1 | uncharacterized protein | 6 | 0.69 | 44.3 | 10.33 |
| 730 | I1LYP8 | uncharacterized protein | 14 | 0.68 | 43.8 | 9.22 |
| 731 | C6TAS0 | uncharacterized protein | 3 | 0.68 | 43.3 | 5.27 |
| 732 | C6TH44 | uncharacterized protein | 6 | 0.68 | 31.8 | 9.72 |
| 733 | A0A0R0KQE9 | uncharacterized protein | 19 | 0.68 | 50.7 | 6.8 |
| 734 | I1LZR2 | uncharacterized protein | 3 | 0.68 | 23.5 | 9.1 |
| 735 | I1KXT4 | uncharacterized protein | 16 | 0.68 | 68.7 | 8.01 |
| 736 | I1MDY6 | uncharacterized protein | 2 | 0.68 | 60.6 | 9.53 |
| 737 | I1JU00 | uncharacterized protein | 3 | 0.68 | 17.9 | 6.62 |
| 738 | I1MDN4 | uncharacterized protein | 8 | 0.68 | 118.7 | 4.91 |
| 739 | K7LSD2 | uncharacterized protein | 16 | 0.68 | 102.5 | 5.14 |
| 740 | I1N727 | uncharacterized protein | 10 | 0.68 | 39.1 | 6.46 |
| 741 | C6TIY9 | uncharacterized protein | 3 | 0.68 | 44.9 | 6.23 |
| 742 | I1N5R9 | formate dehydrogenase | 15 | 0.68 | 41.3 | 6.87 |
| 743 | I1LDX0 | uncharacterized protein | 12 | 0.68 | 68.4 | 6.01 |
| 744 | I1L1Q8 | uncharacterized protein | 23 | 0.67 | 54.4 | 5.8 |
| 745 | I1JET4 | uncharacterized protein | 13 | 0.67 | 60.2 | 8.96 |
| 746 | I1K7A1 | dolichyl-diphosphooligosaccharide--protein glycosyltransferase subunit 1 | 14 | 0.67 | 68.4 | 6.62 |
| 747 | I1JY87 | 4-alpha-glucanotransferase | 8 | 0.67 | 63.3 | 5.55 |
| 748 | C6TKH1 | uncharacterized protein | 4 | 0.67 | 34.3 | 6.46 |
| 749 | I1LPE4 | uncharacterized protein | 17 | 0.67 | 56.2 | 8.33 |
| 750 | I1LGL7 | uncharacterized protein | 7 | 0.67 | 16.5 | 4.73 |
| 751 | A0A0R0E4D1 | uncharacterized protein | 3 | 0.66 | 78.1 | 9.65 |
| 752 | A0A0R0IXP9 | uncharacterized protein | 5 | 0.66 | 26.3 | 9.4 |
| 753 | I1JV68 | uncharacterized protein | 18 | 0.66 | 56.8 | 5.7 |
| 754 | C6SYM8 | uncharacterized protein | 2 | 0.66 | 21.9 | 4.93 |
| 755 | I1MWP7 | cytochrome b-c1 complex subunit 7 | 5 | 0.66 | 14.5 | 9.58 |
| 756 | I1KS01 | lipoxygenase | 15 | 0.65 | 103.6 | 7.96 |
| 757 | I1KZ13 | uncharacterized protein | 17 | 0.65 | 58.9 | 6.49 |
| 758 | I1KW16 | uncharacterized protein | 3 | 0.65 | 9.3 | 9.21 |
| 759 | I1KWF3 | uncharacterized protein | 4 | 0.65 | 73.4 | 6.68 |
| 760 | I1KSB7 | uncharacterized protein | 3 | 0.65 | 53 | 4.82 |
| 761 | K7MCF4 | uncharacterized protein | 5 | 0.65 | 113.2 | 5.8 |
| 762 | I1MQT0 | uncharacterized protein | 13 | 0.65 | 99.6 | 9.14 |
| 763 | A0A1V0CIA1 | 2-methyl-6-phytyl-1,4-benzoquinol methyltransferase | 5 | 0.65 | 38.5 | 9.07 |
| 764 | C6TBL0 | Mg-protoporphyrin ix chelatase | 8 | 0.64 | 45.8 | 5.6 |
| 765 | I1JNC7 | uncharacterized protein | 17 | 0.64 | 108.5 | 5.61 |
| 766 | A0A0R4J2K3 | uncharacterized protein | 18 | 0.64 | 51.7 | 7.64 |
| 767 | A0A0R0LCZ4 | uncharacterized protein | 10 | 0.64 | 60.7 | 6.61 |
| 768 | I1LL87 | uncharacterized protein | 3 | 0.64 | 19.3 | 4.84 |
| 769 | I1NGN1 | uncharacterized protein | 9 | 0.63 | 37.3 | 8.63 |
| 770 | I1M4J9 | uncharacterized protein | 6 | 0.63 | 94.5 | 4.56 |
| 771 | I1KA79 | uncharacterized protein | 7 | 0.63 | 34.7 | 8.57 |
| 772 | I1K4M6 | uncharacterized protein | 30 | 0.63 | 229.2 | 5.24 |
| 773 | I1KPN2 | uncharacterized protein | 27 | 0.63 | 96.3 | 4.67 |
| 774 | I1J5Q3 | uncharacterized protein | 13 | 0.63 | 68.9 | 6.63 |
| 775 | A0A0R0H1W3 | uncharacterized protein | 11 | 0.63 | 96.9 | 5.92 |
| 776 | I1LBQ2 | uncharacterized protein | 7 | 0.63 | 108.4 | 5.25 |
| 777 | C6SY72 | uncharacterized protein | 2 | 0.63 | 14.4 | 10.23 |
| 778 | A0A0R0KR16 | uncharacterized protein | 5 | 0.63 | 13.8 | 9.61 |
| 779 | K7MFA8 | uncharacterized protein | 4 | 0.62 | 76.8 | 6.99 |
| 780 | I1JBG1 | uncharacterized protein | 3 | 0.62 | 35.4 | 7.95 |
| 781 | I1LHT2 | uncharacterized protein | 3 | 0.62 | 70.8 | 8.49 |
| 782 | I1KAK6 | uncharacterized protein | 4 | 0.62 | 27.4 | 6.02 |
| 783 | I1LE27 | uncharacterized protein | 5 | 0.62 | 93.3 | 8.66 |
| 784 | I1JYL3 | uncharacterized protein | 8 | 0.62 | 33.9 | 7.09 |
| 785 | I1K4S0 | uncharacterized protein | 12 | 0.62 | 27.5 | 6.09 |
| 786 | I1MDY7 | uncharacterized protein | 4 | 0.61 | 59 | 5.76 |
| 787 | I1JHQ7 | uncharacterized protein | 9 | 0.61 | 59.9 | 7.6 |
| 788 | A0A0R4J305 | uncharacterized protein | 18 | 0.61 | 44.6 | 8.25 |
| 789 | I1L5K6 | carboxypeptidase | 7 | 0.61 | 55.6 | 6.39 |
| 790 | I1KS18 | uncharacterized protein | 4 | 0.61 | 25.3 | 6.05 |
| 791 | I1MCM4 | uncharacterized protein | 4 | 0.61 | 48.1 | 4.82 |
| 792 | A0A0R0JBP2 | uncharacterized protein | 8 | 0.61 | 24.1 | 5.46 |
| 793 | I1LTT3 | uncharacterized protein | 7 | 0.61 | 11.2 | 4.29 |
| 794 | A0A0R0ESN9 | uncharacterized protein | 10 | 0.61 | 76.4 | 5.02 |
| 795 | I1ML66 | glyceraldehyde-3-phosphate dehydrogenase | 10 | 0.61 | 43.2 | 7.61 |
| 796 | K7M1P8 | uncharacterized protein | 3 | 0.61 | 107.6 | 5.64 |
| 797 | C6TJ77 | uncharacterized protein | 6 | 0.6 | 48.9 | 5.28 |
| 798 | I1MGW1 | uncharacterized protein | 6 | 0.6 | 80.4 | 6.14 |
| 799 | A0A0R0HYB1 | uncharacterized protein | 9 | 0.6 | 51.6 | 8.58 |
| 800 | I1M2Z5 | uncharacterized protein | 11 | 0.6 | 24 | 5.87 |
| 801 | A0A0R0FAJ6 | uncharacterized protein | 20 | 0.6 | 62.6 | 6.5 |
| 802 | A0A0R4J594 | peroxidase | 7 | 0.6 | 34.8 | 8.14 |
| 803 | A0A0R0H141 | nascent polypeptide-associated complex subunit beta | 10 | 0.6 | 17.5 | 7.91 |
| 804 | K7M9L4 | uncharacterized protein | 21 | 0.6 | 126.7 | 5.28 |
| 805 | K7MQM3 | uncharacterized protein | 9 | 0.6 | 46.5 | 4.55 |
| 806 | K7L8U3 | uncharacterized protein | 3 | 0.59 | 81.5 | 5 |
| 807 | I1NH15 | uncharacterized protein | 13 | 0.59 | 147.9 | 5.2 |
| 808 | I1MJE1 | uncharacterized protein | 18 | 0.59 | 52.8 | 6 |
| 809 | C6TDD0 | uncharacterized protein | 3 | 0.59 | 39.7 | 5.15 |
| 810 | I1JV39 | glucose-6-phosphate isomerase | 23 | 0.59 | 67.3 | 5.66 |
| 811 | I1KEW2 | uncharacterized protein | 5 | 0.59 | 40.6 | 5.66 |
| 812 | I1MKT8 | glycosyltransferase | 8 | 0.58 | 54.4 | 6.36 |
| 813 | I1M6M0 | uncharacterized protein | 16 | 0.58 | 65.3 | 5.14 |
| 814 | I1KQG0 | t-complex protein 1 subunit delta | 23 | 0.58 | 57.6 | 6.85 |
| 815 | I1MFN0 | uncharacterized protein | 11 | 0.58 | 45.8 | 5.84 |
| 816 | C6THV7 | NADH-cytochrome b5 reductase | 9 | 0.58 | 35.6 | 9.06 |
| 817 | A0A0R0GL56 | uncharacterized protein | 3 | 0.58 | 87 | 5.57 |
| 818 | I1KZH4 | uncharacterized protein | 2 | 0.58 | 225.6 | 9.15 |
| 819 | I1KQC4 | uncharacterized protein | 5 | 0.58 | 16.2 | 9.69 |
| 820 | I1NDE9 | uncharacterized protein | 9 | 0.58 | 54.4 | 4.64 |
| 821 | I1MLW9 | uncharacterized protein | 16 | 0.58 | 34.6 | 6.2 |
| 822 | K7MQT5 | uncharacterized protein | 18 | 0.58 | 63 | 5.86 |
| 823 | I1KSB3 | uncharacterized protein | 4 | 0.58 | 80.3 | 7.6 |
| 824 | A0A0R4J5N5 | uncharacterized protein | 13 | 0.58 | 44.5 | 5.32 |
| 825 | C6SZT9 | uncharacterized protein | 8 | 0.58 | 25.7 | 5.59 |
| 826 | I1M2Y6 | uncharacterized protein | 8 | 0.58 | 37.8 | 5.59 |
| 827 | Q9LLQ8 | carboxyl transferase alpha subunit | 16 | 0.57 | 76.7 | 8.85 |
| 828 | C6SWZ6 | uncharacterized protein | 7 | 0.57 | 26.2 | 10.24 |
| 829 | I1JYL5 | uncharacterized protein | 5 | 0.57 | 16 | 5.48 |
| 830 | C6T9H7 | uncharacterized protein | 11 | 0.57 | 47.6 | 5.66 |
| 831 | I1M599 | lipoxygenase | 44 | 0.57 | 92.7 | 6.52 |
| 832 | A0A0R4J4M5 | uncharacterized protein | 5 | 0.57 | 36.7 | 6.56 |
| 833 | C6SWH3 | uncharacterized protein | 3 | 0.57 | 15.1 | 7.92 |
| 834 | C6TMI1 | serine/threonine-protein phosphatase | 7 | 0.57 | 36.9 | 5.49 |
| 835 | C6T2Y2 | uncharacterized protein | 3 | 0.57 | 18.6 | 4.95 |
| 836 | K7L5L6 | uncharacterized protein | 19 | 0.57 | 411.3 | 4.9 |
| 837 | K7LSQ6 | uncharacterized protein | 2 | 0.57 | 117.4 | 6.12 |
| 838 | I1JXQ5 | alpha-mannosidase | 17 | 0.57 | 114.2 | 6.3 |
| 839 | I1LUJ6 | uncharacterized protein | 8 | 0.57 | 37.1 | 9.64 |
| 840 | C6T1N4 | uncharacterized protein | 3 | 0.56 | 17.5 | 4.85 |
| 841 | I1L5H6 | uncharacterized protein | 9 | 0.56 | 92.6 | 5.05 |
| 842 | I1JJ04 | reticulon-like protein | 3 | 0.56 | 27.7 | 9.32 |
| 843 | I1MNL7 | carboxypeptidase | 6 | 0.56 | 55.5 | 5.12 |
| 844 | C6TGW1 | reticulon-like protein | 6 | 0.56 | 28.5 | 8.97 |
| 845 | C6TD38 | uncharacterized protein | 5 | 0.56 | 22 | 6.43 |
| 846 | I1LBK9 | uncharacterized protein | 4 | 0.56 | 19.7 | 5.1 |
| 847 | C6T8S2 | uncharacterized protein | 5 | 0.56 | 32.5 | 5.15 |
| 848 | K7LS61 | uncharacterized protein | 3 | 0.56 | 32 | 9.21 |
| 849 | I1MUR2 | uncharacterized protein | 15 | 0.56 | 36.7 | 5.95 |
| 850 | I1JEV7 | uncharacterized protein | 4 | 0.56 | 29.8 | 4.98 |
| 851 | C6T129 | uncharacterized protein | 6 | 0.56 | 17.2 | 6.14 |
| 852 | I1M281 | uncharacterized protein | 5 | 0.56 | 66.2 | 5.18 |
| 853 | I1KY39 | citrate synthase | 13 | 0.56 | 56.4 | 9.22 |
| 854 | I1KYS4 | uncharacterized protein | 19 | 0.55 | 203.5 | 6.82 |
| 855 | K7LA80 | uncharacterized protein | 11 | 0.55 | 41.3 | 8.15 |
| 856 | I1K0K7 | uncharacterized protein | 7 | 0.55 | 63.4 | 5.79 |
| 857 | A0A0R4J2P9 | cysteine synthase | 14 | 0.55 | 41.6 | 6.91 |
| 858 | K7MZ43 | glycosyltransferase | 2 | 0.55 | 61 | 6.07 |
| 859 | I1JX24 | uncharacterized protein | 8 | 0.55 | 45.8 | 6.5 |
| 860 | C6T2G1 | uncharacterized protein | 3 | 0.55 | 26.4 | 5.42 |
| 861 | K7LKF8 | histone h2b | 2 | 0.55 | 22.6 | 10 |
| 862 | C6SVJ8 | uncharacterized protein | 4 | 0.55 | 15.9 | 5.58 |
| 863 | I1L977 | uncharacterized protein | 2 | 0.55 | 35.6 | 9.66 |
| 864 | I1LJA4 | uncharacterized protein | 15 | 0.54 | 60.8 | 9.07 |
| 865 | C6SVW8 | uncharacterized protein | 2 | 0.54 | 13 | 5.27 |
| 866 | I1KLQ4 | uncharacterized protein | 7 | 0.54 | 120.1 | 7.24 |
| 867 | I1LLR5 | uncharacterized protein | 7 | 0.54 | 27.7 | 5.55 |
| 868 | I1MT23 | uncharacterized protein | 24 | 0.54 | 59.8 | 6.69 |
| 869 | I1NCA0 | wound-induced protein | 4 | 0.54 | 22.5 | 5.29 |
| 870 | V6CKR0 | expansin | 5 | 0.54 | 27.5 | 9.16 |
| 871 | I1JTB8 | glucose-6-phosphate isomerase | 19 | 0.54 | 62.6 | 5.9 |
| 872 | Q2PMT2 | 30s ribosomal protein s2 | 2 | 0.54 | 26.9 | 9.37 |
| 873 | I1L8G3 | succinate--coa ligase | 12 | 0.54 | 45.3 | 5.89 |
| 874 | C6T899 | uncharacterized protein | 10 | 0.54 | 55.7 | 5.94 |
| 875 | I1LKK2 | uncharacterized protein | 2 | 0.53 | 46.4 | 5.49 |
| 876 | I1LMQ7 | uncharacterized protein | 4 | 0.53 | 89.5 | 6.81 |
| 877 | I1LWP5 | uncharacterized protein | 15 | 0.52 | 117.4 | 5.62 |
| 878 | C6TBK9 | uncharacterized protein | 5 | 0.52 | 33.9 | 8.51 |
| 879 | C6T0P8 | uncharacterized protein | 3 | 0.52 | 15.1 | 6.59 |
| 880 | I1KQ42 | uncharacterized protein | 9 | 0.52 | 44.6 | 6.04 |
| 881 | I1JHP9 | uncharacterized protein | 8 | 0.52 | 22.7 | 8.71 |
| 882 | I1J8Y0 | uncharacterized protein | 21 | 0.52 | 73 | 5.46 |
| 883 | I1J6K4 | uncharacterized protein | 17 | 0.51 | 102.9 | 6.16 |
| 884 | A0A0R0KKK6 | NADH-cytochrome b5 reductase | 8 | 0.51 | 30.8 | 8.23 |
| 885 | I1K778 | coatomer subunit gamma | 33 | 0.51 | 98.2 | 5.07 |
| 886 | I1K8C1 | uncharacterized protein | 6 | 0.51 | 23.5 | 9.53 |
| 887 | A0A0R0KL81 | uncharacterized protein | 4 | 0.51 | 80.9 | 6.65 |
| 888 | I1LEQ8 | fructose-bisphosphate aldolase | 11 | 0.51 | 42.5 | 8.16 |
| 889 | I1MZR2 | uncharacterized protein | 14 | 0.51 | 127.4 | 5.13 |
| 890 | I1MBZ3 | uncharacterized protein | 12 | 0.51 | 98.4 | 5.3 |
| 891 | C6TDW7 | uncharacterized protein | 4 | 0.51 | 25.5 | 8.05 |
| 892 | K7MIH4 | uncharacterized protein | 7 | 0.51 | 24.2 | 4.54 |
| 893 | I1MXZ6 | thioredoxin | 8 | 0.5 | 13.4 | 4.76 |
| 894 | K7LC34 | calcium-transporting atpase | 7 | 0.5 | 117.8 | 7.87 |
| 895 | I1MS26 | uncharacterized protein | 25 | 0.5 | 115.6 | 5.54 |
| 896 | A0A0R0K2F5 | uncharacterized protein | 6 | 0.5 | 34.6 | 8.75 |
| 897 | I1LHJ6 | uncharacterized protein | 10 | 0.5 | 50.1 | 4.41 |
| 898 | I1M5U8 | uncharacterized protein | 31 | 0.5 | 275.7 | 8.84 |
| 899 | I1KYQ4 | glycosyltransferase | 3 | 0.5 | 54.2 | 5.62 |
| 900 | I1KQE2 | uncharacterized protein | 5 | 0.5 | 34.6 | 7.68 |
| 901 | K7MVK8 | uncharacterized protein | 11 | 0.5 | 31.5 | 5.9 |
| 902 | I1MTJ0 | uncharacterized protein | 2 | 0.5 | 20.1 | 9.3 |
| 903 | C6TEU3 | 40s ribosomal protein s6 | 6 | 0.5 | 28.4 | 10.77 |
| 904 | I1KW20 | uncharacterized protein | 6 | 0.49 | 30.5 | 7.96 |
| 905 | I1LVL8 | uncharacterized protein | 5 | 0.49 | 46.7 | 5.76 |
| 906 | I1JY81 | uncharacterized protein | 8 | 0.49 | 41.2 | 8.62 |
| 907 | I1LJA7 | coatomer subunit delta | 14 | 0.49 | 57.9 | 5.5 |
| 908 | C6TKS1 | uncharacterized protein | 13 | 0.49 | 29.6 | 5.79 |
| 909 | I1LWC7 | uncharacterized protein | 16 | 0.49 | 72.7 | 5.09 |
| 910 | A5JVZ7 | superoxide dismutase | 8 | 0.49 | 26.7 | 8.56 |
| 911 | D2D5G4 | methyltransferase | 12 | 0.48 | 40.3 | 6.28 |
| 912 | C6TGF5 | uncharacterized protein | 2 | 0.48 | 31.8 | 7.6 |
| 913 | I1N7B1 | uncharacterized protein | 5 | 0.48 | 17.4 | 7.9 |
| 914 | A0A0R0G797 | uncharacterized protein | 11 | 0.48 | 43.2 | 6.48 |
| 915 | A0A0R0KHB4 | delta-aminolevulinic acid dehydratase | 8 | 0.48 | 45.2 | 6.35 |
| 916 | K7MLF2 | uncharacterized protein | 9 | 0.48 | 132.6 | 6.19 |
| 917 | A0A0R0GYV0 | uncharacterized protein | 2 | 0.48 | 20.4 | 10.16 |
| 918 | I1MBI6 | uncharacterized protein | 16 | 0.47 | 109.8 | 5.88 |
| 919 | A0A0R0FJI5 | uncharacterized protein | 21 | 0.47 | 152.8 | 6.07 |
| 920 | I1LNM2 | NADH dehydrogenase subunit 9 | 6 | 0.47 | 22.6 | 7.71 |
| 921 | A0A0R4J2U8 | uncharacterized protein | 26 | 0.47 | 78.9 | 6.4 |
| 922 | I1KVR6 | coatomer subunit beta | 29 | 0.46 | 105.9 | 5.57 |
| 923 | K7M1S4 | uncharacterized protein | 2 | 0.46 | 30.6 | 9.23 |
| 924 | I1J4S3 | uncharacterized protein | 5 | 0.46 | 39.6 | 4.58 |
| 925 | K7MIV6 | uncharacterized protein | 9 | 0.46 | 23.6 | 5.9 |
| 926 | A0A0R4J4D2 | uncharacterized protein | 15 | 0.46 | 39.1 | 5.61 |
| 927 | Q2PMV0 | ATP synthase subunit beta | 22 | 0.46 | 53.7 | 5.29 |
| 928 | Q8W198 | phosphate transporter | 12 | 0.46 | 36.3 | 9.17 |
| 929 | I1KLV9 | eukaryotic translation initiation factor 2a | 6 | 0.46 | 56.7 | 8.91 |
| 930 | I1JFY8 | glycerol-3-phosphate dehydrogenase | 11 | 0.46 | 68.7 | 8.67 |
| 931 | I1MRV6 | uncharacterized protein | 18 | 0.46 | 52.9 | 6.9 |
| 932 | C6SVC4 | uncharacterized protein | 8 | 0.46 | 27.6 | 5.67 |
| 933 | I1K146 | uncharacterized protein | 11 | 0.45 | 33.9 | 6.72 |
| 934 | A0A0R0K7P8 | uncharacterized protein | 20 | 0.45 | 110.4 | 5.49 |
| 935 | I1MQL3 | uncharacterized protein | 17 | 0.45 | 116.4 | 9.39 |
| 936 | K7M9T4 | uncharacterized protein | 5 | 0.45 | 66.2 | 9.28 |
| 937 | I1LI96 | phosphoserine aminotransferase | 12 | 0.45 | 45 | 8.04 |
| 938 | I1JJF7 | uncharacterized protein | 16 | 0.45 | 81.8 | 5.62 |
| 939 | I1JR26 | uncharacterized protein | 14 | 0.45 | 112.9 | 7.85 |
| 940 | I1M2M2 | uncharacterized protein | 15 | 0.45 | 90.9 | 6.54 |
| 941 | K7KPQ8 | uncharacterized protein | 7 | 0.45 | 48.9 | 8.42 |
| 942 | I1K246 | uncharacterized protein | 9 | 0.45 | 62.1 | 5.6 |
| 943 | I1LPA6 | uncharacterized protein | 4 | 0.45 | 38.8 | 8.54 |
| 944 | I1NES7 | uncharacterized protein | 17 | 0.45 | 83.1 | 6.89 |
| 945 | A0A0R0IU60 | uncharacterized protein | 22 | 0.45 | 62.8 | 5.85 |
| 946 | I1LQ43 | adenylyl cyclase-associated protein | 8 | 0.45 | 50.6 | 6.57 |
| 947 | I1MJC7 | phosphoglycerate kinase | 22 | 0.45 | 42.4 | 5.96 |
| 948 | I1LJP3 | uncharacterized protein | 14 | 0.44 | 35.4 | 4.96 |
| 949 | C6T5F7 | uncharacterized protein | 2 | 0.44 | 21.2 | 9.45 |
| 950 | I1JPY7 | uncharacterized protein | 22 | 0.44 | 99 | 5.22 |
| 951 | C6TKA3 | NADH-cytochrome b5 reductase | 10 | 0.44 | 35.1 | 8.63 |
| 952 | K7LZ48 | uncharacterized protein | 12 | 0.44 | 88.6 | 6.03 |
| 953 | I1MRA7 | amine oxidase | 34 | 0.44 | 75.8 | 6.15 |
| 954 | I1MG28 | uncharacterized protein | 10 | 0.44 | 72 | 6.17 |
| 955 | I1M2U5 | uncharacterized protein | 6 | 0.44 | 39.9 | 5.63 |
| 956 | A0A0R4J2H1 | terpene cyclase/mutase family member | 10 | 0.44 | 86.3 | 6.32 |
| 957 | A0A0R0IPU2 | uncharacterized protein | 8 | 0.44 | 59.4 | 7 |
| 958 | C6TMN0 | ATP-dependent clp protease proteolytic subunit | 5 | 0.44 | 26 | 7.76 |
| 959 | K7KHE6 | uncharacterized protein | 5 | 0.43 | 34 | 9.61 |
| 960 | C6T2H1 | uncharacterized protein | 9 | 0.43 | 21.7 | 6.43 |
| 961 | C6T2C1 | uncharacterized protein | 4 | 0.43 | 17.1 | 6.9 |
| 962 | I1M2J1 | uncharacterized protein | 8 | 0.43 | 32.3 | 6.37 |
| 963 | I1J4J6 | malic enzyme | 25 | 0.43 | 65 | 5.83 |
| 964 | O48561 | catalase-4 | 18 | 0.43 | 56.7 | 6.8 |
| 965 | I1JW13 | uncharacterized protein | 7 | 0.43 | 30.4 | 7.95 |
| 966 | I1NDD4 | uncharacterized protein | 9 | 0.43 | 41.3 | 6.21 |
| 967 | I1JKQ4 | arginase | 13 | 0.43 | 38.7 | 6.04 |
| 968 | I1JRF8 | glutathione synthetase | 14 | 0.43 | 61.2 | 6.22 |
| 969 | I1MR46 | uncharacterized protein | 8 | 0.42 | 62 | 9.48 |
| 970 | I1L2C2 | transmembrane 9 superfamily member | 7 | 0.42 | 74.9 | 5.94 |
| 971 | I1J5B0 | uncharacterized protein | 15 | 0.42 | 95.3 | 6.22 |
| 972 | I1LLM7 | uncharacterized protein | 20 | 0.42 | 65.2 | 6.16 |
| 973 | I1KFE9 | uncharacterized protein | 10 | 0.42 | 60.1 | 6.93 |
| 974 | Q06197 | isocitrate dehydrogenase | 20 | 0.42 | 46 | 5.87 |
| 975 | C6TAJ6 | carboxypeptidase | 3 | 0.42 | 54.4 | 8.66 |
| 976 | I1KG47 | d-3-phosphoglycerate dehydrogenase | 12 | 0.42 | 66.2 | 7.61 |
| 977 | A0A0R0GMF6 | uncharacterized protein | 3 | 0.42 | 32.8 | 5.79 |
| 978 | I1KXG9 | uncharacterized protein | 22 | 0.41 | 58.4 | 7.19 |
| 979 | A0A0R0E5J0 | glycine cleavage system h protein | 2 | 0.41 | 17 | 4.76 |
| 980 | K7M084 | uncharacterized protein | 3 | 0.41 | 16.6 | 7.71 |
| 981 | A0A0R0I6U8 | uncharacterized protein | 14 | 0.41 | 62.2 | 6.01 |
| 982 | O48560 | catalase-3 | 17 | 0.41 | 56.9 | 6.77 |
| 983 | K7N525 | uncharacterized protein | 7 | 0.41 | 68.6 | 6.34 |
| 984 | I1K7H0 | uncharacterized protein | 20 | 0.41 | 55.6 | 8.93 |
| 985 | I1KZ41 | glycosyltransferase | 5 | 0.41 | 54.3 | 6.27 |
| 986 | I1MM08 | malic enzyme | 23 | 0.4 | 65.2 | 5.75 |
| 987 | I1KQ93 | uncharacterized protein | 29 | 0.4 | 63.4 | 5.35 |
| 988 | I1LQ06 | coatomer subunit delta | 12 | 0.4 | 58.4 | 5.42 |
| 989 | I1KDF1 | uncharacterized protein | 2 | 0.4 | 32.1 | 8.83 |
| 990 | I1N1W7 | uncharacterized protein | 19 | 0.4 | 58.8 | 6.49 |
| 991 | K7LP97 | uncharacterized protein | 6 | 0.4 | 16.9 | 7.82 |
| 992 | K7L8T1 | uncharacterized protein | 30 | 0.4 | 544.6 | 5.9 |
| 993 | O22378 | metallothionein-ii protein | 2 | 0.4 | 8.3 | 7.36 |
| 994 | I1M9F7 | aspartate aminotransferase | 19 | 0.4 | 50.6 | 7.71 |
| 995 | I1JZ72 | uncharacterized protein | 7 | 0.4 | 43.2 | 4.98 |
| 996 | I1L932 | glycosyltransferase | 8 | 0.4 | 54.4 | 5.29 |
| 997 | I1L9H8 | uncharacterized protein | 7 | 0.4 | 16.6 | 6.2 |
| 998 | A0A0R0JD84 | glycine cleavage system h protein | 2 | 0.39 | 20 | 4.84 |
| 999 | K7LZH1 | uncharacterized protein | 30 | 0.39 | 69.2 | 5.13 |
| 1000 | C6TMC5 | uncharacterized protein | 3 | 0.39 | 23.7 | 9.16 |
| 1001 | A0A0R4J4T0 | uncharacterized protein | 16 | 0.39 | 80.1 | 5.24 |
| 1002 | I1N933 | adenylosuccinate synthetase | 10 | 0.39 | 52.6 | 7.55 |
| 1003 | C6SXC3 | uncharacterized protein | 3 | 0.39 | 12.8 | 10.58 |
| 1004 | C6SY14 | uncharacterized protein | 5 | 0.39 | 11 | 9.35 |
| 1005 | Q2I0H4 | glyceraldehyde-3-phosphate dehydrogenase | 21 | 0.39 | 36.7 | 6.72 |
| 1006 | O64458 | 34 kda maturing seed protein | 3 | 0.39 | 42.7 | 5.63 |
| 1007 | A0A0R4J656 | uncharacterized protein | 33 | 0.38 | 61 | 6.38 |
| 1008 | K7L949 | uncharacterized protein | 2 | 0.38 | 14.2 | 6.11 |
| 1009 | C6SY64 | proteasome subunit beta type | 9 | 0.38 | 24.5 | 6.81 |
| 1010 | I1K1C9 | uncharacterized protein | 11 | 0.38 | 38.4 | 5.19 |
| 1011 | Q09WE7 | UDP-sugar pyrophosphorylase 1 | 15 | 0.38 | 66.1 | 5.7 |
| 1012 | C6TJ17 | uncharacterized protein | 11 | 0.38 | 33.6 | 6.46 |
| 1013 | K7MHB7 | uncharacterized protein | 12 | 0.37 | 54.8 | 6.4 |
| 1014 | I1LYC1 | uncharacterized protein | 34 | 0.37 | 197.6 | 5.48 |
| 1015 | I1M8K6 | uncharacterized protein | 10 | 0.37 | 35.2 | 6.11 |
| 1016 | I1LH18 | uncharacterized protein | 12 | 0.37 | 48 | 5.28 |
| 1017 | I1JAD2 | uncharacterized protein | 12 | 0.37 | 74 | 7.11 |
| 1018 | I1LY51 | uncharacterized protein | 15 | 0.37 | 44.1 | 8.22 |
| 1019 | I1NGS1 | uncharacterized protein | 10 | 0.37 | 24.9 | 4.51 |
| 1020 | C6TI81 | ferritin | 6 | 0.37 | 27.9 | 5.71 |
| 1021 | C6TC46 | uncharacterized protein | 8 | 0.37 | 49.2 | 4.87 |
| 1022 | C6THB8 | uncharacterized protein | 9 | 0.36 | 50.1 | 4.72 |
| 1023 | I1KGB8 | uncharacterized protein | 16 | 0.36 | 61.1 | 7.13 |
| 1024 | C6TLT7 | late-embryogenesis abundant protein 1 | 16 | 0.36 | 35.6 | 4.8 |
| 1025 | Q8RVH5 | basic 7s globulin 2 | 10 | 0.36 | 47.2 | 8.72 |
| 1026 | I1NJE8 | peptidyl-prolyl cis-trans isomerase | 5 | 0.36 | 27.1 | 9.56 |
| 1027 | I1N318 | proteasome subunit alpha type | 15 | 0.36 | 27.3 | 6.61 |
| 1028 | K7MY09 | uncharacterized protein | 7 | 0.36 | 51.2 | 5.02 |
| 1029 | C6TGI0 | uncharacterized protein | 8 | 0.36 | 23.6 | 7.64 |
| 1030 | I1N7E6 | uncharacterized protein | 8 | 0.36 | 47.7 | 5.64 |
| 1031 | P24095 | seed linoleate 9s-lipoxygenase | 48 | 0.36 | 96.8 | 5.78 |
| 1032 | I1LKT2 | uncharacterized protein | 5 | 0.35 | 48.5 | 8.46 |
| 1033 | I1JSD2 | uncharacterized protein | 16 | 0.35 | 50 | 6.05 |
| 1034 | A8IKE1 | alanine aminotransferase 1 | 17 | 0.35 | 53.3 | 5.31 |
| 1035 | A0A0R4J336 | uncharacterized protein | 6 | 0.35 | 25.5 | 8.28 |
| 1036 | I1LF17 | uncharacterized protein | 6 | 0.34 | 30.8 | 4.86 |
| 1037 | I1JMH3 | uncharacterized protein | 3 | 0.34 | 43.5 | 6.71 |
| 1038 | I1MK72 | uncharacterized protein | 16 | 0.34 | 59.4 | 6.13 |
| 1039 | A0A0R0F2G6 | uncharacterized protein | 13 | 0.34 | 52.5 | 8.04 |
| 1040 | I1JMX7 | uncharacterized protein | 5 | 0.34 | 49 | 8.37 |
| 1041 | I1NCF0 | uncharacterized protein | 49 | 0.33 | 287.7 | 6.38 |
| 1042 | K7MBG3 | sucrose synthase | 21 | 0.33 | 91.5 | 5.84 |
| 1043 | I1LWV7 | uncharacterized protein | 13 | 0.33 | 40.5 | 5.49 |
| 1044 | A0A0R0KAL0 | uncharacterized protein | 15 | 0.33 | 61.8 | 4.72 |
| 1045 | I1KY80 | uncharacterized protein | 5 | 0.33 | 19.7 | 9.06 |
| 1046 | I1KLY0 | uncharacterized protein | 5 | 0.33 | 47.3 | 6.65 |
| 1047 | I1LKY8 | uncharacterized protein | 5 | 0.33 | 61.5 | 6.65 |
| 1048 | C6TJ00 | uncharacterized protein | 5 | 0.33 | 40.1 | 5.56 |
| 1049 | I1KZX0 | uncharacterized protein | 11 | 0.33 | 35.8 | 5.14 |
| 1050 | I1LJJ4 | uncharacterized protein | 18 | 0.32 | 53.5 | 6.39 |
| 1051 | I1M5E5 | uncharacterized protein | 13 | 0.32 | 48.4 | 5.42 |
| 1052 | I1N6R2 | 60s ribosomal protein l36 | 2 | 0.32 | 13.3 | 10.99 |
| 1053 | I1N5E4 | uncharacterized protein | 18 | 0.32 | 48 | 6.14 |
| 1054 | I1LSZ2 | ubiquitinyl hydrolase 1 | 20 | 0.31 | 89.8 | 5.07 |
| 1055 | I1JCI7 | peptidyl-prolyl cis-trans isomerase | 3 | 0.31 | 13.6 | 9.56 |
| 1056 | I1KSB2 | uncharacterized protein | 6 | 0.31 | 49.1 | 7 |
| 1057 | I1LFG6 | uncharacterized protein | 26 | 0.31 | 67.6 | 7.2 |
| 1058 | I1LUL9 | ketol-acid reductoisomerase | 21 | 0.3 | 63.3 | 6.85 |
| 1059 | I1K099 | uncharacterized protein | 12 | 0.3 | 52.3 | 6.54 |
| 1060 | I1MIC1 | uncharacterized protein | 8 | 0.3 | 45.2 | 4.84 |
| 1061 | I1JRL2 | uncharacterized protein | 2 | 0.3 | 44.6 | 5 |
| 1062 | I1L5Z2 | aldose 1-epimerase | 10 | 0.3 | 40.7 | 9.35 |
| 1063 | A0A0R4J3N1 | uncharacterized protein | 16 | 0.3 | 47.7 | 6.14 |
| 1064 | I1JUS6 | aspartate aminotransferase | 23 | 0.3 | 45.3 | 7.75 |
| 1065 | Q76LA6 | cytosolic ascorbate peroxidase 2 | 12 | 0.29 | 27.1 | 5.65 |
| 1066 | P34811 | elongation factor g-1 | 9 | 0.29 | 86.8 | 5.04 |
| 1067 | I1KV97 | uncharacterized protein | 19 | 0.29 | 103 | 6.59 |
| 1068 | I1NJ89 | uncharacterized protein | 14 | 0.29 | 68.5 | 5.54 |
| 1069 | K7KHJ1 | glucose-1-phosphate adenylyltransferase | 9 | 0.28 | 57 | 5.98 |
| 1070 | P29756 | catalase-1/2 | 18 | 0.28 | 56.8 | 6.77 |
| 1071 | C6TG44 | uncharacterized protein | 9 | 0.28 | 38.4 | 5.06 |
| 1072 | I1LN41 | uncharacterized protein | 19 | 0.28 | 84.5 | 5.9 |
| 1073 | I1MG10 | protein root hair defective 3 homolog | 12 | 0.28 | 92.9 | 5.68 |
| 1074 | I1KQD8 | uncharacterized protein | 9 | 0.27 | 41.4 | 6.35 |
| 1075 | A0A0R0EC54 | uncharacterized protein | 7 | 0.26 | 17.5 | 10.46 |
| 1076 | I1J9U5 | uncharacterized protein | 31 | 0.26 | 126.3 | 6.16 |
| 1077 | I1LMW7 | uncharacterized protein | 13 | 0.26 | 32.6 | 5.07 |
| 1078 | K7L145 | uncharacterized protein | 7 | 0.26 | 50.3 | 5.98 |
| 1079 | I1KC56 | uncharacterized protein | 20 | 0.25 | 53.3 | 6.31 |
| 1080 | I1JXY2 | uncharacterized protein | 26 | 0.25 | 103.9 | 5.87 |
| 1081 | I1L4J2 | uncharacterized protein | 24 | 0.25 | 78.1 | 9.28 |
| 1082 | I1JB16 | uncharacterized protein | 6 | 0.25 | 82.2 | 4.86 |
| 1083 | I1K5M9 | uncharacterized protein | 29 | 0.24 | 63.5 | 5.33 |
| 1084 | I1KSR6 | uncharacterized protein | 17 | 0.24 | 39 | 5.12 |
| 1085 | K7LSL2 | uncharacterized protein | 11 | 0.24 | 37.9 | 6.18 |
| 1086 | C6SZN7 | thioredoxin | 5 | 0.24 | 13 | 5.29 |
| 1087 | I1MIW5 | uncharacterized protein | 10 | 0.24 | 51.4 | 6.16 |
| 1088 | I1N8S9 | uncharacterized protein | 25 | 0.24 | 172.1 | 6.69 |
| 1089 | C6TJG0 | uncharacterized protein | 11 | 0.23 | 26.6 | 6.77 |
| 1090 | A0A0R0FLF0 | xyloglucan endotransglucosylase/hydrolase | 13 | 0.23 | 40.4 | 9.31 |
| 1091 | I1JDR2 | uncharacterized protein | 39 | 0.23 | 116.4 | 6.34 |
| 1092 | I1JP48 | uncharacterized protein | 17 | 0.23 | 74.8 | 5.83 |
| 1093 | I1MKN3 | uncharacterized protein | 16 | 0.23 | 34 | 9.35 |
| 1094 | C6T5D6 | uncharacterized protein | 7 | 0.23 | 13.6 | 6.91 |
| 1095 | I1KZJ9 | uncharacterized protein | 22 | 0.23 | 81 | 7.03 |
| 1096 | A0A0R4J2P7 | uncharacterized protein | 9 | 0.22 | 28.5 | 5.97 |
| 1097 | I1NF29 | uncharacterized protein | 25 | 0.22 | 86.4 | 5.8 |
| 1098 | I1L9B1 | lysine--trna ligase | 19 | 0.22 | 68.1 | 6.04 |
| 1099 | I1M322 | uncharacterized protein | 17 | 0.21 | 46.8 | 6.48 |
| 1100 | I1KT56 | uncharacterized protein | 12 | 0.21 | 50.2 | 7.6 |
| 1101 | I1JCC5 | uncharacterized protein | 2 | 0.21 | 64.6 | 4.85 |
| 1102 | I1N3B9 | uncharacterized protein | 7 | 0.21 | 36.9 | 8.93 |
| 1103 | I1KVU0 | uncharacterized protein | 34 | 0.2 | 68.7 | 5.48 |
| 1104 | A0A0R4J321 | proteasome subunit beta type | 11 | 0.2 | 25.1 | 5.16 |
| 1105 | I1M6K6 | uncharacterized protein | 18 | 0.19 | 81.7 | 5.51 |
| 1106 | I1JGB5 | eukaryotic translation initiation factor 3 subunit e | 20 | 0.17 | 51.1 | 5.53 |
| 1107 | C6T9B4 | uncharacterized protein | 9 | 0.17 | 38.3 | 6.37 |
| 1108 | I1MP01 | glycosyltransferase | 7 | 0.17 | 53.8 | 5.62 |
| 1109 | I1NJ85 | uncharacterized protein | 27 | 0.17 | 67.4 | 6.51 |
| 1110 | C6SWQ6 | uncharacterized protein | 10 | 0.15 | 26.9 | 9.54 |
| 1111 | C6TJT0 | uncharacterized protein | 13 | 0.15 | 39.9 | 8.99 |
| 1112 | C6TCX8 | uncharacterized protein | 14 | 0.15 | 51.7 | 6.35 |
| 1113 | K7MU41 | uncharacterized protein | 7 | 0.14 | 66.9 | 8.8 |
| 1114 | I1KSC8 | uncharacterized protein | 32 | 0.12 | 123.2 | 4.75 |
| 1115 | I1MJU7 | uncharacterized protein | 21 | 0.12 | 73.7 | 5.2 |
| 1116 | C6T857 | isocitrate dehydrogenase | 20 | -0.08 | 46.1 | 5.87 |
| 1117 | I1JXS8 | uncharacterized protein | 40 | -0.12 | 108.9 | 6.13 |
| 1118 | I1KMP3 | uncharacterized protein | 15 | -0.14 | 54.2 | 6.04 |
| 1119 | I1K670 | uncharacterized protein | 28 | -0.15 | 73.6 | 5.02 |
| 1120 | I1L6W0 | uncharacterized protein | 26 | -0.15 | 60.8 | 5.51 |
| 1121 | I1N4I6 | UDP-glucose 6-dehydrogenase | 21 | -0.16 | 52.9 | 5.74 |
| 1122 | A0A0R0KZ26 | uncharacterized protein | 8 | -0.16 | 21.9 | 9.17 |
| 1123 | I1MB76 | uncharacterized protein | 8 | -0.16 | 64.7 | 5.19 |
| 1124 | B0M1A6 | betaine aldehyde dehydrogenase | 29 | -0.18 | 54.7 | 5.23 |
| 1125 | A0A0R4J3L5 | proteasome subunit alpha type | 13 | -0.18 | 27.4 | 5.57 |
| 1126 | A0A0R0K6S7 | uncharacterized protein | 62 | -0.18 | 252.2 | 6 |
| 1127 | A0A0R0FUV4 | uncharacterized protein | 18 | -0.19 | 46.6 | 5.34 |
| 1128 | I1MGE7 | uncharacterized protein | 30 | -0.2 | 65.9 | 7.95 |
| 1129 | I1LCQ1 | pyruvate kinase | 19 | -0.21 | 57.8 | 6.93 |
| 1130 | I1K2I1 | tubulin alpha chain | 22 | -0.21 | 49.6 | 4.96 |
| 1131 | C6T514 | 40s ribosomal protein s8 | 9 | -0.21 | 27.6 | 10.19 |
| 1132 | I1MTH1 | pantothenate kinase 2 | 7 | -0.22 | 100 | 6.02 |
| 1133 | C6TGR3 | uncharacterized protein | 6 | -0.22 | 25.5 | 9.08 |
| 1134 | D6C500 | hsp90-2 | 41 | -0.23 | 80.1 | 4.97 |
| 1135 | C6T8K3 | uncharacterized protein | 14 | -0.23 | 27.4 | 5.13 |
| 1136 | I1JR35 | uncharacterized protein | 12 | -0.23 | 39.5 | 7.65 |
| 1137 | K7KN32 | l-lactate dehydrogenase | 11 | -0.23 | 44.9 | 7.18 |
| 1138 | I1N4A4 | thioredoxin | 3 | -0.24 | 14.7 | 5.35 |
| 1139 | I1MYX8 | alpha-snap protein | 14 | -0.24 | 32.7 | 5.07 |
| 1140 | I1N520 | uncharacterized protein | 21 | -0.24 | 35.1 | 6.12 |
| 1141 | K7MBV6 | uncharacterized protein | 9 | -0.25 | 31.9 | 10.06 |
| 1142 | Q39817 | calnexin homolog | 17 | -0.25 | 62 | 4.77 |
| 1143 | C6T973 | uncharacterized protein | 2 | -0.25 | 13.7 | 9.66 |
| 1144 | I1K489 | guanosine nucleotide diphosphate dissociation inhibitor | 23 | -0.25 | 49.6 | 5.4 |
| 1145 | I1L0U6 | importin subunit alpha | 16 | -0.25 | 58.6 | 5.15 |
| 1146 | C6TAW2 | 40s ribosomal protein s24 | 3 | -0.26 | 15.8 | 10.64 |
| 1147 | I1JDM8 | uncharacterized protein | 6 | -0.26 | 48.7 | 6.22 |
| 1148 | C6TKE9 | uncharacterized protein | 8 | -0.26 | 27 | 7.1 |
| 1149 | I1NA39 | lambda class glutathione s-transferase | 12 | -0.26 | 27 | 5.49 |
| 1150 | Q02909 | phosphoenolpyruvate carboxylase | 46 | -0.27 | 110.6 | 5.66 |
| 1151 | C6T898 | tubulin alpha chain | 23 | -0.27 | 49.6 | 5.03 |
| 1152 | I1KH61 | uncharacterized protein | 20 | -0.28 | 103.1 | 6.49 |
| 1153 | Q944T2 | translationally-controlled tumor protein homolog | 9 | -0.28 | 19 | 4.57 |
| 1154 | I1MTZ8 | uncharacterized protein | 16 | -0.28 | 110 | 5.02 |
| 1155 | C6SZ14 | uncharacterized protein | 7 | -0.28 | 26.6 | 5.33 |
| 1156 | I1M261 | uncharacterized protein | 20 | -0.28 | 56.7 | 5.52 |
| 1157 | I1N7G4 | uncharacterized protein | 24 | -0.28 | 60.8 | 6.38 |
| 1158 | Q71EW8 | methionine synthase | 43 | -0.29 | 84.2 | 5.93 |
| 1159 | I1NIT7 | 40s ribosomal protein s24 | 3 | -0.29 | 16.1 | 10.34 |
| 1160 | A0A0R0I0B9 | uncharacterized protein | 13 | -0.29 | 26.3 | 9.59 |
| 1161 | B1ACD5 | kunitz trypsin protease inhibitor | 6 | -0.3 | 22.7 | 5.07 |
| 1162 | C6SXW4 | proteasome subunit beta type | 5 | -0.3 | 22.8 | 5.45 |
| 1163 | I1JSX8 | uncharacterized protein | 11 | -0.3 | 60.6 | 9.42 |
| 1164 | I1KKN7 | pyrophosphate--fructose 6-phosphate 1-phosphotransferase subunit alpha | 26 | -0.3 | 67.4 | 6.89 |
| 1165 | A0A762 | calreticulin-1 | 19 | -0.31 | 48.1 | 4.39 |
| 1166 | I1KNH4 | pyrophosphate--fructose 6-phosphate 1-phosphotransferase subunit beta | 19 | -0.32 | 62.1 | 6.26 |
| 1167 | I1MC31 | uncharacterized protein | 30 | -0.32 | 97.3 | 4.87 |
| 1168 | C6SV78 | ribosomal protein l15 | 4 | -0.32 | 24.1 | 11.64 |
| 1169 | I1KS65 | adenosylhomocysteinase | 26 | -0.32 | 53.3 | 5.6 |
| 1170 | I1KJI7 | nucleoside diphosphate kinase | 9 | -0.33 | 16.5 | 6.29 |
| 1171 | I1JJ67 | small nuclear ribonucleoprotein-associated protein | 3 | -0.33 | 29.4 | 11.28 |
| 1172 | I1JUQ0 | uncharacterized protein | 21 | -0.33 | 46.9 | 5.67 |
| 1173 | A0A0R0GTU4 | uncharacterized protein | 7 | -0.33 | 38.6 | 6.88 |
| 1174 | I1MZ13 | clathrin heavy chain | 68 | -0.34 | 192.8 | 5.36 |
| 1175 | A0A0R4J2Q8 | serine hydroxymethyltransferase | 17 | -0.34 | 56.9 | 8.13 |
| 1176 | I1LB97 | uncharacterized protein | 6 | -0.34 | 14.1 | 9.57 |
| 1177 | I1MUM8 | malate synthase | 22 | -0.34 | 62.7 | 5.95 |
| 1178 | K7LU80 | uncharacterized protein | 6 | -0.34 | 35.8 | 6.6 |
| 1179 | I1MH79 | translation machinery-associated protein 22 | 3 | -0.34 | 21 | 6.43 |
| 1180 | C6T160 | uncharacterized protein | 3 | -0.34 | 10.3 | 9.99 |
| 1181 | C6T851 | uncharacterized protein | 6 | -0.34 | 45.1 | 4.42 |
| 1182 | K7MU75 | uncharacterized protein | 3 | -0.34 | 129.2 | 5.04 |
| 1183 | I1KSQ0 | guanosine nucleotide diphosphate dissociation inhibitor | 22 | -0.34 | 49.5 | 5.4 |
| 1184 | I1L4U3 | uncharacterized protein | 29 | -0.34 | 88.4 | 5.93 |
| 1185 | C6SZI8 | uncharacterized protein | 11 | -0.34 | 22.1 | 6.52 |
| 1186 | C6T034 | uncharacterized protein | 15 | -0.35 | 24.3 | 4.53 |
| 1187 | C6T918 | uncharacterized protein | 11 | -0.35 | 37.1 | 4.92 |
| 1188 | A0A0R0FZ23 | uncharacterized protein | 10 | -0.35 | 16.3 | 11.02 |
| 1189 | I1NH59 | uncharacterized protein | 9 | -0.35 | 24.7 | 4.78 |
| 1190 | I1MEH8 | uncharacterized protein | 8 | -0.35 | 22.2 | 6.75 |
| 1191 | A0A0R0G6T3 | uncharacterized protein | 20 | -0.35 | 27.2 | 5.87 |
| 1192 | A0A0R0FJR0 | 40s ribosomal protein | 15 | -0.35 | 34 | 5.02 |
| 1193 | I1MFL0 | uncharacterized protein | 9 | -0.35 | 32.9 | 5.48 |
| 1194 | A0A0R0EXW6 | uncharacterized protein | 16 | -0.35 | 49.5 | 5.91 |
| 1195 | I1JTG3 | uncharacterized protein | 13 | -0.36 | 42.7 | 6.13 |
| 1196 | I1LA46 | uncharacterized protein | 5 | -0.36 | 30.6 | 6.2 |
| 1197 | I1KP22 | uncharacterized protein | 2 | -0.36 | 63.8 | 5.47 |
| 1198 | I1JFX0 | uncharacterized protein | 7 | -0.36 | 20.7 | 6.65 |
| 1199 | I1JBV5 | pyruvate dehydrogenase e1 component subunit alpha | 9 | -0.36 | 43.3 | 7.62 |
| 1200 | I1JQV8 | s-adenosylmethionine synthase | 17 | -0.36 | 43.2 | 5.57 |
| 1201 | I1LVA7 | uncharacterized protein | 25 | -0.36 | 75.6 | 6.65 |
| 1202 | I1KZW7 | pyrophosphate--fructose 6-phosphate 1-phosphotransferase subunit beta | 20 | -0.36 | 61.4 | 6.58 |
| 1203 | I1KM08 | uncharacterized protein | 2 | -0.36 | 19.5 | 9.1 |
| 1204 | C6T0A8 | uncharacterized protein | 6 | -0.36 | 27.7 | 7.05 |
| 1205 | I1NFK8 | uncharacterized protein | 25 | -0.37 | 84.8 | 6.09 |
| 1206 | I1ND04 | uncharacterized protein | 13 | -0.37 | 161.1 | 4.45 |
| 1207 | Q8W1A0 | cysteine synthase | 11 | -0.37 | 34.2 | 5.69 |
| 1208 | A0A0R0HR30 | uncharacterized protein | 17 | -0.37 | 47 | 5.49 |
| 1209 | A0A0R0FEM3 | uncharacterized protein | 8 | -0.37 | 49.4 | 9.2 |
| 1210 | I1JPX8 | fructose-bisphosphate aldolase | 16 | -0.38 | 38.5 | 6.35 |
| 1211 | I1NEX1 | phosphoacetylglucosamine mutase | 5 | -0.38 | 60.7 | 5.45 |
| 1212 | I1KFZ1 | uncharacterized protein | 2 | -0.38 | 58.4 | 10.02 |
| 1213 | A0A0R0K553 | uncharacterized protein | 7 | -0.38 | 34.8 | 5.42 |
| 1214 | I1JMI9 | malic enzyme | 15 | -0.38 | 68.9 | 5.71 |
| 1215 | I1NF54 | uncharacterized protein | 11 | -0.38 | 63.7 | 7.61 |
| 1216 | I1KWS2 | uncharacterized protein | 28 | -0.4 | 119 | 4.82 |
| 1217 | I1JXA0 | uncharacterized protein | 39 | -0.4 | 90.8 | 5.11 |
| 1218 | A0A0R4J307 | uncharacterized protein | 3 | -0.4 | 25.4 | 6.01 |
| 1219 | I1KUN0 | uncharacterized protein | 34 | -0.4 | 184 | 5.71 |
| 1220 | C6TJX5 | uncharacterized protein | 9 | -0.4 | 16.6 | 6.96 |
| 1221 | O23960 | acetyl-coa carboxylase | 19 | -0.4 | 58.9 | 7.22 |
| 1222 | I1KT81 | uncharacterized protein | 8 | -0.4 | 51.3 | 7.7 |
| 1223 | C6TB70 | uncharacterized protein | 9 | -0.41 | 24.4 | 6.44 |
| 1224 | I1J5Y9 | UDP-glucose 6-dehydrogenase | 17 | -0.41 | 52.9 | 6.34 |
| 1225 | C6SXA0 | uncharacterized protein | 4 | -0.41 | 15 | 8.62 |
| 1226 | I1JEI0 | uncharacterized protein | 21 | -0.41 | 41.5 | 5.66 |
| 1227 | K7MHE5 | v-type proton atpase subunit c | 9 | -0.41 | 42.5 | 5.27 |
| 1228 | K7N529 | uncharacterized protein | 3 | -0.41 | 67.9 | 5.45 |
| 1229 | I1MBS8 | uncharacterized protein | 6 | -0.42 | 58.3 | 5.31 |
| 1230 | C6SWL4 | uncharacterized protein | 6 | -0.42 | 20.4 | 10.24 |
| 1231 | I1MY33 | tubulin beta chain | 27 | -0.42 | 50.5 | 4.73 |
| 1232 | C6T193 | uncharacterized protein | 9 | -0.42 | 26.2 | 10.76 |
| 1233 | A0A0R0ERR0 | uncharacterized protein | 19 | -0.42 | 47.4 | 4.98 |
| 1234 | C6T9Q3 | uncharacterized protein | 7 | -0.42 | 17.2 | 10.29 |
| 1235 | I1K5X6 | lactoylglutathione lyase | 6 | -0.42 | 40.6 | 8.17 |
| 1236 | C6T7Y1 | uncharacterized protein | 17 | -0.43 | 41.7 | 5.31 |
| 1237 | K7LSZ2 | uncharacterized protein | 7 | -0.43 | 62.8 | 6.08 |
| 1238 | C6T008 | uncharacterized protein | 4 | -0.43 | 25.3 | 8.98 |
| 1239 | C6TL29 | phosphoribulokinase | 7 | -0.43 | 45.3 | 5.89 |
| 1240 | C6TDL5 | uncharacterized protein | 9 | -0.43 | 28.1 | 10.72 |
| 1241 | A0A0R0E8N5 | uricase | 13 | -0.43 | 35.1 | 8.31 |
| 1242 | A0A0R0GZF0 | annexin | 19 | -0.44 | 35.7 | 6.78 |
| 1243 | I1K467 | uncharacterized protein | 23 | -0.44 | 59 | 6.09 |
| 1244 | Q53B72 | chalcone-flavonone isomerase family protein | 11 | -0.44 | 23.5 | 4.89 |
| 1245 | C6TIY1 | uncharacterized protein | 5 | -0.44 | 21.8 | 4.38 |
| 1246 | C6T670 | uncharacterized protein | 3 | -0.44 | 16.6 | 10.71 |
| 1247 | I1J9Y0 | uncharacterized protein | 20 | -0.44 | 65.6 | 6.07 |
| 1248 | A0A0R0HH38 | uncharacterized protein | 4 | -0.44 | 15.5 | 4.35 |
| 1249 | A0A0R0GBB6 | uncharacterized protein | 13 | -0.44 | 45.9 | 5.9 |
| 1250 | I1LIV8 | uncharacterized protein | 12 | -0.44 | 29.4 | 10.21 |
| 1251 | C6T0A7 | uncharacterized protein | 6 | -0.44 | 17.5 | 10.56 |
| 1252 | A0A0R0K474 | uncharacterized protein | 18 | -0.44 | 46.7 | 5.29 |
| 1253 | C6TFC1 | non-specific lipid-transfer protein | 5 | -0.44 | 12.4 | 9.03 |
| 1254 | K7K5V1 | uncharacterized protein | 11 | -0.45 | 23.2 | 8.59 |
| 1255 | A0A0R0KSS1 | uncharacterized protein | 11 | -0.45 | 37.9 | 5.93 |
| 1256 | I1J9W0 | 40s ribosomal protein s30 | 2 | -0.45 | 11.6 | 12.07 |
| 1257 | I1KJR2 | uncharacterized protein | 13 | -0.45 | 67.8 | 5.47 |
| 1258 | K7LQG0 | uncharacterized protein | 6 | -0.45 | 20.8 | 8.39 |
| 1259 | I1JRN4 | uncharacterized protein | 10 | -0.45 | 49.7 | 5.16 |
| 1260 | A0A0R0E4D5 | uncharacterized protein | 3 | -0.45 | 17.9 | 5.46 |
| 1261 | C6SWY6 | uncharacterized protein | 8 | -0.45 | 16.5 | 4.68 |
| 1262 | C6TKQ3 | uncharacterized protein | 18 | -0.45 | 34.7 | 5.93 |
| 1263 | C6TAT2 | tau class glutathione s-transferase | 6 | -0.45 | 25.6 | 5.57 |
| 1264 | C6TAK2 | probable cytosolic iron-sulfur protein assembly protein ciao1 homolog | 3 | -0.45 | 38.7 | 4.74 |
| 1265 | I1LZ92 | uncharacterized protein | 14 | -0.46 | 37.5 | 5.29 |
| 1266 | I1NF03 | uncharacterized protein | 20 | -0.46 | 52 | 4.43 |
| 1267 | F6KBT3 | allene oxide cyclase 3 | 7 | -0.46 | 28 | 8.75 |
| 1268 | C6T2W2 | uncharacterized protein | 4 | -0.46 | 10.8 | 6.71 |
| 1269 | A0A0R4J5B7 | 40s ribosomal protein sa | 13 | -0.47 | 34.1 | 5.09 |
| 1270 | I1K361 | uncharacterized protein | 8 | -0.47 | 54.1 | 5.88 |
| 1271 | I1JU53 | uncharacterized protein | 3 | -0.47 | 10.9 | 6.16 |
| 1272 | I1MA57 | aldehyde dehydrogenase | 7 | -0.47 | 54 | 9.03 |
| 1273 | I1LY74 | uncharacterized protein | 7 | -0.47 | 21.8 | 5.18 |
| 1274 | C6SWN5 | uncharacterized protein | 4 | -0.47 | 13.7 | 11.6 |
| 1275 | I1K7Q8 | uncharacterized protein | 11 | -0.47 | 34.2 | 5.73 |
| 1276 | I1M7B9 | uncharacterized protein | 24 | -0.47 | 60.2 | 6.19 |
| 1277 | C6TAW8 | uncharacterized protein | 5 | -0.47 | 17.5 | 5.32 |
| 1278 | I1KIS3 | uncharacterized protein | 13 | -0.48 | 54.5 | 5.93 |
| 1279 | I1MJX0 | acetyltransferase component of pyruvate dehydrogenase complex | 14 | -0.48 | 59.6 | 7.59 |
| 1280 | I1LVZ8 | uncharacterized protein | 27 | -0.48 | 85.5 | 8 |
| 1281 | I1K5E6 | uncharacterized protein | 17 | -0.48 | 35.6 | 7.62 |
| 1282 | A0A0R0I9G1 | uncharacterized protein | 19 | -0.48 | 116.8 | 9.41 |
| 1283 | C6TGA6 | 60s acidic ribosomal protein p0 | 11 | -0.48 | 34.2 | 5 |
| 1284 | A0A0R0HEI5 | uncharacterized protein | 19 | -0.48 | 58.2 | 6.61 |
| 1285 | I1KNN0 | ATP-dependent 6-phosphofructokinase | 17 | -0.49 | 51.1 | 6.22 |
| 1286 | C6T8X3 | uncharacterized protein | 19 | -0.49 | 46.9 | 5.46 |
| 1287 | C6SY97 | mitochondrial pyruvate carrier | 4 | -0.49 | 12.2 | 9.79 |
| 1288 | I1LTL9 | uncharacterized protein | 35 | -0.49 | 89.7 | 5.18 |
| 1289 | Q9FQE8 | glutathione s-transferase | 10 | -0.49 | 25.6 | 5.71 |
| 1290 | K7MGN4 | uncharacterized protein | 9 | -0.49 | 132.7 | 6.08 |
| 1291 | C6T8F0 | glutamine synthetase | 14 | -0.49 | 39.2 | 5.22 |
| 1292 | I1MKG5 | uncharacterized protein | 2 | -0.49 | 31.2 | 10.01 |
| 1293 | K7K5P1 | uncharacterized protein | 4 | -0.5 | 27.1 | 5.37 |
| 1294 | I1K108 | uncharacterized protein | 15 | -0.5 | 69.1 | 8.17 |
| 1295 | C6SXS1 | ribosomal protein l15 | 4 | -0.5 | 24.2 | 11.58 |
| 1296 | I1JB18 | uncharacterized protein | 2 | -0.5 | 19.2 | 6.16 |
| 1297 | I1LJ90 | coatomer subunit alpha | 28 | -0.5 | 136.7 | 6.49 |
| 1298 | C6T460 | tau class glutathione s-transferase | 9 | -0.5 | 25 | 5.69 |
| 1299 | I1L8E9 | uncharacterized protein | 14 | -0.5 | 75.1 | 6.37 |
| 1300 | I1LDP2 | uncharacterized protein | 7 | -0.51 | 27.9 | 9 |
| 1301 | I1MNJ7 | uncharacterized protein | 3 | -0.51 | 18.7 | 9.12 |
| 1302 | A0A0B5E2K6 | tau class glutathione s-transferase | 9 | -0.51 | 25.4 | 5.88 |
| 1303 | I1K5F2 | uncharacterized protein | 17 | -0.51 | 56.1 | 5.08 |
| 1304 | K7KJ72 | uncharacterized protein | 19 | -0.51 | 404.8 | 5.26 |
| 1305 | I1JD52 | uncharacterized protein | 19 | -0.51 | 58.6 | 5.25 |
| 1306 | I1LIH4 | uncharacterized protein | 7 | -0.51 | 68.7 | 9.23 |
| 1307 | I1JHQ1 | uncharacterized protein | 15 | -0.52 | 60.6 | 6 |
| 1308 | I1KYT6 | helicase | 10 | -0.52 | 51 | 5.34 |
| 1309 | I1N828 | uncharacterized protein | 2 | -0.52 | 43 | 5.86 |
| 1310 | Q39828 | dynamin-related protein 5a | 18 | -0.52 | 68.3 | 8.04 |
| 1311 | C6SVX3 | uncharacterized protein | 8 | -0.52 | 17.7 | 5.68 |
| 1312 | I1J582 | eukaryotic translation initiation factor 3 subunit i | 13 | -0.52 | 35.8 | 6.84 |
| 1313 | I1KTQ4 | citrate synthase | 17 | -0.53 | 52.5 | 8.24 |
| 1314 | I1MKC8 | starch synthase | 12 | -0.53 | 68.1 | 6.62 |
| 1315 | I1M8A5 | proteasome subunit alpha type | 9 | -0.53 | 31.1 | 5.15 |
| 1316 | A0A0R0ENW6 | uncharacterized protein | 9 | -0.53 | 46.3 | 8.73 |
| 1317 | Q42797 | trans-cinnamate 4-monooxygenase | 16 | -0.53 | 58 | 9.05 |
| 1318 | C6SVY0 | uncharacterized protein | 7 | -0.53 | 19.9 | 8.72 |
| 1319 | C6T1Q2 | uncharacterized protein | 3 | -0.54 | 21.3 | 8.64 |
| 1320 | I1NBI4 | uncharacterized protein | 19 | -0.54 | 58.5 | 6.12 |
| 1321 | I1N8M8 | uncharacterized protein | 18 | -0.54 | 111.3 | 5.62 |
| 1322 | I1LV12 | uncharacterized protein | 19 | -0.54 | 68.3 | 9.24 |
| 1323 | I1KU21 | uncharacterized protein | 38 | -0.54 | 94 | 5.8 |
| 1324 | K7KPN5 | uncharacterized protein | 6 | -0.54 | 50.9 | 8 |
| 1325 | K7KHJ7 | uncharacterized protein | 7 | -0.54 | 18 | 7.83 |
| 1326 | C6T3K3 | uncharacterized protein | 6 | -0.54 | 23.7 | 10.28 |
| 1327 | I1MHU4 | oxidoreductin 1a | 3 | -0.54 | 53.3 | 5.69 |
| 1328 | I1N512 | uncharacterized protein | 7 | -0.54 | 37.9 | 6.22 |
| 1329 | I1LN28 | uncharacterized protein | 4 | -0.54 | 51.3 | 9.62 |
| 1330 | Q8H928 | phosphoenolpyruvate carboxylase | 43 | -0.55 | 110.6 | 6.05 |
| 1331 | I1M5L7 | uncharacterized protein | 32 | -0.56 | 95.4 | 5.12 |
| 1332 | C6SZA1 | uncharacterized protein | 7 | -0.56 | 16.6 | 4.2 |
| 1333 | I1JRS9 | uncharacterized protein | 6 | -0.56 | 62 | 4.97 |
| 1334 | A0A0R4J5C6 | uncharacterized protein | 5 | -0.56 | 14.9 | 5.74 |
| 1335 | I1LZJ5 | uncharacterized protein | 6 | -0.56 | 42 | 7.27 |
| 1336 | I1MJD2 | cysteine synthase | 6 | -0.56 | 33.9 | 5.34 |
| 1337 | I1LGI7 | hydroxymethylglutaryl-coa synthase | 14 | -0.56 | 52.4 | 5.77 |
| 1338 | I1K9A0 | eukaryotic translation initiation factor 3 subunit l | 17 | -0.56 | 60.8 | 5.88 |
| 1339 | C6T1X3 | uncharacterized protein | 2 | -0.57 | 20.6 | 7.83 |
| 1340 | C6TA11 | uncharacterized protein | 15 | -0.57 | 54 | 6.32 |
| 1341 | I1LZP7 | uncharacterized protein | 8 | -0.57 | 79.9 | 5.81 |
| 1342 | I1LPS2 | uncharacterized protein | 6 | -0.57 | 202.2 | 5.81 |
| 1343 | C6T0B5 | uncharacterized protein | 4 | -0.57 | 14 | 5.73 |
| 1344 | C6T017 | uncharacterized protein | 11 | -0.57 | 21.7 | 9.58 |
| 1345 | K7LKF5 | uncharacterized protein | 10 | -0.57 | 55.7 | 5.25 |
| 1346 | I1JSK2 | uncharacterized protein | 6 | -0.57 | 43.1 | 6.13 |
| 1347 | I1NFB9 | diphosphomevalonate decarboxylase | 9 | -0.57 | 46.4 | 5.83 |
| 1348 | A0A0R0I5G1 | uncharacterized protein | 27 | -0.57 | 124.7 | 6.44 |
| 1349 | C6TFV2 | uncharacterized protein | 4 | -0.57 | 22.5 | 7.37 |
| 1350 | C6SVT0 | uncharacterized protein | 6 | -0.57 | 18 | 6.72 |
| 1351 | I1JGR5 | uncharacterized protein | 27 | -0.58 | 71.6 | 5.2 |
| 1352 | C6SZ55 | uncharacterized protein | 3 | -0.58 | 10.6 | 7.99 |
| 1353 | I1ND96 | uncharacterized protein | 5 | -0.58 | 10.6 | 6.82 |
| 1354 | I1KA75 | uncharacterized protein | 11 | -0.58 | 48.5 | 7.64 |
| 1355 | I1MQN0 | transmembrane 9 superfamily member | 6 | -0.58 | 67.8 | 6.92 |
| 1356 | I1KB73 | uncharacterized protein | 9 | -0.58 | 42.3 | 5.91 |
| 1357 | I1LII4 | uncharacterized protein | 19 | -0.59 | 123.9 | 5.42 |
| 1358 | I1N1Y9 | peptidylprolyl isomerase | 13 | -0.59 | 60.9 | 5.16 |
| 1359 | K7N4I7 | uncharacterized protein | 3 | -0.59 | 22.1 | 9.27 |
| 1360 | C6T871 | uncharacterized protein | 6 | -0.59 | 40.1 | 6.22 |
| 1361 | I1JAN9 | uncharacterized protein | 3 | -0.59 | 17.3 | 9.56 |
| 1362 | C6TIC4 | reticulon-like protein | 3 | -0.6 | 28.5 | 7.78 |
| 1363 | I1KJS2 | ATP-dependent 6-phosphofructokinase | 11 | -0.6 | 58.5 | 7.25 |
| 1364 | I1JIA6 | uncharacterized protein | 2 | -0.6 | 7.7 | 9.74 |
| 1365 | I1L1J3 | uncharacterized protein | 11 | -0.6 | 41.5 | 4.39 |
| 1366 | I1KMC2 | pyruvate kinase | 17 | -0.6 | 54.3 | 7.53 |
| 1367 | C6TN20 | eukaryotic translation initiation factor 3 subunit h | 9 | -0.6 | 38.8 | 4.73 |
| 1368 | K7LBW0 | 60s ribosomal protein l27 | 5 | -0.61 | 21.8 | 10.94 |
| 1369 | I1K5Q5 | uncharacterized protein | 6 | -0.61 | 14.9 | 9.33 |
| 1370 | I1KLC9 | uncharacterized protein | 18 | -0.61 | 65.2 | 7.55 |
| 1371 | Q9FQ95 | in2-1 protein | 13 | -0.61 | 27 | 5.21 |
| 1372 | I1L3W4 | eukaryotic translation initiation factor 3 subunit j | 6 | -0.61 | 25.8 | 4.72 |
| 1373 | I1K974 | uncharacterized protein | 13 | -0.61 | 50.8 | 9.31 |
| 1374 | C6TK30 | 60s ribosomal protein l36 | 3 | -0.62 | 12.4 | 11.32 |
| 1375 | I1KB25 | uncharacterized protein | 8 | -0.62 | 24.2 | 4.27 |
| 1376 | C6SWH1 | uncharacterized protein | 4 | -0.62 | 17.7 | 5.44 |
| 1377 | K7LSN8 | peptidyl-prolyl cis-trans isomerase | 7 | -0.62 | 25.2 | 9.01 |
| 1378 | I1J4D5 | uncharacterized protein | 11 | -0.62 | 26.6 | 5.62 |
| 1379 | A0A0R0HDA3 | protein disulfide-isomerase | 15 | -0.62 | 67.3 | 4.82 |
| 1380 | A0A0R4J460 | uncharacterized protein | 11 | -0.63 | 46.3 | 9.42 |
| 1381 | K7MVB9 | uncharacterized protein | 37 | -0.63 | 116 | 6.39 |
| 1382 | I1K1I4 | uncharacterized protein | 4 | -0.63 | 83.8 | 9.6 |
| 1383 | I1JGU8 | phospho-2-dehydro-3-deoxyheptonate aldolase | 12 | -0.63 | 59 | 8.57 |
| 1384 | K7MIR3 | uncharacterized protein | 17 | -0.64 | 38.3 | 6.35 |
| 1385 | I1JK06 | reticulon-like protein | 4 | -0.64 | 62.3 | 8.64 |
| 1386 | A0A0R0KIF2 | uncharacterized protein | 19 | -0.64 | 86.6 | 4.64 |
| 1387 | I1N272 | acyl-[acyl-carrier-protein] hydrolase | 4 | -0.64 | 41.9 | 8.11 |
| 1388 | I1MYZ6 | uncharacterized protein | 5 | -0.64 | 79.1 | 5.12 |
| 1389 | A0A0R0EXE0 | uncharacterized protein | 4 | -0.64 | 26 | 4.76 |
| 1390 | K7MMF4 | uncharacterized protein | 2 | -0.65 | 22 | 10.01 |
| 1391 | I1JS35 | uncharacterized protein | 8 | -0.65 | 65.5 | 6.6 |
| 1392 | I1NFP9 | uncharacterized protein | 6 | -0.65 | 78.2 | 5.83 |
| 1393 | C6TJ08 | uncharacterized protein | 12 | -0.65 | 35.2 | 5.82 |
| 1394 | I1JH86 | fructose-bisphosphate aldolase | 22 | -0.65 | 38.2 | 7.12 |
| 1395 | K7S7Y8 | acyl-[acyl-carrier-protein] desaturase | 9 | -0.65 | 45.6 | 6.35 |
| 1396 | C6SVN9 | uncharacterized protein | 2 | -0.66 | 7.9 | 9.06 |
| 1397 | C6TML0 | uncharacterized protein | 9 | -0.66 | 33.1 | 6.17 |
| 1398 | A0A0R4J364 | uncharacterized protein | 16 | -0.66 | 40.9 | 5.97 |
| 1399 | I1JMQ3 | uncharacterized protein | 10 | -0.66 | 39.1 | 5.86 |
| 1400 | C6T4R9 | uncharacterized protein | 7 | -0.66 | 17.7 | 9.77 |
| 1401 | I1LMS9 | clathrin heavy chain | 65 | -0.66 | 192.9 | 5.29 |
| 1402 | I1JLN6 | uncharacterized protein | 4 | -0.66 | 40.2 | 8.29 |
| 1403 | C6TI82 | methyltransferase | 8 | -0.67 | 38.5 | 6.22 |
| 1404 | K7MZ19 | uncharacterized protein | 14 | -0.67 | 43.9 | 6.67 |
| 1405 | C6T1X0 | uncharacterized protein | 3 | -0.67 | 20.2 | 9.48 |
| 1406 | I1LBI3 | cysteine synthase | 4 | -0.67 | 34.5 | 5.38 |
| 1407 | I1M7P3 | uncharacterized protein | 2 | -0.67 | 14.7 | 4.76 |
| 1408 | C6TKZ2 | uncharacterized protein | 14 | -0.67 | 38.5 | 6.68 |
| 1409 | C6TFW0 | uncharacterized protein | 4 | -0.67 | 13.3 | 9.32 |
| 1410 | K7LTT9 | ferredoxin | 3 | -0.67 | 22.7 | 7.04 |
| 1411 | Q5ECI5 | plastid 3-keto-acyl-acp synthase ii-b | 4 | -0.68 | 52.4 | 8.67 |
| 1412 | I1JSU0 | uncharacterized protein | 2 | -0.68 | 20.2 | 8.34 |
| 1413 | A0A0R4J3M4 | uncharacterized protein | 16 | -0.68 | 65.1 | 5.85 |
| 1414 | I1L954 | threonine dehydratase | 7 | -0.68 | 65.8 | 6.96 |
| 1415 | I1JUT9 | uncharacterized protein | 20 | -0.68 | 110.3 | 6.49 |
| 1416 | I1LU78 | uncharacterized protein | 8 | -0.69 | 30.8 | 6.97 |
| 1417 | I1MZ47 | uncharacterized protein | 14 | -0.69 | 34.7 | 5.85 |
| 1418 | C6SYZ4 | 60s ribosomal protein l13 | 6 | -0.7 | 26.8 | 11.08 |
| 1419 | O65016 | actin 4 | 19 | -0.7 | 41.4 | 5.6 |
| 1420 | I1MLB1 | uncharacterized protein | 8 | -0.7 | 97.6 | 5.1 |
| 1421 | C6TJF3 | uncharacterized protein | 2 | -0.7 | 33.8 | 5 |
| 1422 | K7L7P5 | uncharacterized protein | 6 | -0.7 | 28.4 | 4.18 |
| 1423 | I1JW44 | uncharacterized protein | 10 | -0.7 | 46.9 | 8.91 |
| 1424 | I1LV40 | uncharacterized protein | 10 | -0.7 | 39.2 | 8.68 |
| 1425 | I1MNX6 | uncharacterized protein | 9 | -0.7 | 47.4 | 6.57 |
| 1426 | I1JBQ4 | uncharacterized protein | 8 | -0.7 | 41.6 | 7.74 |
| 1427 | C6SWG4 | 40s ribosomal protein s21 | 2 | -0.71 | 9.2 | 6.81 |
| 1428 | I1LKR1 | uncharacterized protein | 10 | -0.71 | 82.8 | 4.8 |
| 1429 | I1KDB2 | uncharacterized protein | 7 | -0.71 | 47.3 | 6.09 |
| 1430 | A0A0R0GC70 | uncharacterized protein | 4 | -0.72 | 36.6 | 4.82 |
| 1431 | C6TCN5 | ferritin | 9 | -0.72 | 28.1 | 5.72 |
| 1432 | I1KAS2 | uncharacterized protein | 5 | -0.72 | 86.8 | 6.43 |
| 1433 | I1LRI7 | uncharacterized protein | 11 | -0.73 | 59.2 | 6.36 |
| 1434 | K7LTG4 | uncharacterized protein | 10 | -0.73 | 44.6 | 6.67 |
| 1435 | C6TMT8 | uncharacterized protein | 4 | -0.73 | 42.9 | 5.12 |
| 1436 | K7K5S3 | uncharacterized protein | 5 | -0.73 | 20.3 | 10.71 |
| 1437 | I1KP94 | glutathione peroxidase | 5 | -0.73 | 25.1 | 9.14 |
| 1438 | C6T032 | uncharacterized protein | 3 | -0.73 | 24.4 | 6.53 |
| 1439 | Q9FQD4 | glutathione s-transferase gst 24 | 9 | -0.73 | 24.8 | 5.74 |
| 1440 | C6TJP8 | uncharacterized protein | 3 | -0.73 | 32.2 | 5.07 |
| 1441 | I1LPJ9 | uncharacterized protein | 12 | -0.74 | 77.5 | 5.35 |
| 1442 | A0A0R0H271 | uncharacterized protein | 2 | -0.74 | 9.2 | 9.84 |
| 1443 | I1J8K5 | uncharacterized protein | 3 | -0.74 | 16.4 | 7.94 |
| 1444 | I1LAW6 | eukaryotic translation initiation factor 3 subunit m | 19 | -0.74 | 46.1 | 4.96 |
| 1445 | D6C4Z9 | hsp90-1 | 40 | -0.74 | 80.3 | 4.94 |
| 1446 | C6T8V8 | branched-chain-amino-acid aminotransferase | 8 | -0.74 | 45.1 | 6.08 |
| 1447 | I1MQT7 | uncharacterized protein | 29 | -0.74 | 134.4 | 5.66 |
| 1448 | A0A0R0G189 | uncharacterized protein | 15 | -0.74 | 86.6 | 6.66 |
| 1449 | K7L8B5 | uncharacterized protein | 8 | -0.75 | 15.3 | 4.44 |
| 1450 | I1K301 | uncharacterized protein | 6 | -0.75 | 14.8 | 5.6 |
| 1451 | A0A0R0IM79 | uncharacterized protein | 8 | -0.75 | 48.5 | 6.9 |
| 1452 | C6TJ03 | uncharacterized protein | 2 | -0.75 | 36.9 | 5.62 |
| 1453 | I1N0U0 | uncharacterized protein | 9 | -0.75 | 50.2 | 8.83 |
| 1454 | I1J8D4 | uncharacterized protein | 9 | -0.75 | 57.2 | 4.77 |
| 1455 | K7L9V4 | uncharacterized protein | 2 | -0.76 | 101.1 | 5.51 |
| 1456 | I1N587 | protein-lysine n-methyltransferase glyma_18g292800 | 8 | -0.76 | 40.3 | 4.49 |
| 1457 | I1KPB0 | uncharacterized protein | 10 | -0.76 | 58.9 | 6.06 |
| 1458 | I1NBM7 | uncharacterized protein | 11 | -0.77 | 55.5 | 6.28 |
| 1459 | A0A0R0G0Q7 | uncharacterized protein | 7 | -0.77 | 40.9 | 8.42 |
| 1460 | A0A0R0F2Z4 | uncharacterized protein | 24 | -0.77 | 60.8 | 5.58 |
| 1461 | C6T6Y4 | uncharacterized protein | 4 | -0.77 | 31.2 | 9.31 |
| 1462 | I1MT11 | uncharacterized protein | 21 | -0.77 | 86.8 | 7.52 |
| 1463 | I1KFL5 | uncharacterized protein | 5 | -0.78 | 35.2 | 6.98 |
| 1464 | I1MV67 | uncharacterized protein | 9 | -0.78 | 27.5 | 9.35 |
| 1465 | C6T1B2 | uncharacterized protein | 5 | -0.78 | 20.2 | 8.33 |
| 1466 | I1MC85 | uncharacterized protein | 7 | -0.78 | 45.8 | 5.55 |
| 1467 | I1MTB1 | uncharacterized protein | 5 | -0.79 | 42.1 | 5.26 |
| 1468 | C6T5M8 | uncharacterized protein | 4 | -0.79 | 20.3 | 6.93 |
| 1469 | I1JYT2 | uncharacterized protein | 5 | -0.79 | 59.5 | 5.06 |
| 1470 | Q39871 | late embryongenesis abundant protein | 18 | -0.79 | 50.6 | 6.33 |
| 1471 | C6TJJ8 | uncharacterized protein | 2 | -0.79 | 22.7 | 9.21 |
| 1472 | C6TNZ1 | uncharacterized protein | 2 | -0.8 | 24.6 | 9.16 |
| 1473 | K7L0V1 | uncharacterized protein | 27 | -0.8 | 148 | 5.67 |
| 1474 | I1LWI3 | uncharacterized protein | 7 | -0.8 | 31.5 | 5.11 |
| 1475 | K7KIL0 | GTP-binding nuclear protein | 8 | -0.8 | 25 | 6.42 |
| 1476 | C6T1P5 | uncharacterized protein | 5 | -0.8 | 15.5 | 9.84 |
| 1477 | A0A0R4J485 | uncharacterized protein | 3 | -0.8 | 29.4 | 5.36 |
| 1478 | I1K4F3 | alpha-galactosidase | 8 | -0.8 | 71.5 | 8.14 |
| 1479 | I1KEL5 | uncharacterized protein | 12 | -0.8 | 97.7 | 5.85 |
| 1480 | I1MVN5 | peroxidase | 15 | -0.8 | 35.5 | 9.13 |
| 1481 | I1JZE2 | methylenetetrahydrofolate reductase | 25 | -0.81 | 67 | 5.76 |
| 1482 | I1NJF1 | uncharacterized protein | 7 | -0.81 | 52.3 | 5.77 |
| 1483 | K7MMN7 | uncharacterized protein | 9 | -0.81 | 66.1 | 8.26 |
| 1484 | A0A0R0JP17 | oleosin | 3 | -0.81 | 21.4 | 10.28 |
| 1485 | K7MID0 | uncharacterized protein | 13 | -0.82 | 96.9 | 5.94 |
| 1486 | I1M4K9 | protein disulfide-isomerase | 15 | -0.82 | 62.3 | 4.7 |
| 1487 | I1M8P8 | uncharacterized protein | 3 | -0.82 | 52.7 | 8.04 |
| 1488 | I1JGB2 | s-formylglutathione hydrolase | 11 | -0.83 | 32.1 | 6.55 |
| 1489 | I1KCD7 | uncharacterized protein | 33 | -0.84 | 90.9 | 5.11 |
| 1490 | A0A0R0F392 | cysteine synthase | 9 | -0.85 | 40.1 | 6.62 |
| 1491 | I1KV92 | coatomer subunit beta' | 22 | -0.85 | 102.1 | 4.99 |
| 1492 | C6T274 | 1,2-dihydroxy-3-keto-5-methylthiopentene dioxygenase | 3 | -0.85 | 22.2 | 5.22 |
| 1493 | I1LJ75 | uncharacterized protein | 6 | -0.86 | 40.9 | 6.37 |
| 1494 | A0A0R4J559 | uncharacterized protein | 9 | -0.86 | 21.7 | 6.43 |
| 1495 | C6TCK6 | uncharacterized protein | 3 | -0.86 | 25.8 | 6.36 |
| 1496 | C6TNQ1 | uncharacterized protein | 9 | -0.86 | 34.9 | 5.21 |
| 1497 | I1NIG1 | uncharacterized protein | 4 | -0.87 | 27.1 | 7.88 |
| 1498 | I1K3S4 | adenosylhomocysteinase | 25 | -0.87 | 55.1 | 5.79 |
| 1499 | I1LY05 | uncharacterized protein | 11 | -0.87 | 43.2 | 4.41 |
| 1500 | A0A0R0EY92 | uncharacterized protein | 9 | -0.87 | 35.8 | 7.04 |
| 1501 | A0A0R0EME4 | uncharacterized protein | 6 | -0.87 | 39.9 | 5.44 |
| 1502 | I1LSM6 | trafficking protein particle complex subunit | 5 | -0.88 | 20.9 | 4.36 |
| 1503 | I1LTM3 | uncharacterized protein | 9 | -0.88 | 66.6 | 6.1 |
| 1504 | C6TBA9 | 40s ribosomal protein s8 | 6 | -0.88 | 24.8 | 10.37 |
| 1505 | A0A0R4J3Q4 | uncharacterized protein | 6 | -0.88 | 36.8 | 4.75 |
| 1506 | I1LBV4 | ubiquitin carboxyl-terminal hydrolase | 5 | -0.88 | 38.3 | 6.1 |
| 1507 | A0A0R0IA82 | uncharacterized protein | 5 | -0.88 | 52.8 | 5.24 |
| 1508 | I1LL14 | lactoylglutathione lyase | 11 | -0.89 | 26.5 | 9.16 |
| 1509 | I1LSW3 | uncharacterized protein | 9 | -0.89 | 50.1 | 5.42 |
| 1510 | C6SZG1 | uncharacterized protein | 2 | -0.89 | 11.6 | 8.38 |
| 1511 | I1K0G4 | uncharacterized protein | 2 | -0.9 | 20.6 | 7.62 |
| 1512 | C6TEE9 | uncharacterized protein | 9 | -0.9 | 30.4 | 5.74 |
| 1513 | I1NF56 | uncharacterized protein | 2 | -0.9 | 16.9 | 6.29 |
| 1514 | I1L088 | uncharacterized protein | 7 | -0.91 | 91.1 | 6.63 |
| 1515 | C6TIR2 | uncharacterized protein | 6 | -0.91 | 16.9 | 4.12 |
| 1516 | I1KMC9 | uncharacterized protein | 8 | -0.91 | 45.2 | 5.46 |
| 1517 | I1LRC1 | uncharacterized protein | 11 | -0.91 | 62.5 | 9.21 |
| 1518 | I1M8K9 | uncharacterized protein | 7 | -0.92 | 25.8 | 6.32 |
| 1519 | I1JKA6 | uncharacterized protein | 8 | -0.92 | 57.1 | 8.81 |
| 1520 | I1JMB4 | glyceraldehyde-3-phosphate dehydrogenase | 9 | -0.92 | 44.6 | 8.71 |
| 1521 | C6SWB0 | uncharacterized protein | 3 | -0.92 | 13.4 | 4.95 |
| 1522 | I1KE09 | uncharacterized protein | 7 | -0.93 | 17.3 | 4.79 |
| 1523 | K7K9D0 | uncharacterized protein | 18 | -0.93 | 107.4 | 5.73 |
| 1524 | K7MZM9 | uncharacterized protein | 7 | -0.93 | 94.9 | 6.23 |
| 1525 | A0A0R0LA35 | uncharacterized protein | 10 | -0.93 | 35.2 | 6.07 |
| 1526 | I1JZ21 | uncharacterized protein | 2 | -0.94 | 14.8 | 9.34 |
| 1527 | C6THE6 | uncharacterized protein | 5 | -0.94 | 24.2 | 9.1 |
| 1528 | A0A0R0HBF1 | uncharacterized protein | 5 | -0.94 | 25.3 | 5.08 |
| 1529 | I1KYL4 | er membrane protein complex subunit 3 | 4 | -0.95 | 27.8 | 9.01 |
| 1530 | A0A0R0FB78 | uncharacterized protein | 11 | -0.95 | 45.8 | 8.61 |
| 1531 | I1KAY3 | uncharacterized protein | 7 | -0.95 | 27.7 | 6.09 |
| 1532 | C6T4Z6 | uncharacterized protein | 6 | -0.95 | 15.9 | 10.18 |
| 1533 | I1K3G1 | uncharacterized protein | 9 | -0.95 | 51.4 | 8.1 |
| 1534 | A0A0R0FC11 | uncharacterized protein | 2 | -0.95 | 52 | 9.32 |
| 1535 | I1LE41 | uncharacterized protein | 9 | -0.95 | 26.2 | 4.75 |
| 1536 | C6SXD3 | 40s ribosomal protein s24 | 3 | -0.95 | 15.8 | 10.64 |
| 1537 | C6T8C4 | phosphomannomutase | 8 | -0.96 | 28 | 5.84 |
| 1538 | I1M0W3 | uncharacterized protein | 4 | -0.96 | 70.9 | 5.16 |
| 1539 | A0A0R0EVI2 | uncharacterized protein | 3 | -0.96 | 30.9 | 8.85 |
| 1540 | Q70MR6 | ornithine decarboxylase | 3 | -0.96 | 46.4 | 5.31 |
| 1541 | I1JKM1 | uncharacterized protein | 9 | -0.97 | 32.3 | 4.98 |
| 1542 | I1ME83 | ubiquitin thioesterase | 7 | -0.97 | 34 | 4.68 |
| 1543 | C6T588 | uncharacterized protein | 8 | -0.97 | 16.8 | 4.73 |
| 1544 | C6SWW6 | uncharacterized protein | 2 | -0.98 | 11.6 | 9.37 |
| 1545 | K7KYH7 | uncharacterized protein | 9 | -0.98 | 80.1 | 7.36 |
| 1546 | I1LRQ2 | nedd8-activating enzyme e1 regulatory subunit | 8 | -0.98 | 57.9 | 5.1 |
| 1547 | C6TGT0 | coatomer subunit epsilon | 11 | -0.98 | 32.3 | 5.3 |
| 1548 | C6TLE0 | uncharacterized protein | 9 | -0.98 | 28.1 | 6.3 |
| 1549 | I1LY26 | uncharacterized protein | 5 | -0.98 | 173.1 | 8.69 |
| 1550 | I1K754 | bifunctional dihydrofolate reductase-thymidylate synthase | 5 | -0.98 | 64.1 | 7.21 |
| 1551 | C6T288 | signal recognition particle 9 kda protein | 2 | -0.99 | 12.1 | 8.87 |
| 1552 | I1LMC1 | uncharacterized protein | 5 | -0.99 | 31.7 | 11.42 |
| 1553 | I1LWT2 | uncharacterized protein | 4 | -1 | 13.5 | 9.45 |
| 1554 | K7MAP3 | uncharacterized protein | 6 | -1 | 29.7 | 9.34 |
| 1555 | I1LUM3 | uncharacterized protein | 12 | -1 | 29.3 | 4.76 |
| 1556 | A0A0R0FDT6 | uncharacterized protein | 17 | -1.01 | 40.1 | 5.61 |
| 1557 | K7LDT9 | uncharacterized protein | 24 | -1.01 | 103.3 | 6.26 |
| 1558 | A0A0R0LB64 | uncharacterized protein | 12 | -1.01 | 34.2 | 9.38 |
| 1559 | C6SVT2 | profilin | 3 | -1.02 | 14 | 4.87 |
| 1560 | I1MAE6 | uncharacterized protein | 13 | -1.02 | 40.8 | 6.07 |
| 1561 | I1K5K8 | uncharacterized protein | 6 | -1.02 | 35.2 | 5.59 |
| 1562 | A0A0R0FH00 | uncharacterized protein | 30 | -1.03 | 71.2 | 5.09 |
| 1563 | I1JI26 | delta-1-pyrroline-5-carboxylate synthase | 12 | -1.03 | 77.5 | 6.37 |
| 1564 | I1JRT9 | uncharacterized protein | 7 | -1.04 | 80.6 | 5.42 |
| 1565 | C6T1X7 | uncharacterized protein | 2 | -1.04 | 12.5 | 9.94 |
| 1566 | C6TBI1 | uncharacterized protein | 9 | -1.05 | 31.6 | 5.37 |
| 1567 | C6KHU4 | phenylalanine ammonia-lyase | 16 | -1.05 | 78.1 | 5.83 |
| 1568 | I1MDJ2 | uncharacterized protein | 5 | -1.05 | 17.3 | 10.21 |
| 1569 | C6T827 | pyruvate dehydrogenase e1 component subunit beta | 10 | -1.05 | 38.6 | 5.7 |
| 1570 | Q9SEK9 | seed maturation protein pm25 | 9 | -1.06 | 25.7 | 4.99 |
| 1571 | I1L007 | uncharacterized protein | 5 | -1.06 | 39 | 8.73 |
| 1572 | C6T736 | uncharacterized protein | 3 | -1.06 | 24.7 | 9.52 |
| 1573 | I1KYX9 | uncharacterized protein | 6 | -1.06 | 17.5 | 7.79 |
| 1574 | C6T3P3 | uncharacterized protein | 4 | -1.06 | 24.3 | 8.35 |
| 1575 | C6T4D3 | uncharacterized protein | 2 | -1.06 | 12.1 | 7.55 |
| 1576 | A0A0R4J629 | uncharacterized protein | 3 | -1.07 | 25.5 | 6.01 |
| 1577 | A0A0R0JU00 | uncharacterized protein | 2 | -1.07 | 30.7 | 6.66 |
| 1578 | C6TJF9 | uncharacterized protein | 9 | -1.08 | 24 | 6.2 |
| 1579 | I1L3I1 | uncharacterized protein | 6 | -1.08 | 33.2 | 4.89 |
| 1580 | I1N0P0 | uncharacterized protein | 3 | -1.08 | 12.8 | 9.12 |
| 1581 | I1KVH4 | uncharacterized protein | 20 | -1.08 | 124.6 | 6.65 |
| 1582 | K7LSC0 | uncharacterized protein | 3 | -1.09 | 41.8 | 5.14 |
| 1583 | I1ML01 | uncharacterized protein | 4 | -1.09 | 69.6 | 6.19 |
| 1584 | I1JT75 | thioredoxin | 6 | -1.1 | 13.1 | 5.24 |
| 1585 | C6SZZ3 | uncharacterized protein | 2 | -1.1 | 15.7 | 5.29 |
| 1586 | I1N1F9 | uncharacterized protein | 22 | -1.1 | 58.3 | 6.69 |
| 1587 | A0A0R0IZU0 | uncharacterized protein | 14 | -1.1 | 37.1 | 5.3 |
| 1588 | K7LTN0 | uncharacterized protein | 12 | -1.11 | 66.3 | 6.25 |
| 1589 | I1M923 | glycine cleavage system p protein | 17 | -1.11 | 114.9 | 7.2 |
| 1590 | I1KQ26 | uncharacterized protein | 13 | -1.11 | 54.4 | 6 |
| 1591 | I1KQE3 | uncharacterized protein | 8 | -1.11 | 17.4 | 10.21 |
| 1592 | I1MEH7 | uncharacterized protein | 4 | -1.11 | 27.9 | 5.56 |
| 1593 | C6TJM1 | fructose-bisphosphate aldolase | 5 | -1.12 | 38.5 | 7.57 |
| 1594 | C6T0H9 | uncharacterized protein | 6 | -1.12 | 15 | 10.4 |
| 1595 | C6T7X6 | signal peptidase i | 4 | -1.12 | 20 | 7.02 |
| 1596 | A0A0R4J581 | 40s ribosomal protein s4 | 16 | -1.12 | 30 | 10.27 |
| 1597 | K7LB21 | uncharacterized protein | 2 | -1.13 | 66.9 | 6.76 |
| 1598 | C6T0R5 | uncharacterized protein | 5 | -1.13 | 21 | 11.18 |
| 1599 | A0A0R0IH13 | uncharacterized protein | 2 | -1.13 | 15 | 8.46 |
| 1600 | C6SVL7 | uncharacterized protein | 2 | -1.14 | 17.3 | 5.37 |
| 1601 | I1K3L8 | uncharacterized protein | 7 | -1.14 | 24.9 | 5.62 |
| 1602 | I1KG22 | uncharacterized protein | 8 | -1.15 | 84.8 | 5.64 |
| 1603 | I1JG42 | uncharacterized protein | 4 | -1.15 | 62 | 4.77 |
| 1604 | I1L2I7 | serine/threonine-protein phosphatase | 8 | -1.15 | 35.8 | 5.21 |
| 1605 | C6SYC1 | ATP synthase subunit d | 10 | -1.15 | 19.9 | 5.2 |
| 1606 | C6THL0 | uncharacterized protein | 2 | -1.16 | 36.2 | 7.29 |
| 1607 | C6T5S4 | uncharacterized protein | 3 | -1.17 | 17.6 | 6.11 |
| 1608 | I1MJ34 | tau class glutathione s-transferase | 8 | -1.17 | 25.5 | 6.23 |
| 1609 | I1L3U2 | uncharacterized protein | 6 | -1.17 | 28.5 | 7.51 |
| 1610 | I1KJM6 | uncharacterized protein | 7 | -1.17 | 55.2 | 8.15 |
| 1611 | E9KNA7 | sgf14e | 9 | -1.18 | 29.5 | 4.83 |
| 1612 | A0A0R0I7Q2 | uncharacterized protein | 13 | -1.18 | 127.8 | 5.47 |
| 1613 | A0A0R0E480 | uncharacterized protein | 3 | -1.18 | 37.1 | 9.15 |
| 1614 | P02580 | actin-3 | 10 | -1.18 | 41.6 | 5.23 |
| 1615 | C6SVD0 | uncharacterized protein | 2 | -1.18 | 12 | 5.29 |
| 1616 | I1KJD9 | ump-cmp kinase | 8 | -1.18 | 26.5 | 8.56 |
| 1617 | C6T0E8 | 60s ribosomal protein l18a | 7 | -1.19 | 21.3 | 10.44 |
| 1618 | A0A0R4J4G9 | uncharacterized protein | 3 | -1.19 | 23.7 | 6.46 |
| 1619 | I1ML54 | calcium load-activated calcium channel | 3 | -1.2 | 21.1 | 9.76 |
| 1620 | C6T922 | uncharacterized protein | 2 | -1.2 | 22.5 | 5.88 |
| 1621 | C6T8I2 | uncharacterized protein | 9 | -1.2 | 43.6 | 5.54 |
| 1622 | I1LVC1 | uncharacterized protein | 5 | -1.2 | 14.9 | 5.54 |
| 1623 | C6SY85 | uncharacterized protein | 11 | -1.2 | 22.6 | 5.86 |
| 1624 | A0A0R0I4T4 | uncharacterized protein | 7 | -1.2 | 31.7 | 7.7 |
| 1625 | A0A0R4J387 | chalcone-flavonone isomerase family protein | 10 | -1.21 | 23.5 | 4.96 |
| 1626 | C6TIZ1 | uncharacterized protein | 2 | -1.21 | 35.7 | 9 |
| 1627 | C6T4I7 | ribosomal protein l19 | 2 | -1.22 | 24.6 | 11.45 |
| 1628 | I1N8I3 | uncharacterized protein | 2 | -1.24 | 30.2 | 5.49 |
| 1629 | I1JZM5 | uncharacterized protein | 3 | -1.24 | 51.5 | 6.29 |
| 1630 | I1KXZ9 | uncharacterized protein | 5 | -1.25 | 43.2 | 5.36 |
| 1631 | I1LI50 | uncharacterized protein | 5 | -1.25 | 32.7 | 8.59 |
| 1632 | I1M5S3 | nuclear pore protein | 4 | -1.25 | 95.8 | 6.37 |
| 1633 | I1KNG0 | uncharacterized protein | 9 | -1.26 | 55.6 | 6.3 |
| 1634 | I1JLC8 | protein sle2 | 4 | -1.26 | 11.5 | 5.53 |
| 1635 | K7LKS0 | uncharacterized protein | 6 | -1.26 | 121.7 | 5.29 |
| 1636 | I1L810 | uncharacterized protein | 5 | -1.27 | 55.2 | 4.41 |
| 1637 | A0A0R4J3P1 | glutamine synthetase | 12 | -1.27 | 39.1 | 5.32 |
| 1638 | C6K8D1 | seed biotinylated protein 68 kda isoform | 15 | -1.27 | 67.9 | 6.18 |
| 1639 | I1LQ46 | coatomer subunit gamma | 33 | -1.29 | 98.7 | 5.05 |
| 1640 | I1KC24 | uncharacterized protein | 3 | -1.3 | 25 | 5.19 |
| 1641 | I1JK63 | peroxidase | 2 | -1.3 | 34.6 | 8.84 |
| 1642 | K7LZJ0 | uncharacterized protein | 6 | -1.3 | 16.6 | 10.53 |
| 1643 | A0A0R4J4D0 | uncharacterized protein | 10 | -1.3 | 23 | 5.09 |
| 1644 | I1NB44 | uncharacterized protein | 10 | -1.31 | 31.5 | 7.72 |
| 1645 | C6T863 | uncharacterized protein | 3 | -1.31 | 37.3 | 5.04 |
| 1646 | A0A0R0I7I9 | uncharacterized protein | 5 | -1.32 | 44.8 | 8.9 |
| 1647 | A0A0R4J318 | peptidyl-prolyl cis-trans isomerase | 6 | -1.32 | 18.9 | 7.69 |
| 1648 | I1KWI0 | uncharacterized protein | 17 | -1.32 | 40.1 | 5.61 |
| 1649 | I1LY84 | uncharacterized protein | 5 | -1.33 | 26.5 | 8.57 |
| 1650 | I1KQ72 | uncharacterized protein | 7 | -1.33 | 15 | 9.12 |
| 1651 | I1JQI7 | uncharacterized protein | 6 | -1.34 | 90.9 | 5.99 |
| 1652 | C6TLH8 | uncharacterized protein | 3 | -1.34 | 43.5 | 7.72 |
| 1653 | C6TMB5 | uncharacterized protein | 6 | -1.35 | 17.8 | 9.06 |
| 1654 | A0A0R0FUC5 | uncharacterized protein | 3 | -1.35 | 21.4 | 5.16 |
| 1655 | C6T7E3 | uncharacterized protein | 3 | -1.36 | 32.7 | 6.15 |
| 1656 | C6TH20 | uncharacterized protein | 8 | -1.36 | 39.4 | 5.96 |
| 1657 | I1N5Z6 | uncharacterized protein | 3 | -1.36 | 158.7 | 5.49 |
| 1658 | I1M3M0 | 14-3-3-like protein d | 14 | -1.36 | 29.4 | 4.8 |
| 1659 | K7LIT1 | uncharacterized protein | 2 | -1.37 | 111.5 | 4.71 |
| 1660 | K7MZ32 | uncharacterized protein | 3 | -1.37 | 18 | 8.3 |
| 1661 | I1KR99 | uncharacterized protein | 5 | -1.38 | 37.4 | 8.13 |
| 1662 | I1MHX4 | uncharacterized protein | 2 | -1.4 | 61.7 | 5.52 |
| 1663 | C6SY33 | uncharacterized protein | 4 | -1.4 | 25.9 | 8.68 |
| 1664 | C6SYI8 | uncharacterized protein | 9 | -1.4 | 18.4 | 5.94 |
| 1665 | I1LPN7 | uncharacterized protein | 6 | -1.4 | 18.8 | 6.28 |
| 1666 | A0A0R4J5R0 | uncharacterized protein | 4 | -1.4 | 37 | 5.6 |
| 1667 | K7LEQ5 | uncharacterized protein | 10 | -1.41 | 26.6 | 6.29 |
| 1668 | I1LH45 | uncharacterized protein | 3 | -1.41 | 51.8 | 4.34 |
| 1669 | I1L602 | uncharacterized protein | 9 | -1.41 | 29.7 | 8.57 |
| 1670 | I1LGR6 | uncharacterized protein | 31 | -1.41 | 127.3 | 6.28 |
| 1671 | I1L2H4 | uncharacterized protein | 9 | -1.42 | 22.7 | 9.51 |
| 1672 | I1L171 | uncharacterized protein | 13 | -1.42 | 46 | 5.89 |
| 1673 | I1JNK9 | uncharacterized protein | 17 | -1.42 | 41.6 | 5.31 |
| 1674 | C6TCN3 | uncharacterized protein | 3 | -1.43 | 32.3 | 5.91 |
| 1675 | A0A0R0E5C1 | carboxypeptidase | 8 | -1.44 | 49.3 | 6.13 |
| 1676 | H2BER4 | 4-coumarate:coa ligase | 4 | -1.44 | 58.7 | 9 |
| 1677 | I1MY38 | uncharacterized protein | 28 | -1.44 | 93.2 | 4.83 |
| 1678 | A0A0R4J653 | uncharacterized protein | 13 | -1.45 | 80 | 6.03 |
| 1679 | I1LIQ3 | uncharacterized protein | 3 | -1.46 | 28.1 | 4.76 |
| 1680 | C6SW13 | uncharacterized protein | 2 | -1.47 | 15.4 | 8.69 |
| 1681 | I1JZI4 | uncharacterized protein | 4 | -1.48 | 28.2 | 4.78 |
| 1682 | I1JL98 | uncharacterized protein | 6 | -1.48 | 18.5 | 5.22 |
| 1683 | C6TDZ0 | glutamate dehydrogenase | 15 | -1.48 | 44.5 | 5.97 |
| 1684 | C6SVN5 | uncharacterized protein | 2 | -1.5 | 19.3 | 8.89 |
| 1685 | A0A0R0IEK0 | uncharacterized protein | 8 | -1.5 | 50.6 | 6.91 |
| 1686 | I1L6V1 | uncharacterized protein | 4 | -1.51 | 47.1 | 8.61 |
| 1687 | I1J4V3 | uncharacterized protein | 3 | -1.51 | 18.8 | 5.48 |
| 1688 | I1L340 | uncharacterized protein | 3 | -1.52 | 30.3 | 4.88 |
| 1689 | I1KQX7 | uncharacterized protein | 2 | -1.53 | 9.9 | 4.91 |
| 1690 | I1K811 | uncharacterized protein | 2 | -1.54 | 51.3 | 6.04 |
| 1691 | K7KEG4 | uncharacterized protein | 7 | -1.55 | 54.8 | 8.38 |
| 1692 | C6SX35 | uncharacterized protein | 4 | -1.55 | 25.6 | 5.44 |
| 1693 | C6TG68 | uncharacterized protein | 6 | -1.56 | 16.6 | 4.2 |
| 1694 | C6T3A2 | uncharacterized protein | 5 | -1.56 | 16.7 | 4.8 |
| 1695 | A0A0R0GIU8 | chorismate mutase | 5 | -1.57 | 32.1 | 5.79 |
| 1696 | O04874 | glutathione transferase | 7 | -1.58 | 25 | 5.76 |
| 1697 | I1MDT4 | uncharacterized protein | 14 | -1.58 | 41.6 | 5.31 |
| 1698 | I1K6P4 | uncharacterized protein | 4 | -1.58 | 27.1 | 5.44 |
| 1699 | I1LRD2 | uncharacterized protein | 5 | -1.59 | 12.8 | 6.03 |
| 1700 | C6SYC3 | uncharacterized protein | 2 | -1.61 | 18.7 | 5 |
| 1701 | A0A0R0IF33 | uncharacterized protein | 16 | -1.61 | 61.6 | 4.77 |
| 1702 | I1NH22 | uncharacterized protein | 2 | -1.62 | 71.2 | 5.7 |
| 1703 | C6TLB6 | uncharacterized protein | 11 | -1.62 | 46.3 | 5.91 |
| 1704 | C6TJF6 | annexin | 18 | -1.62 | 35.8 | 7.11 |
| 1705 | I1KC70 | glyceraldehyde-3-phosphate dehydrogenase | 21 | -1.63 | 36.7 | 6.72 |
| 1706 | A0A0R0GWU7 | uncharacterized protein | 4 | -1.63 | 29.3 | 9.57 |
| 1707 | I1LQ57 | dolichyl-diphosphooligosaccharide--protein glycosyltransferase 48 kda subunit | 13 | -1.63 | 48.2 | 5.92 |
| 1708 | I1L3U0 | uncharacterized protein | 5 | -1.63 | 23.6 | 8.96 |
| 1709 | I1K9M3 | uncharacterized protein | 14 | -1.64 | 73 | 5.11 |
| 1710 | Q9ZTY1 | 35 kda seed maturation protein | 7 | -1.64 | 35.3 | 5.96 |
| 1711 | C6SXM8 | ribosomal protein l19 | 2 | -1.66 | 24.6 | 11.45 |
| 1712 | I1N2Z5 | protein sle1 | 2 | -1.66 | 12.2 | 5.33 |
| 1713 | I1K0C8 | uncharacterized protein | 6 | -1.66 | 65.7 | 4.64 |
| 1714 | C6SX10 | mitochondrial fission 1 protein | 4 | -1.66 | 18.7 | 6.96 |
| 1715 | C6TK57 | uncharacterized protein | 5 | -1.67 | 36.2 | 7.05 |
| 1716 | I1N036 | proliferating cell nuclear antigen | 9 | -1.67 | 29.5 | 4.68 |
| 1717 | I1K8D2 | uncharacterized protein | 2 | -1.68 | 28.1 | 9.87 |
| 1718 | I1L8Q7 | uncharacterized protein | 2 | -1.69 | 41.3 | 5.06 |
| 1719 | I1LPR5 | uncharacterized protein | 5 | -1.71 | 81.9 | 9.17 |
| 1720 | K7LPZ3 | uncharacterized protein | 2 | -1.73 | 8.8 | 4.27 |
| 1721 | C6TBA0 | uncharacterized protein | 5 | -1.74 | 30.4 | 9.69 |
| 1722 | I1KXH0 | uncharacterized protein | 8 | -1.76 | 36.6 | 4.83 |
| 1723 | C6SVC0 | uncharacterized protein | 5 | -1.78 | 23.9 | 6.26 |
| 1724 | I1KA95 | uncharacterized protein | 4 | -1.79 | 76 | 7.84 |
| 1725 | I1M0R3 | uncharacterized protein | 6 | -1.8 | 50.6 | 9.62 |
| 1726 | A0A0R0G7B5 | uncharacterized protein | 6 | -1.81 | 61.6 | 5.16 |
| 1727 | I1M228 | eukaryotic translation initiation factor 3 subunit d | 14 | -1.81 | 63.7 | 5.47 |
| 1728 | K7K8T8 | uncharacterized protein | 6 | -1.83 | 21 | 9.4 |
| 1729 | I1MRI4 | uncharacterized protein | 7 | -1.84 | 59.9 | 5.83 |
| 1730 | A0A0R0KSG7 | ribosomal protein l19 | 2 | -1.85 | 24.7 | 11.39 |
| 1731 | I1KRB5 | uncharacterized protein | 9 | -1.88 | 58.8 | 8.91 |
| 1732 | A0A0R0KFE8 | chlorophyll a-b binding protein | 2 | -1.89 | 31.4 | 5.9 |
| 1733 | A0A0R0EIR6 | glycosyltransferase | 8 | -1.93 | 54.4 | 5.6 |
| 1734 | I1L0D9 | peroxidase | 7 | -1.93 | 38 | 6.58 |
| 1735 | C6SX76 | uncharacterized protein | 3 | -1.94 | 12.6 | 7.77 |
| 1736 | C6SV88 | peptidylprolyl isomerase | 5 | -1.95 | 15.9 | 5.94 |
| 1737 | C6TF76 | uncharacterized protein | 3 | -1.96 | 11.2 | 7.9 |
| 1738 | K7KGC5 | pectinesterase | 2 | -1.98 | 61 | 8.92 |
| 1739 | B2BF98 | 40s ribosomal protein s6 | 5 | -1.99 | 28 | 10.71 |
| 1740 | C6SXA4 | trafficking protein particle complex subunit | 4 | -1.99 | 21 | 4.28 |
| 1741 | C6TJL2 | uncharacterized protein | 16 | -2.01 | 44.1 | 5.44 |
| 1742 | K7MPZ4 | uncharacterized protein | 2 | -2.01 | 15.7 | 5.29 |
| 1743 | I1LN34 | uncharacterized protein | 21 | -2.02 | 53.1 | 5.74 |
| 1744 | C6TMI3 | uncharacterized protein | 4 | -2.03 | 30.9 | 7.18 |
| 1745 | I1L7V6 | uncharacterized protein | 3 | -2.04 | 17.6 | 7.73 |
| 1746 | I1LKB2 | mitogen-activated protein kinase | 3 | -2.05 | 42.6 | 5.52 |
| 1747 | C6T294 | uncharacterized protein | 4 | -2.05 | 10.9 | 6.71 |
| 1748 | K7MPC2 | uncharacterized protein | 2 | -2.05 | 198 | 4.69 |
| 1749 | F8WRI3 | gamma-tocopherol methyltransferase | 4 | -2.06 | 33.3 | 6.33 |
| 1750 | M1FIU0 | ribosomal protein s3 | 2 | -2.06 | 65.1 | 10.48 |
| 1751 | I1MFX5 | endoglucanase | 6 | -2.08 | 68.3 | 8.81 |
| 1752 | I1LF63 | uncharacterized protein | 14 | -2.09 | 99.4 | 4.92 |
| 1753 | I1L691 | biotin carboxyl carrier protein of acetyl-coa carboxylase | 3 | -2.1 | 27.5 | 9.37 |
| 1754 | I1KU63 | uncharacterized protein | 32 | -2.11 | 247.2 | 5.58 |
| 1755 | I1LZP4 | uncharacterized protein | 2 | -2.14 | 146.7 | 6.58 |
| 1756 | C6TCU2 | uncharacterized protein | 3 | -2.15 | 41.7 | 6.68 |
| 1757 | K7MNL1 | uncharacterized protein | 2 | -2.17 | 29.9 | 5.78 |
| 1758 | A0A0R4J5G6 | uncharacterized protein | 6 | -2.18 | 25.6 | 6.16 |
| 1759 | I1KNA2 | uncharacterized protein | 7 | -2.18 | 49.5 | 5.44 |
| 1760 | I1K3Y1 | uncharacterized protein | 2 | -2.18 | 79.7 | 6.56 |
| 1761 | I1LJ15 | uncharacterized protein | 3 | -2.18 | 46.3 | 5.47 |
| 1762 | I1MTA6 | uncharacterized protein | 4 | -2.18 | 38.2 | 6.35 |
| 1763 | I1L8M8 | uncharacterized protein | 8 | -2.19 | 48 | 9.38 |
| 1764 | I1N7M0 | uncharacterized protein | 3 | -2.19 | 25.6 | 9.58 |
| 1765 | I1MB74 | uncharacterized protein | 2 | -2.2 | 92 | 6.16 |
| 1766 | I1LC43 | alpha-1,2-mannosidase | 2 | -2.2 | 69.6 | 5.05 |
| 1767 | I1KLI2 | clathrin light chain | 6 | -2.22 | 34.1 | 5.21 |
| 1768 | A0A0R0I1Y2 | uncharacterized protein | 5 | -2.22 | 42.6 | 6.11 |
| 1769 | I1JHT1 | glutamate decarboxylase | 9 | -2.23 | 57.2 | 5.27 |
| 1770 | I1LHP6 | uncharacterized protein | 10 | -2.24 | 81.7 | 5.29 |
| 1771 | I1LEB8 | pyruvate kinase | 3 | -2.24 | 63.4 | 5.43 |
| 1772 | C6SVE9 | uncharacterized protein | 3 | -2.25 | 20.4 | 11.41 |
| 1773 | I1KRP8 | uncharacterized protein | 5 | -2.25 | 52.3 | 5.75 |
| 1774 | C6SXP0 | uncharacterized protein | 2 | -2.27 | 15.2 | 8.76 |
| 1775 | C6TEQ1 | uncharacterized protein | 4 | -2.28 | 24.2 | 4.73 |
| 1776 | I1L6I1 | uncharacterized protein | 2 | -2.29 | 21.1 | 4.32 |
| 1777 | I1N3E1 | uncharacterized protein | 5 | -2.3 | 46.9 | 8.35 |
| 1778 | I1JKB3 | uncharacterized protein | 3 | -2.31 | 22 | 6.41 |
| 1779 | I1MGM0 | uncharacterized protein | 7 | -2.32 | 19 | 4.57 |
| 1780 | I1N6M1 | uncharacterized protein | 2 | -2.35 | 55.6 | 8.57 |
| 1781 | I1JC42 | uncharacterized protein | 6 | -2.36 | 28.9 | 9.03 |
| 1782 | C6TC08 | uncharacterized protein | 4 | -2.36 | 33.7 | 9.17 |
| 1783 | I1LE33 | uncharacterized protein | 8 | -2.36 | 68.2 | 5.94 |
| 1784 | I1KRI7 | 60s ribosomal protein l27 | 5 | -2.38 | 15.7 | 10.42 |
| 1785 | I1MRK1 | uncharacterized protein | 18 | -2.38 | 78.7 | 9.14 |
| 1786 | A0A0R0KJN1 | uncharacterized protein | 2 | -2.39 | 77.8 | 6.01 |
| 1787 | K7K9Y7 | uncharacterized protein | 2 | -2.39 | 9.8 | 9.41 |
| 1788 | A0A0R4J302 | uncharacterized protein | 16 | -2.4 | 49.5 | 5.91 |
| 1789 | A0A0R4J5B2 | uncharacterized protein | 6 | -2.4 | 14.8 | 5.34 |
| 1790 | Q9XES8 | seed maturation protein pm28 | 2 | -2.41 | 9.5 | 4.66 |
| 1791 | K7MND3 | uncharacterized protein | 3 | -2.43 | 11.4 | 9.16 |
| 1792 | K7KR21 | RNA cytidine acetyltransferase | 3 | -2.44 | 116.1 | 7.8 |
| 1793 | I1MJ53 | uncharacterized protein | 2 | -2.45 | 45.6 | 9.57 |
| 1794 | I1M8B3 | uncharacterized protein | 5 | -2.46 | 78.6 | 5.29 |
| 1795 | I1LF06 | coatomer subunit delta | 15 | -2.47 | 58.4 | 5.58 |
| 1796 | C6T6T6 | uncharacterized protein | 3 | -2.47 | 7.8 | 9.4 |
| 1797 | I1L146 | uncharacterized protein | 4 | -2.48 | 38 | 5.75 |
| 1798 | I1LYA4 | eukaryotic translation initiation factor 3 subunit k | 8 | -2.51 | 26.2 | 5.47 |
| 1799 | I1KLP7 | uncharacterized protein | 3 | -2.55 | 44 | 9.5 |
| 1800 | A0A0R4J5L2 | uncharacterized protein | 5 | -2.56 | 27.1 | 6.54 |
| 1801 | I1LGN1 | flavin-containing monooxygenase | 2 | -2.56 | 51.6 | 5.66 |
| 1802 | I1MD86 | glycosyltransferase | 2 | -2.56 | 53.7 | 5.5 |
| 1803 | K7K2V6 | uncharacterized protein | 4 | -2.58 | 113.3 | 4.72 |
| 1804 | C6TI18 | uncharacterized protein | 4 | -2.58 | 29.9 | 5.58 |
| 1805 | C6T9H6 | uncharacterized protein | 6 | -2.59 | 42.3 | 7.13 |
| 1806 | I1JJA3 | uncharacterized protein | 2 | -2.6 | 59.5 | 6.33 |
| 1807 | I1KW77 | uncharacterized protein | 2 | -2.63 | 37.7 | 5.12 |
| 1808 | A0A0R0LK87 | uncharacterized protein | 7 | -2.64 | 30.8 | 9.13 |
| 1809 | A0A0R0I960 | uncharacterized protein | 7 | -2.64 | 27 | 6.54 |
| 1810 | C6SW67 | uncharacterized protein | 2 | -2.64 | 17.4 | 5.16 |
| 1811 | C6TIW0 | fructose-bisphosphate aldolase | 10 | -2.66 | 42.5 | 8.73 |
| 1812 | I1J8W9 | uncharacterized protein | 10 | -2.72 | 43.2 | 6.17 |
| 1813 | I1LUW6 | uncharacterized protein | 2 | -2.74 | 52.5 | 8.6 |
| 1814 | I1KGY8 | uncharacterized protein | 6 | -2.76 | 56.1 | 5.78 |
| 1815 | A7BID0 | malonyl-coa:isoflavone 7-o-glucoside-6''-o-malonyltransferase | 7 | -2.78 | 51.2 | 6.07 |
| 1816 | I1JIE6 | uncharacterized protein | 3 | -2.79 | 67.9 | 8.78 |
| 1817 | I1JJT8 | uncharacterized protein | 18 | -2.8 | 41.6 | 6.16 |
| 1818 | I1MGN0 | uncharacterized protein | 2 | -2.8 | 13.6 | 7.84 |
| 1819 | I1L849 | uncharacterized protein | 8 | -2.8 | 27.4 | 5.16 |
| 1820 | A0A0R0HYT3 | uncharacterized protein | 9 | -2.81 | 24.2 | 5.49 |
| 1821 | I1KMW4 | uncharacterized protein | 2 | -2.83 | 50 | 8.24 |
| 1822 | I1NBR9 | uncharacterized protein | 3 | -2.84 | 19.4 | 6.82 |
| 1823 | I1LAH8 | uncharacterized protein | 3 | -2.85 | 23.9 | 5.69 |
| 1824 | I1MRP0 | uncharacterized protein | 2 | -2.87 | 62.4 | 8.58 |
| 1825 | I1K795 | uncharacterized protein | 2 | -2.87 | 39.5 | 5.51 |
| 1826 | C6SWK4 | uncharacterized protein | 3 | -2.88 | 17.8 | 10.15 |
| 1827 | I1K4R1 | uncharacterized protein | 9 | -2.9 | 25.8 | 10.18 |
| 1828 | I1KJF2 | uncharacterized protein | 4 | -2.9 | 109.4 | 5.67 |
| 1829 | K7M052 | uncharacterized protein | 12 | -2.92 | 117.9 | 6.04 |
| 1830 | I1LTV2 | uncharacterized protein | 2 | -2.92 | 38.6 | 6.28 |
| 1831 | C6TL75 | uncharacterized protein | 3 | -2.95 | 28.3 | 9.25 |
| 1832 | C6THJ6 | uncharacterized protein | 2 | -2.96 | 34.9 | 6.56 |
| 1833 | I1J5S9 | uncharacterized protein | 3 | -2.97 | 17.4 | 4.75 |
| 1834 | C6SYU5 | uncharacterized protein | 5 | -2.99 | 20.9 | 11.06 |
| 1835 | K7LEW3 | serine/threonine-protein phosphatase | 2 | -2.99 | 61.5 | 6.75 |
| 1836 | I1JYP3 | uncharacterized protein | 3 | -3.01 | 37.9 | 4.46 |
| 1837 | C6SVA4 | uncharacterized protein | 3 | -3.01 | 24.2 | 9.26 |
| 1838 | I1M0G8 | uncharacterized protein | 4 | -3.04 | 50.5 | 8.22 |
| 1839 | A0A0R0HHA4 | uncharacterized protein | 4 | -3.08 | 53.7 | 6.81 |
| 1840 | I1LQA8 | uncharacterized protein | 5 | -3.08 | 38.9 | 9.34 |
| 1841 | A0A0R0LCR8 | uncharacterized protein | 3 | -3.1 | 52.5 | 9.19 |
| 1842 | I1KJ86 | uncharacterized protein | 3 | -3.13 | 14.9 | 8.85 |
| 1843 | C6TCA2 | uncharacterized protein | 2 | -3.16 | 25.7 | 9.83 |
| 1844 | I1MEF8 | clathrin light chain | 5 | -3.17 | 35.3 | 5.08 |
| 1845 | I1LEH0 | uncharacterized protein | 2 | -3.18 | 24.4 | 5.42 |
| 1846 | K7M2H6 | uncharacterized protein | 4 | -3.2 | 20.1 | 5.41 |
| 1847 | A0A0R0GML1 | uncharacterized protein | 2 | -3.23 | 26 | 9.21 |
| 1848 | I1LNN8 | uncharacterized protein | 3 | -3.24 | 39.9 | 9.35 |
| 1849 | C6TNX5 | uncharacterized protein | 5 | -3.25 | 32.2 | 4.89 |
| 1850 | I1M6K8 | uncharacterized protein | 2 | -3.27 | 77.7 | 5.55 |
| 1851 | I1L860 | uncharacterized protein | 12 | -3.28 | 58 | 5.92 |
| 1852 | I1MNU2 | uncharacterized protein | 2 | -3.28 | 37.9 | 6.75 |
| 1853 | I1N8M4 | uncharacterized protein | 8 | -3.32 | 22 | 4.4 |
| 1854 | A0A0R0GEW3 | uncharacterized protein | 4 | -3.32 | 30.6 | 6.31 |
| 1855 | K7KTR9 | oleosin | 2 | -3.39 | 22.6 | 8.81 |
| 1856 | I1LQS4 | uncharacterized protein | 10 | -3.4 | 38.6 | 6.41 |
| 1857 | I1M544 | uncharacterized protein | 2 | -3.42 | 67.2 | 5.13 |
| 1858 | C6TIX1 | uncharacterized protein | 2 | -3.42 | 48.6 | 8.9 |
| 1859 | K7K4G2 | uncharacterized protein | 8 | -3.47 | 71.7 | 5.38 |
| 1860 | I1KJZ4 | uncharacterized protein | 4 | -3.48 | 52.5 | 5.11 |
| 1861 | I1KVT5 | uncharacterized protein | 2 | -3.53 | 15.5 | 5.76 |
| 1862 | I1NDC0 | uncharacterized protein | 4 | -3.53 | 35.7 | 4.71 |
| 1863 | A0A0R0ER52 | uncharacterized protein | 3 | -3.54 | 15.4 | 9.15 |
| 1864 | I1LXL0 | uncharacterized protein | 2 | -3.59 | 40.3 | 6.39 |
| 1865 | I1L090 | endoglucanase | 7 | -3.59 | 68.3 | 8.81 |
| 1866 | I1K9S0 | uncharacterized protein | 3 | -3.62 | 79.1 | 5.76 |
| 1867 | C6TJ53 | uncharacterized protein | 2 | -3.63 | 39.4 | 5.34 |
| 1868 | K7M352 | uncharacterized protein | 3 | -3.63 | 60.4 | 5.5 |
| 1869 | I1MC13 | uncharacterized protein | 13 | -3.66 | 56.1 | 9.11 |
| 1870 | I1KQX5 | uncharacterized protein | 13 | -3.69 | 89.4 | 6.36 |
| 1871 | I1MG34 | uncharacterized protein | 3 | -3.7 | 23.5 | 10.33 |
| 1872 | C6T9Q0 | uncharacterized protein | 4 | -3.71 | 27.6 | 6.65 |
| 1873 | I1KVZ0 | uncharacterized protein | 2 | -3.83 | 92.6 | 7.45 |
| 1874 | I1JSP6 | coatomer subunit gamma | 31 | -3.84 | 98.5 | 5.09 |
| 1875 | K7M834 | uncharacterized protein | 9 | -3.85 | 35.2 | 6.07 |
| 1876 | I1LZU4 | uncharacterized protein | 3 | -3.92 | 22.3 | 5.28 |
| 1877 | I1JHE4 | uncharacterized protein | 19 | -3.97 | 65.6 | 6.01 |
| 1878 | I1KN10 | uncharacterized protein | 13 | -3.98 | 86.7 | 6.66 |
| 1879 | K7N3W0 | uncharacterized protein | 2 | -4.04 | 27.2 | 8.57 |
| 1880 | K7M2A2 | uncharacterized protein | 2 | -4.05 | 77 | 6.51 |
| 1881 | C6T5E5 | uncharacterized protein | 3 | -4.08 | 24.1 | 6.24 |
| 1882 | K7MA29 | uncharacterized protein | 6 | -4.09 | 104.4 | 6.3 |
| 1883 | I1L7P6 | uncharacterized protein | 2 | -4.09 | 38.8 | 8.99 |
| 1884 | I1LKD3 | uncharacterized protein | 12 | -4.13 | 52.9 | 5.73 |
| 1885 | I1JYK1 | uncharacterized protein | 3 | -4.14 | 26 | 7.74 |
| 1886 | I1MQU2 | uncharacterized protein | 4 | -4.16 | 28.4 | 5.15 |
| 1887 | I1M9G4 | uncharacterized protein | 2 | -4.17 | 41.2 | 8.67 |
| 1888 | I1KI08 | uncharacterized protein | 2 | -4.2 | 16.4 | 5.09 |
| 1889 | I1J4K6 | uncharacterized protein | 4 | -4.31 | 86.5 | 9.29 |
| 1890 | Q9MAZ1 | nonclathrin coat protein zeta2-cop | 3 | -4.39 | 19.8 | 4.74 |
| 1891 | C6TJS1 | uncharacterized protein | 5 | -4.43 | 33.9 | 8.67 |
| 1892 | P29531 | p24 oleosin isoform b | 4 | -4.54 | 23.4 | 8.89 |
| 1893 | I1KLR6 | uncharacterized protein | 10 | -4.55 | 61.2 | 8.75 |
| 1894 | I1J7P1 | non-specific lipid-transfer protein | 5 | -4.55 | 11.8 | 9.18 |
| 1895 | I1LC49 | uncharacterized protein | 3 | -4.57 | 47.3 | 8.47 |
| 1896 | A0A0R0JM36 | uncharacterized protein | 3 | -4.6 | 12.5 | 8.57 |
| 1897 | A0A0R4J2I4 | uncharacterized protein | 14 | -4.63 | 65.6 | 5.62 |
| 1898 | A0A0R4J4Q5 | uncharacterized protein | 4 | -4.68 | 39.7 | 5.25 |
| 1899 | K7KTZ6 | malic enzyme | 9 | -4.7 | 68.3 | 6.91 |
| 1900 | C6TEW0 | uncharacterized protein | 4 | -4.76 | 40.6 | 5.42 |
| 1901 | K7MQ84 | uncharacterized protein | 18 | -4.77 | 65.2 | 6.25 |
| 1902 | A0A0R0JVY2 | xyloglucan endotransglucosylase/hydrolase | 3 | -4.8 | 32.3 | 9.07 |
| 1903 | I1N4F1 | uncharacterized protein | 2 | -4.86 | 53.3 | 6.97 |
| 1904 | I1LPL2 | uncharacterized protein | 12 | -4.93 | 29.4 | 10.21 |
| 1905 | A0A0R0J569 | uncharacterized protein | 3 | -5.07 | 46 | 7.67 |
| 1906 | K7M9V5 | uncharacterized protein | 11 | -5.09 | 55.5 | 6.05 |
| 1907 | A0A0R0H9B4 | uncharacterized protein | 6 | -5.15 | 11.9 | 5.66 |
| 1908 | I1KRN5 | uncharacterized protein | 4 | -5.16 | 64.5 | 6.19 |
| 1909 | I1MR60 | uncharacterized protein | 5 | -5.27 | 59.7 | 5.8 |
| 1910 | Q5F2M7 | pyruvate kinase | 17 | -5.31 | 54.3 | 6.8 |
| 1911 | I1JT46 | uncharacterized protein | 2 | -5.36 | 14.6 | 5.56 |
| 1912 | I1KZ32 | uncharacterized protein | 2 | -5.68 | 49.4 | 5 |
| 1913 | I1JUP4 | oleosin | 3 | -5.76 | 17.8 | 9.06 |
| 1914 | C6T1Q7 | uncharacterized protein | 2 | -5.89 | 17.8 | 5.99 |
| 1915 | A0A0R0F137 | uncharacterized protein | 5 | -6.01 | 25.5 | 11.05 |
| 1916 | C6TFY7 | uncharacterized protein | 3 | -6.07 | 10.6 | 7.99 |
| 1917 | O22518 | 40s ribosomal protein sa | 15 | -6.33 | 33.9 | 5.08 |
| 1918 | I1JC09 | uncharacterized protein | 2 | -6.55 | 90.3 | 6.62 |
| 1919 | C6SW35 | uncharacterized protein | 6 | -6.71 | 13.8 | 9.86 |

a “accession” is determined according to UniprotKB *Glycine* max (Soybean) protein database. b “M.P.” means the number of matched peptides. c “fold change” indicates log2 fold change of identified proteins from soybean treated with millimeter waves radiation compared to unirradiated soybean after flooding.

**Table S4**. List of changed proteins from unirradiated and unflooded soybean seedlings during growth.

| number | accessiona | description | M.P.b | fold changec | MW in kDa | pI |
| --- | --- | --- | --- | --- | --- | --- |
| 1 | I1J7P1 | non-specific lipid-transfer protein | 5 | 12.21 | 11.8 | 9.18 |
| 2 | A0A0R0JD77 | uncharacterized protein | 4 | 7.69 | 33.6 | 8.03 |
| 3 | O22518 | 40s ribosomal protein sa | 15 | 7.58 | 33.9 | 5.08 |
| 4 | C6SW35 | uncharacterized protein | 6 | 6.48 | 13.8 | 9.86 |
| 5 | C6SZH2 | uncharacterized protein | 6 | 6.41 | 17.5 | 4.52 |
| 6 | C6SZA9 | uncharacterized protein | 6 | 6.02 | 17.1 | 5.10 |
| 7 | A0A0R0H9B4 | uncharacterized protein | 6 | 5.96 | 11.9 | 5.66 |
| 8 | C6TFY7 | uncharacterized protein | 3 | 5.89 | 10.6 | 7.99 |
| 9 | A0A0R0GEW3 | uncharacterized protein | 4 | 5.86 | 30.6 | 6.31 |
| 10 | K7N3W0 | uncharacterized protein | 2 | 5.81 | 27.2 | 8.57 |
| 11 | A0A0R0G0Q7 | uncharacterized protein | 7 | 5.37 | 40.9 | 8.42 |
| 12 | G9I8U0 | inositol methyltransferase | 14 | 5.30 | 41.5 | 5.93 |
| 13 | A0A0R0F139 | uncharacterized protein | 6 | 5.27 | 39.3 | 6.86 |
| 14 | A0A0R0FTZ3 | uncharacterized protein | 7 | 5.01 | 51.0 | 8.88 |
| 15 | C6TJS1 | uncharacterized protein | 5 | 4.88 | 33.9 | 8.67 |
| 16 | P29531 | p24 oleosin isoform b | 4 | 4.79 | 23.4 | 8.89 |
| 17 | I1KVT1 | uncharacterized protein | 3 | 4.77 | 40.8 | 4.53 |
| 18 | A0A0R0ELP2 | uncharacterized protein | 2 | 4.72 | 43.7 | 8.70 |
| 19 | A0A0R4J2V8 | dirigent protein | 3 | 4.69 | 23.9 | 5.85 |
| 20 | I1LZU4 | uncharacterized protein | 3 | 4.69 | 22.3 | 5.28 |
| 21 | I1N4J8 | uncharacterized protein | 4 | 4.67 | 25.5 | 4.72 |
| 22 | C6TKJ2 | xyloglucan endotransglucosylase/hydrolase | 3 | 4.62 | 35.6 | 8.53 |
| 23 | I1LH06 | uncharacterized protein | 4 | 4.59 | 50.3 | 5.96 |
| 24 | K7L3S3 | uncharacterized protein | 4 | 4.58 | 410.5 | 5.16 |
| 25 | I1JE21 | glycosyltransferase | 2 | 4.52 | 54.2 | 5.31 |
| 26 | A0A0R0KFE8 | chlorophyll a-b binding protein | 2 | 4.51 | 31.4 | 5.90 |
| 27 | A0A0R0II00 | peptidylprolyl isomerase | 2 | 4.47 | 42.2 | 5.72 |
| 28 | I1LXL0 | uncharacterized protein | 2 | 4.45 | 40.3 | 6.39 |
| 29 | C6TEQ1 | uncharacterized protein | 4 | 4.43 | 24.2 | 4.73 |
| 30 | C6T085 | uncharacterized protein | 2 | 4.42 | 19.2 | 6.88 |
| 31 | C6T4K7 | uncharacterized protein | 2 | 4.41 | 20.3 | 5.14 |
| 32 | Q70EM0 | dehydrin | 9 | 4.38 | 23.8 | 5.97 |
| 33 | I1K8M1 | uncharacterized protein | 2 | 4.29 | 19.2 | 7.95 |
| 34 | C6T390 | uncharacterized protein | 5 | 4.26 | 16.2 | 5.66 |
| 35 | A0A0R0J324 | uncharacterized protein | 2 | 4.26 | 25.7 | 7.12 |
| 36 | I1KTK9 | uncharacterized protein | 2 | 4.19 | 47.9 | 8.77 |
| 37 | K7KQW5 | uncharacterized protein | 3 | 4.14 | 24.6 | 4.99 |
| 38 | I1LTU5 | uncharacterized protein | 4 | 4.13 | 48.5 | 7.04 |
| 39 | I1L0H6 | uncharacterized protein | 2 | 4.11 | 61.2 | 6.58 |
| 40 | I1KI08 | uncharacterized protein | 2 | 4.07 | 16.4 | 5.09 |
| 41 | C6SYT6 | uncharacterized protein | 2 | 4.07 | 7.6 | 8.09 |
| 42 | C6TJL7 | xyloglucan endotransglucosylase/hydrolase | 4 | 3.98 | 34.2 | 8.25 |
| 43 | I1N0P0 | uncharacterized protein | 3 | 3.97 | 12.8 | 9.12 |
| 44 | I1LTJ8 | uncharacterized protein | 4 | 3.95 | 95.0 | 4.58 |
| 45 | A5A339 | endonuclease | 3 | 3.94 | 33.5 | 5.92 |
| 46 | Q9XFI6 | peroxidase | 6 | 3.93 | 37.4 | 8.54 |
| 47 | C6T883 | uncharacterized protein | 3 | 3.93 | 45.0 | 5.14 |
| 48 | I1LBJ5 | zeta-carotene desaturase | 3 | 3.93 | 62.9 | 7.92 |
| 49 | K7MUM8 | uncharacterized protein | 4 | 3.89 | 38.0 | 5.07 |
| 50 | K7M834 | uncharacterized protein | 9 | 3.88 | 35.2 | 6.07 |
| 51 | I1L7P6 | uncharacterized protein | 2 | 3.85 | 38.8 | 8.99 |
| 52 | I1JSC6 | uncharacterized protein | 5 | 3.85 | 28.8 | 10.44 |
| 53 | I1M228 | eukaryotic translation initiation factor 3 subunit d | 14 | 3.83 | 63.7 | 5.47 |
| 54 | K7LPM5 | uncharacterized protein | 6 | 3.82 | 113.4 | 5.85 |
| 55 | Q2PMN3 | photosystem i iron-sulfur center | 3 | 3.81 | 9.0 | 6.65 |
| 56 | C6T1T3 | uncharacterized protein | 4 | 3.77 | 22.4 | 5.86 |
| 57 | C6SWJ2 | prefoldin subunit 3 | 6 | 3.76 | 21.0 | 4.76 |
| 58 | K7MVS5 | uncharacterized protein | 2 | 3.75 | 75.2 | 6.23 |
| 59 | A0A0R0KYU7 | xyloglucan endotransglucosylase/hydrolase | 9 | 3.71 | 36.0 | 7.18 |
| 60 | I1L0F6 | 3-hydroxyisobutyrate dehydrogenase | 2 | 3.70 | 37.0 | 7.55 |
| 61 | I1JY50 | uncharacterized protein | 3 | 3.70 | 19.7 | 9.04 |
| 62 | I1KZ20 | peptidylprolyl isomerase | 12 | 3.69 | 60.9 | 5.31 |
| 63 | I1KXP5 | uncharacterized protein | 7 | 3.69 | 99.0 | 5.87 |
| 64 | A0A0R0L372 | grpe protein homolog | 6 | 3.68 | 38.4 | 8.67 |
| 65 | I1N1R1 | uncharacterized protein | 4 | 3.67 | 16.8 | 4.64 |
| 66 | K7LDA5 | uncharacterized protein | 2 | 3.65 | 35.4 | 6.28 |
| 67 | C6SX14 | ferredoxin | 3 | 3.63 | 16.5 | 6.41 |
| 68 | I1MLU4 | uncharacterized protein | 8 | 3.63 | 38.8 | 7.10 |
| 69 | I1MGN0 | uncharacterized protein | 2 | 3.60 | 13.6 | 7.84 |
| 70 | I1LC43 | alpha-1,2-mannosidase | 2 | 3.58 | 69.6 | 5.05 |
| 71 | K7K4A7 | uncharacterized protein | 2 | 3.56 | 69.7 | 7.30 |
| 72 | I1K5L7 | 40s ribosomal protein s7 | 6 | 3.55 | 22.0 | 9.72 |
| 73 | I1JDS6 | beta-hexosaminidase | 11 | 3.54 | 62.9 | 5.54 |
| 74 | I1L0D9 | peroxidase | 7 | 3.53 | 38.0 | 6.58 |
| 75 | A0A0R0K0E5 | uncharacterized protein | 4 | 3.51 | 59.2 | 7.78 |
| 76 | Q2PMU9 | atp synthase epsilon chain | 3 | 3.48 | 14.7 | 5.41 |
| 77 | I1N1U5 | glycosyltransferase | 9 | 3.47 | 53.3 | 5.44 |
| 78 | C6TJ75 | peroxidase | 2 | 3.47 | 34.2 | 4.78 |
| 79 | K7MJN4 | uncharacterized protein | 2 | 3.46 | 40.0 | 6.19 |
| 80 | C6TBI8 | uncharacterized protein | 3 | 3.45 | 24.8 | 7.01 |
| 81 | I1KN30 | casp-like protein | 3 | 3.43 | 20.9 | 9.70 |
| 82 | I1LAK8 | uncharacterized protein | 2 | 3.42 | 64.2 | 5.08 |
| 83 | A0A0R0H261 | uncharacterized protein | 2 | 3.42 | 21.7 | 5.96 |
| 84 | Q2PMU3 | photosystem i p700 chlorophyll a apoprotein a1 | 2 | 3.42 | 83.4 | 6.74 |
| 85 | I1JHE4 | uncharacterized protein | 19 | 3.41 | 65.6 | 6.01 |
| 86 | C6TLM4 | chlorophyll a-b binding protein | 6 | 3.38 | 27.9 | 5.29 |
| 87 | A0A0R0HHA4 | uncharacterized protein | 4 | 3.37 | 53.7 | 6.81 |
| 88 | I1NDA1 | uncharacterized protein | 4 | 3.35 | 31.8 | 5.79 |
| 89 | C6T1V4 | nucleoside diphosphate kinase | 2 | 3.35 | 25.5 | 9.16 |
| 90 | C6TCK6 | uncharacterized protein | 3 | 3.33 | 25.8 | 6.36 |
| 91 | I1KZP9 | uncharacterized protein | 9 | 3.32 | 44.5 | 10.09 |
| 92 | I1JQR6 | dna helicase | 6 | 3.28 | 81.0 | 5.69 |
| 93 | C6TM81 | uncharacterized protein | 4 | 3.28 | 32.6 | 8.29 |
| 94 | I1KT48 | uncharacterized protein | 9 | 3.25 | 58.9 | 5.74 |
| 95 | I1KXV6 | uncharacterized protein | 3 | 3.25 | 64.8 | 9.69 |
| 96 | C6TEX2 | chlorophyll a-b binding protein | 3 | 3.24 | 29.8 | 8.60 |
| 97 | K7M5U1 | uncharacterized protein | 6 | 3.24 | 103.8 | 6.46 |
| 98 | I1M4T8 | uncharacterized protein | 8 | 3.23 | 23.8 | 6.74 |
| 99 | C6TLI1 | chlorophyll a-b binding protein | 3 | 3.22 | 27.4 | 6.91 |
| 100 | A0A0R0JM36 | uncharacterized protein | 3 | 3.22 | 12.5 | 8.57 |
| 101 | A0A0R0JG07 | uncharacterized protein | 2 | 3.19 | 49.3 | 5.72 |
| 102 | C6T7N2 | uncharacterized protein | 8 | 3.19 | 34.6 | 6.07 |
| 103 | I1K246 | uncharacterized protein | 9 | 3.18 | 62.1 | 5.60 |
| 104 | K7MXH4 | uncharacterized protein | 4 | 3.18 | 179.0 | 5.77 |
| 105 | I1JUP4 | oleosin | 3 | 3.18 | 17.8 | 9.06 |
| 106 | I1LR48 | uncharacterized protein | 2 | 3.16 | 27.2 | 5.48 |
| 107 | I1J7Z7 | elongation factor ts | 8 | 3.15 | 122.5 | 4.63 |
| 108 | A0A0R0JYG7 | uncharacterized protein | 2 | 3.13 | 76.1 | 6.09 |
| 109 | I1JDM5 | protein-lysine n-methyltransferase glyma_02g090500 | 7 | 3.12 | 37.5 | 4.50 |
| 110 | I1JT46 | uncharacterized protein | 2 | 3.11 | 14.6 | 5.56 |
| 111 | I1JJ22 | uncharacterized protein | 2 | 3.10 | 54.4 | 5.57 |
| 112 | I1M8P8 | uncharacterized protein | 3 | 3.09 | 52.7 | 8.04 |
| 113 | K7K6S5 | uncharacterized protein | 5 | 3.08 | 29.3 | 5.39 |
| 114 | I1LS52 | uncharacterized protein | 2 | 3.08 | 41.9 | 5.06 |
| 115 | C6SVC0 | uncharacterized protein | 5 | 3.07 | 23.9 | 6.26 |
| 116 | I1LJG3 | uncharacterized protein | 4 | 3.07 | 27.3 | 5.74 |
| 117 | K7KW51 | uncharacterized protein | 2 | 3.06 | 237.3 | 6.16 |
| 118 | I1KXF2 | uncharacterized protein | 9 | 3.02 | 18.4 | 6.05 |
| 119 | A0A0R0HW29 | uncharacterized protein | 7 | 3.02 | 47.7 | 6.18 |
| 120 | I1N4T2 | uncharacterized protein | 3 | 3.02 | 80.8 | 5.01 |
| 121 | I1L4Y5 | inositol-tetrakisphosphate 1-kinase | 2 | 3.00 | 39.4 | 7.60 |
| 122 | K7MP10 | uncharacterized protein | 2 | 3.00 | 118.6 | 4.97 |
| 123 | I1KIQ3 | uncharacterized protein | 2 | 3.00 | 70.8 | 8.35 |
| 124 | C6TLX4 | uncharacterized protein | 10 | 3.00 | 35.1 | 5.53 |
| 125 | I1NA25 | uncharacterized protein | 15 | 2.98 | 116.5 | 5.22 |
| 126 | A0A0R0HYB1 | uncharacterized protein | 9 | 2.97 | 51.6 | 8.58 |
| 127 | C6F122 | beta-galactosidase | 4 | 2.96 | 79.8 | 8.12 |
| 128 | C6THJ6 | uncharacterized protein | 2 | 2.95 | 34.9 | 6.56 |
| 129 | K7KID6 | uncharacterized protein | 2 | 2.94 | 24.4 | 8.93 |
| 130 | I1J753 | uncharacterized protein | 2 | 2.94 | 23.2 | 7.75 |
| 131 | C6SVE9 | uncharacterized protein | 3 | 2.93 | 20.4 | 11.41 |
| 132 | I1LZJ8 | uncharacterized protein | 6 | 2.91 | 51.1 | 5.37 |
| 133 | I1KAS7 | uncharacterized protein | 3 | 2.90 | 71.4 | 5.56 |
| 134 | I1N383 | uncharacterized protein | 4 | 2.88 | 68.6 | 5.50 |
| 135 | I1KGU0 | 40s ribosomal protein s12 | 8 | 2.87 | 15.1 | 5.50 |
| 136 | C6TLN0 | alpha-galactosidase | 5 | 2.87 | 46.9 | 5.96 |
| 137 | I1J919 | uncharacterized protein | 10 | 2.87 | 50.3 | 4.37 |
| 138 | I1LTV2 | uncharacterized protein | 2 | 2.85 | 38.6 | 6.28 |
| 139 | C6SYE7 | uncharacterized protein | 4 | 2.84 | 17.6 | 6.02 |
| 140 | A0A0R0G789 | uncharacterized protein | 18 | 2.83 | 56.1 | 9.12 |
| 141 | A0A0R0IS91 | uncharacterized protein | 6 | 2.79 | 27.9 | 9.05 |
| 142 | Q9FQF3 | glutathione s-transferase gst 5 | 4 | 2.78 | 26.4 | 5.10 |
| 143 | A0A0R4J3J0 | uncharacterized protein | 11 | 2.78 | 39.8 | 5.40 |
| 144 | I1KYC4 | uncharacterized protein | 6 | 2.76 | 51.9 | 8.43 |
| 145 | K7MH03 | uncharacterized protein | 3 | 2.75 | 24.7 | 7.47 |
| 146 | C6T7K0 | uncharacterized protein | 3 | 2.74 | 27.8 | 4.75 |
| 147 | I1MNR2 | xyloglucan endotransglucosylase/hydrolase | 8 | 2.74 | 25.4 | 6.31 |
| 148 | I1LWM2 | uncharacterized protein | 9 | 2.73 | 46.2 | 4.73 |
| 149 | C6T5Z9 | uncharacterized protein | 3 | 2.72 | 27.0 | 7.81 |
| 150 | A0A0R4J5A3 | uncharacterized protein | 6 | 2.72 | 55.4 | 7.60 |
| 151 | I1KXZ9 | uncharacterized protein | 5 | 2.71 | 43.2 | 5.36 |
| 152 | O48922 | cytochrome p450 98a2 | 2 | 2.71 | 57.6 | 8.58 |
| 153 | A0A0R4J2I4 | uncharacterized protein | 14 | 2.71 | 65.6 | 5.62 |
| 154 | I1MJ89 | uncharacterized protein | 2 | 2.71 | 41.8 | 5.02 |
| 155 | I1LPL2 | uncharacterized protein | 12 | 2.70 | 29.4 | 10.21 |
| 156 | C6TBY2 | uncharacterized protein | 3 | 2.69 | 17.7 | 5.62 |
| 157 | C6T4N0 | uncharacterized protein | 2 | 2.69 | 21.9 | 4.40 |
| 158 | C6THF2 | uncharacterized protein | 2 | 2.67 | 31.5 | 5.27 |
| 159 | I1LLS4 | uncharacterized protein | 6 | 2.66 | 37.4 | 7.59 |
| 160 | I1MR60 | uncharacterized protein | 5 | 2.64 | 59.7 | 5.80 |
| 161 | K7MPZ4 | uncharacterized protein | 2 | 2.62 | 15.7 | 5.29 |
| 162 | I1LPP3 | uncharacterized protein | 4 | 2.61 | 14.7 | 5.63 |
| 163 | K7MPC2 | uncharacterized protein | 2 | 2.61 | 198.0 | 4.69 |
| 164 | I1J8T1 | uncharacterized protein | 5 | 2.60 | 33.7 | 5.95 |
| 165 | I1J4V3 | uncharacterized protein | 3 | 2.59 | 18.8 | 5.48 |
| 166 | I1MB74 | uncharacterized protein | 2 | 2.59 | 92.0 | 6.16 |
| 167 | I1L327 | uncharacterized protein | 2 | 2.58 | 27.2 | 6.90 |
| 168 | Q9MAZ1 | nonclathrin coat protein zeta2-cop | 3 | 2.58 | 19.8 | 4.74 |
| 169 | K7LSD2 | uncharacterized protein | 16 | 2.57 | 102.5 | 5.14 |
| 170 | I1LQS4 | uncharacterized protein | 10 | 2.57 | 38.6 | 6.41 |
| 171 | I1N6Y7 | uncharacterized protein | 3 | 2.57 | 85.9 | 6.66 |
| 172 | I1J4K5 | uncharacterized protein | 3 | 2.56 | 19.4 | 4.83 |
| 173 | K7LPZ3 | uncharacterized protein | 2 | 2.56 | 8.8 | 4.27 |
| 174 | I1N036 | proliferating cell nuclear antigen | 9 | 2.56 | 29.5 | 4.68 |
| 175 | I1KYC6 | uncharacterized protein | 3 | 2.56 | 51.9 | 8.14 |
| 176 | A0A0R0IF33 | uncharacterized protein | 16 | 2.55 | 61.6 | 4.77 |
| 177 | K7LQ40 | uncharacterized protein | 2 | 2.55 | 39.4 | 8.70 |
| 178 | C6TI18 | uncharacterized protein | 4 | 2.54 | 29.9 | 5.58 |
| 179 | I1LBX5 | uncharacterized protein | 2 | 2.52 | 44.7 | 7.79 |
| 180 | B3GJZ6 | chilling-induced protein | 2 | 2.50 | 13.9 | 6.28 |
| 181 | C6TJ98 | uncharacterized protein | 7 | 2.50 | 27.6 | 4.76 |
| 182 | I1MG34 | uncharacterized protein | 3 | 2.48 | 23.5 | 10.33 |
| 183 | I1KPH2 | uncharacterized protein | 10 | 2.47 | 26.8 | 5.87 |
| 184 | I1N747 | oleosin | 5 | 2.43 | 23.6 | 8.89 |
| 185 | C6TCI6 | glutamate decarboxylase | 12 | 2.42 | 56.2 | 5.57 |
| 186 | C6SWA5 | 40s ribosomal protein s26 | 5 | 2.41 | 14.9 | 10.92 |
| 187 | K7LM54 | uncharacterized protein | 6 | 2.41 | 93.2 | 4.76 |
| 188 | I1JCY0 | chlorophyll a-b binding protein | 2 | 2.37 | 26.7 | 6.21 |
| 189 | I1JYM6 | uncharacterized protein | 4 | 2.37 | 50.5 | 6.34 |
| 190 | I1KLI2 | clathrin light chain | 6 | 2.37 | 34.1 | 5.21 |
| 191 | I1L6I1 | uncharacterized protein | 2 | 2.37 | 21.1 | 4.32 |
| 192 | K7MA29 | uncharacterized protein | 6 | 2.36 | 104.4 | 6.30 |
| 193 | C6SYK2 | uncharacterized protein | 4 | 2.35 | 22.4 | 4.91 |
| 194 | C6TEW0 | uncharacterized protein | 4 | 2.34 | 40.6 | 5.42 |
| 195 | I1MUF2 | uncharacterized protein | 8 | 2.34 | 282.2 | 5.78 |
| 196 | I1JY77 | uncharacterized protein | 2 | 2.33 | 40.8 | 4.70 |
| 197 | I1J8H7 | peroxidase | 3 | 2.33 | 34.4 | 9.35 |
| 198 | I1N349 | uncharacterized protein | 4 | 2.33 | 104.4 | 5.99 |
| 199 | I1LFG0 | uncharacterized protein | 4 | 2.31 | 34.6 | 6.60 |
| 200 | I1KW27 | uncharacterized protein | 2 | 2.31 | 55.2 | 7.81 |
| 201 | A0A0R4J4L1 | uncharacterized protein | 16 | 2.29 | 34.9 | 5.65 |
| 202 | I1LRP4 | uncharacterized protein | 9 | 2.28 | 60.2 | 6.65 |
| 203 | A0A0R0IMY2 | uncharacterized protein | 9 | 2.26 | 235.3 | 5.07 |
| 204 | I1KW85 | uncharacterized protein | 2 | 2.26 | 52.4 | 5.40 |
| 205 | C6TKX3 | uncharacterized protein | 2 | 2.25 | 27.2 | 5.98 |
| 206 | A0A0R4J629 | uncharacterized protein | 3 | 2.24 | 25.5 | 6.01 |
| 207 | I1M923 | glycine cleavage system p protein | 17 | 2.24 | 114.9 | 7.20 |
| 208 | I1LN34 | uncharacterized protein | 21 | 2.22 | 53.1 | 5.74 |
| 209 | A0A0R0EIR6 | glycosyltransferase | 8 | 2.21 | 54.4 | 5.60 |
| 210 | I1KY46 | uncharacterized protein | 4 | 2.21 | 52.3 | 8.67 |
| 211 | I1M4F8 | uncharacterized protein | 3 | 2.20 | 32.9 | 5.13 |
| 212 | C6TE99 | uncharacterized protein | 4 | 2.19 | 39.8 | 5.64 |
| 213 | I1N6M1 | uncharacterized protein | 2 | 2.19 | 55.6 | 8.57 |
| 214 | C6SVZ6 | uncharacterized protein | 3 | 2.19 | 12.4 | 8.69 |
| 215 | A0A0R4J559 | uncharacterized protein | 9 | 2.18 | 21.7 | 6.43 |
| 216 | C6T9R1 | uncharacterized protein | 4 | 2.18 | 42.4 | 4.79 |
| 217 | C6SWY6 | uncharacterized protein | 8 | 2.17 | 16.5 | 4.68 |
| 218 | Q2PMU2 | photosystem i p700 chlorophyll a apoprotein a2 | 8 | 2.16 | 82.4 | 6.80 |
| 219 | A0A0R4J645 | uncharacterized protein | 2 | 2.16 | 41.6 | 5.26 |
| 220 | I1MKF6 | uncharacterized protein | 3 | 2.15 | 16.3 | 5.29 |
| 221 | A0A0R0JNE0 | glycosyltransferase | 3 | 2.15 | 52.6 | 5.55 |
| 222 | Q2PMT8 | photosystem ii d2 protein | 5 | 2.15 | 39.5 | 5.33 |
| 223 | I1M9K9 | uncharacterized protein | 5 | 2.14 | 39.8 | 6.53 |
| 224 | I1LAY3 | uncharacterized protein | 15 | 2.13 | 101.4 | 4.22 |
| 225 | C6KXH6 | protein disulfide isomerase l-3b | 11 | 2.12 | 58.8 | 4.79 |
| 226 | C6TMX0 | xyloglucan endotransglucosylase/hydrolase | 8 | 2.11 | 34.4 | 9.12 |
| 227 | C6T6Y4 | uncharacterized protein | 4 | 2.10 | 31.2 | 9.31 |
| 228 | A0A0R4J338 | nadph--cytochrome p450 reductase | 6 | 2.09 | 76.5 | 5.25 |
| 229 | I1K5C5 | uncharacterized protein | 5 | 2.09 | 26.3 | 9.05 |
| 230 | K7MND3 | uncharacterized protein | 3 | 2.09 | 11.4 | 9.16 |
| 231 | I1JKE2 | uncharacterized protein | 5 | 2.08 | 67.0 | 8.93 |
| 232 | I1KQ26 | uncharacterized protein | 13 | 2.08 | 54.4 | 6.00 |
| 233 | I1M8D6 | uncharacterized protein | 4 | 2.07 | 51.2 | 4.98 |
| 234 | C6TLR5 | uncharacterized protein | 4 | 2.06 | 23.1 | 9.62 |
| 235 | I1ML46 | uncharacterized protein | 13 | 2.06 | 56.5 | 5.00 |
| 236 | I1M712 | oxygen-evolving enhancer protein 2 | 3 | 2.05 | 28.6 | 7.69 |
| 237 | I1KAB1 | uncharacterized protein | 10 | 2.05 | 47.1 | 7.13 |
| 238 | I1JP51 | uncharacterized protein | 3 | 2.04 | 53.5 | 9.76 |
| 239 | I1N4U1 | 6-phosphogluconate dehydrogenase | 23 | 2.04 | 53.6 | 5.68 |
| 240 | I1MQN7 | uncharacterized protein | 14 | 2.04 | 36.1 | 6.34 |
| 241 | I1K2X6 | uncharacterized protein | 9 | 2.03 | 59.5 | 9.09 |
| 242 | I1MYV4 | uncharacterized protein | 9 | 2.03 | 33.0 | 10.05 |
| 243 | A0A0R0HCH7 | uncharacterized protein | 4 | 2.03 | 58.7 | 5.59 |
| 244 | I1K850 | uncharacterized protein | 3 | 2.03 | 82.5 | 8.91 |
| 245 | C6TLI8 | uncharacterized protein | 8 | 2.02 | 25.9 | 10.20 |
| 246 | I1JGY5 | glucose-6-phosphate isomerase | 19 | 2.02 | 67.2 | 5.76 |
| 247 | I1K4A8 | uncharacterized protein | 6 | 2.01 | 38.9 | 5.22 |
| 248 | C6T1C8 | uncharacterized protein | 3 | 2.01 | 22.8 | 9.32 |
| 249 | Q2LAL4 | cytochrome p450 monooxygenase cyp83e8 | 7 | 2.00 | 57.4 | 8.20 |
| 250 | I1MIC0 | uncharacterized protein | 9 | 2.00 | 21.5 | 6.29 |
| 251 | C6T5E7 | uncharacterized protein | 4 | 2.00 | 24.6 | 6.84 |
| 252 | I1MRI4 | uncharacterized protein | 7 | 2.00 | 59.9 | 5.83 |
| 253 | K7MSK3 | uncharacterized protein | 2 | 1.99 | 51.0 | 5.27 |
| 254 | F8WRI3 | gamma-tocopherol methyltransferase | 4 | 1.98 | 33.3 | 6.33 |
| 255 | I1KJF2 | uncharacterized protein | 4 | 1.98 | 109.4 | 5.67 |
| 256 | I1J8R4 | peroxidase | 9 | 1.97 | 36.4 | 9.26 |
| 257 | C6TBA0 | uncharacterized protein | 5 | 1.97 | 30.4 | 9.69 |
| 258 | I1N8L7 | uncharacterized protein | 10 | 1.97 | 72.0 | 5.04 |
| 259 | I1JU62 | transmembrane 9 superfamily member | 8 | 1.96 | 73.1 | 7.17 |
| 260 | I1K3Y1 | uncharacterized protein | 2 | 1.95 | 79.7 | 6.56 |
| 261 | I1JLY3 | peptidylprolyl isomerase | 6 | 1.95 | 69.9 | 5.36 |
| 262 | I1JHQ6 | uncharacterized protein | 10 | 1.95 | 60.9 | 8.78 |
| 263 | I1MVS1 | uncharacterized protein | 4 | 1.94 | 46.2 | 6.16 |
| 264 | I1MY38 | uncharacterized protein | 28 | 1.94 | 93.2 | 4.83 |
| 265 | I1JFJ9 | uncharacterized protein | 11 | 1.93 | 31.9 | 6.51 |
| 266 | I1LDJ1 | pyruvate kinase | 12 | 1.93 | 63.7 | 7.31 |
| 267 | P15490 | stem 28 kda glycoprotein | 13 | 1.93 | 29.0 | 8.38 |
| 268 | I1JKS0 | pectinesterase | 9 | 1.91 | 51.8 | 8.53 |
| 269 | I1KUQ6 | lipoxygenase | 18 | 1.91 | 97.1 | 6.93 |
| 270 | K7KLY3 | uncharacterized protein | 5 | 1.91 | 54.9 | 5.79 |
| 271 | K7LEH0 | uncharacterized protein | 2 | 1.90 | 143.1 | 5.64 |
| 272 | K7N3L7 | uncharacterized protein | 2 | 1.90 | 27.8 | 8.82 |
| 273 | K7MTZ4 | uncharacterized protein | 3 | 1.90 | 41.0 | 6.44 |
| 274 | P10743 | stem 31 kda glycoprotein | 21 | 1.89 | 29.3 | 5.84 |
| 275 | Q43437 | chlorophyll a-b binding protein | 8 | 1.89 | 28.0 | 5.14 |
| 276 | A0A0R0IH13 | uncharacterized protein | 2 | 1.87 | 15.0 | 8.46 |
| 277 | Q8HVY4 | dna-directed rna polymerase subunit beta | 3 | 1.87 | 79.6 | 9.09 |
| 278 | I1JE93 | uncharacterized protein | 2 | 1.87 | 25.9 | 8.26 |
| 279 | C6TJ03 | uncharacterized protein | 2 | 1.87 | 36.9 | 5.62 |
| 280 | A0A0R4J389 | uncharacterized protein | 3 | 1.87 | 24.3 | 9.73 |
| 281 | C6SWP9 | uncharacterized protein | 4 | 1.85 | 24.8 | 9.85 |
| 282 | A0A0R0GFB2 | uncharacterized protein | 23 | 1.85 | 57.5 | 8.99 |
| 283 | I1LPA3 | uncharacterized protein | 6 | 1.85 | 37.8 | 8.08 |
| 284 | I1KU63 | uncharacterized protein | 32 | 1.84 | 247.2 | 5.58 |
| 285 | Q2PMT9 | photosystem ii cp43 reaction center protein | 5 | 1.84 | 51.9 | 6.34 |
| 286 | A0A0R4J3W6 | uncharacterized protein | 3 | 1.83 | 59.9 | 6.22 |
| 287 | I1M4P9 | uncharacterized protein | 3 | 1.83 | 40.6 | 8.70 |
| 288 | I1MRU5 | carboxypeptidase | 2 | 1.82 | 54.8 | 5.51 |
| 289 | I1M170 | glutamine synthetase | 7 | 1.81 | 47.6 | 6.42 |
| 290 | K7LVU7 | uncharacterized protein | 2 | 1.81 | 95.0 | 5.03 |
| 291 | A0A0R0K666 | uncharacterized protein | 9 | 1.80 | 47.0 | 8.34 |
| 292 | C6SVR0 | plastocyanin | 3 | 1.80 | 16.8 | 5.04 |
| 293 | I1LZT0 | purple acid phosphatase | 8 | 1.79 | 54.4 | 6.20 |
| 294 | B0M194 | peroxisomal 3-hydroxyacyl-coa dehydrogenase-like protein | 3 | 1.79 | 35.0 | 9.48 |
| 295 | C6T0C7 | uncharacterized protein | 7 | 1.79 | 23.4 | 4.73 |
| 296 | I1JKQ4 | arginase | 13 | 1.78 | 38.7 | 6.04 |
| 297 | C6TD43 | uncharacterized protein | 8 | 1.77 | 37.8 | 6.61 |
| 298 | I1KQX7 | uncharacterized protein | 2 | 1.77 | 9.9 | 4.91 |
| 299 | C6T3N5 | uncharacterized protein | 4 | 1.77 | 14.3 | 8.66 |
| 300 | C6SW13 | uncharacterized protein | 2 | 1.76 | 15.4 | 8.69 |
| 301 | I1LRN2 | uncharacterized protein | 3 | 1.75 | 64.3 | 5.98 |
| 302 | C6TM42 | uncharacterized protein | 2 | 1.75 | 34.0 | 6.49 |
| 303 | I1J870 | uncharacterized protein | 6 | 1.75 | 105.9 | 5.88 |
| 304 | C6SYW1 | uncharacterized protein | 6 | 1.74 | 17.0 | 4.99 |
| 305 | I1MCI8 | uncharacterized protein | 11 | 1.74 | 40.7 | 8.18 |
| 306 | K7MTC5 | laccase | 2 | 1.73 | 64.2 | 8.47 |
| 307 | A0A0R0KAK4 | uncharacterized protein | 2 | 1.72 | 44.0 | 7.56 |
| 308 | I1L4I8 | uncharacterized protein | 2 | 1.72 | 70.1 | 8.09 |
| 309 | A0A0R0JQA9 | uncharacterized protein | 2 | 1.72 | 21.5 | 6.85 |
| 310 | A0A0R0FVK4 | carboxypeptidase | 3 | 1.71 | 55.7 | 5.53 |
| 311 | C6TD35 | uncharacterized protein | 2 | 1.70 | 37.0 | 5.20 |
| 312 | A0A0R0HTR5 | uncharacterized protein | 16 | 1.70 | 125.8 | 6.24 |
| 313 | I1L1Q3 | uncharacterized protein | 3 | 1.70 | 45.1 | 6.56 |
| 314 | A0A0R4J598 | cysteine proteinase inhibitor | 3 | 1.70 | 12.4 | 8.94 |
| 315 | I1KPJ3 | endoglucanase | 4 | 1.69 | 68.7 | 8.81 |
| 316 | I1LLA1 | uncharacterized protein | 7 | 1.68 | 79.4 | 8.20 |
| 317 | C6T5A8 | uncharacterized protein | 2 | 1.67 | 7.5 | 10.44 |
| 318 | I1J9V8 | protein transport protein sec16 | 4 | 1.67 | 154.3 | 4.96 |
| 319 | K7KZJ7 | uncharacterized protein | 18 | 1.67 | 86.2 | 6.16 |
| 320 | C6TN86 | uncharacterized protein | 2 | 1.67 | 38.3 | 5.66 |
| 321 | A0A0R0I8Z5 | chlorophyll a-b binding protein | 5 | 1.67 | 31.0 | 5.72 |
| 322 | I1KDK5 | uncharacterized protein | 5 | 1.66 | 67.4 | 6.12 |
| 323 | I1MYQ1 | uncharacterized protein | 2 | 1.65 | 49.6 | 5.39 |
| 324 | I1MDV0 | uncharacterized protein | 7 | 1.65 | 83.8 | 5.97 |
| 325 | C6TNE6 | chlorophyll a-b binding protein | 8 | 1.64 | 27.9 | 5.29 |
| 326 | I1JTZ3 | uncharacterized protein | 3 | 1.64 | 58.2 | 5.80 |
| 327 | K7K8T8 | uncharacterized protein | 6 | 1.64 | 21.0 | 9.40 |
| 328 | Q2PMS9 | atp synthase subunit b | 3 | 1.63 | 21.1 | 8.55 |
| 329 | I1MCL4 | uncharacterized protein | 11 | 1.63 | 102.8 | 6.48 |
| 330 | I1LKM5 | uncharacterized protein | 9 | 1.63 | 39.3 | 9.43 |
| 331 | C6TMK9 | uncharacterized protein | 3 | 1.62 | 34.0 | 6.00 |
| 332 | A0A0R0G7B5 | uncharacterized protein | 6 | 1.62 | 61.6 | 5.16 |
| 333 | I1JYX6 | transcription elongation factor spt5 | 5 | 1.61 | 116.1 | 5.17 |
| 334 | I1MYV2 | uncharacterized protein | 6 | 1.61 | 78.0 | 4.62 |
| 335 | C6TAU4 | uncharacterized protein | 7 | 1.60 | 18.8 | 3.97 |
| 336 | I1LN47 | uncharacterized protein | 2 | 1.60 | 63.4 | 7.58 |
| 337 | I1L6V1 | uncharacterized protein | 4 | 1.59 | 47.1 | 8.61 |
| 338 | C6T0P2 | uncharacterized protein | 4 | 1.59 | 21.7 | 7.78 |
| 339 | I1K9B3 | uncharacterized protein | 4 | 1.59 | 48.2 | 8.85 |
| 340 | I1J5P9 | glycosyltransferase | 6 | 1.59 | 56.1 | 5.59 |
| 341 | I1J8V9 | succinate dehydrogenase | 17 | 1.58 | 69.3 | 6.20 |
| 342 | I1M0C6 | clathrin light chain | 5 | 1.58 | 33.5 | 5.22 |
| 343 | I1NC50 | uncharacterized protein | 4 | 1.58 | 134.0 | 5.95 |
| 344 | I1MFH3 | uncharacterized protein | 4 | 1.58 | 41.3 | 6.68 |
| 345 | C6T3S4 | uncharacterized protein | 2 | 1.58 | 26.8 | 6.41 |
| 346 | I1MQH3 | succinate--coa ligase | 7 | 1.58 | 33.9 | 8.98 |
| 347 | A0A0R0GPK2 | uncharacterized protein | 7 | 1.57 | 14.8 | 10.58 |
| 348 | I1LBH6 | uncharacterized protein | 18 | 1.57 | 50.5 | 4.87 |
| 349 | Q2PMQ5 | cytochrome b6 | 2 | 1.56 | 24.1 | 8.89 |
| 350 | I1LWC8 | uncharacterized protein | 3 | 1.56 | 13.1 | 5.13 |
| 351 | C6TJF6 | annexin | 18 | 1.55 | 35.8 | 7.11 |
| 352 | I1M3Z4 | uncharacterized protein | 5 | 1.55 | 26.8 | 5.05 |
| 353 | B2BF98 | 40s ribosomal protein s6 | 5 | 1.55 | 28.0 | 10.71 |
| 354 | A0A0R4J4T3 | peroxidase | 7 | 1.55 | 33.9 | 8.67 |
| 355 | I1LP98 | uncharacterized protein | 10 | 1.55 | 77.4 | 9.19 |
| 356 | A0A0R0GWU7 | uncharacterized protein | 4 | 1.54 | 29.3 | 9.57 |
| 357 | I1K4T0 | uncharacterized protein | 2 | 1.54 | 74.0 | 6.39 |
| 358 | I1JUN3 | uncharacterized protein | 6 | 1.53 | 44.1 | 7.27 |
| 359 | K7LNG2 | uncharacterized protein | 9 | 1.53 | 41.8 | 8.20 |
| 360 | I1JGU8 | phospho-2-dehydro-3-deoxyheptonate aldolase | 12 | 1.52 | 59.0 | 8.57 |
| 361 | I1MBI9 | peroxidase | 9 | 1.52 | 40.0 | 9.62 |
| 362 | I1LV30 | uncharacterized protein | 5 | 1.52 | 37.5 | 5.22 |
| 363 | I1K0G4 | uncharacterized protein | 2 | 1.52 | 20.6 | 7.62 |
| 364 | Q2TUV8 | anthocyanidin synthase 2 | 5 | 1.51 | 39.9 | 5.62 |
| 365 | I1JMX7 | uncharacterized protein | 5 | 1.51 | 49.0 | 8.37 |
| 366 | A0A0R0FGT9 | uncharacterized protein | 3 | 1.51 | 19.3 | 6.73 |
| 367 | A0A0R0KJN1 | uncharacterized protein | 2 | 1.51 | 77.8 | 6.01 |
| 368 | C6SXD0 | uncharacterized protein | 9 | 1.51 | 17.8 | 5.96 |
| 369 | C6TGL6 | uncharacterized protein | 3 | 1.50 | 39.7 | 9.77 |
| 370 | C6TC69 | uncharacterized protein | 3 | 1.50 | 42.0 | 5.91 |
| 371 | A0A0R4J302 | uncharacterized protein | 16 | 1.50 | 49.5 | 5.91 |
| 372 | I1MNK0 | uncharacterized protein | 9 | 1.49 | 35.0 | 6.66 |
| 373 | I1K4M2 | glycosyltransferase | 11 | 1.49 | 55.8 | 5.74 |
| 374 | A0A0R0GIC4 | uncharacterized protein | 2 | 1.49 | 34.0 | 6.08 |
| 375 | I1M680 | uncharacterized protein | 3 | 1.47 | 52.3 | 4.53 |
| 376 | A0A0R4J549 | uncharacterized protein | 4 | 1.46 | 55.0 | 5.38 |
| 377 | I1K795 | uncharacterized protein | 2 | 1.46 | 39.5 | 5.51 |
| 378 | I1K3U6 | uncharacterized protein | 5 | 1.45 | 46.9 | 6.73 |
| 379 | I1KQE3 | uncharacterized protein | 8 | 1.45 | 17.4 | 10.21 |
| 380 | C6TKW2 | chlorophyll a-b binding protein | 5 | 1.43 | 28.8 | 5.11 |
| 381 | I1K6T6 | uncharacterized protein | 3 | 1.43 | 35.1 | 7.02 |
| 382 | C6TGT5 | uncharacterized protein | 2 | 1.43 | 37.7 | 6.71 |
| 383 | I1JG42 | uncharacterized protein | 4 | 1.43 | 62.0 | 4.77 |
| 384 | Q2PMQ9 | photosystem ii cp47 reaction center protein | 6 | 1.42 | 56.0 | 6.20 |
| 385 | A0A0R4J5L2 | uncharacterized protein | 5 | 1.41 | 27.1 | 6.54 |
| 386 | A0A0R0HXE9 | uncharacterized protein | 2 | 1.41 | 18.7 | 10.00 |
| 387 | K7MQY6 | uncharacterized protein | 21 | 1.41 | 75.4 | 4.83 |
| 388 | I1MIW4 | uncharacterized protein | 6 | 1.41 | 39.8 | 5.55 |
| 389 | C6SVA4 | uncharacterized protein | 3 | 1.41 | 24.2 | 9.26 |
| 390 | K7K9D0 | uncharacterized protein | 18 | 1.41 | 107.4 | 5.73 |
| 391 | A0A0R0F8K4 | uncharacterized protein | 7 | 1.41 | 54.5 | 8.70 |
| 392 | C6SX81 | ferredoxin | 2 | 1.41 | 15.5 | 4.66 |
| 393 | I1JZL5 | flavin-containing monooxygenase | 5 | 1.40 | 52.2 | 5.43 |
| 394 | I1KZ34 | hexosyltransferase | 2 | 1.40 | 64.1 | 9.21 |
| 395 | P49161 | cytochrome f | 4 | 1.40 | 35.3 | 8.38 |
| 396 | I1JW44 | uncharacterized protein | 10 | 1.40 | 46.9 | 8.91 |
| 397 | Q5F2M7 | pyruvate kinase | 17 | 1.39 | 54.3 | 6.80 |
| 398 | C6SWW6 | uncharacterized protein | 2 | 1.39 | 11.6 | 9.37 |
| 399 | I1MVV5 | uncharacterized protein | 3 | 1.39 | 47.1 | 6.45 |
| 400 | B1ACD0 | kunitz trypsin inhibitor p20-1-like protein | 4 | 1.38 | 22.7 | 5.20 |
| 401 | I1KFL5 | uncharacterized protein | 5 | 1.38 | 35.2 | 6.98 |
| 402 | I1KC70 | glyceraldehyde-3-phosphate dehydrogenase | 21 | 1.38 | 36.7 | 6.72 |
| 403 | K7MEV8 | uncharacterized protein | 3 | 1.38 | 89.5 | 9.65 |
| 404 | I1LJG0 | glycosyltransferase | 3 | 1.38 | 49.4 | 8.89 |
| 405 | C6TJZ9 | uncharacterized protein | 4 | 1.38 | 38.7 | 4.93 |
| 406 | K7M9Q3 | chlorophyll a-b binding protein | 2 | 1.37 | 27.6 | 6.15 |
| 407 | K7KIL0 | gtp-binding nuclear protein | 8 | 1.37 | 25.0 | 6.42 |
| 408 | C6TAW8 | uncharacterized protein | 5 | 1.37 | 17.5 | 5.32 |
| 409 | A0A0R0HBF1 | uncharacterized protein | 5 | 1.37 | 25.3 | 5.08 |
| 410 | C6TK33 | uncharacterized protein | 10 | 1.36 | 32.5 | 6.24 |
| 411 | C6SXP0 | uncharacterized protein | 2 | 1.36 | 15.2 | 8.76 |
| 412 | A0A0R0GIU4 | peroxidase | 2 | 1.36 | 35.2 | 7.69 |
| 413 | I1L2X8 | pyruvate kinase | 12 | 1.35 | 63.3 | 7.28 |
| 414 | K7KVG4 | uncharacterized protein | 9 | 1.35 | 32.4 | 10.00 |
| 415 | C6T064 | 40s ribosomal protein s12 | 8 | 1.35 | 14.9 | 5.50 |
| 416 | I1JTM0 | uncharacterized protein | 4 | 1.35 | 40.8 | 4.83 |
| 417 | I1KC24 | uncharacterized protein | 3 | 1.34 | 25.0 | 5.19 |
| 418 | A7LCD5 | lipoxygenase | 25 | 1.34 | 96.3 | 6.54 |
| 419 | C6SYI8 | uncharacterized protein | 9 | 1.34 | 18.4 | 5.94 |
| 420 | A0A0R0IEK0 | uncharacterized protein | 8 | 1.33 | 50.6 | 6.91 |
| 421 | P02580 | actin-3 | 10 | 1.33 | 41.6 | 5.23 |
| 422 | C6T5V0 | uncharacterized protein | 2 | 1.33 | 19.5 | 9.65 |
| 423 | C6T8R7 | chlorophyll a-b binding protein | 6 | 1.32 | 28.6 | 5.46 |
| 424 | I1MRC4 | uncharacterized protein | 9 | 1.32 | 97.1 | 7.37 |
| 425 | I1J983 | uncharacterized protein | 3 | 1.32 | 28.0 | 5.21 |
| 426 | I1LMU4 | uncharacterized protein | 3 | 1.31 | 82.1 | 5.38 |
| 427 | C6TJP5 | uncharacterized protein | 6 | 1.31 | 25.7 | 5.01 |
| 428 | A0A0R0I7Q2 | uncharacterized protein | 13 | 1.30 | 127.8 | 5.47 |
| 429 | I1L4U2 | serine hydroxymethyltransferase | 17 | 1.30 | 57.0 | 7.20 |
| 430 | K7M2P2 | uncharacterized protein | 3 | 1.30 | 203.2 | 8.95 |
| 431 | K7L827 | uncharacterized protein | 10 | 1.30 | 28.9 | 9.57 |
| 432 | I1N6P9 | uncharacterized protein | 4 | 1.30 | 42.6 | 6.21 |
| 433 | I1M841 | uncharacterized protein | 10 | 1.29 | 52.4 | 5.73 |
| 434 | I1M4G0 | carboxypeptidase | 16 | 1.29 | 51.0 | 4.82 |
| 435 | K7KT64 | branched-chain-amino-acid aminotransferase | 7 | 1.29 | 50.8 | 8.51 |
| 436 | I1MYQ4 | uncharacterized protein | 4 | 1.28 | 24.9 | 7.73 |
| 437 | C6T0E8 | 60s ribosomal protein l18a | 7 | 1.28 | 21.3 | 10.44 |
| 438 | I1MVN5 | peroxidase | 15 | 1.28 | 35.5 | 9.13 |
| 439 | A0A0R0E3W8 | serine/threonine-protein phosphatase | 7 | 1.28 | 108.0 | 5.81 |
| 440 | B0M195 | peroxisomal short-chain dehydrogenase/reductase family protein | 7 | 1.28 | 26.3 | 8.80 |
| 441 | C6SVL0 | uncharacterized protein | 6 | 1.28 | 21.8 | 4.91 |
| 442 | I1M984 | uncharacterized protein | 24 | 1.28 | 60.2 | 5.81 |
| 443 | I1LYA4 | eukaryotic translation initiation factor 3 subunit k | 8 | 1.27 | 26.2 | 5.47 |
| 444 | I1M5R0 | uncharacterized protein | 4 | 1.27 | 19.9 | 6.19 |
| 445 | I1KL55 | uncharacterized protein | 7 | 1.27 | 45.6 | 6.02 |
| 446 | C6TL29 | phosphoribulokinase | 7 | 1.26 | 45.3 | 5.89 |
| 447 | K7KYH7 | uncharacterized protein | 9 | 1.26 | 80.1 | 7.36 |
| 448 | C6THL0 | uncharacterized protein | 2 | 1.26 | 36.2 | 7.29 |
| 449 | I1JC17 | uncharacterized protein | 14 | 1.25 | 58.5 | 6.48 |
| 450 | I1MTB1 | uncharacterized protein | 5 | 1.25 | 42.1 | 5.26 |
| 451 | C6SXW7 | uncharacterized protein | 3 | 1.25 | 23.1 | 4.95 |
| 452 | I1MDT4 | uncharacterized protein | 14 | 1.24 | 41.6 | 5.31 |
| 453 | I1KM30 | uncharacterized protein | 13 | 1.24 | 49.8 | 8.89 |
| 454 | I1KQ47 | uncharacterized protein | 3 | 1.24 | 134.2 | 5.76 |
| 455 | C6TCA2 | uncharacterized protein | 2 | 1.24 | 25.7 | 9.83 |
| 456 | I1M4L7 | beta-galactosidase | 6 | 1.24 | 91.6 | 6.18 |
| 457 | C6TB75 | uncharacterized protein | 3 | 1.23 | 35.3 | 6.09 |
| 458 | I1LQA8 | uncharacterized protein | 5 | 1.23 | 38.9 | 9.34 |
| 459 | C6TL64 | peroxidase | 5 | 1.23 | 36.2 | 9.06 |
| 460 | I1J4K6 | uncharacterized protein | 4 | 1.23 | 86.5 | 9.29 |
| 461 | K7L949 | uncharacterized protein | 2 | 1.22 | 14.2 | 6.11 |
| 462 | I1KJM6 | uncharacterized protein | 7 | 1.22 | 55.2 | 8.15 |
| 463 | I1KNI2 | uncharacterized protein | 4 | 1.22 | 61.4 | 6.25 |
| 464 | I1LY51 | uncharacterized protein | 15 | 1.21 | 44.1 | 8.22 |
| 465 | C6SYU5 | uncharacterized protein | 5 | 1.21 | 20.9 | 11.06 |
| 466 | I1MRM5 | uncharacterized protein | 7 | 1.21 | 17.1 | 4.98 |
| 467 | I1M3W8 | uncharacterized protein | 3 | 1.21 | 114.9 | 5.42 |
| 468 | I1LL18 | uncharacterized protein | 12 | 1.20 | 66.3 | 6.25 |
| 469 | I1JK63 | peroxidase | 2 | 1.19 | 34.6 | 8.84 |
| 470 | I1JAQ7 | uncharacterized protein | 20 | 1.19 | 45.2 | 6.36 |
| 471 | A0A0R0FJI5 | uncharacterized protein | 21 | 1.19 | 152.8 | 6.07 |
| 472 | C6TIR2 | uncharacterized protein | 6 | 1.19 | 16.9 | 4.12 |
| 473 | A0A0R0F2G6 | uncharacterized protein | 13 | 1.19 | 52.5 | 8.04 |
| 474 | I1K6P4 | uncharacterized protein | 4 | 1.18 | 27.1 | 5.44 |
| 475 | I1K4C0 | uncharacterized protein | 6 | 1.18 | 57.9 | 6.47 |
| 476 | A0A0R0HM17 | uncharacterized protein | 7 | 1.17 | 17.5 | 4.55 |
| 477 | C6TDJ3 | uncharacterized protein | 9 | 1.17 | 37.6 | 4.51 |
| 478 | I1LJ93 | dna helicase | 5 | 1.16 | 92.8 | 5.79 |
| 479 | A0A0R0J8D5 | uncharacterized protein | 5 | 1.16 | 24.9 | 9.80 |
| 480 | H2BER4 | 4-coumarate:coa ligase | 4 | 1.16 | 58.7 | 9.00 |
| 481 | C6T2R9 | uncharacterized protein | 4 | 1.16 | 19.8 | 9.13 |
| 482 | A0A0R0EZ62 | reticulon-like protein | 3 | 1.15 | 29.4 | 8.27 |
| 483 | C6SYC1 | atp synthase subunit d | 10 | 1.14 | 19.9 | 5.20 |
| 484 | I1KV09 | uncharacterized protein | 10 | 1.14 | 30.3 | 5.81 |
| 485 | I1KRP8 | uncharacterized protein | 5 | 1.14 | 52.3 | 5.75 |
| 486 | K7LFX8 | uncharacterized protein | 2 | 1.14 | 26.4 | 8.99 |
| 487 | A0A0R4J318 | peptidyl-prolyl cis-trans isomerase | 6 | 1.14 | 18.9 | 7.69 |
| 488 | A0A0R0FC11 | uncharacterized protein | 2 | 1.14 | 52.0 | 9.32 |
| 489 | K7K557 | uncharacterized protein | 5 | 1.13 | 49.5 | 5.51 |
| 490 | A0A0R4J307 | uncharacterized protein | 3 | 1.13 | 25.4 | 6.01 |
| 491 | A0A0R0LKB6 | uncharacterized protein | 8 | 1.13 | 33.9 | 4.56 |
| 492 | C6SW67 | uncharacterized protein | 2 | 1.13 | 17.4 | 5.16 |
| 493 | I1L8U0 | uncharacterized protein | 4 | 1.13 | 29.8 | 5.64 |
| 494 | C6SZA1 | uncharacterized protein | 7 | 1.12 | 16.6 | 4.20 |
| 495 | C6T588 | uncharacterized protein | 8 | 1.12 | 16.8 | 4.73 |
| 496 | I1L171 | uncharacterized protein | 13 | 1.12 | 46.0 | 5.89 |
| 497 | I1N2Z4 | uncharacterized protein | 2 | 1.12 | 41.0 | 6.90 |
| 498 | I1JTW0 | uncharacterized protein | 6 | 1.12 | 23.5 | 9.53 |
| 499 | I1KKN0 | uncharacterized protein | 6 | 1.12 | 140.7 | 4.49 |
| 500 | I1KWA0 | uncharacterized protein | 14 | 1.11 | 65.2 | 4.81 |
| 501 | C6TIW0 | fructose-bisphosphate aldolase | 10 | 1.10 | 42.5 | 8.73 |
| 502 | B3TDK9 | lipoxygenase | 43 | 1.10 | 96.3 | 6.34 |
| 503 | K7MRN9 | uncharacterized protein | 3 | 1.10 | 93.7 | 5.40 |
| 504 | C6T019 | uncharacterized protein | 7 | 1.10 | 11.8 | 5.49 |
| 505 | I1K7Q8 | uncharacterized protein | 11 | 1.10 | 34.2 | 5.73 |
| 506 | C6T5E5 | uncharacterized protein | 3 | 1.09 | 24.1 | 6.24 |
| 507 | K7LM18 | serine/threonine-protein phosphatase | 11 | 1.09 | 34.8 | 4.87 |
| 508 | I1MEZ2 | uncharacterized protein | 3 | 1.09 | 26.3 | 5.01 |
| 509 | I1JV39 | glucose-6-phosphate isomerase | 23 | 1.08 | 67.3 | 5.66 |
| 510 | C6T265 | ferredoxin | 3 | 1.07 | 16.5 | 5.59 |
| 511 | C6SWE8 | superoxide dismutase | 2 | 1.07 | 15.2 | 5.76 |
| 512 | C6T1E0 | uncharacterized protein | 5 | 1.07 | 10.1 | 6.24 |
| 513 | C6SZV4 | plastocyanin | 3 | 1.07 | 16.5 | 4.82 |
| 514 | C6T863 | uncharacterized protein | 3 | 1.07 | 37.3 | 5.04 |
| 515 | C6SXV7 | uncharacterized protein | 4 | 1.06 | 10.1 | 6.24 |
| 516 | I1N5R9 | formate dehydrogenase | 15 | 1.06 | 41.3 | 6.87 |
| 517 | A0A0R4J573 | uncharacterized protein | 3 | 1.06 | 39.9 | 9.23 |
| 518 | I1NHT9 | uncharacterized protein | 3 | 1.06 | 72.9 | 6.72 |
| 519 | I1JV54 | nad(p)h-hydrate epimerase | 6 | 1.06 | 61.4 | 8.38 |
| 520 | A0A0R0GQB4 | uncharacterized protein | 8 | 1.05 | 69.5 | 5.94 |
| 521 | C6TK57 | uncharacterized protein | 5 | 1.05 | 36.2 | 7.05 |
| 522 | I1KMC5 | uncharacterized protein | 5 | 1.05 | 31.5 | 5.64 |
| 523 | I1L4V4 | uncharacterized protein | 10 | 1.04 | 85.1 | 5.38 |
| 524 | A0A0R4J5K2 | ferredoxin--nadp reductase | 10 | 1.04 | 40.5 | 8.04 |
| 525 | K7LSM2 | uncharacterized protein | 41 | 1.04 | 153.5 | 4.70 |
| 526 | I1MYZ6 | uncharacterized protein | 5 | 1.04 | 79.1 | 5.12 |
| 527 | A0A0R0EVX5 | uncharacterized protein | 6 | 1.03 | 72.2 | 5.23 |
| 528 | I1L090 | endoglucanase | 7 | 1.03 | 68.3 | 8.81 |
| 529 | I1JEV7 | uncharacterized protein | 4 | 1.03 | 29.8 | 4.98 |
| 530 | I1KXM1 | pyruvate dehydrogenase e1 component subunit alpha | 9 | 1.03 | 43.6 | 7.12 |
| 531 | I1MAE6 | uncharacterized protein | 13 | 1.03 | 40.8 | 6.07 |
| 532 | C6TML0 | uncharacterized protein | 9 | 1.03 | 33.1 | 6.17 |
| 533 | A0A0R0EY92 | uncharacterized protein | 9 | 1.02 | 35.8 | 7.04 |
| 534 | I1LWE5 | uncharacterized protein | 3 | 1.02 | 66.1 | 7.86 |
| 535 | A0A0R4J3I5 | uncharacterized protein | 12 | 1.02 | 39.9 | 5.58 |
| 536 | I1KXH0 | uncharacterized protein | 8 | 1.01 | 36.6 | 4.83 |
| 537 | K7L4F4 | uncharacterized protein | 36 | 1.00 | 104.1 | 5.71 |
| 538 | I1LN30 | adenosylhomocysteinase | 19 | 1.00 | 53.3 | 5.84 |
| 539 | I1JUJ6 | uncharacterized protein | 5 | 1.00 | 37.5 | 8.90 |
| 540 | C6TFE2 | uncharacterized protein | 6 | 1.00 | 22.6 | 8.92 |
| 541 | I1LSW3 | uncharacterized protein | 9 | 0.99 | 50.1 | 5.42 |
| 542 | I1L4W3 | uncharacterized protein | 6 | 0.99 | 73.1 | 7.12 |
| 543 | Q5D1M7 | chloroplast glutamine synthetase | 4 | 0.99 | 30.9 | 5.56 |
| 544 | I1JUS4 | uncharacterized protein | 7 | 0.99 | 54.9 | 5.14 |
| 545 | I1KYS6 | uncharacterized protein | 2 | 0.99 | 35.2 | 6.39 |
| 546 | I1LR23 | uncharacterized protein | 16 | 0.99 | 109.5 | 7.06 |
| 547 | I1JEB4 | uncharacterized protein | 4 | 0.99 | 26.0 | 5.02 |
| 548 | A0A0R0EZU8 | uncharacterized protein | 4 | 0.99 | 25.9 | 5.64 |
| 549 | K7LZJ0 | uncharacterized protein | 6 | 0.98 | 16.6 | 10.53 |
| 550 | I1JAV9 | pectinesterase | 13 | 0.98 | 61.0 | 8.79 |
| 551 | I1MF04 | uncharacterized protein | 4 | 0.98 | 81.8 | 9.28 |
| 552 | C6TKG0 | uncharacterized protein | 5 | 0.97 | 22.7 | 5.71 |
| 553 | C6K8D0 | trypsin inhibitor 26 kda isoform | 8 | 0.97 | 25.9 | 7.83 |
| 554 | C6SVN9 | uncharacterized protein | 2 | 0.97 | 7.9 | 9.06 |
| 555 | A0A0R0FLF0 | xyloglucan endotransglucosylase/hydrolase | 13 | 0.97 | 40.4 | 9.31 |
| 556 | C6SVG2 | eukaryotic translation initiation factor 5a | 5 | 0.97 | 17.4 | 5.60 |
| 557 | A0A0R0EME4 | uncharacterized protein | 6 | 0.96 | 39.9 | 5.44 |
| 558 | C6TG68 | uncharacterized protein | 6 | 0.96 | 16.6 | 4.20 |
| 559 | C6T3S9 | uncharacterized protein | 4 | 0.96 | 24.9 | 6.43 |
| 560 | C6TJ63 | uncharacterized protein | 5 | 0.96 | 37.0 | 8.75 |
| 561 | K7LBW0 | 60s ribosomal protein l27 | 5 | 0.96 | 21.8 | 10.94 |
| 562 | C6TBK9 | uncharacterized protein | 5 | 0.96 | 33.9 | 8.51 |
| 563 | C6T5S4 | uncharacterized protein | 3 | 0.96 | 17.6 | 6.11 |
| 564 | I1MHG0 | uncharacterized protein | 4 | 0.96 | 39.0 | 6.20 |
| 565 | C6SWX2 | uncharacterized protein | 3 | 0.96 | 15.1 | 5.92 |
| 566 | C6THW5 | cytochrome b-c1 complex subunit rieske | 7 | 0.95 | 29.2 | 8.66 |
| 567 | C6TFC1 | non-specific lipid-transfer protein | 5 | 0.95 | 12.4 | 9.03 |
| 568 | K7LE13 | uncharacterized protein | 2 | 0.95 | 36.6 | 4.98 |
| 569 | I1N055 | uncharacterized protein | 4 | 0.95 | 41.1 | 8.85 |
| 570 | I1M261 | uncharacterized protein | 20 | 0.95 | 56.7 | 5.52 |
| 571 | I1KJJ6 | uncharacterized protein | 3 | 0.95 | 19.2 | 5.84 |
| 572 | A0A0R0GIJ9 | uncharacterized protein | 4 | 0.95 | 48.1 | 6.77 |
| 573 | I1L0M6 | uncharacterized protein | 4 | 0.95 | 44.6 | 5.58 |
| 574 | K7LQI0 | dirigent protein | 8 | 0.95 | 38.5 | 9.28 |
| 575 | I1M8R6 | uncharacterized protein | 2 | 0.95 | 13.1 | 4.49 |
| 576 | I1LGS5 | acyl-coenzyme a oxidase | 17 | 0.95 | 74.5 | 7.01 |
| 577 | A0A0R0GNH6 | uncharacterized protein | 6 | 0.94 | 50.5 | 6.64 |
| 578 | I1N5Z3 | alpha-mannosidase | 4 | 0.94 | 132.0 | 7.03 |
| 579 | I1KMF4 | uncharacterized protein | 3 | 0.94 | 47.8 | 7.81 |
| 580 | Q06765 | adr6 protein | 5 | 0.94 | 30.4 | 6.15 |
| 581 | K7MVB9 | uncharacterized protein | 37 | 0.93 | 116.0 | 6.39 |
| 582 | I1LQ46 | coatomer subunit gamma | 33 | 0.93 | 98.7 | 5.05 |
| 583 | I1KUN9 | transmembrane 9 superfamily member | 6 | 0.93 | 66.9 | 7.53 |
| 584 | A0A0R4J532 | uncharacterized protein | 6 | 0.92 | 21.7 | 6.43 |
| 585 | I1MXB1 | glycine cleavage system p protein | 21 | 0.92 | 114.5 | 6.92 |
| 586 | I1MIC1 | uncharacterized protein | 8 | 0.92 | 45.2 | 4.84 |
| 587 | C6SX59 | uncharacterized protein | 2 | 0.92 | 28.2 | 6.24 |
| 588 | C6TIJ7 | uncharacterized protein | 13 | 0.92 | 39.9 | 5.74 |
| 589 | I1N272 | acyl-[acyl-carrier-protein] hydrolase | 4 | 0.91 | 41.9 | 8.11 |
| 590 | I1MXL3 | uncharacterized protein | 10 | 0.91 | 52.1 | 5.55 |
| 591 | I1JTU1 | branched-chain-amino-acid aminotransferase | 8 | 0.91 | 42.0 | 6.76 |
| 592 | A0A0R0GU41 | uncharacterized protein | 4 | 0.91 | 21.7 | 9.17 |
| 593 | C6TIC4 | reticulon-like protein | 3 | 0.91 | 28.5 | 7.78 |
| 594 | I1NH00 | pyruvate kinase | 13 | 0.91 | 63.1 | 6.82 |
| 595 | I1MY33 | tubulin beta chain | 27 | 0.91 | 50.5 | 4.73 |
| 596 | I1MDJ2 | uncharacterized protein | 5 | 0.91 | 17.3 | 10.21 |
| 597 | C6T763 | uncharacterized protein | 12 | 0.91 | 30.8 | 5.04 |
| 598 | Q2PMV0 | atp synthase subunit beta | 22 | 0.91 | 53.7 | 5.29 |
| 599 | B3TDK7 | lipoxygenase | 48 | 0.90 | 96.4 | 5.76 |
| 600 | C6SVU3 | uncharacterized protein | 7 | 0.90 | 13.1 | 4.00 |
| 601 | C6TMC5 | uncharacterized protein | 3 | 0.90 | 23.7 | 9.16 |
| 602 | I1JSY1 | uncharacterized protein | 10 | 0.90 | 82.0 | 5.35 |
| 603 | I1MKS9 | mitogen-activated protein kinase | 5 | 0.90 | 42.8 | 6.15 |
| 604 | I1K754 | bifunctional dihydrofolate reductase-thymidylate synthase | 5 | 0.90 | 64.1 | 7.21 |
| 605 | C6TNC4 | aminomethyltransferase | 11 | 0.89 | 44.4 | 8.68 |
| 606 | A0A0R0FMH4 | uncharacterized protein | 57 | 0.89 | 486.8 | 4.22 |
| 607 | I1ML66 | glyceraldehyde-3-phosphate dehydrogenase | 10 | 0.89 | 43.2 | 7.61 |
| 608 | I1MZC9 | uncharacterized protein | 7 | 0.89 | 39.7 | 6.51 |
| 609 | I1MTN1 | uncharacterized protein | 6 | 0.89 | 107.3 | 5.39 |
| 610 | K7L6U4 | uncharacterized protein | 13 | 0.89 | 42.0 | 9.15 |
| 611 | I1L735 | uncharacterized protein | 6 | 0.88 | 41.9 | 6.98 |
| 612 | I1MJ34 | tau class glutathione s-transferase | 8 | 0.88 | 25.5 | 6.23 |
| 613 | C6TJQ0 | uncharacterized protein | 6 | 0.88 | 30.4 | 8.29 |
| 614 | I1LYG4 | uncharacterized protein | 3 | 0.88 | 30.3 | 6.17 |
| 615 | I1M1M2 | uncharacterized protein | 2 | 0.88 | 41.2 | 5.02 |
| 616 | C6TLS1 | uncharacterized protein | 9 | 0.88 | 39.8 | 8.37 |
| 617 | I1L7G1 | cyanate hydratase | 6 | 0.88 | 18.6 | 6.43 |
| 618 | I1K7L1 | uncharacterized protein | 3 | 0.88 | 58.0 | 8.70 |
| 619 | I1M2Y5 | uncharacterized protein | 14 | 0.88 | 38.5 | 6.71 |
| 620 | I1J4B5 | uncharacterized protein | 6 | 0.87 | 51.0 | 6.20 |
| 621 | C6THU1 | uncharacterized protein | 10 | 0.87 | 39.2 | 5.65 |
| 622 | A0A0R0KK28 | uncharacterized protein | 5 | 0.87 | 72.1 | 8.59 |
| 623 | I1KJR7 | uncharacterized protein | 3 | 0.87 | 28.4 | 4.66 |
| 624 | C6TCN5 | ferritin | 9 | 0.87 | 28.1 | 5.72 |
| 625 | K7LNW5 | uncharacterized protein | 4 | 0.87 | 24.8 | 7.03 |
| 626 | A0A0R0FKU0 | uncharacterized protein | 2 | 0.87 | 29.7 | 4.18 |
| 627 | I1NEH3 | uncharacterized protein | 12 | 0.87 | 48.4 | 7.52 |
| 628 | A0A0R0K672 | uncharacterized protein | 2 | 0.86 | 39.5 | 6.16 |
| 629 | C6T8Y4 | ferredoxin--nadp reductase | 8 | 0.86 | 42.2 | 8.38 |
| 630 | I1LDZ8 | uncharacterized protein | 4 | 0.86 | 37.7 | 5.89 |
| 631 | K7MZM9 | uncharacterized protein | 7 | 0.86 | 94.9 | 6.23 |
| 632 | I1JL51 | dirigent protein | 4 | 0.86 | 24.3 | 9.34 |
| 633 | C6SZ73 | protein rer1 | 2 | 0.85 | 22.4 | 9.50 |
| 634 | C6TBZ8 | uncharacterized protein | 2 | 0.85 | 40.4 | 8.40 |
| 635 | K7LKF5 | uncharacterized protein | 10 | 0.84 | 55.7 | 5.25 |
| 636 | I1MST3 | uncharacterized protein | 2 | 0.84 | 64.2 | 5.63 |
| 637 | I1JIR2 | uncharacterized protein | 6 | 0.84 | 101.1 | 9.04 |
| 638 | C6T7G2 | glycosyltransferase | 2 | 0.83 | 49.0 | 5.64 |
| 639 | A0A0R0JPL6 | uncharacterized protein | 9 | 0.83 | 50.0 | 8.57 |
| 640 | A0A0R0ISD1 | uncharacterized protein | 6 | 0.83 | 84.6 | 5.60 |
| 641 | I1LZQ3 | uncharacterized protein | 3 | 0.83 | 75.3 | 5.56 |
| 642 | I1NA10 | uncharacterized protein | 9 | 0.82 | 38.8 | 6.18 |
| 643 | I1M6X0 | uncharacterized protein | 6 | 0.82 | 35.0 | 5.04 |
| 644 | A0A0R0IYE6 | lipoxygenase | 29 | 0.82 | 96.3 | 6.22 |
| 645 | I1K4F3 | alpha-galactosidase | 8 | 0.82 | 71.5 | 8.14 |
| 646 | K7M8E9 | uncharacterized protein | 6 | 0.82 | 59.8 | 5.28 |
| 647 | C6SXK8 | uncharacterized protein | 2 | 0.82 | 12.9 | 9.41 |
| 648 | F6KBT3 | allene oxide cyclase 3 | 7 | 0.82 | 28.0 | 8.75 |
| 649 | C6TKL8 | uncharacterized protein | 9 | 0.82 | 34.8 | 6.49 |
| 650 | K7M247 | uncharacterized protein | 4 | 0.81 | 112.9 | 7.22 |
| 651 | C6SYC3 | uncharacterized protein | 2 | 0.81 | 18.7 | 5.00 |
| 652 | C6TBI1 | uncharacterized protein | 9 | 0.81 | 31.6 | 5.37 |
| 653 | I1N512 | uncharacterized protein | 7 | 0.81 | 37.9 | 6.22 |
| 654 | I1KM16 | uncharacterized protein | 11 | 0.81 | 28.1 | 5.46 |
| 655 | K7L0V1 | uncharacterized protein | 27 | 0.81 | 148.0 | 5.67 |
| 656 | I1LU49 | uncharacterized protein | 6 | 0.80 | 38.2 | 5.71 |
| 657 | I1MNI2 | uncharacterized protein | 3 | 0.80 | 39.6 | 6.33 |
| 658 | C6TM31 | uncharacterized protein | 2 | 0.80 | 31.7 | 8.37 |
| 659 | I1K6M2 | uncharacterized protein | 9 | 0.80 | 23.8 | 8.16 |
| 660 | I1KG22 | uncharacterized protein | 8 | 0.80 | 84.8 | 5.64 |
| 661 | C6TDJ1 | uncharacterized protein | 4 | 0.80 | 52.5 | 5.40 |
| 662 | A0A0B4PJM3 | beta-glucosidase | 9 | 0.80 | 68.7 | 9.09 |
| 663 | I1LY05 | uncharacterized protein | 11 | 0.80 | 43.2 | 4.41 |
| 664 | A0A0R0GWM0 | uncharacterized protein | 8 | 0.80 | 26.6 | 7.99 |
| 665 | A0A0R0EDY2 | uncharacterized protein | 4 | 0.80 | 77.2 | 7.22 |
| 666 | I1KAS2 | uncharacterized protein | 5 | 0.79 | 86.8 | 6.43 |
| 667 | I1KXW8 | reticulon-like protein | 4 | 0.79 | 27.0 | 8.53 |
| 668 | A0A0R0HCJ1 | uncharacterized protein | 6 | 0.79 | 37.1 | 6.56 |
| 669 | K7MNQ0 | uncharacterized protein | 5 | 0.78 | 15.3 | 6.81 |
| 670 | K7MAP3 | uncharacterized protein | 6 | 0.78 | 29.7 | 9.34 |
| 671 | I1LZ92 | uncharacterized protein | 14 | 0.78 | 37.5 | 5.29 |
| 672 | I1MC85 | uncharacterized protein | 7 | 0.78 | 45.8 | 5.55 |
| 673 | I1KE09 | uncharacterized protein | 7 | 0.78 | 17.3 | 4.79 |
| 674 | C6TA74 | uncharacterized protein | 3 | 0.78 | 39.9 | 5.11 |
| 675 | I1JU53 | uncharacterized protein | 3 | 0.78 | 10.9 | 6.16 |
| 676 | Q43453 | g.max mrna from stress-induced gene (h4) | 9 | 0.77 | 16.7 | 4.93 |
| 677 | C6SWE3 | uncharacterized protein | 5 | 0.77 | 23.9 | 5.69 |
| 678 | A0A0R0I7I9 | uncharacterized protein | 5 | 0.77 | 44.8 | 8.90 |
| 679 | I1KZ32 | uncharacterized protein | 2 | 0.76 | 49.4 | 5.00 |
| 680 | I1LIQ3 | uncharacterized protein | 3 | 0.76 | 28.1 | 4.76 |
| 681 | A0A0R0I5R9 | uncharacterized protein | 14 | 0.76 | 48.0 | 6.06 |
| 682 | C6T0H9 | uncharacterized protein | 6 | 0.76 | 15.0 | 10.40 |
| 683 | K7LTN0 | uncharacterized protein | 12 | 0.76 | 66.3 | 6.25 |
| 684 | C6KHU4 | phenylalanine ammonia-lyase | 16 | 0.76 | 78.1 | 5.83 |
| 685 | K7KZI7 | uncharacterized protein | 2 | 0.76 | 154.1 | 6.50 |
| 686 | I1LFG4 | uncharacterized protein | 14 | 0.75 | 80.2 | 6.12 |
| 687 | Q42795 | beta-amylase | 26 | 0.75 | 56.0 | 5.33 |
| 688 | P24095 | seed linoleate 9s-lipoxygenase | 48 | 0.75 | 96.8 | 5.78 |
| 689 | G0T440 | purple acid phosphatase | 15 | 0.75 | 70.3 | 5.93 |
| 690 | I1K5U0 | uncharacterized protein | 14 | 0.75 | 58.5 | 6.00 |
| 691 | C6TNH2 | uncharacterized protein | 6 | 0.74 | 40.8 | 4.79 |
| 692 | K7MNZ5 | uncharacterized protein | 3 | 0.74 | 40.4 | 6.07 |
| 693 | C6TCN3 | uncharacterized protein | 3 | 0.73 | 32.3 | 5.91 |
| 694 | I1LVZ8 | uncharacterized protein | 27 | 0.73 | 85.5 | 8.00 |
| 695 | I1JT65 | uncharacterized protein | 4 | 0.73 | 40.1 | 5.91 |
| 696 | C6TMT8 | uncharacterized protein | 4 | 0.73 | 42.9 | 5.12 |
| 697 | B1ACD3 | putative kunitz trypsin protease inhibitor | 7 | 0.73 | 23.6 | 4.68 |
| 698 | K7KY22 | uncharacterized protein | 4 | 0.73 | 20.6 | 8.59 |
| 699 | I1MMH5 | pectin acetylesterase | 14 | 0.72 | 45.8 | 8.69 |
| 700 | I1LFQ4 | uncharacterized protein | 4 | 0.72 | 13.7 | 9.61 |
| 701 | I1LLG3 | uncharacterized protein | 9 | 0.72 | 46.8 | 9.20 |
| 702 | I1KL72 | uncharacterized protein | 24 | 0.72 | 72.4 | 5.82 |
| 703 | C6SYP7 | dirigent protein | 2 | 0.72 | 24.2 | 7.09 |
| 704 | I1L007 | uncharacterized protein | 5 | 0.72 | 39.0 | 8.73 |
| 705 | K7LVH5 | uncharacterized protein | 13 | 0.71 | 82.3 | 5.98 |
| 706 | C6T9Q7 | atp-dependent clp protease proteolytic subunit | 3 | 0.71 | 29.5 | 8.57 |
| 707 | I1NBL9 | uncharacterized protein | 3 | 0.71 | 23.0 | 9.08 |
| 708 | C6SVL2 | ribulose bisphosphate carboxylase small chain | 6 | 0.71 | 20.0 | 8.87 |
| 709 | I1MNL7 | carboxypeptidase | 6 | 0.71 | 55.5 | 5.12 |
| 710 | I1J8T0 | uncharacterized protein | 12 | 0.71 | 35.6 | 5.30 |
| 711 | I1KTK0 | uncharacterized protein | 11 | 0.71 | 25.9 | 10.07 |
| 712 | C6T6T6 | uncharacterized protein | 3 | 0.70 | 7.8 | 9.40 |
| 713 | I1KG93 | glycylpeptide n-tetradecanoyltransferase | 13 | 0.70 | 49.9 | 6.11 |
| 714 | I1KUJ7 | uncharacterized protein | 3 | 0.70 | 58.4 | 6.08 |
| 715 | A0A0R0FM59 | glucose-6-phosphate 1-dehydrogenase | 18 | 0.70 | 59.2 | 5.80 |
| 716 | I1KID4 | uncharacterized protein | 4 | 0.70 | 21.6 | 8.43 |
| 717 | I1JQY5 | pectin acetylesterase | 6 | 0.70 | 45.0 | 5.76 |
| 718 | A0A0R0IXV4 | glucose-6-phosphate 1-dehydrogenase | 3 | 0.70 | 66.5 | 8.03 |
| 719 | I1K395 | uncharacterized protein | 6 | 0.70 | 32.0 | 5.28 |
| 720 | C6TI42 | uncharacterized protein | 3 | 0.69 | 6.3 | 4.28 |
| 721 | C6SY33 | uncharacterized protein | 4 | 0.69 | 25.9 | 8.68 |
| 722 | I1LZJ5 | uncharacterized protein | 6 | 0.69 | 42.0 | 7.27 |
| 723 | I1JX24 | uncharacterized protein | 8 | 0.69 | 45.8 | 6.50 |
| 724 | O81413 | ferric leghemoglobin reductase-2 | 19 | 0.69 | 53.0 | 6.91 |
| 725 | I1LIT1 | uncharacterized protein | 11 | 0.69 | 41.3 | 8.82 |
| 726 | I1N5W9 | uncharacterized protein | 2 | 0.69 | 21.1 | 9.37 |
| 727 | C6SVY4 | uncharacterized protein | 10 | 0.69 | 19.1 | 4.14 |
| 728 | I1MJC1 | uncharacterized protein | 2 | 0.68 | 137.9 | 5.56 |
| 729 | A0A0B5E2K6 | tau class glutathione s-transferase | 9 | 0.68 | 25.4 | 5.88 |
| 730 | I1JGB2 | s-formylglutathione hydrolase | 11 | 0.68 | 32.1 | 6.55 |
| 731 | K7MU75 | uncharacterized protein | 3 | 0.67 | 129.2 | 5.04 |
| 732 | C6SYG3 | uncharacterized protein | 4 | 0.67 | 23.8 | 9.48 |
| 733 | C6TJM2 | uncharacterized protein | 8 | 0.67 | 33.3 | 6.67 |
| 734 | P27066 | ribulose bisphosphate carboxylase large chain | 17 | 0.67 | 52.6 | 6.01 |
| 735 | I1LAH8 | uncharacterized protein | 3 | 0.67 | 23.9 | 5.69 |
| 736 | I1JMB4 | glyceraldehyde-3-phosphate dehydrogenase | 9 | 0.67 | 44.6 | 8.71 |
| 737 | C6T2R6 | uncharacterized protein | 2 | 0.66 | 16.8 | 9.44 |
| 738 | K7LWI4 | atp synthase subunit alpha | 8 | 0.66 | 55.7 | 5.15 |
| 739 | I1NHP0 | uncharacterized protein | 12 | 0.66 | 117.7 | 6.44 |
| 740 | I1MLW9 | uncharacterized protein | 16 | 0.66 | 34.6 | 6.20 |
| 741 | K7L441 | uncharacterized protein | 2 | 0.66 | 31.1 | 9.01 |
| 742 | I1MUX7 | uncharacterized protein | 10 | 0.66 | 66.7 | 9.22 |
| 743 | K7KKK3 | uncharacterized protein | 3 | 0.66 | 61.0 | 6.87 |
| 744 | I1M039 | uncharacterized protein | 4 | 0.66 | 32.1 | 6.13 |
| 745 | C6TNC2 | uncharacterized protein | 8 | 0.66 | 41.9 | 5.52 |
| 746 | I1LPX6 | fructose-bisphosphate aldolase | 12 | 0.66 | 42.8 | 6.86 |
| 747 | K7N544 | uncharacterized protein | 4 | 0.66 | 20.6 | 5.03 |
| 748 | I1JJN9 | uncharacterized protein | 8 | 0.66 | 51.5 | 5.50 |
| 749 | B0M1A6 | betaine aldehyde dehydrogenase | 29 | 0.66 | 54.7 | 5.23 |
| 750 | K7L7P5 | uncharacterized protein | 6 | 0.65 | 28.4 | 4.18 |
| 751 | C6T4R9 | uncharacterized protein | 7 | 0.65 | 17.7 | 9.77 |
| 752 | Q9BBU5 | cu/zn-superoxide dismutase copper chaperone | 10 | 0.65 | 32.5 | 5.41 |
| 753 | I1KQG0 | t-complex protein 1 subunit delta | 23 | 0.65 | 57.6 | 6.85 |
| 754 | I1MUQ0 | uncharacterized protein | 2 | 0.64 | 27.4 | 5.98 |
| 755 | K7LBF4 | uncharacterized protein | 10 | 0.64 | 72.2 | 6.00 |
| 756 | I1L4K9 | elongation factor ts | 10 | 0.64 | 122.7 | 4.66 |
| 757 | K7LXH7 | starch synthase | 4 | 0.64 | 86.5 | 5.59 |
| 758 | I1MBN4 | uncharacterized protein | 9 | 0.64 | 22.7 | 8.69 |
| 759 | C6TMA8 | uncharacterized protein | 6 | 0.64 | 12.2 | 4.69 |
| 760 | I1KY49 | uncharacterized protein | 8 | 0.64 | 51.2 | 6.73 |
| 761 | A0A0R0ESN9 | uncharacterized protein | 10 | 0.64 | 76.4 | 5.02 |
| 762 | I1KVZ9 | uncharacterized protein | 8 | 0.64 | 17.6 | 5.38 |
| 763 | C6TF02 | aminomethyltransferase | 11 | 0.64 | 44.2 | 8.91 |
| 764 | C6SVF1 | actin depolymerizing factor 1 | 5 | 0.64 | 16.0 | 6.15 |
| 765 | I1JNW2 | trna (guanine-n(7)-)-methyltransferase non-catalytic subunit | 2 | 0.64 | 44.7 | 5.59 |
| 766 | I1MTH5 | uncharacterized protein | 13 | 0.63 | 51.4 | 5.73 |
| 767 | I1N5C6 | uncharacterized protein | 3 | 0.63 | 40.7 | 4.43 |
| 768 | I1L5P9 | uncharacterized protein | 7 | 0.63 | 45.1 | 5.08 |
| 769 | I1KJS2 | atp-dependent 6-phosphofructokinase | 11 | 0.63 | 58.5 | 7.25 |
| 770 | C6SVF6 | uncharacterized protein | 6 | 0.63 | 11.3 | 6.05 |
| 771 | A8IKE5 | alanine aminotransferase 2 | 11 | 0.63 | 53.5 | 5.42 |
| 772 | I1MAR9 | uncharacterized protein | 24 | 0.63 | 85.0 | 7.14 |
| 773 | I1NJE8 | peptidyl-prolyl cis-trans isomerase | 5 | 0.62 | 27.1 | 9.56 |
| 774 | C6SW63 | uncharacterized protein | 5 | 0.62 | 11.4 | 4.25 |
| 775 | I1MRK4 | dihydrolipoamide acetyltransferase component of pyruvate dehydrogenase complex | 9 | 0.62 | 56.2 | 6.57 |
| 776 | I1K6B9 | 6-phosphogluconate dehydrogenase | 16 | 0.62 | 53.8 | 6.25 |
| 777 | I1K9J7 | tubulin alpha chain | 21 | 0.62 | 49.7 | 4.87 |
| 778 | C6SZH5 | uncharacterized protein | 3 | 0.62 | 15.3 | 9.98 |
| 779 | C6TNX5 | uncharacterized protein | 5 | 0.62 | 32.2 | 4.89 |
| 780 | K7MHE5 | v-type proton atpase subunit c | 9 | 0.62 | 42.5 | 5.27 |
| 781 | I1N898 | tubulin alpha chain | 22 | 0.62 | 49.9 | 4.97 |
| 782 | I1JZ45 | uncharacterized protein | 11 | 0.61 | 47.3 | 7.66 |
| 783 | I1LYU9 | arginine biosynthesis bifunctional protein argj | 4 | 0.61 | 48.5 | 5.95 |
| 784 | I1L340 | uncharacterized protein | 3 | 0.61 | 30.3 | 4.88 |
| 785 | I1M8K9 | uncharacterized protein | 7 | 0.61 | 25.8 | 6.32 |
| 786 | I1M0W3 | uncharacterized protein | 4 | 0.61 | 70.9 | 5.16 |
| 787 | I1N1Y9 | peptidylprolyl isomerase | 13 | 0.61 | 60.9 | 5.16 |
| 788 | K7M387 | uncharacterized protein | 25 | 0.61 | 127.4 | 5.18 |
| 789 | I1KH71 | acetyltransferase component of pyruvate dehydrogenase complex | 12 | 0.61 | 59.4 | 8.01 |
| 790 | A0A0R0KQE9 | uncharacterized protein | 19 | 0.61 | 50.7 | 6.80 |
| 791 | I1KFE9 | uncharacterized protein | 10 | 0.61 | 60.1 | 6.93 |
| 792 | I1MBS8 | uncharacterized protein | 6 | 0.61 | 58.3 | 5.31 |
| 793 | Q38IW8 | triosephosphate isomerase | 20 | 0.61 | 27.2 | 5.87 |
| 794 | I1JNC7 | uncharacterized protein | 17 | 0.60 | 108.5 | 5.61 |
| 795 | I1K9P5 | uncharacterized protein | 7 | 0.60 | 29.4 | 6.07 |
| 796 | I1LST9 | uncharacterized protein | 5 | 0.60 | 66.7 | 5.82 |
| 797 | K7LRK7 | uncharacterized protein | 21 | 0.60 | 154.3 | 5.17 |
| 798 | I1N1N3 | uncharacterized protein | 11 | 0.60 | 52.2 | 7.06 |
| 799 | I1NBM7 | uncharacterized protein | 11 | 0.60 | 55.5 | 6.28 |
| 800 | I1JSA4 | uncharacterized protein | 3 | 0.60 | 52.0 | 7.12 |
| 801 | C6T0A8 | uncharacterized protein | 6 | 0.60 | 27.7 | 7.05 |
| 802 | I1LRE4 | uncharacterized protein | 23 | 0.60 | 57.1 | 5.50 |
| 803 | I1KRB4 | uncharacterized protein | 3 | 0.59 | 60.4 | 8.57 |
| 804 | I1JK48 | grpe protein homolog | 5 | 0.59 | 37.2 | 4.59 |
| 805 | C6SZ14 | uncharacterized protein | 7 | 0.59 | 26.6 | 5.33 |
| 806 | I1JAL3 | uncharacterized protein | 4 | 0.59 | 61.0 | 8.91 |
| 807 | K7MMN7 | uncharacterized protein | 9 | 0.58 | 66.1 | 8.26 |
| 808 | I1MNG0 | uncharacterized protein | 5 | 0.58 | 61.3 | 6.60 |
| 809 | I1NH84 | uncharacterized protein | 5 | 0.58 | 42.1 | 4.40 |
| 810 | C6THE6 | uncharacterized protein | 5 | 0.58 | 24.2 | 9.10 |
| 811 | A0A0R0FAD6 | uncharacterized protein | 6 | 0.58 | 51.2 | 9.15 |
| 812 | I1JV02 | uncharacterized protein | 16 | 0.58 | 61.0 | 5.64 |
| 813 | I1LIJ6 | sulfite reductase | 11 | 0.57 | 77.2 | 9.22 |
| 814 | I1KJM9 | uncharacterized protein | 3 | 0.57 | 29.9 | 5.91 |
| 815 | C6T3A2 | uncharacterized protein | 5 | 0.57 | 16.7 | 4.80 |
| 816 | I1KXB5 | amine oxidase | 10 | 0.57 | 85.4 | 6.59 |
| 817 | C6T504 | uncharacterized protein | 2 | 0.57 | 10.3 | 8.06 |
| 818 | A0A0R0IAH0 | plasma membrane atpase | 21 | 0.57 | 112.4 | 6.00 |
| 819 | I1MJX0 | acetyltransferase component of pyruvate dehydrogenase complex | 14 | 0.57 | 59.6 | 7.59 |
| 820 | I1M6M4 | uncharacterized protein | 5 | 0.57 | 61.2 | 5.12 |
| 821 | K7L350 | carboxypeptidase | 12 | 0.57 | 50.7 | 8.13 |
| 822 | K7LEQ8 | uncharacterized protein | 6 | 0.57 | 79.8 | 5.27 |
| 823 | I1J8X9 | uncharacterized protein | 4 | 0.56 | 28.0 | 6.66 |
| 824 | I1MQX3 | uncharacterized protein | 13 | 0.56 | 42.3 | 8.53 |
| 825 | I1MSD9 | uncharacterized protein | 5 | 0.56 | 34.3 | 5.69 |
| 826 | C6SVD7 | phi class glutathione s-transferase | 8 | 0.56 | 24.8 | 5.73 |
| 827 | I1N7Z4 | v-type proton atpase subunit c | 10 | 0.56 | 42.8 | 5.76 |
| 828 | I1KSD7 | uncharacterized protein | 12 | 0.56 | 32.0 | 9.41 |
| 829 | K7LNQ6 | thioredoxin reductase | 11 | 0.56 | 40.0 | 8.42 |
| 830 | O04874 | glutathione transferase | 7 | 0.56 | 25.0 | 5.76 |
| 831 | I1LVR2 | uncharacterized protein | 6 | 0.56 | 26.7 | 5.79 |
| 832 | K7KNJ0 | uncharacterized protein | 2 | 0.56 | 16.5 | 10.42 |
| 833 | I1KLC9 | uncharacterized protein | 18 | 0.55 | 65.2 | 7.55 |
| 834 | A0A0R0GAK0 | uncharacterized protein | 13 | 0.55 | 30.6 | 8.60 |
| 835 | K7MZ43 | glycosyltransferase | 2 | 0.55 | 61.0 | 6.07 |
| 836 | I1LJW4 | uncharacterized protein | 2 | 0.55 | 59.3 | 9.79 |
| 837 | C6TNZ1 | uncharacterized protein | 2 | 0.55 | 24.6 | 9.16 |
| 838 | I1N786 | uncharacterized protein | 12 | 0.55 | 43.5 | 6.42 |
| 839 | C6TFM6 | peptidyl-prolyl cis-trans isomerase | 2 | 0.54 | 26.0 | 6.16 |
| 840 | I1MHG6 | uncharacterized protein | 24 | 0.54 | 54.1 | 5.54 |
| 841 | I1LJ75 | uncharacterized protein | 6 | 0.54 | 40.9 | 6.37 |
| 842 | A0A0R0GYV0 | uncharacterized protein | 2 | 0.54 | 20.4 | 10.16 |
| 843 | C6THI3 | uncharacterized protein | 11 | 0.54 | 39.1 | 6.37 |
| 844 | Q6YGT9 | purple acid phosphatase | 16 | 0.54 | 59.5 | 8.10 |
| 845 | C6T9Q3 | uncharacterized protein | 7 | 0.54 | 17.2 | 10.29 |
| 846 | A0A0R0FND9 | uncharacterized protein | 11 | 0.54 | 26.6 | 7.65 |
| 847 | I1JI26 | delta-1-pyrroline-5-carboxylate synthase | 12 | 0.54 | 77.5 | 6.37 |
| 848 | I1MZ47 | uncharacterized protein | 14 | 0.54 | 34.7 | 5.85 |
| 849 | A0A0R4J5C6 | uncharacterized protein | 5 | 0.54 | 14.9 | 5.74 |
| 850 | C6TFW0 | uncharacterized protein | 4 | 0.54 | 13.3 | 9.32 |
| 851 | C6SX35 | uncharacterized protein | 4 | 0.54 | 25.6 | 5.44 |
| 852 | I1K0L4 | uncharacterized protein | 17 | 0.54 | 71.7 | 5.66 |
| 853 | I1K361 | uncharacterized protein | 8 | 0.53 | 54.1 | 5.88 |
| 854 | I1M9A0 | uncharacterized protein | 3 | 0.53 | 21.6 | 5.69 |
| 855 | A0A0R4J4S6 | uncharacterized protein | 13 | 0.53 | 43.2 | 8.95 |
| 856 | C6TN20 | eukaryotic translation initiation factor 3 subunit h | 9 | 0.53 | 38.8 | 4.73 |
| 857 | I1JAN9 | uncharacterized protein | 3 | 0.53 | 17.3 | 9.56 |
| 858 | I1L6Y9 | alpha-galactosidase | 11 | 0.53 | 45.6 | 6.70 |
| 859 | I1KUF1 | uncharacterized protein | 8 | 0.52 | 83.8 | 5.67 |
| 860 | I1LI93 | uncharacterized protein | 10 | 0.52 | 39.3 | 6.47 |
| 861 | A0A0R0JP17 | oleosin | 3 | 0.52 | 21.4 | 10.28 |
| 862 | C6T2H1 | uncharacterized protein | 9 | 0.52 | 21.7 | 6.43 |
| 863 | I1M599 | lipoxygenase | 44 | 0.52 | 92.7 | 6.52 |
| 864 | K7KG80 | uncharacterized protein | 3 | 0.52 | 35.1 | 7.10 |
| 865 | I1JI59 | uncharacterized protein | 8 | 0.52 | 20.7 | 9.34 |
| 866 | C6TDL2 | uncharacterized protein | 4 | 0.52 | 13.7 | 9.16 |
| 867 | I1KNH9 | uncharacterized protein | 9 | 0.52 | 36.0 | 5.23 |
| 868 | C6T0B5 | uncharacterized protein | 4 | 0.52 | 14.0 | 5.73 |
| 869 | I1LGN1 | flavin-containing monooxygenase | 2 | 0.52 | 51.6 | 5.66 |
| 870 | K7LC06 | uncharacterized protein | 17 | 0.52 | 107.6 | 5.91 |
| 871 | I1M8Q5 | uncharacterized protein | 10 | 0.52 | 54.4 | 5.70 |
| 872 | A0A0R0GBB6 | uncharacterized protein | 13 | 0.52 | 45.9 | 5.90 |
| 873 | A0A0R0HR30 | uncharacterized protein | 17 | 0.52 | 47.0 | 5.49 |
| 874 | A0A0R0F3J1 | uncharacterized protein | 3 | 0.51 | 53.2 | 5.95 |
| 875 | I1K974 | uncharacterized protein | 13 | 0.51 | 50.8 | 9.31 |
| 876 | I1K5Q4 | uncharacterized protein | 2 | 0.51 | 51.2 | 6.54 |
| 877 | I1L1J3 | uncharacterized protein | 11 | 0.51 | 41.5 | 4.39 |
| 878 | I1NBI4 | uncharacterized protein | 19 | 0.51 | 58.5 | 6.12 |
| 879 | I1MHY4 | uncharacterized protein | 2 | 0.51 | 59.8 | 6.20 |
| 880 | C6TBL0 | mg-protoporphyrin ix chelatase | 8 | 0.51 | 45.8 | 5.60 |
| 881 | I1JR44 | uncharacterized protein | 5 | 0.50 | 18.6 | 6.33 |
| 882 | C6TGT0 | coatomer subunit epsilon | 11 | 0.50 | 32.3 | 5.30 |
| 883 | I1JFY3 | uncharacterized protein | 9 | 0.50 | 66.3 | 8.00 |
| 884 | I1KL66 | uncharacterized protein | 16 | 0.50 | 57.6 | 6.53 |
| 885 | I1L2D3 | uncharacterized protein | 6 | 0.50 | 23.0 | 6.16 |
| 886 | I1LYP2 | uncharacterized protein | 3 | 0.50 | 10.3 | 10.26 |
| 887 | C6SVT2 | profilin | 3 | 0.50 | 14.0 | 4.87 |
| 888 | Q5ECI5 | plastid 3-keto-acyl-acp synthase ii-b | 4 | 0.50 | 52.4 | 8.67 |
| 889 | I1KMX5 | uncharacterized protein | 21 | 0.49 | 86.0 | 9.01 |
| 890 | I1M2K9 | uncharacterized protein | 25 | 0.49 | 72.4 | 5.68 |
| 891 | I1K5T3 | uncharacterized protein | 10 | 0.49 | 44.6 | 6.28 |
| 892 | Q76LA6 | cytosolic ascorbate peroxidase 2 | 12 | 0.49 | 27.1 | 5.65 |
| 893 | A0A0R0JYY8 | tubulin beta chain | 21 | 0.49 | 49.9 | 4.89 |
| 894 | A0A0R0H3D0 | uncharacterized protein | 7 | 0.49 | 28.4 | 7.17 |
| 895 | A0A0R0FB29 | uncharacterized protein | 3 | 0.49 | 29.6 | 8.66 |
| 896 | A8C8H3 | glutamate decarboxylase | 23 | 0.49 | 57.1 | 5.52 |
| 897 | I1NJF1 | uncharacterized protein | 7 | 0.49 | 52.3 | 5.77 |
| 898 | I1LEI1 | uncharacterized protein | 8 | 0.49 | 98.1 | 6.29 |
| 899 | I1NHY2 | uncharacterized protein | 3 | 0.49 | 24.6 | 5.12 |
| 900 | C6T5K5 | prefoldin subunit 3 | 7 | 0.49 | 21.4 | 4.67 |
| 901 | I1N524 | beta-hexosaminidase | 13 | 0.49 | 63.5 | 5.55 |
| 902 | I1LJP3 | uncharacterized protein | 14 | 0.48 | 35.4 | 4.96 |
| 903 | I1M3M0 | 14-3-3-like protein d | 14 | 0.48 | 29.4 | 4.80 |
| 904 | I1NGB9 | uncharacterized protein | 5 | 0.48 | 38.6 | 5.41 |
| 905 | I1K8L5 | uncharacterized protein | 3 | 0.48 | 12.2 | 7.78 |
| 906 | I1JZR6 | uncharacterized protein | 15 | 0.48 | 61.4 | 7.23 |
| 907 | F6KBT4 | allene oxide cyclase 4 | 7 | 0.48 | 27.7 | 8.77 |
| 908 | A0A0R0I5G1 | uncharacterized protein | 27 | 0.48 | 124.7 | 6.44 |
| 909 | C6T777 | uncharacterized protein | 2 | 0.47 | 32.6 | 6.34 |
| 910 | C6TJ97 | uncharacterized protein | 2 | 0.47 | 31.9 | 9.19 |
| 911 | A0A0R0EPH6 | uncharacterized protein | 12 | 0.47 | 33.1 | 6.35 |
| 912 | I1MSD1 | uncharacterized protein | 3 | 0.47 | 35.1 | 7.09 |
| 913 | I1JH86 | fructose-bisphosphate aldolase | 22 | 0.47 | 38.2 | 7.12 |
| 914 | I1MQL3 | uncharacterized protein | 17 | 0.47 | 116.4 | 9.39 |
| 915 | K7LTG4 | uncharacterized protein | 10 | 0.47 | 44.6 | 6.67 |
| 916 | I1L8G3 | succinate--coa ligase | 12 | 0.47 | 45.3 | 5.89 |
| 917 | Q9FUK4 | glutamine synthetase | 16 | 0.47 | 39.0 | 5.48 |
| 918 | I1LV12 | uncharacterized protein | 19 | 0.47 | 68.3 | 9.24 |
| 919 | I1KQ89 | uncharacterized protein | 2 | 0.46 | 22.8 | 4.67 |
| 920 | C6TKH1 | uncharacterized protein | 4 | 0.46 | 34.3 | 6.46 |
| 921 | A0A0R0KZ26 | uncharacterized protein | 8 | 0.46 | 21.9 | 9.17 |
| 922 | A0A0R0HEY5 | uncharacterized protein | 2 | 0.46 | 43.4 | 9.04 |
| 923 | K7KQN4 | uncharacterized protein | 8 | 0.46 | 133.3 | 4.48 |
| 924 | I1KWM7 | 6-phosphogluconate dehydrogenase | 23 | 0.46 | 53.5 | 6.11 |
| 925 | I1JBQ4 | uncharacterized protein | 8 | 0.46 | 41.6 | 7.74 |
| 926 | A0A0R0G797 | uncharacterized protein | 11 | 0.46 | 43.2 | 6.48 |
| 927 | A0A0R4J4D2 | uncharacterized protein | 15 | 0.46 | 39.1 | 5.61 |
| 928 | I1NG28 | uncharacterized protein | 17 | 0.45 | 69.8 | 5.25 |
| 929 | A0A0R0EXX1 | pyruvate kinase | 16 | 0.45 | 59.3 | 6.66 |
| 930 | I1K4R1 | uncharacterized protein | 9 | 0.45 | 25.8 | 10.18 |
| 931 | C6SZN6 | uncharacterized protein | 8 | 0.45 | 17.9 | 5.60 |
| 932 | A0A0R0G189 | uncharacterized protein | 15 | 0.44 | 86.6 | 6.66 |
| 933 | I1JJD2 | pyruvate dehydrogenase e1 component subunit beta | 11 | 0.44 | 38.7 | 5.70 |
| 934 | A0A0R0H1W3 | uncharacterized protein | 11 | 0.44 | 96.9 | 5.92 |
| 935 | K7MPJ5 | phosphoinositide phospholipase c | 8 | 0.43 | 63.3 | 5.70 |
| 936 | K7MZ19 | uncharacterized protein | 14 | 0.43 | 43.9 | 6.67 |
| 937 | Q76LA8 | cytosolic ascorbate peroxidase 1 | 11 | 0.43 | 27.9 | 5.78 |
| 938 | I1JR35 | uncharacterized protein | 12 | 0.43 | 39.5 | 7.65 |
| 939 | I1LL59 | protein disulfide-isomerase | 19 | 0.43 | 64.8 | 4.58 |
| 940 | C6TJ77 | uncharacterized protein | 6 | 0.43 | 48.9 | 5.28 |
| 941 | K7LI85 | amine oxidase | 8 | 0.43 | 82.9 | 6.02 |
| 942 | C6TJJ8 | uncharacterized protein | 2 | 0.43 | 22.7 | 9.21 |
| 943 | C6TBP8 | uncharacterized protein | 11 | 0.43 | 26.6 | 7.65 |
| 944 | I1K711 | uncharacterized protein | 6 | 0.42 | 40.0 | 4.82 |
| 945 | I1L053 | uncharacterized protein | 11 | 0.42 | 39.2 | 5.49 |
| 946 | C6SY97 | mitochondrial pyruvate carrier | 4 | 0.42 | 12.2 | 9.79 |
| 947 | I1J7Y4 | uncharacterized protein | 15 | 0.42 | 201.7 | 5.49 |
| 948 | C6TDZ7 | uncharacterized protein | 3 | 0.42 | 40.2 | 5.78 |
| 949 | D4N5G0 | alpha-form rubisco activase | 9 | 0.42 | 52.2 | 5.94 |
| 950 | C6T2X0 | uncharacterized protein | 7 | 0.42 | 21.5 | 7.91 |
| 951 | I1K5E6 | uncharacterized protein | 17 | 0.42 | 35.6 | 7.62 |
| 952 | I1MSB3 | uncharacterized protein | 11 | 0.42 | 44.2 | 8.22 |
| 953 | I1LVA7 | uncharacterized protein | 25 | 0.42 | 75.6 | 6.65 |
| 954 | C6SVE0 | eukaryotic translation initiation factor 5a | 6 | 0.42 | 17.4 | 5.42 |
| 955 | I1NGB7 | uncharacterized protein | 14 | 0.42 | 57.9 | 4.61 |
| 956 | K7KJ72 | uncharacterized protein | 19 | 0.42 | 404.8 | 5.26 |
| 957 | I1K9N4 | glucose-6-phosphate isomerase | 20 | 0.41 | 67.4 | 5.52 |
| 958 | I1JMI9 | malic enzyme | 15 | 0.41 | 68.9 | 5.71 |
| 959 | I1MZR2 | uncharacterized protein | 14 | 0.40 | 127.4 | 5.13 |
| 960 | I1MGE7 | uncharacterized protein | 30 | 0.40 | 65.9 | 7.95 |
| 961 | I1LWI3 | uncharacterized protein | 7 | 0.40 | 31.5 | 5.11 |
| 962 | I1MH86 | uncharacterized protein | 10 | 0.40 | 41.4 | 4.36 |
| 963 | I1MUR2 | uncharacterized protein | 15 | 0.40 | 36.7 | 5.95 |
| 964 | Q9M5K7 | 14-3-3-like protein | 9 | 0.40 | 30.2 | 4.84 |
| 965 | O23960 | acetyl-coa carboxylase | 19 | 0.40 | 58.9 | 7.22 |
| 966 | C6SXD3 | 40s ribosomal protein s24 | 3 | 0.40 | 15.8 | 10.64 |
| 967 | C6SXS9 | uncharacterized protein | 16 | 0.40 | 23.5 | 5.81 |
| 968 | A0A0R0G3Z6 | uncharacterized protein | 12 | 0.39 | 38.3 | 5.64 |
| 969 | Q53B72 | chalcone-flavonone isomerase family protein | 11 | 0.39 | 23.5 | 4.89 |
| 970 | C6TKQ3 | uncharacterized protein | 18 | 0.39 | 34.7 | 5.93 |
| 971 | I1MUX8 | uncharacterized protein | 18 | 0.39 | 71.9 | 5.18 |
| 972 | C6TF34 | uncharacterized protein | 6 | 0.39 | 32.4 | 6.04 |
| 973 | I1KS65 | adenosylhomocysteinase | 26 | 0.39 | 53.3 | 5.60 |
| 974 | I1J9U6 | uncharacterized protein | 5 | 0.39 | 25.3 | 5.08 |
| 975 | I1KL36 | uncharacterized protein | 9 | 0.39 | 46.7 | 8.28 |
| 976 | A0A0R0HSJ5 | peptidyl-prolyl cis-trans isomerase | 4 | 0.39 | 21.9 | 8.60 |
| 977 | I1JHQ7 | uncharacterized protein | 9 | 0.39 | 59.9 | 7.60 |
| 978 | A0A0R4J5G6 | uncharacterized protein | 6 | 0.39 | 25.6 | 6.16 |
| 979 | I1LAL4 | uncharacterized protein | 34 | 0.38 | 61.1 | 5.99 |
| 980 | C6T8J1 | glucose-6-phosphate 1-epimerase | 14 | 0.38 | 36.5 | 5.85 |
| 981 | I1JIW1 | uncharacterized protein | 7 | 0.38 | 57.8 | 6.04 |
| 982 | I1KNE6 | uncharacterized protein | 8 | 0.38 | 57.1 | 5.59 |
| 983 | I1LDC1 | uncharacterized protein | 8 | 0.38 | 24.2 | 4.76 |
| 984 | C6SXY4 | uncharacterized protein | 4 | 0.38 | 18.7 | 5.05 |
| 985 | A0A0R0I9G1 | uncharacterized protein | 19 | 0.38 | 116.8 | 9.41 |
| 986 | I1MG28 | uncharacterized protein | 10 | 0.38 | 72.0 | 6.17 |
| 987 | A0A0R0G6T3 | uncharacterized protein | 20 | 0.38 | 27.2 | 5.87 |
| 988 | I1LNG1 | alpha-galactosidase | 5 | 0.38 | 73.8 | 5.59 |
| 989 | I1J6Z7 | uncharacterized protein | 19 | 0.38 | 34.9 | 6.19 |
| 990 | K7M5B1 | uncharacterized protein | 3 | 0.38 | 165.0 | 5.40 |
| 991 | A0A0R4J3E9 | uncharacterized protein | 5 | 0.38 | 17.7 | 7.82 |
| 992 | A0A0R0HDA3 | protein disulfide-isomerase | 15 | 0.37 | 67.3 | 4.82 |
| 993 | K7KDF4 | uncharacterized protein | 3 | 0.37 | 20.9 | 4.80 |
| 994 | I1KA75 | uncharacterized protein | 11 | 0.37 | 48.5 | 7.64 |
| 995 | A0A0R0JRI6 | uncharacterized protein | 2 | 0.37 | 35.9 | 5.25 |
| 996 | I1JSK2 | uncharacterized protein | 6 | 0.37 | 43.1 | 6.13 |
| 997 | I1JKS9 | uncharacterized protein | 3 | 0.37 | 31.7 | 5.77 |
| 998 | I1MNX4 | uncharacterized protein | 15 | 0.37 | 54.0 | 5.63 |
| 999 | I1KZX0 | uncharacterized protein | 11 | 0.37 | 35.8 | 5.14 |
| 1000 | K7K900 | uncharacterized protein | 6 | 0.37 | 15.6 | 5.35 |
| 1001 | C6TGA6 | 60s acidic ribosomal protein p0 | 11 | 0.37 | 34.2 | 5.00 |
| 1002 | I1KQH1 | uncharacterized protein | 15 | 0.36 | 56.3 | 5.15 |
| 1003 | I1MBZ0 | uncharacterized protein | 7 | 0.36 | 59.5 | 8.44 |
| 1004 | A0A0R0KPC8 | uncharacterized protein | 4 | 0.36 | 29.8 | 5.50 |
| 1005 | B1ACD5 | kunitz trypsin protease inhibitor | 6 | 0.36 | 22.7 | 5.07 |
| 1006 | I1MP01 | glycosyltransferase | 7 | 0.36 | 53.8 | 5.62 |
| 1007 | I1LLM7 | uncharacterized protein | 20 | 0.36 | 65.2 | 6.16 |
| 1008 | I1K7F0 | uncharacterized protein | 7 | 0.36 | 74.3 | 5.93 |
| 1009 | I1KAR6 | uncharacterized protein | 6 | 0.36 | 34.2 | 5.61 |
| 1010 | I1N2K4 | uncharacterized protein | 5 | 0.36 | 48.6 | 5.71 |
| 1011 | K7LIQ6 | uncharacterized protein | 19 | 0.36 | 107.3 | 5.62 |
| 1012 | A0A0R0E4D5 | uncharacterized protein | 3 | 0.35 | 17.9 | 5.46 |
| 1013 | I1KPJ5 | nucleoside diphosphate kinase | 5 | 0.35 | 25.5 | 9.30 |
| 1014 | I1N6A6 | udp-glucose 6-dehydrogenase | 17 | 0.35 | 53.1 | 6.29 |
| 1015 | I1K8U2 | uncharacterized protein | 9 | 0.35 | 70.3 | 5.50 |
| 1016 | A0A0R0EHR6 | 6-phosphogluconate dehydrogenase | 20 | 0.35 | 59.3 | 8.23 |
| 1017 | I1LAW6 | eukaryotic translation initiation factor 3 subunit m | 19 | 0.34 | 46.1 | 4.96 |
| 1018 | C6TH02 | uncharacterized protein | 3 | 0.34 | 25.3 | 5.98 |
| 1019 | C6T034 | uncharacterized protein | 15 | 0.34 | 24.3 | 4.53 |
| 1020 | C6TLT7 | late-embryogenesis abundant protein 1 | 16 | 0.34 | 35.6 | 4.80 |
| 1021 | C6T1N4 | uncharacterized protein | 3 | 0.34 | 17.5 | 4.85 |
| 1022 | I1M329 | uncharacterized protein | 3 | 0.34 | 63.5 | 5.18 |
| 1023 | C6TGV2 | isocitrate dehydrogenase | 11 | 0.34 | 46.7 | 8.67 |
| 1024 | C6TB96 | uncharacterized protein | 6 | 0.34 | 35.2 | 5.36 |
| 1025 | I1JWD2 | carboxypeptidase | 10 | 0.34 | 54.8 | 5.73 |
| 1026 | I1J5Q3 | uncharacterized protein | 13 | 0.34 | 68.9 | 6.63 |
| 1027 | I1ND96 | uncharacterized protein | 5 | 0.34 | 10.6 | 6.82 |
| 1028 | I1L456 | aldose 1-epimerase | 5 | 0.34 | 36.9 | 5.89 |
| 1029 | I1K4P5 | uncharacterized protein | 5 | 0.33 | 40.2 | 5.37 |
| 1030 | I1NF03 | uncharacterized protein | 20 | 0.33 | 52.0 | 4.43 |
| 1031 | C6TH97 | uncharacterized protein | 4 | 0.33 | 39.7 | 7.05 |
| 1032 | I1JHQ1 | uncharacterized protein | 15 | 0.33 | 60.6 | 6.00 |
| 1033 | G0T431 | phytase | 2 | 0.32 | 45.5 | 5.70 |
| 1034 | I1LRA7 | carboxypeptidase | 8 | 0.32 | 51.0 | 5.11 |
| 1035 | I1JTG3 | uncharacterized protein | 13 | 0.32 | 42.7 | 6.13 |
| 1036 | I1LE01 | uncharacterized protein | 7 | 0.32 | 38.7 | 6.09 |
| 1037 | A0A0R0JD84 | glycine cleavage system h protein | 2 | 0.32 | 20.0 | 4.84 |
| 1038 | C6THR8 | uncharacterized protein | 11 | 0.32 | 35.6 | 8.91 |
| 1039 | I1JZT5 | plasma membrane atpase | 25 | 0.32 | 105.0 | 6.28 |
| 1040 | C6TGV7 | uncharacterized protein | 12 | 0.32 | 46.6 | 5.58 |
| 1041 | A0A0R4J574 | serine hydroxymethyltransferase | 19 | 0.31 | 56.1 | 8.35 |
| 1042 | I1M540 | uncharacterized protein | 6 | 0.31 | 39.4 | 7.09 |
| 1043 | I1JXC1 | elongation factor tu | 14 | 0.31 | 52.2 | 6.21 |
| 1044 | C6SZ50 | uncharacterized protein | 2 | 0.31 | 23.1 | 5.80 |
| 1045 | B0M197 | peroxisomal voltage-dependent anion-selective channel protein | 9 | 0.31 | 29.8 | 8.57 |
| 1046 | I1KWT3 | uncharacterized protein | 12 | 0.30 | 37.5 | 5.96 |
| 1047 | A0A0R0HFE2 | uncharacterized protein | 7 | 0.30 | 20.8 | 9.97 |
| 1048 | I1MQD2 | uncharacterized protein | 2 | 0.30 | 12.2 | 5.70 |
| 1049 | I1LU45 | uncharacterized protein | 9 | 0.30 | 59.7 | 6.68 |
| 1050 | C6SZI9 | uncharacterized protein | 6 | 0.30 | 16.8 | 4.67 |
| 1051 | I1K8C1 | uncharacterized protein | 6 | 0.29 | 23.5 | 9.53 |
| 1052 | C6TIQ5 | uncharacterized protein | 18 | 0.29 | 37.9 | 5.93 |
| 1053 | E9KNA7 | sgf14e | 9 | 0.29 | 29.5 | 4.83 |
| 1054 | C6TK02 | uncharacterized protein | 6 | 0.29 | 47.1 | 5.66 |
| 1055 | A0A0R0IYY7 | uncharacterized protein | 22 | 0.29 | 59.0 | 8.78 |
| 1056 | C6TN55 | uncharacterized protein | 13 | 0.29 | 43.5 | 6.56 |
| 1057 | A0A0R0GZF0 | annexin | 19 | 0.29 | 35.7 | 6.78 |
| 1058 | I1KYK0 | uncharacterized protein | 8 | 0.28 | 43.7 | 5.81 |
| 1059 | I1KB25 | uncharacterized protein | 8 | 0.28 | 24.2 | 4.27 |
| 1060 | I1M7D1 | uncharacterized protein | 37 | 0.28 | 132.0 | 5.44 |
| 1061 | C6TJX5 | uncharacterized protein | 9 | 0.28 | 16.6 | 6.96 |
| 1062 | C6TNV4 | succinate--coa ligase | 8 | 0.28 | 33.8 | 8.98 |
| 1063 | I1KEW2 | uncharacterized protein | 5 | 0.28 | 40.6 | 5.66 |
| 1064 | K7M7P8 | uncharacterized protein | 15 | 0.27 | 37.8 | 4.79 |
| 1065 | I1K380 | uncharacterized protein | 3 | 0.27 | 40.2 | 6.40 |
| 1066 | K7KGI6 | uncharacterized protein | 11 | 0.27 | 55.3 | 6.28 |
| 1067 | C6TEZ8 | uncharacterized protein | 11 | 0.27 | 30.8 | 7.01 |
| 1068 | I1N8S9 | uncharacterized protein | 25 | 0.27 | 172.1 | 6.69 |
| 1069 | I1MLB1 | uncharacterized protein | 8 | 0.27 | 97.6 | 5.10 |
| 1070 | C6T750 | late-embryogenesis abundant protein 2 | 8 | 0.27 | 34.3 | 4.72 |
| 1071 | I1MK13 | uncharacterized protein | 13 | 0.26 | 61.1 | 6.91 |
| 1072 | I1K6C1 | uncharacterized protein | 11 | 0.26 | 26.7 | 6.02 |
| 1073 | Q53B75 | chalcone--flavonone isomerase 1b-1 | 10 | 0.26 | 25.0 | 5.26 |
| 1074 | I1JUD5 | uncharacterized protein | 16 | 0.26 | 48.2 | 5.46 |
| 1075 | Q01915 | atp synthase subunit alpha | 18 | 0.26 | 55.3 | 6.23 |
| 1076 | I1M4K9 | protein disulfide-isomerase | 15 | 0.25 | 62.3 | 4.70 |
| 1077 | B0M1A5 | betaine aldehyde dehydrogenase | 28 | 0.25 | 54.6 | 5.35 |
| 1078 | I1JJS4 | uncharacterized protein | 16 | 0.25 | 35.6 | 5.82 |
| 1079 | K7KU93 | uncharacterized protein | 14 | 0.25 | 87.0 | 5.72 |
| 1080 | I1N3B9 | uncharacterized protein | 7 | 0.25 | 36.9 | 8.93 |
| 1081 | C6T8K3 | uncharacterized protein | 14 | 0.25 | 27.4 | 5.13 |
| 1082 | Q39833 | alfa-carboxyltransferase | 15 | 0.25 | 76.9 | 8.63 |
| 1083 | I1KG02 | methylenetetrahydrofolate reductase | 26 | 0.24 | 66.9 | 5.60 |
| 1084 | I1JS35 | uncharacterized protein | 8 | 0.24 | 65.5 | 6.60 |
| 1085 | I1JLC7 | uncharacterized protein | 12 | 0.24 | 96.2 | 6.34 |
| 1086 | A0A0R4J2P7 | uncharacterized protein | 9 | 0.24 | 28.5 | 5.97 |
| 1087 | C6TDX4 | uncharacterized protein | 5 | 0.24 | 37.9 | 5.79 |
| 1088 | I1M6K6 | uncharacterized protein | 18 | 0.24 | 81.7 | 5.51 |
| 1089 | I1JPX8 | fructose-bisphosphate aldolase | 16 | 0.23 | 38.5 | 6.35 |
| 1090 | I1LDE9 | uncharacterized protein | 18 | 0.23 | 44.4 | 10.17 |
| 1091 | A0A0R0IZU0 | uncharacterized protein | 14 | 0.23 | 37.1 | 5.30 |
| 1092 | C6TFT8 | annexin | 11 | 0.22 | 35.6 | 6.06 |
| 1093 | I1LSZ2 | ubiquitinyl hydrolase 1 | 20 | 0.22 | 89.8 | 5.07 |
| 1094 | I1MNX6 | uncharacterized protein | 9 | 0.22 | 47.4 | 6.57 |
| 1095 | C6TJH0 | uncharacterized protein | 8 | 0.22 | 25.3 | 5.76 |
| 1096 | I1KVA8 | adp,atp carrier protein | 6 | 0.22 | 67.8 | 9.34 |
| 1097 | I1KRU6 | uncharacterized protein | 16 | 0.21 | 40.6 | 9.11 |
| 1098 | C6SVC4 | uncharacterized protein | 8 | 0.21 | 27.6 | 5.67 |
| 1099 | I1KP22 | uncharacterized protein | 2 | 0.21 | 63.8 | 5.47 |
| 1100 | I1JXP9 | uncharacterized protein | 32 | 0.20 | 102.5 | 6.16 |
| 1101 | C6SZN7 | thioredoxin | 5 | 0.20 | 13.0 | 5.29 |
| 1102 | C6SZJ2 | atp synthase subunit d | 10 | 0.20 | 19.9 | 5.11 |
| 1103 | K7N4T8 | pyruvate kinase | 14 | 0.20 | 69.6 | 6.20 |
| 1104 | I1K489 | guanosine nucleotide diphosphate dissociation inhibitor | 23 | 0.19 | 49.6 | 5.40 |
| 1105 | I1NFH5 | dihydrolipoamide acetyltransferase component of pyruvate dehydrogenase complex | 12 | 0.18 | 48.9 | 8.78 |
| 1106 | K7MY05 | translocase of chloroplast | 8 | 0.16 | 39.6 | 9.46 |
| 1107 | I1J4J6 | malic enzyme | 25 | 0.16 | 65.0 | 5.83 |
| 1108 | I1M665 | uncharacterized protein | 13 | 0.16 | 47.9 | 7.29 |
| 1109 | I1MWX6 | aspartate aminotransferase | 19 | 0.15 | 50.9 | 8.49 |
| 1110 | I1LZ03 | uncharacterized protein | 17 | 0.13 | 44.6 | 10.48 |
| 1111 | C6T898 | tubulin alpha chain | 23 | 0.12 | 49.6 | 5.03 |
| 1112 | I1K5Y5 | malic enzyme | 25 | 0.10 | 70.0 | 7.15 |
| 1113 | I1KPF0 | uncharacterized protein | 9 | -0.12 | 53.3 | 8.69 |
| 1114 | C6T008 | uncharacterized protein | 4 | -0.13 | 25.3 | 8.98 |
| 1115 | I1KGB8 | uncharacterized protein | 16 | -0.16 | 61.1 | 7.13 |
| 1116 | I1K672 | uncharacterized protein | 27 | -0.17 | 96.3 | 4.66 |
| 1117 | K7KUE2 | uncharacterized protein | 4 | -0.18 | 83.7 | 4.86 |
| 1118 | C6SWZ6 | uncharacterized protein | 7 | -0.19 | 26.2 | 10.24 |
| 1119 | A0A0R4J4L8 | uncharacterized protein | 17 | -0.19 | 47.4 | 5.42 |
| 1120 | I1KVD8 | phospholipase d | 33 | -0.19 | 91.6 | 5.42 |
| 1121 | I1M6E1 | uncharacterized protein | 40 | -0.20 | 80.1 | 4.98 |
| 1122 | C6SWH1 | uncharacterized protein | 4 | -0.20 | 17.7 | 5.44 |
| 1123 | I1L4U3 | uncharacterized protein | 29 | -0.20 | 88.4 | 5.93 |
| 1124 | I1JPW5 | uncharacterized protein | 24 | -0.21 | 47.6 | 5.49 |
| 1125 | I1JMI6 | ump-cmp kinase | 9 | -0.21 | 26.5 | 8.24 |
| 1126 | I1KZT2 | lactoylglutathione lyase | 10 | -0.21 | 32.4 | 5.74 |
| 1127 | C6T973 | uncharacterized protein | 2 | -0.21 | 13.7 | 9.66 |
| 1128 | I1JXE8 | uncharacterized protein | 18 | -0.22 | 77.9 | 5.09 |
| 1129 | I1JXA0 | uncharacterized protein | 39 | -0.22 | 90.8 | 5.11 |
| 1130 | Q42806 | pyruvate kinase | 22 | -0.22 | 55.3 | 7.50 |
| 1131 | I1KGC3 | transmembrane 9 superfamily member | 10 | -0.22 | 73.7 | 8.16 |
| 1132 | C6T977 | uncharacterized protein | 15 | -0.22 | 39.7 | 9.77 |
| 1133 | I1KGN9 | uncharacterized protein | 9 | -0.22 | 34.2 | 5.77 |
| 1134 | I1KIP9 | uncharacterized protein | 29 | -0.22 | 78.1 | 9.31 |
| 1135 | I1MG10 | protein root hair defective 3 homolog | 12 | -0.22 | 92.9 | 5.68 |
| 1136 | I1MUL3 | uncharacterized protein | 19 | -0.22 | 75.3 | 5.11 |
| 1137 | K7LBM2 | uncharacterized protein | 30 | -0.22 | 66.0 | 7.95 |
| 1138 | K7LKS0 | uncharacterized protein | 6 | -0.23 | 121.7 | 5.29 |
| 1139 | I1J752 | tubulin beta chain | 28 | -0.23 | 50.6 | 4.77 |
| 1140 | I1K2C5 | uncharacterized protein | 9 | -0.23 | 48.2 | 8.75 |
| 1141 | I1LTT3 | uncharacterized protein | 7 | -0.23 | 11.2 | 4.29 |
| 1142 | Q2PMR0 | atp-dependent clp protease proteolytic subunit | 2 | -0.24 | 22.1 | 4.59 |
| 1143 | I1MYB9 | uncharacterized protein | 20 | -0.24 | 84.3 | 5.82 |
| 1144 | I1KXQ3 | uncharacterized protein | 7 | -0.24 | 29.8 | 7.07 |
| 1145 | I1LKU1 | uncharacterized protein | 23 | -0.25 | 57.0 | 5.55 |
| 1146 | I1MVZ2 | uncharacterized protein | 19 | -0.25 | 176.5 | 8.51 |
| 1147 | I1LME6 | uncharacterized protein | 8 | -0.25 | 53.6 | 5.42 |
| 1148 | I1K5M9 | uncharacterized protein | 29 | -0.25 | 63.5 | 5.33 |
| 1149 | A0A0R4J669 | pyruvate kinase | 18 | -0.25 | 57.9 | 6.34 |
| 1150 | I1JZP0 | uncharacterized protein | 13 | -0.25 | 43.6 | 6.47 |
| 1151 | C6TKA3 | nadh-cytochrome b5 reductase | 10 | -0.25 | 35.1 | 8.63 |
| 1152 | C6TMQ6 | uncharacterized protein | 9 | -0.26 | 44.0 | 9.21 |
| 1153 | Q39828 | dynamin-related protein 5a | 18 | -0.26 | 68.3 | 8.04 |
| 1154 | I1JDR2 | uncharacterized protein | 39 | -0.26 | 116.4 | 6.34 |
| 1155 | I1KEH3 | uncharacterized protein | 8 | -0.26 | 37.6 | 6.20 |
| 1156 | K7M9J2 | histone h4 | 5 | -0.26 | 20.6 | 10.45 |
| 1157 | Q00M91 | polyadenylate-binding protein | 9 | -0.26 | 68.5 | 6.15 |
| 1158 | A0A0R0KAL0 | uncharacterized protein | 15 | -0.26 | 61.8 | 4.72 |
| 1159 | I1N5M3 | elongation factor ts | 7 | -0.26 | 38.3 | 8.32 |
| 1160 | I1LQ62 | uncharacterized protein | 19 | -0.27 | 83.7 | 5.57 |
| 1161 | I1KSR6 | uncharacterized protein | 17 | -0.27 | 39.0 | 5.12 |
| 1162 | C6SY14 | uncharacterized protein | 5 | -0.27 | 11.0 | 9.35 |
| 1163 | I1LEQ8 | fructose-bisphosphate aldolase | 11 | -0.27 | 42.5 | 8.16 |
| 1164 | C6SVK2 | uncharacterized protein | 5 | -0.27 | 13.5 | 9.47 |
| 1165 | C6SV65 | histone h2a | 3 | -0.28 | 14.7 | 10.36 |
| 1166 | I1JY44 | uncharacterized protein | 4 | -0.28 | 58.7 | 5.67 |
| 1167 | A5JVZ7 | superoxide dismutase | 8 | -0.28 | 26.7 | 8.56 |
| 1168 | C6TDL5 | uncharacterized protein | 9 | -0.28 | 28.1 | 10.72 |
| 1169 | A0A0R4J4D0 | uncharacterized protein | 10 | -0.28 | 23.0 | 5.09 |
| 1170 | I1LI35 | vacuolar protein sorting-associated protein 35 | 11 | -0.29 | 89.7 | 5.28 |
| 1171 | C6T5C9 | uncharacterized protein | 9 | -0.29 | 27.6 | 6.39 |
| 1172 | K7L432 | uncharacterized protein | 30 | -0.29 | 134.2 | 5.70 |
| 1173 | K7MV52 | uncharacterized protein | 3 | -0.29 | 70.9 | 9.08 |
| 1174 | K7M7P5 | phospho-2-dehydro-3-deoxyheptonate aldolase | 10 | -0.29 | 56.5 | 7.70 |
| 1175 | I7FST9 | protein disulfide-isomerase | 26 | -0.29 | 58.7 | 4.92 |
| 1176 | I1NC65 | proteasome subunit beta | 7 | -0.29 | 26.3 | 6.98 |
| 1177 | K7KFI9 | uncharacterized protein | 13 | -0.29 | 91.2 | 4.80 |
| 1178 | I1L5G4 | amidophosphoribosyltransferase | 12 | -0.30 | 62.0 | 6.38 |
| 1179 | I1JBN4 | uncharacterized protein | 6 | -0.30 | 26.0 | 6.20 |
| 1180 | I1KBK8 | uncharacterized protein | 16 | -0.30 | 91.0 | 5.14 |
| 1181 | I1LU78 | uncharacterized protein | 8 | -0.30 | 30.8 | 6.97 |
| 1182 | I1L5L1 | ribosomal protein | 4 | -0.30 | 37.9 | 9.25 |
| 1183 | I1M137 | uncharacterized protein | 14 | -0.30 | 64.1 | 5.76 |
| 1184 | I1NCV4 | uncharacterized protein | 14 | -0.30 | 50.0 | 6.29 |
| 1185 | O65016 | actin 4 | 19 | -0.30 | 41.4 | 5.60 |
| 1186 | I1LI99 | uncharacterized protein | 10 | -0.30 | 57.3 | 4.74 |
| 1187 | A0A0R0HF66 | uncharacterized protein | 3 | -0.30 | 21.0 | 4.66 |
| 1188 | I1LMA5 | uncharacterized protein | 23 | -0.30 | 81.3 | 6.29 |
| 1189 | I1N5N0 | pyruvate kinase | 21 | -0.30 | 55.2 | 7.50 |
| 1190 | I1MTZ8 | uncharacterized protein | 16 | -0.30 | 110.0 | 5.02 |
| 1191 | A0A0R0KHH6 | uncharacterized protein | 8 | -0.30 | 22.5 | 5.09 |
| 1192 | I1LK48 | uncharacterized protein | 4 | -0.30 | 26.2 | 10.10 |
| 1193 | I1KAV1 | uncharacterized protein | 23 | -0.31 | 96.6 | 4.62 |
| 1194 | I1K4S0 | uncharacterized protein | 12 | -0.31 | 27.5 | 6.09 |
| 1195 | B6ZK00 | peroxisomal biogenesis factor 11 family protein | 6 | -0.31 | 25.9 | 9.78 |
| 1196 | A0A0R0IQ81 | glutaredoxin | 3 | -0.31 | 17.9 | 6.82 |
| 1197 | C6SZW0 | uncharacterized protein | 9 | -0.31 | 22.1 | 4.43 |
| 1198 | K7LHD8 | uncharacterized protein | 3 | -0.31 | 21.9 | 9.51 |
| 1199 | I1JPP3 | uncharacterized protein | 35 | -0.31 | 89.6 | 5.13 |
| 1200 | A0A0R0F7T3 | uncharacterized protein | 5 | -0.31 | 44.8 | 7.19 |
| 1201 | K7N1Q0 | uncharacterized protein | 9 | -0.32 | 106.1 | 8.93 |
| 1202 | I1MZ99 | uncharacterized protein | 5 | -0.32 | 29.3 | 6.43 |
| 1203 | I1MU23 | probable trna n6-adenosine threonylcarbamoyltransferase | 3 | -0.32 | 38.5 | 5.56 |
| 1204 | C6TG44 | uncharacterized protein | 9 | -0.32 | 38.4 | 5.06 |
| 1205 | I1JUT9 | uncharacterized protein | 20 | -0.32 | 110.3 | 6.49 |
| 1206 | I1ND14 | pyrophosphate--fructose 6-phosphate 1-phosphotransferase subunit alpha | 26 | -0.32 | 67.6 | 6.52 |
| 1207 | I1LFD6 | dhar class glutathione s-transferase | 14 | -0.32 | 23.4 | 5.98 |
| 1208 | C6SW24 | uncharacterized protein | 11 | -0.32 | 22.6 | 5.86 |
| 1209 | K7MGK0 | uncharacterized protein | 16 | -0.33 | 107.7 | 5.75 |
| 1210 | I1KW16 | uncharacterized protein | 3 | -0.33 | 9.3 | 9.21 |
| 1211 | I1LVF3 | uncharacterized protein | 9 | -0.33 | 39.1 | 6.23 |
| 1212 | K7LWT5 | uncharacterized protein | 5 | -0.33 | 87.5 | 6.88 |
| 1213 | C6SWR8 | uncharacterized protein | 3 | -0.33 | 13.8 | 6.57 |
| 1214 | I1KAB7 | protein disulfide-isomerase | 25 | -0.33 | 56.1 | 4.88 |
| 1215 | A0A0R4J410 | obg-like atpase 1 | 17 | -0.33 | 44.4 | 6.36 |
| 1216 | I1LCU6 | uncharacterized protein | 18 | -0.33 | 115.8 | 6.20 |
| 1217 | I1MHA4 | dna-directed rna polymerase subunit beta | 11 | -0.33 | 135.2 | 7.35 |
| 1218 | I1KC56 | uncharacterized protein | 20 | -0.34 | 53.3 | 6.31 |
| 1219 | I1L3P6 | t-complex protein 1 subunit gamma | 20 | -0.34 | 60.2 | 5.92 |
| 1220 | I1MFH9 | lactoylglutathione lyase | 9 | -0.34 | 32.4 | 5.74 |
| 1221 | I1NIH4 | uncharacterized protein | 8 | -0.34 | 58.5 | 4.86 |
| 1222 | I1L8Z3 | uncharacterized protein | 33 | -0.34 | 90.0 | 5.16 |
| 1223 | A0A0R0FHA5 | uncharacterized protein | 11 | -0.34 | 43.5 | 4.36 |
| 1224 | I1LJ17 | uncharacterized protein | 7 | -0.34 | 61.7 | 6.28 |
| 1225 | I1MM91 | peptidylprolyl isomerase | 14 | -0.34 | 63.8 | 5.20 |
| 1226 | I1LAJ3 | uncharacterized protein | 13 | -0.34 | 36.6 | 7.61 |
| 1227 | I1KEY6 | uncharacterized protein | 46 | -0.34 | 97.3 | 5.04 |
| 1228 | I1KFU0 | uncharacterized protein | 4 | -0.34 | 36.0 | 6.54 |
| 1229 | I1KBT9 | uncharacterized protein | 7 | -0.34 | 68.7 | 5.43 |
| 1230 | K7LP96 | uncharacterized protein | 8 | -0.35 | 16.6 | 5.49 |
| 1231 | I1JGB5 | eukaryotic translation initiation factor 3 subunit e | 20 | -0.35 | 51.1 | 5.53 |
| 1232 | C6TM75 | uncharacterized protein | 11 | -0.35 | 34.1 | 5.78 |
| 1233 | K7MET7 | uncharacterized protein | 27 | -0.35 | 107.8 | 5.22 |
| 1234 | K7KMB3 | uncharacterized protein | 2 | -0.35 | 10.5 | 5.27 |
| 1235 | C6TKS1 | uncharacterized protein | 13 | -0.35 | 29.6 | 5.79 |
| 1236 | I1K2P0 | uncharacterized protein | 2 | -0.35 | 31.2 | 11.39 |
| 1237 | C6TG34 | 4-hydroxy-tetrahydrodipicolinate synthase | 6 | -0.35 | 39.9 | 6.41 |
| 1238 | C6TH59 | proteasome subunit alpha type | 13 | -0.35 | 25.6 | 5.48 |
| 1239 | I1JIA6 | uncharacterized protein | 2 | -0.35 | 7.7 | 9.74 |
| 1240 | C6T032 | uncharacterized protein | 3 | -0.35 | 24.4 | 6.53 |
| 1241 | I1JIA7 | uncharacterized protein | 7 | -0.35 | 43.2 | 6.99 |
| 1242 | C6TE22 | uncharacterized protein | 3 | -0.36 | 27.0 | 5.41 |
| 1243 | B3TDK6 | lipoxygenase | 55 | -0.36 | 96.7 | 6.12 |
| 1244 | C6TMX6 | cysteine synthase | 12 | -0.36 | 34.4 | 5.53 |
| 1245 | I1MGV6 | 60s ribosomal protein l27 | 5 | -0.36 | 15.6 | 10.38 |
| 1246 | I1LJD2 | uncharacterized protein | 9 | -0.36 | 56.0 | 6.94 |
| 1247 | C6TCU7 | homoserine dehydrogenase | 10 | -0.36 | 40.6 | 6.40 |
| 1248 | I1ND04 | uncharacterized protein | 13 | -0.36 | 161.1 | 4.45 |
| 1249 | I1LVQ6 | ribulose bisphosphate carboxylase small chain | 6 | -0.36 | 19.6 | 8.95 |
| 1250 | A0A0R0KK47 | coatomer subunit alpha | 28 | -0.36 | 137.1 | 7.21 |
| 1251 | C6SXJ9 | cytochrome b-c1 complex subunit 7 | 5 | -0.36 | 14.6 | 9.61 |
| 1252 | C6T4V0 | uncharacterized protein | 2 | -0.36 | 8.0 | 4.25 |
| 1253 | A0A0R0EC54 | uncharacterized protein | 7 | -0.36 | 17.5 | 10.46 |
| 1254 | I1LQ43 | adenylyl cyclase-associated protein | 8 | -0.36 | 50.6 | 6.57 |
| 1255 | I1NDE9 | uncharacterized protein | 9 | -0.36 | 54.4 | 4.64 |
| 1256 | I1JP48 | uncharacterized protein | 17 | -0.36 | 74.8 | 5.83 |
| 1257 | I1KY80 | uncharacterized protein | 5 | -0.37 | 19.7 | 9.06 |
| 1258 | I1M1V8 | uncharacterized protein | 17 | -0.37 | 63.4 | 5.73 |
| 1259 | I1NGS1 | uncharacterized protein | 10 | -0.37 | 24.9 | 4.51 |
| 1260 | K7LFS7 | uncharacterized protein | 8 | -0.37 | 120.6 | 5.82 |
| 1261 | I1JDD2 | uncharacterized protein | 7 | -0.37 | 48.8 | 6.74 |
| 1262 | I1KNG0 | uncharacterized protein | 9 | -0.37 | 55.6 | 6.30 |
| 1263 | C6SVL1 | ribosomal protein | 12 | -0.37 | 24.6 | 9.83 |
| 1264 | K7KNG2 | uncharacterized protein | 13 | -0.37 | 70.7 | 5.38 |
| 1265 | A0A0R4J3H1 | uncharacterized protein | 8 | -0.37 | 30.4 | 4.18 |
| 1266 | I1NDD4 | uncharacterized protein | 9 | -0.37 | 41.3 | 6.21 |
| 1267 | I1L6A1 | uncharacterized protein | 17 | -0.37 | 47.2 | 5.36 |
| 1268 | I1LA47 | eukaryotic translation initiation factor 3 subunit e | 20 | -0.38 | 51.2 | 5.41 |
| 1269 | I1JMZ4 | uncharacterized protein | 8 | -0.38 | 21.5 | 4.30 |
| 1270 | I1KUU5 | protein root hair defective 3 homolog | 15 | -0.38 | 89.5 | 5.33 |
| 1271 | I1KDR0 | uncharacterized protein | 21 | -0.38 | 57.1 | 5.23 |
| 1272 | I1JIU4 | uncharacterized protein | 14 | -0.38 | 35.8 | 4.85 |
| 1273 | C6TGK4 | uncharacterized protein | 7 | -0.38 | 39.3 | 6.63 |
| 1274 | A9CPA7 | protein disulfide isomerase family | 14 | -0.38 | 47.6 | 5.19 |
| 1275 | I1K7H0 | uncharacterized protein | 20 | -0.38 | 55.6 | 8.93 |
| 1276 | I1LE85 | uncharacterized protein | 5 | -0.38 | 37.4 | 9.40 |
| 1277 | A0A0R4J309 | uncharacterized protein | 5 | -0.38 | 18.6 | 10.50 |
| 1278 | I1L8V4 | v-type proton atpase subunit g | 3 | -0.38 | 15.2 | 8.04 |
| 1279 | K7M4J0 | uncharacterized protein | 6 | -0.38 | 23.6 | 5.07 |
| 1280 | I1LFX6 | uncharacterized protein | 7 | -0.39 | 96.9 | 4.87 |
| 1281 | C6SVL7 | uncharacterized protein | 2 | -0.39 | 17.3 | 5.37 |
| 1282 | K7M3S8 | uncharacterized protein | 3 | -0.39 | 60.4 | 5.49 |
| 1283 | K7KMT9 | elongation factor tu | 13 | -0.39 | 52.5 | 6.33 |
| 1284 | C6TMV9 | uncharacterized protein | 12 | -0.39 | 31.4 | 8.31 |
| 1285 | A0A0R0FUV4 | uncharacterized protein | 18 | -0.39 | 46.6 | 5.34 |
| 1286 | I1JUS7 | eukaryotic translation initiation factor 3 subunit l | 17 | -0.39 | 60.5 | 5.80 |
| 1287 | C6SWM6 | uncharacterized protein | 2 | -0.39 | 19.1 | 8.86 |
| 1288 | I1KS98 | uncharacterized protein | 10 | -0.39 | 46.7 | 6.02 |
| 1289 | I1K9S1 | uncharacterized protein | 9 | -0.39 | 24.2 | 9.12 |
| 1290 | I1JS33 | uncharacterized protein | 2 | -0.39 | 53.3 | 4.84 |
| 1291 | I1K006 | uncharacterized protein | 14 | -0.39 | 51.6 | 6.04 |
| 1292 | K7MQF6 | uridine kinase | 16 | -0.39 | 53.8 | 5.83 |
| 1293 | I1LV40 | uncharacterized protein | 10 | -0.40 | 39.2 | 8.68 |
| 1294 | I1LHP2 | uncharacterized protein | 10 | -0.40 | 43.2 | 5.51 |
| 1295 | C6SZZ5 | histone h2a | 3 | -0.40 | 14.0 | 10.05 |
| 1296 | I1NBT0 | importin subunit alpha | 19 | -0.40 | 58.8 | 5.15 |
| 1297 | I1LHT2 | uncharacterized protein | 3 | -0.40 | 70.8 | 8.49 |
| 1298 | I1JYL3 | uncharacterized protein | 8 | -0.40 | 33.9 | 7.09 |
| 1299 | I1JBS3 | uncharacterized protein | 10 | -0.40 | 48.6 | 5.15 |
| 1300 | I1NDM3 | uncharacterized protein | 3 | -0.40 | 35.9 | 6.71 |
| 1301 | I1NEA8 | uncharacterized protein | 9 | -0.40 | 79.3 | 5.92 |
| 1302 | I1NEY0 | uncharacterized protein | 5 | -0.40 | 53.0 | 6.52 |
| 1303 | C6T8H5 | tubulin gamma chain | 3 | -0.40 | 53.1 | 5.60 |
| 1304 | A0A0R4J455 | uricase | 13 | -0.40 | 35.1 | 8.31 |
| 1305 | I1N9Z5 | uncharacterized protein | 7 | -0.40 | 53.2 | 7.63 |
| 1306 | K7K4H6 | clathrin heavy chain | 41 | -0.41 | 195.1 | 5.26 |
| 1307 | I1LMW7 | uncharacterized protein | 13 | -0.41 | 32.6 | 5.07 |
| 1308 | Q39856 | epoxide hydrolase | 11 | -0.41 | 39.1 | 5.64 |
| 1309 | I1L645 | uncharacterized protein | 12 | -0.41 | 31.8 | 7.63 |
| 1310 | K7N039 | uncharacterized protein | 7 | -0.41 | 49.4 | 5.11 |
| 1311 | K7MYQ2 | uncharacterized protein | 17 | -0.41 | 74.7 | 5.53 |
| 1312 | I1J582 | eukaryotic translation initiation factor 3 subunit i | 13 | -0.41 | 35.8 | 6.84 |
| 1313 | I1KK35 | uncharacterized protein | 3 | -0.41 | 28.5 | 4.60 |
| 1314 | I1NCB1 | uncharacterized protein | 7 | -0.41 | 47.0 | 5.46 |
| 1315 | I1L7R5 | protein disufide isomerase-like protein | 9 | -0.41 | 40.3 | 5.46 |
| 1316 | I1J9N5 | uncharacterized protein | 6 | -0.41 | 138.5 | 6.40 |
| 1317 | C6T447 | uncharacterized protein | 7 | -0.42 | 25.1 | 5.98 |
| 1318 | I1KZI6 | uncharacterized protein | 5 | -0.42 | 98.5 | 5.54 |
| 1319 | I1KQZ7 | uncharacterized protein | 18 | -0.42 | 137.7 | 5.81 |
| 1320 | I1N520 | uncharacterized protein | 21 | -0.42 | 35.1 | 6.12 |
| 1321 | I1KQ93 | uncharacterized protein | 29 | -0.42 | 63.4 | 5.35 |
| 1322 | I1JD36 | uncharacterized protein | 3 | -0.42 | 30.0 | 4.67 |
| 1323 | I1LT58 | uncharacterized protein | 6 | -0.42 | 21.3 | 8.48 |
| 1324 | I1M8Z1 | uncharacterized protein | 6 | -0.42 | 44.1 | 6.24 |
| 1325 | A0A0R0G6V6 | exocyst complex component | 4 | -0.42 | 92.7 | 6.20 |
| 1326 | I1M322 | uncharacterized protein | 17 | -0.42 | 46.8 | 6.48 |
| 1327 | C6T1E4 | uncharacterized protein | 6 | -0.42 | 11.4 | 4.27 |
| 1328 | C6SVR8 | uncharacterized protein | 5 | -0.42 | 16.0 | 10.24 |
| 1329 | C6SVH9 | uncharacterized protein | 3 | -0.42 | 8.8 | 9.22 |
| 1330 | A0A0R0G342 | uncharacterized protein | 11 | -0.42 | 26.3 | 9.73 |
| 1331 | I1ML51 | uncharacterized protein | 6 | -0.43 | 50.9 | 6.05 |
| 1332 | I1MR46 | uncharacterized protein | 8 | -0.43 | 62.0 | 9.48 |
| 1333 | I1L6D0 | uncharacterized protein | 12 | -0.43 | 38.5 | 5.66 |
| 1334 | I1KA91 | uncharacterized protein | 6 | -0.43 | 43.4 | 4.77 |
| 1335 | I1JD99 | uncharacterized protein | 3 | -0.43 | 21.2 | 4.32 |
| 1336 | C6TGX2 | uncharacterized protein | 3 | -0.43 | 28.4 | 9.27 |
| 1337 | A0A0R0K7P8 | uncharacterized protein | 20 | -0.43 | 110.4 | 5.49 |
| 1338 | I1LUL9 | ketol-acid reductoisomerase | 21 | -0.43 | 63.3 | 6.85 |
| 1339 | I1LPY8 | coatomer subunit alpha | 31 | -0.43 | 136.7 | 6.55 |
| 1340 | C6TGK9 | uncharacterized protein | 16 | -0.43 | 47.2 | 5.30 |
| 1341 | I1LPZ4 | wbb225l1.9 protein | 2 | -0.43 | 39.8 | 5.71 |
| 1342 | A0A0R0KFI8 | uncharacterized protein | 12 | -0.43 | 38.4 | 5.78 |
| 1343 | I1L088 | uncharacterized protein | 7 | -0.43 | 91.1 | 6.63 |
| 1344 | I1KB56 | uncharacterized protein | 2 | -0.43 | 37.7 | 9.60 |
| 1345 | I1K7F4 | uncharacterized protein | 9 | -0.43 | 60.8 | 9.38 |
| 1346 | I1K0G0 | uncharacterized protein | 11 | -0.43 | 43.1 | 4.89 |
| 1347 | I1JXH8 | uncharacterized protein | 16 | -0.44 | 53.2 | 6.21 |
| 1348 | P45621 | glutamate-1-semialdehyde 2,1-aminomutase | 16 | -0.44 | 49.6 | 5.37 |
| 1349 | A0A0R0FB49 | uncharacterized protein | 4 | -0.44 | 66.5 | 5.61 |
| 1350 | I1KAF3 | uncharacterized protein | 11 | -0.44 | 65.9 | 5.82 |
| 1351 | I1LCZ2 | uncharacterized protein | 6 | -0.44 | 43.8 | 8.51 |
| 1352 | I1K146 | uncharacterized protein | 11 | -0.44 | 33.9 | 6.72 |
| 1353 | K7LQG0 | uncharacterized protein | 6 | -0.44 | 20.8 | 8.39 |
| 1354 | A0A0R0F137 | uncharacterized protein | 5 | -0.44 | 25.5 | 11.05 |
| 1355 | I1K2Y0 | tubulin beta chain | 26 | -0.44 | 49.7 | 4.82 |
| 1356 | I1MT23 | uncharacterized protein | 24 | -0.44 | 59.8 | 6.69 |
| 1357 | K7M9L4 | uncharacterized protein | 21 | -0.45 | 126.7 | 5.28 |
| 1358 | I1K5J8 | uncharacterized protein | 19 | -0.45 | 69.3 | 7.71 |
| 1359 | C6T2S4 | uncharacterized protein | 8 | -0.45 | 22.2 | 6.91 |
| 1360 | K7MZJ0 | sucrose synthase | 33 | -0.45 | 92.2 | 5.95 |
| 1361 | C6T4P6 | uncharacterized protein | 5 | -0.45 | 20.1 | 7.59 |
| 1362 | I1LCG9 | uncharacterized protein | 3 | -0.45 | 65.5 | 6.48 |
| 1363 | C6T968 | 40s ribosomal protein s8 | 8 | -0.45 | 24.7 | 10.43 |
| 1364 | I1KXE7 | uncharacterized protein | 18 | -0.45 | 64.2 | 4.86 |
| 1365 | I1KGP0 | uncharacterized protein | 11 | -0.45 | 28.4 | 9.99 |
| 1366 | I1KGY6 | uncharacterized protein | 16 | -0.45 | 80.7 | 5.40 |
| 1367 | C6TJH2 | uncharacterized protein | 12 | -0.45 | 21.7 | 9.45 |
| 1368 | I1KM07 | uncharacterized protein | 12 | -0.45 | 60.0 | 8.93 |
| 1369 | C6TFM7 | uncharacterized protein | 4 | -0.45 | 25.1 | 8.85 |
| 1370 | K7LA80 | uncharacterized protein | 11 | -0.46 | 41.3 | 8.15 |
| 1371 | A0A0R0HYT3 | uncharacterized protein | 9 | -0.46 | 24.2 | 5.49 |
| 1372 | I1K5G1 | t-complex protein 1 subunit delta | 22 | -0.46 | 57.5 | 6.85 |
| 1373 | A0A0R0IJV1 | uncharacterized protein | 7 | -0.46 | 36.9 | 8.39 |
| 1374 | I1K0K7 | uncharacterized protein | 7 | -0.46 | 63.4 | 5.79 |
| 1375 | I1K1S1 | uncharacterized protein | 9 | -0.46 | 40.0 | 5.98 |
| 1376 | I1MT80 | uncharacterized protein | 4 | -0.46 | 14.4 | 9.39 |
| 1377 | I1LGM2 | uncharacterized protein | 19 | -0.46 | 65.5 | 6.26 |
| 1378 | C6TFI8 | uncharacterized protein | 4 | -0.46 | 16.7 | 11.02 |
| 1379 | G3ECQ4 | rfls1 protein | 8 | -0.46 | 69.0 | 6.26 |
| 1380 | K7LGW1 | uncharacterized protein | 12 | -0.46 | 60.4 | 9.32 |
| 1381 | I1JE70 | uncharacterized protein | 10 | -0.46 | 160.6 | 7.40 |
| 1382 | I1MU67 | uncharacterized protein | 20 | -0.46 | 124.8 | 6.61 |
| 1383 | I1MUT2 | uncharacterized protein | 13 | -0.46 | 43.5 | 4.93 |
| 1384 | K7LNL2 | aconitate hydratase | 26 | -0.46 | 98.4 | 5.75 |
| 1385 | I1LXY1 | uncharacterized protein | 27 | -0.46 | 89.0 | 6.33 |
| 1386 | A0A0R0HUG2 | uncharacterized protein | 7 | -0.47 | 40.0 | 6.38 |
| 1387 | I1JE17 | glycosyltransferase | 16 | -0.47 | 53.1 | 5.61 |
| 1388 | I1N959 | uncharacterized protein | 6 | -0.47 | 62.1 | 5.43 |
| 1389 | A0A0R0J5W4 | cysteine synthase | 14 | -0.47 | 41.3 | 8.09 |
| 1390 | I1NJA5 | uncharacterized protein | 17 | -0.47 | 67.7 | 6.35 |
| 1391 | I1LIL5 | uncharacterized protein | 9 | -0.47 | 36.7 | 8.40 |
| 1392 | P50346 | 60s acidic ribosomal protein p0 | 11 | -0.48 | 34.1 | 5.13 |
| 1393 | I1M5P5 | uncharacterized protein | 17 | -0.48 | 160.8 | 7.58 |
| 1394 | I1MF39 | uncharacterized protein | 7 | -0.48 | 43.0 | 4.67 |
| 1395 | I1MDV8 | uncharacterized protein | 6 | -0.48 | 24.5 | 9.45 |
| 1396 | I1N8M5 | uncharacterized protein | 9 | -0.48 | 21.9 | 4.36 |
| 1397 | I1M6M0 | uncharacterized protein | 16 | -0.48 | 65.3 | 5.14 |
| 1398 | K7KYQ4 | uncharacterized protein | 6 | -0.48 | 68.0 | 7.06 |
| 1399 | C6TCK0 | uncharacterized protein | 2 | -0.48 | 30.2 | 4.36 |
| 1400 | A0A0R0HA20 | uncharacterized protein | 8 | -0.48 | 40.0 | 5.52 |
| 1401 | C6TLT3 | 40s ribosomal protein s3a | 12 | -0.48 | 29.7 | 9.81 |
| 1402 | I1LMC1 | uncharacterized protein | 5 | -0.49 | 31.7 | 11.42 |
| 1403 | I1NIY9 | mitochondrial rho gtpase | 16 | -0.49 | 71.9 | 5.36 |
| 1404 | I1JPH8 | uncharacterized protein | 8 | -0.49 | 41.3 | 7.05 |
| 1405 | I1KBN0 | uncharacterized protein | 10 | -0.49 | 50.7 | 5.57 |
| 1406 | Q8RVH5 | basic 7s globulin 2 | 10 | -0.49 | 47.2 | 8.72 |
| 1407 | I1M0N4 | uncharacterized protein | 11 | -0.49 | 124.1 | 5.70 |
| 1408 | I1NF53 | dihydroorotate dehydrogenase (quinone) | 6 | -0.49 | 48.2 | 9.14 |
| 1409 | I1LWT5 | eukaryotic translation initiation factor 3 subunit m | 12 | -0.49 | 46.3 | 4.92 |
| 1410 | C6TJ15 | uncharacterized protein | 4 | -0.49 | 32.5 | 5.83 |
| 1411 | C6TIY9 | uncharacterized protein | 3 | -0.50 | 44.9 | 6.23 |
| 1412 | K7LSP6 | uncharacterized protein | 2 | -0.50 | 37.2 | 4.89 |
| 1413 | A0A0R0H2V0 | nucleolar gtp-binding protein 1 | 5 | -0.50 | 76.8 | 9.51 |
| 1414 | C6TB70 | uncharacterized protein | 9 | -0.50 | 24.4 | 6.44 |
| 1415 | A0A0R4J4Q1 | nadph--cytochrome p450 reductase | 13 | -0.50 | 78.3 | 5.40 |
| 1416 | I1MJN7 | uncharacterized protein | 10 | -0.50 | 26.9 | 9.47 |
| 1417 | I1M5B6 | beta-galactosidase | 8 | -0.50 | 80.1 | 7.65 |
| 1418 | I1LZ47 | uncharacterized protein | 4 | -0.50 | 17.7 | 9.06 |
| 1419 | Q2Q021 | threonine synthase | 9 | -0.50 | 56.3 | 6.68 |
| 1420 | Q5QJB6 | harpin binding protein 1 | 5 | -0.50 | 28.4 | 7.88 |
| 1421 | I1MBI6 | uncharacterized protein | 16 | -0.50 | 109.8 | 5.88 |
| 1422 | C6TN36 | glyceraldehyde-3-phosphate dehydrogenase | 19 | -0.50 | 37.0 | 6.97 |
| 1423 | I1N783 | proteasome subunit alpha type | 10 | -0.51 | 27.3 | 5.59 |
| 1424 | I1J495 | uncharacterized protein | 5 | -0.51 | 87.5 | 6.43 |
| 1425 | I1M8K6 | uncharacterized protein | 10 | -0.51 | 35.2 | 6.11 |
| 1426 | I1MVG7 | uncharacterized protein | 5 | -0.51 | 76.9 | 4.26 |
| 1427 | C6TMN0 | atp-dependent clp protease proteolytic subunit | 5 | -0.51 | 26.0 | 7.76 |
| 1428 | I1NIY6 | uncharacterized protein | 18 | -0.51 | 51.4 | 6.04 |
| 1429 | Q2PMP7 | 30s ribosomal protein s3 | 5 | -0.51 | 24.7 | 9.93 |
| 1430 | I1JIR8 | uncharacterized protein | 5 | -0.51 | 54.0 | 5.22 |
| 1431 | I1JEN8 | uncharacterized protein | 2 | -0.51 | 27.5 | 5.69 |
| 1432 | C6TB35 | uncharacterized protein | 4 | -0.51 | 31.5 | 9.10 |
| 1433 | I1MI23 | uncharacterized protein | 11 | -0.51 | 81.6 | 5.74 |
| 1434 | K7MPH5 | uncharacterized protein | 11 | -0.51 | 56.1 | 8.04 |
| 1435 | I1KTL3 | uncharacterized protein | 2 | -0.51 | 75.4 | 9.29 |
| 1436 | I1M375 | uncharacterized protein | 18 | -0.51 | 69.3 | 9.02 |
| 1437 | I1JDH6 | uncharacterized protein | 11 | -0.51 | 30.2 | 10.36 |
| 1438 | K7KS32 | uncharacterized protein | 4 | -0.52 | 54.8 | 5.31 |
| 1439 | I1LRB8 | uncharacterized protein | 17 | -0.52 | 43.1 | 5.96 |
| 1440 | C6TGE9 | reticulon-like protein | 5 | -0.52 | 28.8 | 6.80 |
| 1441 | A0A0R0ELE0 | pyrroline-5-carboxylate reductase | 5 | -0.52 | 28.5 | 7.92 |
| 1442 | I1J717 | proteasome subunit alpha type | 12 | -0.52 | 25.6 | 5.48 |
| 1443 | I1KP14 | uncharacterized protein | 30 | -0.52 | 108.7 | 6.15 |
| 1444 | I1MI18 | uncharacterized protein | 5 | -0.52 | 40.1 | 8.62 |
| 1445 | I1NCF0 | uncharacterized protein | 49 | -0.52 | 287.7 | 6.38 |
| 1446 | I1N318 | proteasome subunit alpha type | 15 | -0.52 | 27.3 | 6.61 |
| 1447 | I1LQR4 | heat shock 70 kda protein | 34 | -0.52 | 71.0 | 5.10 |
| 1448 | I1JJG3 | uncharacterized protein | 4 | -0.52 | 50.3 | 6.93 |
| 1449 | I1KT65 | uncharacterized protein | 33 | -0.52 | 229.3 | 5.23 |
| 1450 | K7LR00 | uncharacterized protein | 25 | -0.52 | 121.8 | 5.20 |
| 1451 | I1KZY9 | uncharacterized protein | 14 | -0.52 | 45.8 | 5.85 |
| 1452 | I1M290 | uncharacterized protein | 5 | -0.52 | 16.7 | 10.92 |
| 1453 | K7MYU5 | eukaryotic translation initiation factor 3 subunit a | 26 | -0.52 | 111.7 | 9.36 |
| 1454 | C6SVG5 | peptidyl-prolyl cis-trans isomerase | 7 | -0.52 | 21.0 | 8.41 |
| 1455 | I1KZ13 | uncharacterized protein | 17 | -0.53 | 58.9 | 6.49 |
| 1456 | A0A0R0L5Y8 | uncharacterized protein | 3 | -0.53 | 28.9 | 8.94 |
| 1457 | Q9S7N8 | seed maturation protein pm21 | 2 | -0.53 | 10.1 | 4.91 |
| 1458 | I1JMZ6 | uncharacterized protein | 7 | -0.53 | 188.4 | 4.95 |
| 1459 | I1LDR2 | tubulin beta chain | 28 | -0.53 | 50.4 | 4.72 |
| 1460 | I1MR83 | uncharacterized protein | 4 | -0.53 | 25.5 | 5.44 |
| 1461 | I1J7Y9 | peptidyl-prolyl cis-trans isomerase | 2 | -0.53 | 18.1 | 7.76 |
| 1462 | D2D5G3 | methyltransferase | 11 | -0.53 | 40.4 | 6.47 |
| 1463 | K7MPN5 | folylpolyglutamate synthase | 5 | -0.53 | 60.1 | 6.10 |
| 1464 | K7KL44 | glyceraldehyde-3-phosphate dehydrogenase | 21 | -0.53 | 46.7 | 7.27 |
| 1465 | I1MBM5 | uncharacterized protein | 2 | -0.53 | 8.3 | 9.65 |
| 1466 | I1NAL2 | uncharacterized protein | 6 | -0.53 | 13.1 | 4.76 |
| 1467 | C6TC46 | uncharacterized protein | 8 | -0.53 | 49.2 | 4.87 |
| 1468 | I1JMN8 | uncharacterized protein | 9 | -0.53 | 46.2 | 6.00 |
| 1469 | A0A0R0FER5 | elongation factor 1-alpha | 16 | -0.53 | 49.4 | 9.14 |
| 1470 | I1MUE2 | uncharacterized protein | 6 | -0.54 | 71.0 | 8.09 |
| 1471 | A0A0R4J2L6 | uncharacterized protein | 33 | -0.54 | 121.3 | 5.78 |
| 1472 | K7MKF1 | uncharacterized protein | 2 | -0.54 | 29.5 | 9.30 |
| 1473 | I1KTW3 | uncharacterized protein | 15 | -0.54 | 57.6 | 7.50 |
| 1474 | I1MT35 | uncharacterized protein | 9 | -0.54 | 53.1 | 6.76 |
| 1475 | I1MJC7 | phosphoglycerate kinase | 22 | -0.54 | 42.4 | 5.96 |
| 1476 | C6T9F2 | uncharacterized protein | 3 | -0.54 | 33.8 | 4.86 |
| 1477 | A0A0R0GHB3 | uncharacterized protein | 22 | -0.54 | 90.4 | 5.13 |
| 1478 | I1J881 | uncharacterized protein | 2 | -0.54 | 13.9 | 10.18 |
| 1479 | I1N6T7 | uncharacterized protein | 5 | -0.54 | 46.0 | 5.96 |
| 1480 | A0A0R0J4R6 | uncharacterized protein | 3 | -0.54 | 42.0 | 4.93 |
| 1481 | I1N1Y6 | 40s ribosomal protein s8 | 9 | -0.55 | 24.7 | 10.40 |
| 1482 | C6SWD5 | uncharacterized protein | 2 | -0.55 | 19.7 | 8.61 |
| 1483 | V6CKR0 | expansin | 5 | -0.55 | 27.5 | 9.16 |
| 1484 | I1JSX8 | uncharacterized protein | 11 | -0.55 | 60.6 | 9.42 |
| 1485 | I1MUN3 | uncharacterized protein | 19 | -0.55 | 61.1 | 8.55 |
| 1486 | I1KVH4 | uncharacterized protein | 20 | -0.55 | 124.6 | 6.65 |
| 1487 | I1JF82 | methylcrotonoyl-coa carboxylase subunit alpha | 14 | -0.55 | 81.1 | 6.88 |
| 1488 | C6SXT8 | uncharacterized protein | 3 | -0.55 | 23.0 | 9.32 |
| 1489 | K7L8K5 | uncharacterized protein | 9 | -0.55 | 48.8 | 8.40 |
| 1490 | A0A0R0FH00 | uncharacterized protein | 30 | -0.55 | 71.2 | 5.09 |
| 1491 | I1LPN7 | uncharacterized protein | 6 | -0.55 | 18.8 | 6.28 |
| 1492 | K7MRH9 | uncharacterized protein | 11 | -0.55 | 68.7 | 8.02 |
| 1493 | I1JDT3 | uncharacterized protein | 3 | -0.56 | 12.8 | 11.06 |
| 1494 | Q2I0H4 | glyceraldehyde-3-phosphate dehydrogenase | 21 | -0.56 | 36.7 | 6.72 |
| 1495 | K7MMK0 | uncharacterized protein | 4 | -0.56 | 42.2 | 9.05 |
| 1496 | I1NBE6 | uncharacterized protein | 17 | -0.56 | 82.1 | 7.00 |
| 1497 | K7M084 | uncharacterized protein | 3 | -0.56 | 16.6 | 7.71 |
| 1498 | Q42797 | trans-cinnamate 4-monooxygenase | 16 | -0.56 | 58.0 | 9.05 |
| 1499 | I1MVW0 | uncharacterized protein | 4 | -0.56 | 49.4 | 6.74 |
| 1500 | I1LB97 | uncharacterized protein | 6 | -0.56 | 14.1 | 9.57 |
| 1501 | C6SZC5 | uncharacterized protein | 5 | -0.56 | 12.3 | 9.34 |
| 1502 | C6T891 | uncharacterized protein | 3 | -0.56 | 32.7 | 9.47 |
| 1503 | K7LP97 | uncharacterized protein | 6 | -0.56 | 16.9 | 7.82 |
| 1504 | I1JKM1 | uncharacterized protein | 9 | -0.56 | 32.3 | 4.98 |
| 1505 | I1KEL5 | uncharacterized protein | 12 | -0.57 | 97.7 | 5.85 |
| 1506 | I1KRD5 | uncharacterized protein | 7 | -0.57 | 14.9 | 5.60 |
| 1507 | I1M6Y5 | uncharacterized protein | 6 | -0.57 | 91.4 | 6.83 |
| 1508 | I1MBZ3 | uncharacterized protein | 12 | -0.57 | 98.4 | 5.30 |
| 1509 | I1JW13 | uncharacterized protein | 7 | -0.57 | 30.4 | 7.95 |
| 1510 | I1LK42 | uncharacterized protein | 13 | -0.57 | 38.7 | 7.72 |
| 1511 | I1KY36 | uncharacterized protein | 12 | -0.57 | 54.5 | 5.75 |
| 1512 | I1MT10 | uncharacterized protein | 27 | -0.57 | 70.8 | 5.27 |
| 1513 | C6TFG0 | uncharacterized protein | 9 | -0.57 | 23.0 | 10.37 |
| 1514 | I1LAQ3 | uncharacterized protein | 7 | -0.57 | 57.0 | 6.03 |
| 1515 | I1LJG9 | uncharacterized protein | 17 | -0.57 | 83.8 | 5.47 |
| 1516 | I1LD52 | uncharacterized protein | 15 | -0.57 | 63.2 | 6.11 |
| 1517 | I1JV14 | uncharacterized protein | 16 | -0.58 | 74.4 | 6.37 |
| 1518 | Q7G1G6 | aspartate aminotransferase | 22 | -0.58 | 45.6 | 7.72 |
| 1519 | I1J7H3 | ferritin | 7 | -0.58 | 28.7 | 5.77 |
| 1520 | C6T5F7 | uncharacterized protein | 2 | -0.58 | 21.2 | 9.45 |
| 1521 | C6TNA3 | uncharacterized protein | 11 | -0.58 | 26.7 | 7.79 |
| 1522 | I1JD96 | uncharacterized protein | 7 | -0.58 | 39.8 | 6.01 |
| 1523 | I1KXX2 | membrin | 3 | -0.58 | 25.9 | 9.54 |
| 1524 | I1LPA6 | uncharacterized protein | 4 | -0.58 | 38.8 | 8.54 |
| 1525 | I1KUM4 | uncharacterized protein | 12 | -0.58 | 115.8 | 4.92 |
| 1526 | I1L3T1 | udp-glycosyltransferase 708d1 | 8 | -0.58 | 52.5 | 5.42 |
| 1527 | K7L2B3 | uncharacterized protein | 9 | -0.58 | 96.9 | 5.56 |
| 1528 | K7MYR4 | uncharacterized protein | 5 | -0.58 | 49.6 | 4.99 |
| 1529 | I1M5G8 | malic enzyme | 7 | -0.58 | 71.3 | 7.24 |
| 1530 | I1KSN3 | uncharacterized protein | 6 | -0.58 | 77.0 | 6.35 |
| 1531 | I1KTK7 | clustered mitochondria protein homolog | 18 | -0.59 | 157.2 | 5.93 |
| 1532 | I1JTW7 | glycine cleavage system p protein | 19 | -0.59 | 111.9 | 6.97 |
| 1533 | A0A0R0LJG6 | uncharacterized protein | 4 | -0.59 | 112.9 | 7.12 |
| 1534 | I1J4Z1 | uncharacterized protein | 4 | -0.59 | 66.6 | 8.14 |
| 1535 | I1MJG6 | uncharacterized protein | 5 | -0.59 | 180.4 | 7.38 |
| 1536 | I1LFH9 | uncharacterized protein | 7 | -0.59 | 219.3 | 8.37 |
| 1537 | I1LY75 | uncharacterized protein | 8 | -0.59 | 33.9 | 5.99 |
| 1538 | C6TAT2 | tau class glutathione s-transferase | 6 | -0.59 | 25.6 | 5.57 |
| 1539 | I1LD03 | dolichyl-diphosphooligosaccharide--protein glycosyltransferase subunit 1 | 11 | -0.59 | 52.7 | 6.79 |
| 1540 | I1NCV6 | uncharacterized protein | 11 | -0.60 | 174.4 | 5.66 |
| 1541 | I1KQK1 | uncharacterized protein | 5 | -0.60 | 27.2 | 9.13 |
| 1542 | I1N3E7 | uncharacterized protein | 5 | -0.60 | 46.5 | 8.64 |
| 1543 | C6T321 | uncharacterized protein | 3 | -0.60 | 19.7 | 4.74 |
| 1544 | K7LK48 | uncharacterized protein | 10 | -0.60 | 70.5 | 6.18 |
| 1545 | C6TI98 | uncharacterized protein | 6 | -0.60 | 35.9 | 8.37 |
| 1546 | C6TJ17 | uncharacterized protein | 11 | -0.60 | 33.6 | 6.46 |
| 1547 | K7KA83 | uncharacterized protein | 21 | -0.60 | 120.5 | 5.09 |
| 1548 | C6TAV3 | uncharacterized protein | 6 | -0.60 | 30.4 | 6.40 |
| 1549 | C6T3K0 | uncharacterized protein | 2 | -0.60 | 22.5 | 4.83 |
| 1550 | I1L602 | uncharacterized protein | 9 | -0.60 | 29.7 | 8.57 |
| 1551 | I1MM85 | uncharacterized protein | 14 | -0.61 | 58.5 | 6.26 |
| 1552 | C6TGB8 | uncharacterized protein | 11 | -0.61 | 46.2 | 5.73 |
| 1553 | I1ND97 | uncharacterized protein | 2 | -0.61 | 22.1 | 5.02 |
| 1554 | I1J4X9 | uncharacterized protein | 29 | -0.61 | 88.4 | 5.85 |
| 1555 | I1M6N8 | uncharacterized protein | 3 | -0.61 | 43.8 | 7.70 |
| 1556 | C6T3P3 | uncharacterized protein | 4 | -0.61 | 24.3 | 8.35 |
| 1557 | K7MJ68 | uncharacterized protein | 2 | -0.61 | 33.5 | 7.09 |
| 1558 | I1JFL5 | uncharacterized protein | 3 | -0.61 | 14.7 | 5.46 |
| 1559 | I1JCR1 | uncharacterized protein | 2 | -0.61 | 51.8 | 3.81 |
| 1560 | Q39873 | lea protein | 17 | -0.61 | 49.4 | 6.72 |
| 1561 | I1K7J4 | tubulin beta chain | 27 | -0.61 | 50.4 | 4.76 |
| 1562 | C6TB56 | uncharacterized protein | 7 | -0.61 | 38.6 | 6.75 |
| 1563 | I1JUQ4 | uncharacterized protein | 12 | -0.61 | 49.5 | 9.18 |
| 1564 | I1KB01 | uncharacterized protein | 3 | -0.61 | 41.6 | 6.47 |
| 1565 | I1JTB8 | glucose-6-phosphate isomerase | 19 | -0.62 | 62.6 | 5.90 |
| 1566 | I1L7W8 | phi class glutathione s-transferase | 3 | -0.62 | 24.7 | 5.26 |
| 1567 | K7LNY5 | uncharacterized protein | 2 | -0.62 | 123.7 | 5.90 |
| 1568 | C6TMK3 | cysteine proteinase inhibitor | 8 | -0.62 | 27.6 | 6.40 |
| 1569 | C6TDX5 | uncharacterized protein | 8 | -0.62 | 42.9 | 5.96 |
| 1570 | K7MNV4 | uncharacterized protein | 4 | -0.62 | 22.1 | 5.13 |
| 1571 | A0A0R4J2R4 | uncharacterized protein | 8 | -0.62 | 63.8 | 9.52 |
| 1572 | K7LKF8 | histone h2b | 2 | -0.62 | 22.6 | 10.00 |
| 1573 | I1LTQ1 | uncharacterized protein | 16 | -0.62 | 43.4 | 6.03 |
| 1574 | I1LG41 | protein phosphatase methylesterase 1 | 5 | -0.62 | 40.5 | 6.43 |
| 1575 | I1KSA8 | serine/threonine-protein phosphatase | 8 | -0.62 | 35.6 | 5.21 |
| 1576 | I1LN41 | uncharacterized protein | 19 | -0.63 | 84.5 | 5.90 |
| 1577 | I1M0X0 | uncharacterized protein | 11 | -0.63 | 64.5 | 6.69 |
| 1578 | Q8H928 | phosphoenolpyruvate carboxylase | 43 | -0.63 | 110.6 | 6.05 |
| 1579 | I1MXM6 | polyadenylate-binding protein | 13 | -0.63 | 71.9 | 6.70 |
| 1580 | I1LTF0 | uncharacterized protein | 2 | -0.63 | 51.8 | 8.89 |
| 1581 | I1KEN4 | uncharacterized protein | 34 | -0.63 | 110.2 | 5.52 |
| 1582 | I1LUJ6 | uncharacterized protein | 8 | -0.63 | 37.1 | 9.64 |
| 1583 | I1JVJ8 | uncharacterized protein | 2 | -0.63 | 54.4 | 8.89 |
| 1584 | I1JNI4 | uncharacterized protein | 7 | -0.63 | 66.9 | 5.74 |
| 1585 | A0A0R0GZT5 | uncharacterized protein | 13 | -0.63 | 27.3 | 5.72 |
| 1586 | I1ND31 | uncharacterized protein | 12 | -0.63 | 42.8 | 6.90 |
| 1587 | I1LK85 | uncharacterized protein | 10 | -0.63 | 84.4 | 5.87 |
| 1588 | C6TC72 | eukaryotic translation initiation factor 3 subunit f | 10 | -0.64 | 31.7 | 5.13 |
| 1589 | K7KGN5 | uncharacterized protein | 10 | -0.64 | 70.5 | 5.25 |
| 1590 | I1KYT6 | ruvb-like helicase | 10 | -0.64 | 51.0 | 5.34 |
| 1591 | I1KMJ8 | uncharacterized protein | 7 | -0.64 | 122.1 | 4.72 |
| 1592 | K7LIK2 | carboxypeptidase | 9 | -0.64 | 55.8 | 5.55 |
| 1593 | I1LH18 | uncharacterized protein | 12 | -0.64 | 48.0 | 5.28 |
| 1594 | C6T336 | uncharacterized protein | 7 | -0.64 | 15.7 | 9.98 |
| 1595 | I1KLQ4 | uncharacterized protein | 7 | -0.64 | 120.1 | 7.24 |
| 1596 | I1MIA8 | uncharacterized protein | 8 | -0.64 | 15.2 | 4.43 |
| 1597 | C6SYI9 | uncharacterized protein | 3 | -0.64 | 22.6 | 5.05 |
| 1598 | I1JUY7 | atp-dependent 6-phosphofructokinase | 12 | -0.64 | 56.0 | 6.33 |
| 1599 | I1L5R3 | ump-cmp kinase | 2 | -0.64 | 26.3 | 6.19 |
| 1600 | I1L7C9 | uncharacterized protein | 2 | -0.65 | 53.6 | 8.42 |
| 1601 | I1M0K3 | cysteine proteinase inhibitor | 9 | -0.65 | 27.7 | 6.10 |
| 1602 | A0A0R0JPA1 | beta-galactosidase | 16 | -0.65 | 94.0 | 7.21 |
| 1603 | C6TLM5 | uncharacterized protein | 8 | -0.65 | 27.7 | 8.67 |
| 1604 | I1KUP2 | uncharacterized protein | 2 | -0.65 | 51.8 | 9.05 |
| 1605 | I1KZR0 | uncharacterized protein | 22 | -0.65 | 100.1 | 9.12 |
| 1606 | K7M988 | uncharacterized protein | 6 | -0.65 | 73.4 | 8.95 |
| 1607 | Q96446 | actin | 4 | -0.65 | 22.4 | 5.45 |
| 1608 | I1KL32 | uncharacterized protein | 11 | -0.65 | 42.8 | 8.17 |
| 1609 | K7LRH4 | uncharacterized protein | 10 | -0.65 | 32.2 | 9.23 |
| 1610 | C6SWA6 | histone h2a | 3 | -0.66 | 15.7 | 10.66 |
| 1611 | I1KIV5 | uncharacterized protein | 20 | -0.66 | 65.3 | 4.92 |
| 1612 | I1KYF3 | uncharacterized protein | 5 | -0.66 | 83.4 | 5.96 |
| 1613 | K7K525 | uncharacterized protein | 12 | -0.66 | 42.1 | 6.27 |
| 1614 | I1L1D9 | uncharacterized protein | 4 | -0.66 | 10.5 | 9.37 |
| 1615 | I1NC67 | cysteine synthase | 12 | -0.66 | 34.3 | 5.50 |
| 1616 | C6SY19 | uncharacterized protein | 5 | -0.66 | 20.1 | 9.69 |
| 1617 | I1KPN2 | uncharacterized protein | 27 | -0.66 | 96.3 | 4.67 |
| 1618 | C6TKK5 | uncharacterized protein | 8 | -0.66 | 22.1 | 6.10 |
| 1619 | I1JPW6 | uncharacterized protein | 2 | -0.66 | 36.2 | 9.52 |
| 1620 | C6SVH4 | uncharacterized protein | 4 | -0.66 | 16.0 | 6.30 |
| 1621 | I1J9W0 | 40s ribosomal protein s30 | 2 | -0.66 | 11.6 | 12.07 |
| 1622 | K7KQP0 | uncharacterized protein | 7 | -0.66 | 52.2 | 9.07 |
| 1623 | C6TBY4 | uncharacterized protein | 6 | -0.67 | 14.1 | 9.47 |
| 1624 | C6TGR9 | uncharacterized protein | 5 | -0.67 | 29.2 | 6.34 |
| 1625 | I1MJD7 | inosine-5'-monophosphate dehydrogenase | 11 | -0.67 | 53.3 | 5.77 |
| 1626 | I1LSZ7 | proteasome subunit alpha type | 13 | -0.67 | 27.4 | 5.58 |
| 1627 | I1L7H1 | uncharacterized protein | 13 | -0.67 | 70.5 | 6.05 |
| 1628 | I1JCI7 | peptidyl-prolyl cis-trans isomerase | 3 | -0.67 | 13.6 | 9.56 |
| 1629 | C6T2H2 | 40s ribosomal protein s7 | 8 | -0.67 | 22.3 | 9.76 |
| 1630 | A0A0R0J965 | uncharacterized protein | 13 | -0.67 | 34.4 | 6.45 |
| 1631 | K7KRC0 | uncharacterized protein | 10 | -0.68 | 48.2 | 5.41 |
| 1632 | I1K3T9 | uncharacterized protein | 8 | -0.68 | 84.6 | 5.92 |
| 1633 | K7MUT7 | uncharacterized protein | 7 | -0.68 | 57.8 | 6.77 |
| 1634 | A0A0R0KK01 | histone h2a | 4 | -0.68 | 14.5 | 10.39 |
| 1635 | I1KF75 | uncharacterized protein | 2 | -0.68 | 33.1 | 8.59 |
| 1636 | I1L3K8 | uncharacterized protein | 6 | -0.68 | 40.9 | 4.87 |
| 1637 | A0A0R0K553 | uncharacterized protein | 7 | -0.68 | 34.8 | 5.42 |
| 1638 | M1FPG4 | nadh dehydrogenase subunit 2 | 3 | -0.68 | 53.3 | 8.89 |
| 1639 | I1NH22 | uncharacterized protein | 2 | -0.68 | 71.2 | 5.70 |
| 1640 | K7KP88 | uncharacterized protein | 3 | -0.69 | 255.0 | 5.72 |
| 1641 | K7LRU6 | uncharacterized protein | 8 | -0.69 | 77.8 | 4.76 |
| 1642 | A0A0R0GUR9 | uncharacterized protein | 9 | -0.69 | 55.8 | 6.31 |
| 1643 | C6TJ33 | uncharacterized protein | 4 | -0.69 | 33.3 | 4.90 |
| 1644 | I1LA46 | uncharacterized protein | 5 | -0.69 | 30.6 | 6.20 |
| 1645 | I1JKA3 | uncharacterized protein | 3 | -0.69 | 114.0 | 9.10 |
| 1646 | I1MM44 | glyceraldehyde-3-phosphate dehydrogenase | 9 | -0.69 | 44.8 | 8.83 |
| 1647 | I1M042 | uncharacterized protein | 10 | -0.69 | 61.4 | 8.75 |
| 1648 | C6TFM1 | uncharacterized protein | 8 | -0.69 | 40.9 | 7.13 |
| 1649 | I1K7A7 | glyceraldehyde-3-phosphate dehydrogenase | 11 | -0.69 | 48.4 | 6.76 |
| 1650 | C6SWA9 | uncharacterized protein | 7 | -0.69 | 11.4 | 4.32 |
| 1651 | C6T0P8 | uncharacterized protein | 3 | -0.69 | 15.1 | 6.59 |
| 1652 | I1K7A1 | dolichyl-diphosphooligosaccharide--protein glycosyltransferase subunit 1 | 14 | -0.70 | 68.4 | 6.62 |
| 1653 | I1N8M1 | uncharacterized protein | 3 | -0.70 | 49.2 | 5.88 |
| 1654 | I1JWV8 | uncharacterized protein | 4 | -0.70 | 35.7 | 5.26 |
| 1655 | I1JYT2 | uncharacterized protein | 5 | -0.70 | 59.5 | 5.06 |
| 1656 | K7MU41 | uncharacterized protein | 7 | -0.70 | 66.9 | 8.80 |
| 1657 | I1KV08 | uncharacterized protein | 15 | -0.70 | 54.4 | 5.84 |
| 1658 | K7LRU2 | uncharacterized protein | 10 | -0.70 | 33.2 | 10.05 |
| 1659 | I1JT75 | thioredoxin | 6 | -0.70 | 13.1 | 5.24 |
| 1660 | C6T129 | uncharacterized protein | 6 | -0.71 | 17.2 | 6.14 |
| 1661 | I1L314 | heat shock protein 90-1 | 27 | -0.71 | 80.3 | 4.95 |
| 1662 | I1MCE2 | signal recognition particle 54 kda protein | 8 | -0.71 | 55.2 | 9.23 |
| 1663 | C6THI7 | uncharacterized protein | 3 | -0.71 | 31.3 | 8.96 |
| 1664 | A0A0R0JH94 | glucose-1-phosphate adenylyltransferase | 8 | -0.71 | 58.7 | 8.50 |
| 1665 | I1M7K1 | uncharacterized protein | 6 | -0.71 | 30.1 | 6.99 |
| 1666 | C6SX10 | mitochondrial fission 1 protein | 4 | -0.71 | 18.7 | 6.96 |
| 1667 | Q9LLQ6 | seed maturation protein pm34 | 27 | -0.71 | 31.7 | 6.60 |
| 1668 | I1K7H3 | uncharacterized protein | 3 | -0.71 | 27.4 | 8.78 |
| 1669 | C6SV69 | uncharacterized protein | 3 | -0.71 | 17.8 | 6.62 |
| 1670 | K7LCK9 | uncharacterized protein | 3 | -0.71 | 108.4 | 5.91 |
| 1671 | I1LN28 | uncharacterized protein | 4 | -0.71 | 51.3 | 9.62 |
| 1672 | I1K4M6 | uncharacterized protein | 30 | -0.72 | 229.2 | 5.24 |
| 1673 | A0A0R4J5Q8 | uncharacterized protein | 21 | -0.72 | 79.9 | 6.35 |
| 1674 | I1K5H2 | uncharacterized protein | 24 | -0.72 | 109.4 | 5.14 |
| 1675 | A0A0R0I4F6 | uncharacterized protein | 25 | -0.72 | 31.8 | 6.95 |
| 1676 | I1MJM1 | clustered mitochondria protein homolog | 14 | -0.72 | 155.9 | 5.94 |
| 1677 | I1JQZ7 | uncharacterized protein | 19 | -0.72 | 145.1 | 6.76 |
| 1678 | I1NHI4 | uncharacterized protein | 10 | -0.72 | 118.1 | 5.60 |
| 1679 | C6T8W5 | elongation factor tu | 14 | -0.72 | 49.9 | 6.62 |
| 1680 | I1LGI8 | uncharacterized protein | 3 | -0.72 | 79.2 | 4.43 |
| 1681 | K7KPK3 | uncharacterized protein | 9 | -0.72 | 73.0 | 4.21 |
| 1682 | C6SWG3 | uncharacterized protein | 4 | -0.72 | 17.4 | 10.41 |
| 1683 | K7KZK7 | uncharacterized protein | 3 | -0.72 | 122.0 | 5.32 |
| 1684 | I1KMS6 | uncharacterized protein | 4 | -0.73 | 19.0 | 6.96 |
| 1685 | C6SW77 | uncharacterized protein | 6 | -0.73 | 25.8 | 9.72 |
| 1686 | A0A0R0EK65 | eukaryotic translation initiation factor 3 subunit m | 19 | -0.73 | 46.1 | 4.91 |
| 1687 | C6SWL0 | glutathione peroxidase | 5 | -0.73 | 26.4 | 9.44 |
| 1688 | A0A0R0K474 | uncharacterized protein | 18 | -0.73 | 46.7 | 5.29 |
| 1689 | I1JSJ3 | fructose-bisphosphate aldolase | 7 | -0.73 | 42.5 | 6.38 |
| 1690 | C6TH44 | uncharacterized protein | 6 | -0.73 | 31.8 | 9.72 |
| 1691 | I1JYB3 | uncharacterized protein | 4 | -0.73 | 105.3 | 6.02 |
| 1692 | A0A0R0EM71 | uncharacterized protein | 13 | -0.73 | 62.8 | 6.01 |
| 1693 | I1LVI3 | uncharacterized protein | 19 | -0.73 | 100.1 | 9.13 |
| 1694 | C6TNL4 | uncharacterized protein | 5 | -0.73 | 37.3 | 8.24 |
| 1695 | I1LRC2 | histone deacetylase hdt1 | 5 | -0.73 | 31.7 | 4.75 |
| 1696 | K7LS61 | uncharacterized protein | 3 | -0.73 | 32.0 | 9.21 |
| 1697 | I1MB71 | fructose-bisphosphate aldolase | 21 | -0.74 | 38.3 | 7.12 |
| 1698 | I1JED0 | udp-glucose 6-dehydrogenase | 17 | -0.74 | 52.8 | 6.18 |
| 1699 | A0A0R0FUC5 | uncharacterized protein | 3 | -0.74 | 21.4 | 5.16 |
| 1700 | I1J6C3 | uncharacterized protein | 9 | -0.74 | 43.2 | 9.03 |
| 1701 | I1KDL4 | phosphoenolpyruvate carboxylase | 46 | -0.74 | 110.6 | 5.72 |
| 1702 | C6TGF3 | uncharacterized protein | 19 | -0.74 | 54.4 | 6.14 |
| 1703 | A0A0R0L273 | uncharacterized protein | 8 | -0.74 | 23.9 | 7.64 |
| 1704 | I1KBE3 | uncharacterized protein | 3 | -0.74 | 27.0 | 10.12 |
| 1705 | K7KWT9 | uncharacterized protein | 8 | -0.74 | 44.9 | 7.82 |
| 1706 | C6TNR9 | uncharacterized protein | 6 | -0.74 | 32.2 | 8.39 |
| 1707 | I1K8C9 | glycine cleavage system p protein | 18 | -0.75 | 111.0 | 6.95 |
| 1708 | I1MN74 | peptidyl-prolyl cis-trans isomerase | 3 | -0.75 | 13.6 | 9.56 |
| 1709 | C6TH20 | uncharacterized protein | 8 | -0.75 | 39.4 | 5.96 |
| 1710 | I1MWP7 | cytochrome b-c1 complex subunit 7 | 5 | -0.75 | 14.5 | 9.58 |
| 1711 | A0A0R0GA14 | uncharacterized protein | 3 | -0.75 | 17.9 | 6.86 |
| 1712 | C6TM13 | uncharacterized protein | 3 | -0.75 | 30.0 | 5.64 |
| 1713 | I1K5E5 | uncharacterized protein | 8 | -0.75 | 66.4 | 6.34 |
| 1714 | A0A0R0GIX5 | nicotinamide-nucleotide adenylyltransferase | 3 | -0.75 | 31.7 | 6.25 |
| 1715 | A0A0R4J305 | uncharacterized protein | 18 | -0.75 | 44.6 | 8.25 |
| 1716 | I1N505 | uncharacterized protein | 3 | -0.75 | 58.3 | 8.48 |
| 1717 | K7MIH4 | uncharacterized protein | 7 | -0.75 | 24.2 | 4.54 |
| 1718 | K7MB15 | uncharacterized protein | 8 | -0.75 | 89.9 | 5.24 |
| 1719 | I1KNV8 | uncharacterized protein | 7 | -0.75 | 95.1 | 5.03 |
| 1720 | C6SVW6 | uncharacterized protein | 3 | -0.76 | 26.6 | 5.84 |
| 1721 | I1MPI1 | uncharacterized protein | 27 | -0.76 | 80.2 | 4.97 |
| 1722 | Q8LJW0 | 40s ribosomal protein s4 | 14 | -0.76 | 29.9 | 10.27 |
| 1723 | I1KEV1 | uncharacterized protein | 2 | -0.76 | 72.5 | 6.36 |
| 1724 | C6TJN4 | uncharacterized protein | 4 | -0.76 | 31.4 | 9.22 |
| 1725 | I1NF56 | uncharacterized protein | 2 | -0.76 | 16.9 | 6.29 |
| 1726 | I1JR56 | uncharacterized protein | 6 | -0.76 | 19.5 | 6.14 |
| 1727 | I1LKT2 | uncharacterized protein | 5 | -0.76 | 48.5 | 8.46 |
| 1728 | A0A0R0EWR2 | uncharacterized protein | 7 | -0.76 | 52.9 | 6.33 |
| 1729 | K7MT90 | uncharacterized protein | 16 | -0.76 | 35.1 | 5.76 |
| 1730 | I1MKG5 | uncharacterized protein | 2 | -0.76 | 31.2 | 10.01 |
| 1731 | I1KJK0 | uncharacterized protein | 9 | -0.76 | 39.3 | 5.95 |
| 1732 | I1MTX9 | dna helicase | 9 | -0.76 | 80.8 | 7.20 |
| 1733 | A0A0R0IW79 | uncharacterized protein | 22 | -0.76 | 146.2 | 5.57 |
| 1734 | C6TI71 | uncharacterized protein | 3 | -0.77 | 16.6 | 4.96 |
| 1735 | I1N5P1 | uncharacterized protein | 5 | -0.77 | 19.9 | 5.45 |
| 1736 | C6T1W7 | nadh dehydrogenase | 4 | -0.77 | 18.3 | 8.87 |
| 1737 | K7MSX8 | uncharacterized protein | 4 | -0.77 | 34.1 | 9.98 |
| 1738 | C6TGA0 | uncharacterized protein | 2 | -0.77 | 35.4 | 5.97 |
| 1739 | K7LW22 | uncharacterized protein | 5 | -0.77 | 116.7 | 7.32 |
| 1740 | C6SWU2 | uncharacterized protein | 3 | -0.77 | 17.7 | 4.81 |
| 1741 | I1M2V7 | uncharacterized protein | 25 | -0.77 | 75.5 | 6.65 |
| 1742 | C6SWV3 | uncharacterized protein | 13 | -0.77 | 27.6 | 5.72 |
| 1743 | I1JUQ6 | trna (guanine-n(7)-)-methyltransferase | 2 | -0.77 | 28.9 | 6.40 |
| 1744 | I1MU56 | uncharacterized protein | 18 | -0.78 | 82.2 | 5.82 |
| 1745 | I1K1B1 | uncharacterized protein | 8 | -0.78 | 283.3 | 5.73 |
| 1746 | M1FIU8 | atpase subunit 8 | 4 | -0.78 | 18.3 | 9.77 |
| 1747 | K7LDR4 | uncharacterized protein | 6 | -0.78 | 101.8 | 4.83 |
| 1748 | I1K992 | uncharacterized protein | 3 | -0.78 | 37.9 | 8.71 |
| 1749 | C6T3U6 | ribosomal protein l19 | 2 | -0.78 | 24.8 | 11.45 |
| 1750 | I1M0Z8 | uncharacterized protein | 3 | -0.79 | 129.9 | 5.29 |
| 1751 | C6TAN6 | uncharacterized protein | 4 | -0.79 | 41.6 | 7.07 |
| 1752 | A0A0R4J3Q7 | 18.5 kda class i heat shock protein | 5 | -0.79 | 18.5 | 5.99 |
| 1753 | A0A0R0K7S9 | uncharacterized protein | 3 | -0.79 | 16.5 | 4.26 |
| 1754 | I1KCP1 | plasma membrane atpase | 19 | -0.79 | 105.2 | 6.39 |
| 1755 | I1NAY9 | serine/threonine-protein phosphatase | 9 | -0.80 | 35.6 | 5.12 |
| 1756 | I1N877 | h/aca ribonucleoprotein complex subunit | 3 | -0.80 | 20.7 | 11.49 |
| 1757 | I1LKY8 | uncharacterized protein | 5 | -0.80 | 61.5 | 6.65 |
| 1758 | I1JP06 | uncharacterized protein | 10 | -0.80 | 56.4 | 5.06 |
| 1759 | I1LEY1 | uncharacterized protein | 18 | -0.81 | 65.4 | 4.92 |
| 1760 | A0A0R0HXT4 | uncharacterized protein | 5 | -0.81 | 29.0 | 6.90 |
| 1761 | I1JXD5 | uncharacterized protein | 5 | -0.81 | 83.1 | 5.17 |
| 1762 | A0A0R4J4T4 | uncharacterized protein | 6 | -0.81 | 44.3 | 8.37 |
| 1763 | I1LV52 | uncharacterized protein | 4 | -0.81 | 39.5 | 9.57 |
| 1764 | I1M2M2 | uncharacterized protein | 15 | -0.81 | 90.9 | 6.54 |
| 1765 | I1LIT2 | uncharacterized protein | 11 | -0.81 | 78.3 | 5.44 |
| 1766 | C6T8H4 | uncharacterized protein | 3 | -0.82 | 35.8 | 7.33 |
| 1767 | I1K195 | uncharacterized protein | 3 | -0.82 | 23.0 | 8.85 |
| 1768 | C6SZU6 | uncharacterized protein | 2 | -0.82 | 11.2 | 4.78 |
| 1769 | A0A0R4J571 | uncharacterized protein | 2 | -0.82 | 31.1 | 5.71 |
| 1770 | I1M8J4 | uncharacterized protein | 3 | -0.82 | 47.0 | 7.12 |
| 1771 | I1KVT5 | uncharacterized protein | 2 | -0.82 | 15.5 | 5.76 |
| 1772 | I1JPD4 | eukaryotic translation initiation factor 3 subunit a | 24 | -0.82 | 111.9 | 9.33 |
| 1773 | A0A0R0J475 | uncharacterized protein | 11 | -0.82 | 79.2 | 7.39 |
| 1774 | I1MAX9 | uncharacterized protein | 5 | -0.83 | 46.0 | 5.90 |
| 1775 | I1KZW4 | uncharacterized protein | 11 | -0.83 | 88.9 | 5.06 |
| 1776 | I1J5R4 | uncharacterized protein | 2 | -0.83 | 25.9 | 4.77 |
| 1777 | I1JIE1 | ferritin | 12 | -0.83 | 27.7 | 5.64 |
| 1778 | I1M281 | uncharacterized protein | 5 | -0.83 | 66.2 | 5.18 |
| 1779 | C6TGU0 | dhar class glutathione s-transferase | 9 | -0.83 | 29.0 | 8.82 |
| 1780 | I1MYV1 | uncharacterized protein | 29 | -0.83 | 228.5 | 5.34 |
| 1781 | C6TH01 | uncharacterized protein | 3 | -0.83 | 29.3 | 6.61 |
| 1782 | I1LRC1 | uncharacterized protein | 11 | -0.83 | 62.5 | 9.21 |
| 1783 | I1KH38 | uncharacterized protein | 4 | -0.83 | 26.0 | 7.05 |
| 1784 | I1MZN4 | uncharacterized protein | 5 | -0.83 | 29.8 | 10.32 |
| 1785 | I1N0P5 | uncharacterized protein | 5 | -0.84 | 20.0 | 9.30 |
| 1786 | C6TMI3 | uncharacterized protein | 4 | -0.84 | 30.9 | 7.18 |
| 1787 | I1KG51 | uncharacterized protein | 4 | -0.84 | 84.8 | 5.16 |
| 1788 | I1MIW3 | ribokinase | 5 | -0.84 | 38.0 | 6.65 |
| 1789 | A0A0R4J3C5 | uncharacterized protein | 7 | -0.84 | 51.4 | 7.31 |
| 1790 | I1NJC2 | uncharacterized protein | 8 | -0.84 | 66.5 | 6.15 |
| 1791 | I1JP41 | uncharacterized protein | 3 | -0.85 | 49.2 | 4.72 |
| 1792 | I1NC12 | uncharacterized protein | 11 | -0.85 | 61.9 | 8.34 |
| 1793 | I1KZH4 | uncharacterized protein | 2 | -0.85 | 225.6 | 9.15 |
| 1794 | I1LTD1 | uncharacterized protein | 3 | -0.85 | 50.8 | 5.90 |
| 1795 | K7MLF2 | uncharacterized protein | 9 | -0.85 | 132.6 | 6.19 |
| 1796 | I1L0S5 | uncharacterized protein | 5 | -0.85 | 19.0 | 6.42 |
| 1797 | I1JR71 | importin subunit alpha | 17 | -0.85 | 58.8 | 5.23 |
| 1798 | I1KCD7 | uncharacterized protein | 33 | -0.86 | 90.9 | 5.11 |
| 1799 | C6TKA2 | uncharacterized protein | 2 | -0.86 | 36.0 | 7.60 |
| 1800 | K7M1C2 | uncharacterized protein | 10 | -0.86 | 129.0 | 6.19 |
| 1801 | K7LXR3 | uncharacterized protein | 6 | -0.86 | 83.1 | 6.46 |
| 1802 | C6TNT8 | uncharacterized protein | 8 | -0.86 | 43.0 | 7.66 |
| 1803 | I1LF33 | mitochondrial pyruvate carrier | 2 | -0.86 | 12.0 | 9.10 |
| 1804 | A0A0R0HL99 | uncharacterized protein | 30 | -0.86 | 237.3 | 5.44 |
| 1805 | I1LJA4 | uncharacterized protein | 15 | -0.87 | 60.8 | 9.07 |
| 1806 | A0A0R0I3N7 | uncharacterized protein | 4 | -0.87 | 52.6 | 9.10 |
| 1807 | I1LEV4 | uncharacterized protein | 22 | -0.87 | 90.2 | 6.03 |
| 1808 | A0A0R0GAV8 | uncharacterized protein | 10 | -0.88 | 92.0 | 8.63 |
| 1809 | A0A0R4J3N9 | uncharacterized protein | 9 | -0.88 | 43.9 | 6.18 |
| 1810 | C6T3Z7 | 40s ribosomal protein s8 | 7 | -0.88 | 29.1 | 10.26 |
| 1811 | I1JSN6 | uncharacterized protein | 2 | -0.88 | 19.9 | 5.90 |
| 1812 | I1JCA6 | uncharacterized protein | 9 | -0.88 | 58.0 | 4.70 |
| 1813 | I1NB78 | uncharacterized protein | 6 | -0.88 | 35.8 | 6.56 |
| 1814 | I1LMM9 | uncharacterized protein | 2 | -0.88 | 21.7 | 9.09 |
| 1815 | K7LAM2 | ubiquinone biosynthesis monooxygenase coq6 | 6 | -0.89 | 55.8 | 8.26 |
| 1816 | C6TEZ9 | uncharacterized protein | 3 | -0.89 | 29.9 | 5.43 |
| 1817 | B0M1A4 | catalase | 17 | -0.89 | 56.7 | 6.77 |
| 1818 | I1MEH7 | uncharacterized protein | 4 | -0.89 | 27.9 | 5.56 |
| 1819 | I1LBB9 | lipoxygenase | 17 | -0.89 | 98.1 | 5.62 |
| 1820 | C6SWE0 | uncharacterized protein | 8 | -0.89 | 17.4 | 5.41 |
| 1821 | I1KV87 | uncharacterized protein | 21 | -0.89 | 94.0 | 5.22 |
| 1822 | I1L1W0 | uncharacterized protein | 9 | -0.89 | 76.6 | 5.43 |
| 1823 | A0A0R0G8R3 | uncharacterized protein | 16 | -0.89 | 198.6 | 6.23 |
| 1824 | C6TGU2 | proteasome subunit alpha type | 10 | -0.90 | 26.0 | 4.70 |
| 1825 | I1KQJ6 | eukaryotic translation initiation factor 3 subunit c | 16 | -0.90 | 104.6 | 5.57 |
| 1826 | C6T9B1 | uncharacterized protein | 12 | -0.90 | 31.4 | 5.99 |
| 1827 | K7M3Y6 | uncharacterized protein | 12 | -0.90 | 45.9 | 6.10 |
| 1828 | P18663 | 50s ribosomal protein l2-a | 4 | -0.90 | 29.8 | 10.80 |
| 1829 | I1KYS4 | uncharacterized protein | 19 | -0.90 | 203.5 | 6.82 |
| 1830 | I1KC06 | uncharacterized protein | 5 | -0.90 | 60.8 | 5.97 |
| 1831 | C6TA41 | uncharacterized protein | 2 | -0.90 | 31.5 | 9.34 |
| 1832 | I1M6D0 | uncharacterized protein | 9 | -0.91 | 83.1 | 5.73 |
| 1833 | A0A0R0J7G8 | uncharacterized protein | 3 | -0.91 | 33.1 | 8.12 |
| 1834 | I1LS05 | uncharacterized protein | 10 | -0.91 | 45.8 | 6.57 |
| 1835 | I1LJE0 | adenylyl cyclase-associated protein | 7 | -0.91 | 50.6 | 6.34 |
| 1836 | A0A0R0E4D1 | uncharacterized protein | 3 | -0.91 | 78.1 | 9.65 |
| 1837 | I1LFD7 | uncharacterized protein | 14 | -0.91 | 134.9 | 5.35 |
| 1838 | C6SWX1 | uncharacterized protein | 3 | -0.91 | 12.0 | 10.59 |
| 1839 | I1MJB1 | proteasome subunit alpha type | 13 | -0.91 | 25.6 | 5.48 |
| 1840 | I1K3R6 | uncharacterized protein | 5 | -0.91 | 54.2 | 5.53 |
| 1841 | I1L122 | dna helicase | 3 | -0.92 | 95.6 | 5.69 |
| 1842 | I1KRU1 | uncharacterized protein | 6 | -0.92 | 44.3 | 6.33 |
| 1843 | C6TEF1 | uncharacterized protein | 4 | -0.92 | 40.8 | 6.93 |
| 1844 | I1KSB2 | uncharacterized protein | 6 | -0.92 | 49.1 | 7.00 |
| 1845 | I1M222 | uncharacterized protein | 9 | -0.92 | 25.6 | 5.50 |
| 1846 | I1KNP5 | endoglucanase | 4 | -0.92 | 68.7 | 8.87 |
| 1847 | I1MCM4 | uncharacterized protein | 4 | -0.92 | 48.1 | 4.82 |
| 1848 | I1ML09 | uncharacterized protein | 14 | -0.92 | 123.4 | 4.88 |
[truncated: 317,898 more chars]
