# Supplementary figures and images for: Proteomic Analysis of Irradiation with Millimeter Waves on Soybean Growth under Flooding Conditions
Source: Int J Mol Sci. 2020 Jan 12;21(2):486. doi: 10.3390/ijms21020486 (PMC7013696; doi:10.3390/ijms21020486)

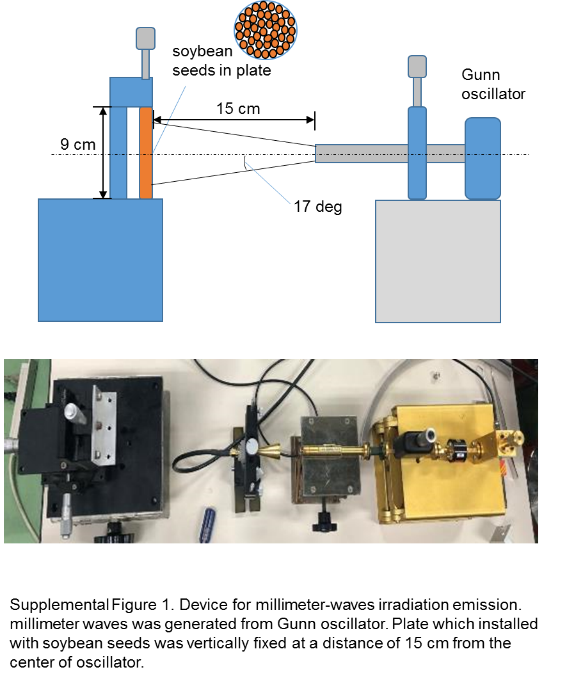


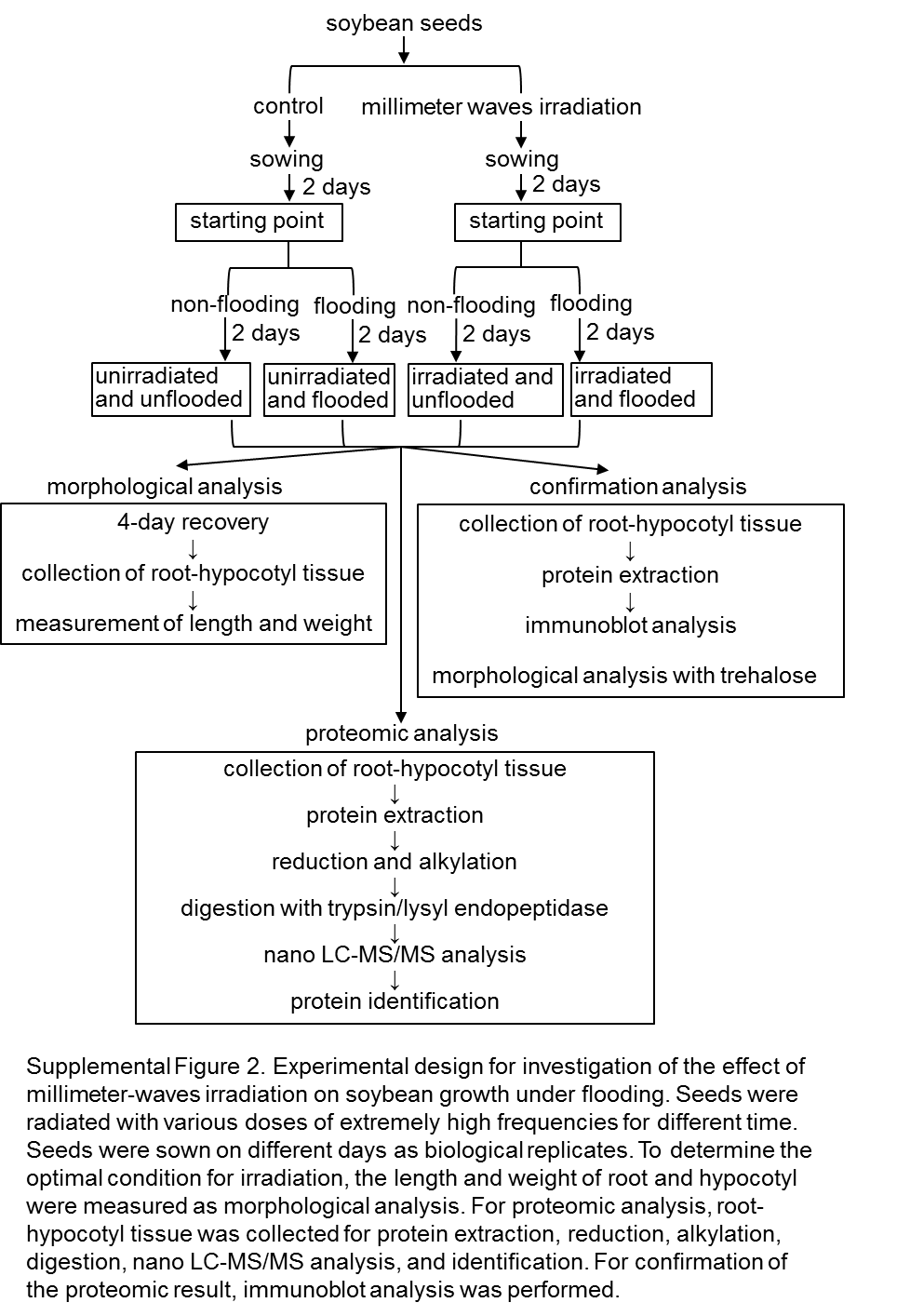


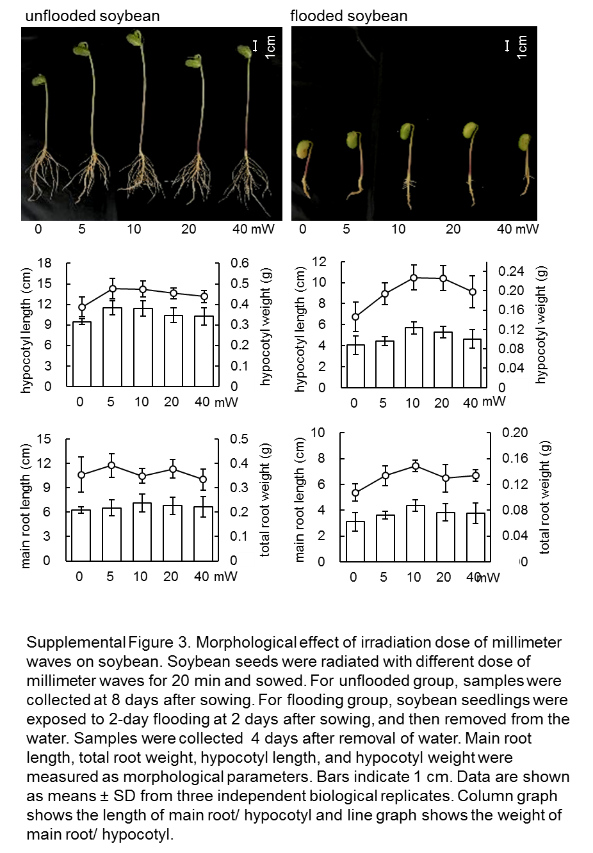


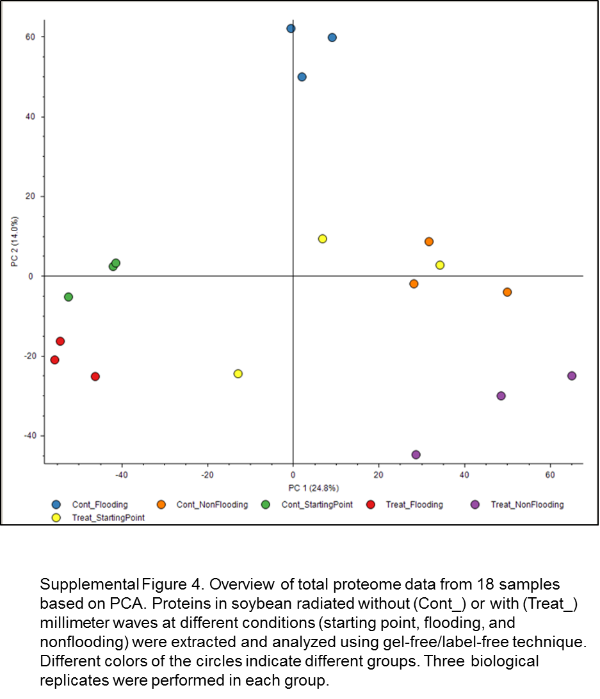


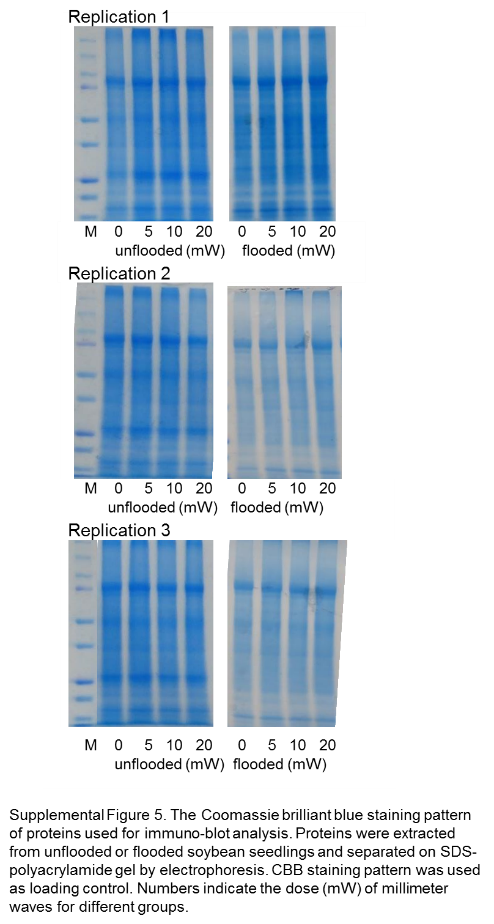


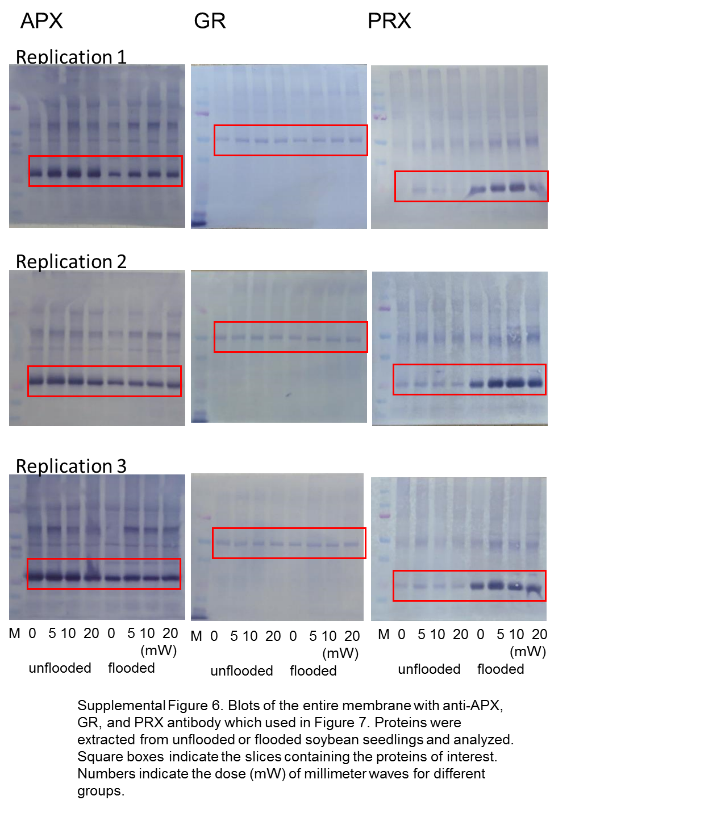


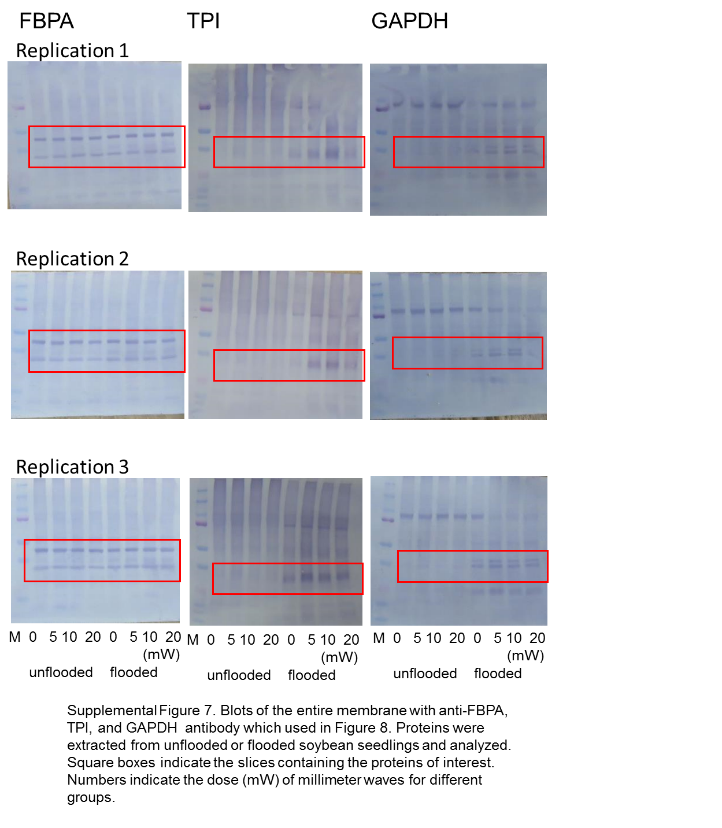

Supplement: Supplementary file 1 [file ijms-21-00486-s001.zip › rev Supplemental_Figures.docx]
